# A combined microphysiological-computational omics approach in dietary protein evaluation

**DOI:** 10.1038/s41538-020-00082-z

**Published:** 2020-12-17

**Authors:** Paulus G. M. Jochems, Willem R. Keusters, Antoine H. P. America, Pascale C. S. Rietveld, Shanna Bastiaan-Net, Renata M. C. Ariëns, Monic M. M. Tomassen, Fraser Lewis, Yang Li, Koen G. C. Westphal, Johan Garssen, Harry J. Wichers, Jeroen van Bergenhenegouwen, Rosalinde Masereeuw

**Affiliations:** 1 Division of Pharmacology, Utrecht Institute for Pharmaceutical Sciences, Utrecht University, Utrecht, The Netherlands; 2 Julius Centre, University Medical Center Utrecht, Utrecht, The Netherlands; 3 Wageningen Plant Research, Wageningen University, Wageningen, The Netherlands; 4 Wageningen Food & Biobased Research, Wageningen University, Wageningen, The Netherlands; 5 Danone Nutricia Research, Utrecht, The Netherlands; 6 Department of Orthopaedics, University Medical Center Utrecht, Utrecht, The Netherlands

**Keywords:** Biological techniques, Cell biology

## Abstract

Food security is under increased pressure due to the ever-growing world population. To tackle this, alternative protein sources need to be evaluated for nutritional value, which requires information on digesta peptide composition in comparison to established protein sources and coupling to biological parameters. Here, a combined experimental and computational approach is presented, which compared seventeen protein sources with cow’s whey protein concentrate (WPC) as the benchmark. In vitro digestion of proteins was followed by proteomics analysis and statistical model-based clustering. Information on digesta peptide composition resulted in 3 cluster groups, primarily driven by the peptide overlap with the benchmark protein WPC. Functional protein data was then incorporated in the computational model after evaluating the effects of eighteen protein digests on intestinal barrier integrity, viability, brush border enzyme activity, and immune parameters using a bioengineered intestine as microphysiological gut system. This resulted in 6 cluster groups. Biological clustering was driven by viability, brush border enzyme activity, and significant differences in immune parameters. Finally, a combination of proteomic and biological efficacy data resulted in 5 clusters groups, driven by a combination of digesta peptide composition and biological effects. The key finding of our holistic approach is that protein source (animal, plant or alternative derived) is not a driving force behind the delivery of bioactive peptides and their biological efficacy.

## Introduction

The ever-expanding world population goes hand in hand with an increase in food demand^1^. One of the primary components of the human diet are proteins. In contrast to other macronutrients, proteins’ primary function is not to provide energy, but proteins are involved in almost all biological processes^2^. Re-evaluating the over 90 million tons of food waste per year and exploring other protein sources may aid in identifying potential sustainable sources to assist in strengthening food security^3^. A relevant example is whey; first considered a waste product during cheese production, it is now one of the most abundant proteins in the modern Western diet. In 2013, global whey ingredient exports were estimated at 1.5 million metric tons/years^4^. Over the years, whey has been studied extensively and was found to contain a complex mixture of bioactive peptides^5^, involved in e.g. immune modulation, maintaining intestinal barrier integrity and regulation of satiety^6,7^. Human clinical trials have been used to collect data on protein evaluation, but such an approach is both expensive and cumbersome as a means of screening for new proteins.

In vitro models are cheaper, less time consuming and well suited for medium to high throughput screening^8^. These models could be an important supportive tool to (pre)select proteins for inclusion in a later clinical trial. A prototype of the gastrointestinal tract, where digestion takes place, is a first relevant model for evaluation, and the small intestine in particular, as the primary site of nutrient absorption. The small intestine has many key functions in addition to selective absorption via its epithelial barrier, e.g. finalizing the enzymatic digestion by the brush border enzymes and a first line of defense against pathogens^9^. Intestinal permeability is associated with both local (e.g. inflammatory bowel disease) and systemic (e.g. Parkinson’s) diseases^10,11^. To gain insights into these intestinal physiological processes, more complex, in vitro models have emerged as advanced technologies with the potential to improve in vitro*-*in vivo translation^12^.

In this study, bioengineered intestinal tubules were used as a microphysiological gut system and exposed to in vitro digested proteins followed by evaluation of key physiological parameters as described previously^13^. Three commonly used proteins sources (acidic WPC, egg, soy) and fourteen potential dietary proteins from a variety of sustainable sources (bovine blood plasma, insect (mealworm), pea, corn, wheat, fungi, yeast, potato) were studied and compared to a benchmark WPC. After static in vitro digestion, the digestive profile was evaluated via proteomics to determine the degree of overlap with WPC. The digests were then assessed for biological effects, *viz*. intestinal epithelial integrity, brush border enzyme activity, cell viability, cytokine, and chemokine release. A statistical cluster analysis was then applied to these data to identify commonalities and differences in physiological parameters across all the various protein sources.

## Results and discussion

### Proteomic data provide insights on dietary protein content and complexity

Details on the protein sources studied are given in Table 1. Acidic WPC (AWPC), a whey protein concentrate, is a byproduct of acidic dairy foods^14^. Due to the proteolysis by rennet, a complex mixture of enzymes, AWPC has a lower protein content in comparison to WPC^14^. The next commonly used protein is egg white (Egg), consisting mainly of water and approx. 11% of protein (77% ovalbumin, ovotransferin and ovomucoid)^15^. The third frequently used protein is soy, often used to replace animal-source proteins, but considered a less ideal protein for consumption due to its low methionine content^16,17^. In addition to these three commonly used proteins, we compared animal-based proteins from bovine blood plasma, plant-derived proteins from pea, wheat, potato, and corn, representing major food crops worldwide, and the alternative-source proteins derived from insect, fungi, and yeast^18^. Compositional data discussing percentages of carbohydrates, fats, proteins and kcal/100 g have been published before^19^. In Supplementary Table 1, additional data on percentage of fiber, moisture, ashes, and salt are provided.

**Table 1:** Overview of the 18 dietary proteins evaluated.

Full name	Protein source	Abbreviation	Protein origin
Digestive enzyme mixtures	–	Blank	–
Whey protein concentrate	Whey	WPC	Animal
Acidic whey protein concentrate	Whey	AWPC	Animal
Egg	Egg white	Egg	Animal
Soy	Soya bean	Soy	Plant
NPP	Pea	NPP	Plant
NWP	Wheat	NWP	Plant
Bovine plasma	Blood protein	BP	Animal
Collagen hydrolysate	Blood protein	CH	Animal
Lesser mealworm	Insect	LM	Alternative
Lesser mealworm concentrate 2	Insect	LMC2	Alternative
TPP1	Potato	TPP1	Plant
TPP2	Potato	TPP2	Plant
Quorn Mycoprotein	Fungi	QM	Alternative
Quorn ready to eat	Fungi/egg	QrtoE	Alternative
Wheat	Wheat	Wheat	Plant
Corn	Corn	Corn	Plant
Yeast	Yeast	YE	Alternative
Yesol	Yeast	Yesol	Alternative

After ingestion, protein digestion immediately starts and continues along the complete gastrointestinal tract. In vivo, peptides from dietary proteins can be formed through hydrolysis by enzymes released from the matrix itself or contaminating microbes, gastrointestinal digestive enzyme hydrolysis, and/or microbial fermentation^20^. It is important to note that during static in vitro digestion only gastrointestinal enzymes are taken into account (without brush border enzymes). Total protein concentration of evaluated protein sources was 2.9–14.8 mg/mL (avg. 9.4 mg/mL) (Supplementary Table 1).

Currently, dietary protein quality is primarily determined by the presence and level of essential amino acids for humans and its digestibility, absorbability, and utilization for metabolic functions^21^. In these experiments, digestibility was examined by a proteome analysis of the digested peptide mixture. Online databases are typically used to correlate data on digestion composition to bioactivity of peptides^22,23^. Based on homology analysis, functions of unknown peptide sequences can then be predicted; however, actual biological efficacy still remains to be determined. A limitation of this approach is that these databases hold data on a limited number of bioactive protein sequences from known sources. Our approach differs in that we compared complete peptide ion profiles generated by LC-MS. Comparison of alternative protein peptides with peptides from a well-studied protein source, viz. WPC, will result in increased understanding of the digestive profile and the potential to induce health benefits of the alternative protein hydrolysate. After enzymatic digestion and bioseparation, LC-MS ion-peak data of the filtrate, containing peptides likely to be absorbed in the small intestine, were used to compare WPC and the other dietary proteins based on relative molar mass (*m/z*), charge, retention time, and abundance. Each protein source was evaluated for the following characteristics: (a) fraction of protein digestive products (dividing di-, tri- and oligopeptides abundancy), (b) total peptide abundance (ranging from 0.12–1.99 × 10^8^), (c) unique peptides, i.e. non-overlapping with WPC (ranging from 65 to 436 peptide ions), (d) WPC peptide overlap relative to a total number of peptides (ranging from 7.8 to 82.6%), and (e) summed peptide abundance of WPC overlapping peptides relative to total peptide abundance (ranging from 5.7 to 95.4%) (Supplementary Fig. 1). The overlap of peptides of dietary protein digests versus WPC was visualized in tile plots (Supplementary Fig. 2), providing insights in the degree of overlap and peptide length. For each overlapping peptide, the abundance of that specific peptide was plotted in comparison to the WPC abundance (Supplementary Fig. 2). In addition, details on ion-peak data of overlapping and non-overlapping peptides are shown in Supplementary Table 2. For a holistic evaluation of the dietary proteins in comparison to WPC, these data were used in a statistical cluster analysis (absolute values are shown in Supplementary Fig. 1). Proteomic data were clustered using the Bayesian Information Criterion (BIC), a standard goodness of fit metric used here to determine the optimal clustering model supported by the data. Fourteen different clustering models were compared resulting in an ellipsoidal, equal volume and shape and variable orientation (EEV) model with three distinct clusters as the best fitting model (BIC results Supplementary Fig. 3)^24^.

**Fig. 1:**
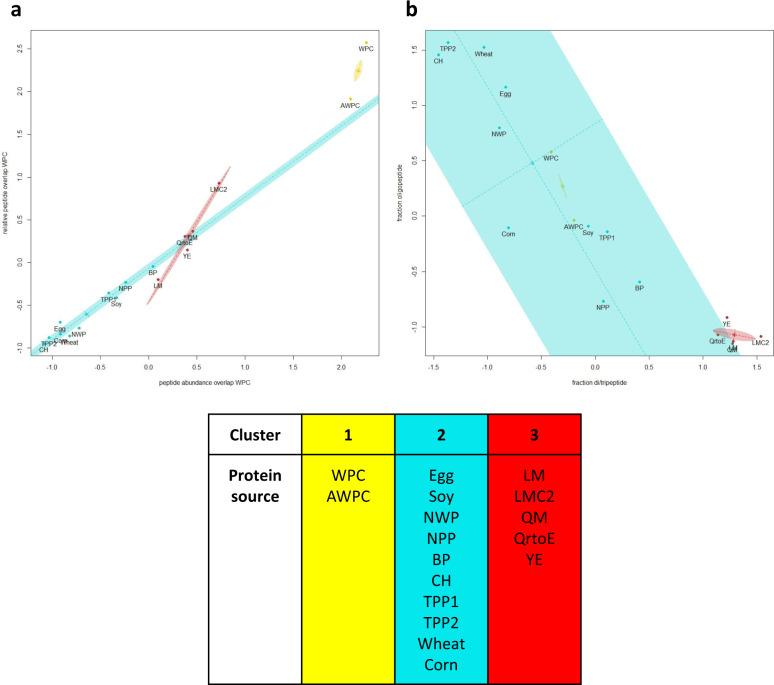
Holistic clustering of potential dietary protein based on proteomic data. The optimal cluster method ellipsoidal, equal volume, shape, and variable orientation (EEV) and number of clusters (3) were determined using the Bayesian Information Criterion. **a** This two-component plot shows relative peptide overlap to total peptide count in WPC on the vertical axis and relative peptide abundance of WPC overlapping peptides on the horizontal axis. **b** Two-component plot showing fraction oligopeptides on the vertical axis and fraction di/tripeptides on the horizontal axis. Supplementary Fig. 4 shows all combinations of two-component plots. Analysis was done based on digest peptide profiles, ion peak overlap, quantity of WPC-matched ion peaks relative to total protein ion peaks and relative abundance of WPC-matched ion peaks.

All combinations of two characteristics plots were designed (Supplementary Fig. 4), of which two examples are shown in Fig. 1. As expected, AWPC protein content showed great similarity to WPC (82.6% peptide overlap to WPC representing 95.4% of AWPC its total peptide content) and as result its direct clustering (Fig. 1, cluster 1). Both animal and plant-sourced proteins, namely Egg, Soy, NWP, NPP, BP, CH, TPP1, TPP2, wheat, and Corn clustered together (Fig. 1, cluster 2). Further, alternative-sourced proteins, LM, LMC2, QM, QrtoE, and YE clustered (Fig. 1, cluster 3). Protein sources in cluster 3 have, in comparison to protein sources in cluster 2, high overlap levels to WPC (Fig. 1a), a high fraction of di/tripeptides and a low fraction of oligopeptides (Fig. 1b) and can therefore be distinguished.

To further compare the concentration of overlapping peptides with WPC, their abundance was plotted for each overlapping peptide (Supplementary Fig. 2). Identification of native digests peptide sequences is not trivial, as protein database search algorithms are mostly developed for longer peptide sequences, and preferentially use a minimum sequence length of 5 or 6 amino acids^25^. As little is known with respect to alternative protein sources, a well-characterized reference protein is instrumental for evaluation. Proteomic analysis focus on global characteristics (peptide length, abundance and distribution) rather than individual peptide sequence. Therefore, de novo interpretation of MS/MS spectra provides a valid alternative approach. However, this depends on sufficient fragmentation information available in the MS/MS spectra, which is not available for all peptide ions. As a proof of concept, WPC peptides that showed overlap with another dietary protein were, if possible, linked to a specific peptide sequence using de novo MS/MS spectrum interpretation^26^ (Table 2).

**Table 2:** Identified peptide sequences with at least a de novo score of >90.

Peptide sequence	#ID	Presence in protein source
Leu-Lys (LK)	33	AWPC, Egg, Soy, NPP, BP, LM, QrtoE, TPP1, TPP2, YE
Asp-Lys (DK)	37	AWPC, Egg, QM, QrtoE, Corn
Val-Arg (VR)	43	AWPC, Egg
Met-Lys (MK)	50	AWPC, Soy, NPP, BP, LM, TPP1, Corn, Wheat, YE
Met-Ala-Pro-Lys (MAPK)	128	AWPC

It is important to note that the de novo identification method is not capable of distinguishing isomeric amino acids (leucine and isoleucine)^27,28^. In total, 323 peptides sequences were identified with a range of 0–98.1 for the de novo sequence score. To ensure a reliable identification, only de novo sequence scores >90 are discussed (Table 2; abundance shown in Supplementary Fig. 5). First, identified sequences LK, MK and MAPK form parts of known antihypertensive peptides LKP (β-lactoglobulin), MKP (α_s2_-casein f) and MAP (β-casein f) present in whey protein^29–31^. Furthermore, VR has been identified as a peptide sequence with angiotensin-converting-enzyme inhibitory capacity^32^. Hence, these peptide sequences might have positive cardiovascular health effects by inhibiting angiotensin I conversion into angiotensin II, preventing vasoconstrictive effects^33^. Before dietary peptides can induce systemic health effects, these must be absorbed in the small intestine. Therefore, their biological efficacy was tested using bioengineered intestinal tubules (Supplementary Fig. 6).

### Cluster analysis distinguishes biological behavior of potential dietary proteins

Protein sources are inherently contaminated with microbial products, such as endotoxins, of which translocation over the epithelial barrier can initiate a systemic immune response^34^. Nevertheless, endotoxin contamination in human nutrition is not subject to legislation. In addition, the small intestine is continuously exposed to endotoxins originating from its own microbiome and acquires tolerance immediately after birth by *e.g*. downregulating interleukin 1 receptor-associated kinase 1^35^. Often applied in vitro digestion methods use chemicals, which result in increased endotoxin contamination in the protein digests that in this study varied between 10.4->65.0 × 10^3^ EU/mL (Supplementary Table 1). However, blank digest did not show any effects on the small intestinal features evaluated (Supplementary Fig. 7), providing evidence that the enzymes and associated endotoxin content had no detrimental effects on the epithelial barrier integrity, cell viability, and alkaline phosphatase activity in our bioengineered intestinal tubules.

**Fig. 2:**
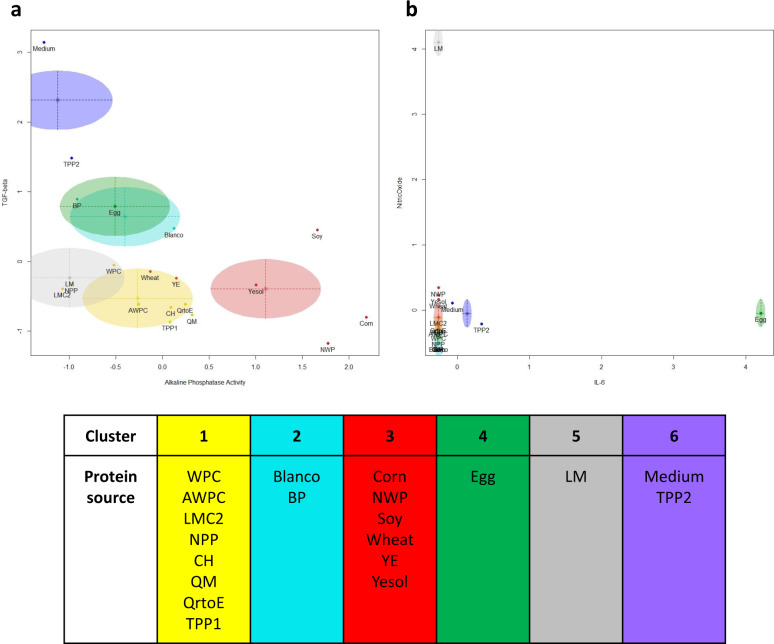
Holistic clustering of potential dietary protein based on intestinal biological efficacy data. The optimal cluster method ellipsoidal, equal volume, shape and orientation (EEE) and number of clusters (6) were determined using the Bayesian Information Criterion. **a** Two-component plot showing TGFβ secretion (vertical axis) and alkaline phosphatase activity (horizontal axis). **b** two-component plot showing nitric oxide (vertical axis) and IL-6 (horizontal axis). Supplementary Fig. 9 shows all combinations of two-component plots. Analysis was done based on inulin-FITC leakage, zonula occludens-1 (ZO-1) expression quantification, tissue viability, alkaline phosphatase activity, IL-6 secretion, TGF-β secretion, and NO production.

Experiment-ready bioengineered intestinal tubules were exposed to dietary protein digests and the impact on epithelial barrier integrity, brush border enzyme activity and cell viability were assessed (Supplementary Fig. 7). The biological data set was further supplemented by dietary protein-induced release of immune markers (*viz*. IL-6, TGFβ, and NO) (Supplementary Fig. 8). The complete set of biological read-outs were clustered using the Bayesian Information Criterion (BIC), a standard goodness of fit metric, used here to determine the optimal clustering model supported by the data. This resulted in an equal shape and volume axis parallel orientation (EEI) model with 6 distinct clusters as best fit (Supplementary Fig. 9)^24^. All combinations of two components plots were plotted (Supplementary Fig. 10), of which two examples are shown in Fig. 2.

In a subsequent analysis, dietary proteins were compared to the reference protein WPC, which did not show a significant difference for most parameters tested (Supplementary Fig. 7). However, analysis of the biological data using a non-biased cluster-based approach demonstrated apparent differences. The first and largest cluster contains WPC together with proteins originating from animal (WPC, AWPC, CH), plant (NPP and TPP1), and alternative sources (LMC2, QM, and QrtoE) (Fig. 2, cluster 1). These proteins showed average levels of intestinal physiological parameters and low levels of cytokine and chemokine secretion/production. Cluster 2 includes the digestion mixture (blank digest) and BP, primarily based on their relatively high levels of ZO-1 expression and average cell viability (Supplementary Fig. 10, vertical-axis: zonula occludens-1, horizontal axis: cell viability). The blank digest did significantly decrease TGFβ and NO secretion, as reported earlier for TGFβ, nitrate and nitrite (as measure for NO)^36–38^. Corn, NWP, Soy, and Wheat (plant-source) next to YE and Yesol (alternative-source) form the second largest cluster (Fig. 2, cluster 3). Cluster 3 is characterized by its relatively high alkaline phosphatase activity (Fig. 2a). Egg forms its own separate cluster because it significantly induces IL-6 secretion (Fig. 2b, cluster 4). Next to Egg, only medium and TPP2 also induced IL-6 secretion (Fig. 2b), however, these levels were not sufficient to support clustering together with Egg, and other variables, i.e. relative high TGFβ (Fig. 2a), accounted for the formation of a separate cluster (Fig. 2, cluster 6). Even though IL-6 concentrations were increased, these remained under an epithelial barrier disruptive concentration (1 ng/mL), confirmed by intestinal epithelial integrity assays^39^. However, IL-6 itself can induce intestinal inflammation by provoking a T-cell response and IgG secretion by B-cells, and is associated with a variety of local and systemic inflammatory disease^40,41^. The ability to induce an immune response by increasing IL-6 levels in Egg and TPP2 digest exposure requires further research. LM clustered separately, primarily due to its induction of high levels of NO (Fig. 2b, cluster 5; Supplementary Fig. 8b), although these levels (avg. 155.4 µM) did not affect the epithelial barrier (Supplementary Fig. 7a, b). NO is able to induce beneficial regulatory effects for essential intestinal features, such as maintaining an intestinal epithelial barrier, but increased levels are associated with pathology in *e.g*. inflammatory bowel disease^42,43^. Its dual effect in vivo challenges translational interpretation and warrants further research focusing on the downstream effects of increased NO levels upon LM digest exposures.

### Statistical cluster analysis based on the proteome and biological efficacy reveals that protein source is not a driving force behind the delivery of biological effects

**Fig. 3:**
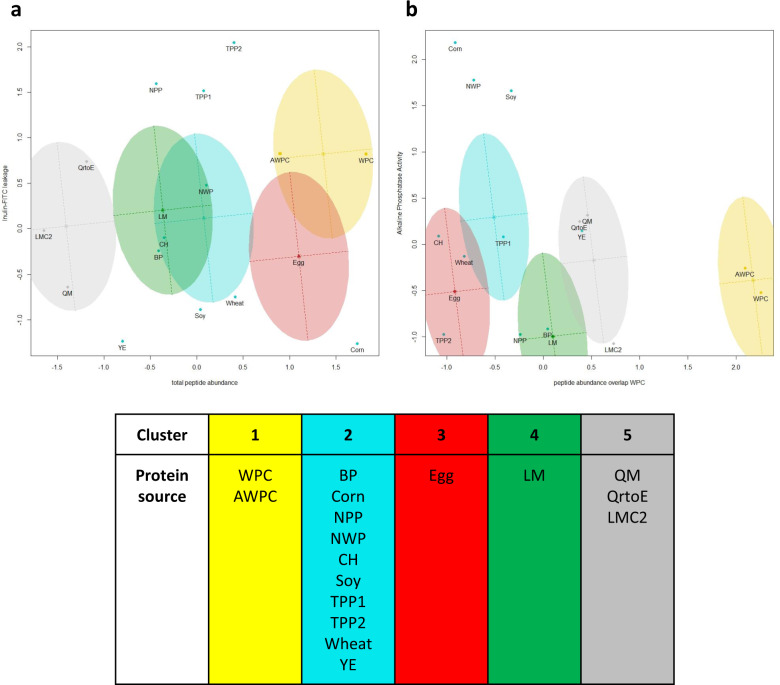
Holistic clustering of potential dietary protein based on proteomic and intestinal biological efficacy data. The optimal statistical cluster model ellipsoidal, equal volume, shape, and orientation (EEE) and number of clusters (5) were determined using the Bayesian Information Criterion. Considered data were peptide abundance, total amount of unique peptides, relative overlap with whey protein concentrate (WPC), the relative peptide abundance of the WPC overlap, inulin-FITC leakage, ZO-1 quantification, cell viability, alkaline phosphatase activity, IL-6, TGFβ, and nitric oxide. **a** two-component plot showing inulin-FITC leakage assay on the vertical axis and total peptide abundance on the horizontal axis. **b** two-component plot showing alkaline phosphatase activity assay on the vertical axis and relative peptide abundance of WPC overlapping peptides on the horizontal axis. Supplementary Fig. 11 shows all combinations of two-component plots. The division of potential dietary proteins in the different clusters is shown below the two two-component plots. Analysis was done based on proteomic data and biological efficacy data.

To create a holistic overview, both the proteomic and biological efficacy data were combined in a cluster analysis. Based on the BIC, an ellipsoidal equal volume, shape, and orientation (EEE) statistical cluster model with 5 distinct clusters had the best fit (Supplementary Fig. 11)^24^. All combinations of two components plots were plotted (Supplementary Fig. 12), of which two examples are shown in Fig. 3.

The emphasis during this clustering analysis was placed automatically on biological efficacy as these parameters outnumbered proteomic parameters in a 2:1 ratio. Placement of WPC and AWPC (Fig. 3, cluster 1) together is thought to be primarily driven by the high level of peptide overlap in proteomic data (Fig. 3b), as no significant differences in biological efficacy were detected. This underscores the potential of AWPC as an alternative to WPC in providing dietary proteins. However, the production process still requires optimization^14^. The largest cluster consists of animal-based protein sources, BP and CH, plant-based sources, Corn, NPP, Soy, TPP1, TPP2, and Wheat, and alternative protein source, YE (Fig. 3, cluster 2). Again, the protein source does not seem to be the driving force for cluster membership of proteomic data nor biological efficacy. Interestingly, a wide variety of protein sources clustered together with the common animal-protein replacer Soy. Hence, these plant-based and alternative protein sources could potentially replace soy. The separate clusters of Egg (Fig. 3, cluster 3) and LM (Fig. 3, cluster 4) are driven by their significant induction of IL-6 (Supplementary Fig. 12, IL-6 on vertical or horizontal axis) and NO (Supplementary Fig. 12, NO on vertical or horizontal axis), respectively. QM, QrtoE, and LMC2 (Fig. 3, cluster 5) did not show differences in biological efficacy and are grouped based on their relatively small peptide content (Fig. 3a) resulting in a high degree of WPC overlap (Fig. 3b). The statistical model-based clustering on proteomics and in vitro biological efficacy data showed capable to distinguish dietary protein characteristics in both datasets, as also clusters driven by either proteomics or in vitro biological efficacy appeared confirming the validity of our approach.

Altogether, By combining microphysiological intestinal evaluation with model-based statistical clustering we generated unique holistic characterizations of eighteen dietary proteins with their digests across proteomic and in vitro biological efficacy domains. This characterization can provide detailed information on a broad spectrum of dietary proteins, i.e. peanut, fish, and algae, in the future.

As a first step in evaluating alternative dietary proteins, we demonstrated that these proteins do not cluster together consistently according to either proteomic or biological efficacy attributes. But, rather individual cluster membership could be driven by either individual or both domains. Neither is the type of protein source a driver behind cluster membership, and therefore similarity across proteomic or biological efficacy attributes.

Our results clearly highlight the complexity and potential value of carefully characterizing dietary proteins using a holistic methodology. This approach may help in increasing the likelihood of identifying new protein sources capable of delivering tangible health benefits and food security.

## Methods

### Chemicals

All chemicals were purchased from Sigma-Aldrich (Zwijndrecht, The Netherlands) unless stated otherwise.

### Dietary proteins

A total of 18 dietary proteins were in vitro digested and evaluated. WPC was used as primary reference protein for 17 sustainable other dietary proteins. These sustainable proteins had a wide variety of protein sources (Table 1).

### Static in vitro digestion

The static in vitro digestions were performed according to the widely accepted INFOGEST consensus protocol^44^. To allow the use of the digestions in cell culture models, the pancreatin enzyme mixture was adjusted to a Trypsin activity at 10 U/ml. After the intestinal incubation phase, samples were aliquoted and snap frozen in liquid nitrogen and stored at −80 for subsequent measurements. Protein content in the digests as assessed in triplicate on a Flash EA 1112 GC (Interscience BV, Breda, The Netherlands) according to the Dumas method^45^. Methionine and cellulose were used as a standard and negative control respectively. Endotoxin content was determined by the EndoZyme II kit according to the manufacturer’s instructions (Biomérieux company, München, Germany). Results were considered valid if the endotoxin spike recovery was in the range of 50 to 200 %. Fluorescence readings were performed on an Infinite F200 plate reader (Tecan Group Ltd., Männedorf, Switzerland). When samples displayed fluorescence overflow values, the endotoxin content was set to >65,000 EU/ml. Next, digested samples were used for proteomics or exposure to the bioengineered intestinal tubules.

### Proteomics

Proteomic analysis has only been conducted on the small intestine absorbable peptide fraction. To that end, the digests were separated by an Amicon® Bioseparations Stirred Cells device over a 1 kDa ultrafiltration disc (Merck Millipore; Amsterdam, The Netherlands) into a retentate and filtrate. In vitro protein digests were analyzed on a UPLC-MS system (Dionex Ultimate 3000 online connected to Qexactive^PLUS^ (Thermofisher, Waltham, U.S.A). Samples were injected (10microliter) on a pentafluorophenyl F5 core-shell column (Kinetex F5, 15 cm × 2.1 mm, 2.6 micrometer particles, Phenomenex, Torrance, USA) operated at 30 °C and flow rate of 0.2 ml per minute. Total run time was developed over a 40 min time window: Starting with buffer A (0.1% formic acid in water) for 5 min and separated with a gradient of 0 to 30% buffer B (0.1% formic acid in 100% Acetonitrile) during 20 min, increasing to 80% B in 5 min, stable at 80% B for 3 min and back to 0% B during 2 min, and stable at 0% B for 5 min. Separated peptides were on-line injected into the Q Exactive^PLUS^ using the standard ESI source in positive mode, with 3.5 kV spray voltage, 290 °C capillary temperature, nitrogen sheath gas flow 40 and auxiliary gas flow 10 heated at 60 °C. MS spectra were collected with alternating scans; first within a *m/z* range of 70–380, followed by MS scan within a range of 350–1200 *m/z* at 70000 resolution (profile) and AGC target of 3 × 10^6^ ions maxIT for 100 ms; followed with data-dependent switch to MSMS mode at 17,500 resolution (centroid) at AGC target 10^5^ ions, minimum AGC 8 × 10^3^, maxIT 50 millliseconds and 4 *m/z* isolation window, loop count 5, a dynamic exclusion of 10 s without further charge exclusion.

### Caco-2 cell culture

The human colon adenocarcinoma-derived intestinal cell line, Caco-2, (ATCC, Wesel, Germany) were maintained in high glucose Dulbecco’s Modified Eagle Medium–high glucose (Gibco, Bleiswijk, The Netherlands) supplemented with fetal calf serum (10% v/v) and penicillin and streptomycin (1% v/v). Media was refreshed every 2–3 days, and cells were passaged and seeded on bioengineered intestinal tubules when reaching 80–90% confluency.

### Bioengineered intestinal tubule construction, coating, seeding, and cultivation

Three-dimensional polylactide chambers (schematic illustration in Supplementary Fig. 13) were printed with the Ultimaker 3 (Ultimaker, Geldermalsen, The Netherlands). Thereafter, SENUOfil type H-MF-0.2 hollow fiber capillary membranes (SENUOFIL, Tianjin, China) were cut and guided through the chamber using a steel wire. 18 g blunt needles 0.5-inch length (OctoInkjet, Hoyland, United Kingdom) were put in the in- and outlet of the chambers and made leak tight using Loctite EA M-31CL glue (Henkel Adhesives, Nieuwegein, The Netherlands). Finally, the bottom of the chamber was sealed with a 24 × 60 mm glass cover slip (Menzel-Gläser, Braunschweig, Germany) using Loctite EA M-31CL glue. Glue dried for 24 h and GI-MASK Automix glue (Coltene, Lezennes, France) was used to make the inner parts of both syringes leak tight.

After chamber construction, extracellular matrix (ECM) coating and cell seeding were executed directly in the 3D-chambers. After 30 min of sterilization in 70% (v/v) EtOH followed by 5 h filter-sterilized L-3,4-di-hydroxy-phenylalanine (L-Dopa, 2 mg mL^−1^ in 10 mM Tris buffer, pH 8.5) followed by 2 h human collagen IV solution (25 µg mL^−1^ in PBS) to complete the ECM-coating. In between each step, the chamber was washed 3× using PBS. Caco-2 cells were trypsinized and seeded in the chambers for 4 h at a seeding density of 1 × 10^6^ Caco-2 cells/bioengineered intestinal tubule.

Bioengineered intestinal tubules were cultivated for 21 days in a 5% CO_2_ and 37 °C incubator, and culture medium was refreshed every 2–3 days. During the final 7 days, chambers were put on a two-dimensional rocking platform (VWR, Breda, The Netherlands) with a speed rate of 1 rotation per minute at an angle of 10°. Thereafter, bioengineered intestinal tubules were experiment ready.

### Protein digest exposures

Protein digests were diluted 1:4 with culture medium and bioengineered intestinal tubules were exposed to 1 mL for 3 h. Thereafter, bioengineered intestinal tubule were assessed on barrier integrity, brush border enzyme activity, epithelial cell viability, and secretory markers.

### Inulin-FITC leakage assay

To quantify the paracellular permeability, bioengineered intestinal tubules were perfused with inulin-FITC (0.1 mg.ml^−1^ in PBS). Prior to perfusion, bioengineered intestinal tubules were washed and placed in PBS. Then, chambers were connected to a Reglo Independent Channel Control pump (Ismatec, Wertheim, Germany) and perfused with inulin-FITC for 10 min at a flow rate of 0.1 mL/min. PBS from the chamber was collected, homogenized and fluorescence was measured at excitation wavelength of 492 nm and emission wavelength of 518 nm using Tecan infinite M200PRO plate reader (Tecan Austria GmbH). Results are shown relative to medium control. After the leakage assay, bioengineered intestinal tubules were washed in wash buffer (4% FCS in HBSS (v/v)) and cut into three pieces of which one was used for immunostaining and two for cell viability and alkaline phosphate activity.

### Immunofluorescent staining and zonula occludens-1 quantification

To investigate the expression levels of zonula occludens-1 bioengineered intestinal tubules were immunofluorescently stained. First, cells were fixed (60% EtOH, 30% chloroform and 10% acetic acid (v/v)) for 5 min, permeabilized (0.3% (v/v) Trition X-100 in HBSS) for 10 min and blocked ((2% (w/v) bovine serium albumin (BSA) fraction V and 0.1% (v/v) Tween-20 in HBSS) for 30 min in between steps bioengineered intestinal tubules were washed using wash buffer. Thereafter, bioengineered intestinal tubules were incubated with zonula occludens-1 (ZO-1) primary antibody (1:1000 diluted in blocking solution) (Thermo Fisher Scientific, Bleiswijk, The Netherland) for 2 h. After washing, secondary antibody goat-anti-rabbit 594 (1:200) (Abcam, Cambridge, United Kingdom) was added. Finally, bioengineered intestinal tubules were mounted using Prolong gold containing DAPI (Cell signaling technology, Leiden, The Netherlands) for nuclei staining. Images were acquired using the Leica TCS SP8 X (Leica Biosystems, Amsterdam, The Netherlands).

For image analyses Fuiji ImageJ was used and a full field of view image was required (Supplementary Methods 1 provides a step-by-step protocol, including representative bioengineered intestinal tubules images). Maximum intensity projections were made of the bioengineered intestinal tubules, channels were separated. ZO-1 intersections were determined using a ROI manager, lines were drawn over the area of interest. Values were corrected for background and the number of intersections per line were counted, providing a quantitative measure for ZO-1. The number of nuclei was counted via a particle size measurement. Thereafter, each value was corrected for surface area expressed in micron^2^.

### Cell viability

As a measure for cell viability, the mitochondrial activity was measured via PrestoBlueTM cell viability reagent assay (Thermo Fisher). PrestoBlue^TM^ reagent was mixed with culture medium at a 1:10 ratio and 100 µL was put on a part of the bioengineered intestinal tubules in a 96 wells plate. The plate was placed in an incubator at 5% CO_2_ and 37°C and incubated for 1 h protected from light. Bioengineered intestinal tubules were removed, and fluorescence was measured at excitation wavelength of 530 nm and emission wavelength of 590 nm using Tecan infinite M200PRO plate reader. The positive control, unexposed bioengineered intestinal tubule, was set to 100% viability.

### Brush border enzyme activity

After the cell viability assay, bioengineered tubules were washed with PBS and alkaline phosphatase activity was measured using Amplite^TM^ Colorimetric Alkaline Phosphatase Assay kit (AAT Bioquest, Sunnyvale, United States). Assay was performed according to manufacturer protocol. Bioengineered intestinal tubules were exposed to the reaction mixture for 30 min and absorbance was measured at 600 nm using colorimetric plate reader (iMARK™ microplate absorbance reader, Bio-Rad, Veenendaal, The Netherlands). Values are shown relative to the medium exposed.

### Immune response

After exposures, supernatant was collected and IL-8 (BioLegend, London, United Kingdom), IL-6 (Biolegend) and TGF-β (Biolegend) were quantified via ELISA. ELISAs were executed according to manufacturer protocol. First, plates were coated and incubated overnight. Thereafter, plates were blocked for 1 h and incubated with samples for 2 h. This was followed by detection antibody incubation for 1 h and Avidin-HRP for 30 min. Wells were exposed for 15 min to substrate solution followed by stop solution. Absorbance was read at 450 nm. Wells were washed in between exposures. Values were corrected for background using the supernatant of the unseeded no-ECM coated bioengineered intestinal tubules, specific for each data set.

NO was determined via Griess reaction (Promega, Leiden, The Netherlands) according to manufacturer protocol. In short, samples were centrifuged for 3 min at 5120 × *g* and supernatants were transferred to a 96 wells plate. Sulfanilamide solution was added to all wells and incubated for 10 min. Thereafter, NED solution was added and incubated for 10 min. Followed by an absorbance measurement at 490 nm.

### In vitro biological data analysis

Every experiment was at least performed in triplicate. Data were analyzed for outliers using Grubbs test, α = 0.05. Statistical analysis was performed in Graphpad version 8 using t-test and one-way ANOVA. A *P*-value of < 0.05 was considered significant.

### Proteomic analysis

LC-MSMS data were loaded per replicate of protein source sample into Progenesis QI software (Non-linear Dynamics, Waters BV, UK). Peak detection was performed with a sensitivity setting of 4 and minimum peak length of 2 s. Peptide ion data were exported to a comma separated file per replicate of protein source sample. Individual peptide ion tables were further filtered and processed in Excel and R-studio.

A total of 422697 ion peaks (over all peptide ion tables) were observed and corrected for background noise. First, data with a raw abundance <1 × 10^5^ were excluded, excluding 414,436 ion peaks. Second, all dietary protein digesta were corrected for digestive enzymes by excluding overlapping ion peaks with the blank. Criteria for matching ion peaks between separate samples were based on absolute differences in charge, *m/z* (±0.003) and retention time (±0.5 min). Matching to Blank, resulted in 3542 ion peaks exclusion, maintaining a total of 4719 ion peaks divided over the eighteen protein digests for further analysis.

All protein digesta were matched to WPC by the matching criteria. If an ion-peak matched to multiple WPC ion peaks, the nearest ion-peak was defined based on the two-dimensional space regarding *m/z* and retention time, weighing both criteria equally (resulting in 0.003 *m/z* = 0.5 min retention time). The distance was measured using Pythagoras equation (see Eq. 1), as shown in the formula. The match with the smallest distance was then selected.

1 $${\rm{Distance}} = \sqrt {\left( {{\Delta}m\!/\!z \times \left( {\frac{{0.5}}{{0.003}}} \right)} \right)^2 +\, {\Delta}{\rm{Retention}}\,{\rm{time}}^2}.$$

If a protein had multiple ion peaks matched to the same ion-peak in WPC, their abundance was shown as total sum.

### Computational statistical model clustering

Proteomics and in vitro biological efficacy data were processed using R version 3.5.1 (packages data.table, ggplot2, Mclust & dplyr). Means across replicates were computed for each variable and then standardized to standard deviation one and mean zero. The Mclust package was used to fit clustering models to the data with the best fitting model chosen using the Bayesian Information Criterion (BIC), (R-script, see code availability statement). These clusters provide a two-dimensional holistic representation of dietary protein properties.

## Supplementary information

Supplementary Files

## Data Availability

The data that support the findings of this study are available from the corresponding author upon reasonable request. Proteomic data are accessible in PRIDE, project name: Dietary protein in vitro digest (LC-MS), project accession: PXD021577.

## Code availability

Custom code available at github.com respiratory PaulJochems/FutureProtein.
