# Supplementary material for: A combined microphysiological-computational omics approach in dietary protein evaluation
Source: NPJ Sci Food. 2020 Dec 17;4:22. doi: 10.1038/s41538-020-00082-z (PMC7746769; doi:10.1038/s41538-020-00082-z)
Supplement: Supplementary file 1 — Supplementary Files [file 41538_2020_82_MOESM1_ESM.pdf]

Supplementary table 1. Overview of the 18 dietary proteins evaluated including protein concentration based on DUMAS method and endotoxin content.

Full name	Protein Source	Abbreviation	Distributor	Fibre (%)	Moisture (%)	ashes (%)	salt (%)	Protein concentration in digest (mg/mL)	Endotoxin (EU/mL*)
Digestive enzyme mixtures	-	Blank		N/A	N/A	N/A	N/A	7.7 ± 0.4	43374
Whey protein concentrate	Whey	WPC	Danone Nutricia Research	N/A	6	4	N/A	12.8 ± 0.0	56019
Acidic whey protein concentrate	Whey	AWPC	Danone Nutricia Research	N/A	4.5	4	N/A	11.6 ± 0.4	57911
Egg	Egg white	Egg	Bulk Powders	0	N/A	N/A	3.12	11.3 ± 0.1	>65000
Soy	Soya bean	Soy	Bulk Powders	0	N/A	N/A	1.67	10.0 ± 0.3	>65000
NPP	Pea	NPP	Roquette	1	7	4	N/A	9.9 ± 0.6	>65000
NWP	Wheat	NWP	Roquette	1	5	1	N/A	11.4 ± 0.2	>65000
Bovine plasma	Blood protein	BP	Darling Ingredients	0	8	16	15	9.8 ± 1.8	13477
Collagen hydrolysate	Blood protein	CH	Darling Ingredients	0	7.5	0.73	1.4	14.8 ± 0.2	63077
Lesser mealworm	Insect	LM	Proti-Farm	63	3	3	0.15	8.7 ± 0.5	55907
Lesser mealworm concentrate 2	Insect	LMC2	Proti-Farm	N/A	N/A	N/A	N/A	11.2 ± 0.1	61471
TPP1	Potato	TPP1	Avebe	0	7	<5	0	4.4 ± 0.6	>65000
TPP2	Potato	TPP2	Avebe	0	7	<5	2	2.9 ± 0.2	10391
Quorn Mycoprotein	Fungi	QM	Quorn Foods	29	N/A	N/A	<0.01	11.7 ± 3.5	>65000
Quorn ready to eat	Fungi/egg	QrtoE	Quorn Foods	7.1	N/A	N/A	0.8	6.1 ± 0.5	>65000
Wheat	Wheat	Wheat	Cargill	1	5	1	0.25	4.9 ± 0.8	61577
Corn	Corn	Corn	Cargill	6.5	4	4.5	<0.5	9.1 ± 0.6	>65000
Yeast	Yeast	YE	Lesaffre	7.7	5.6	2.7	N/A	7.9 ± 0.5	>65000
Yesol	Yeast	Yesol	Lesaffre	N/A	N/A	N/A	N/A	12.3 ± 0.1	>65000

*endotoxin units per milliliter.

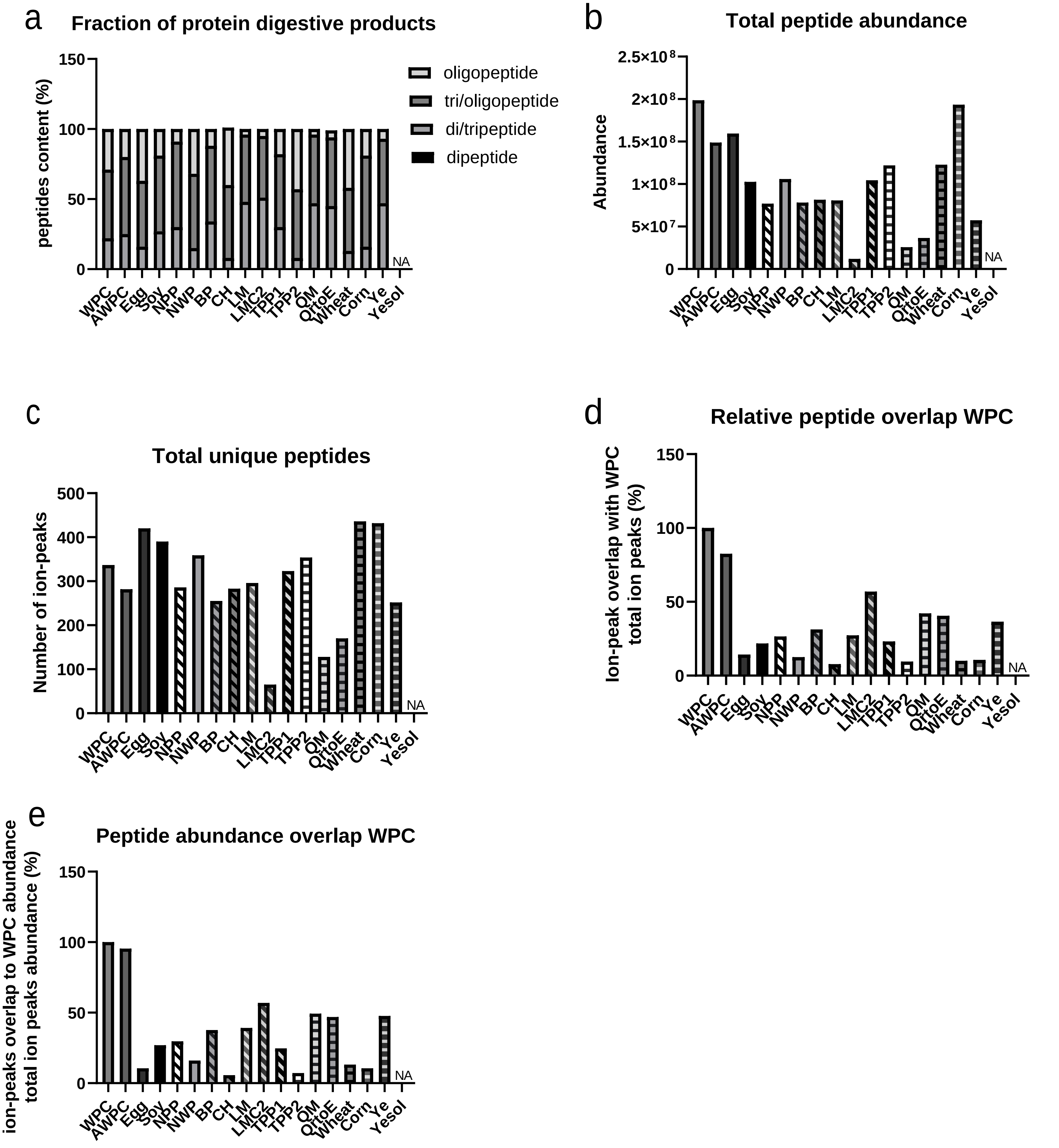

Supplementary figure 1. Proteomic analysis of potential dietary protein in comparison to whey protein concentrate (WPC). Protein samples were first corrected for digestive enzyme ion-peaks. Thereafter, A) digestive peptide profile was made by subdividing based on molar mass; dipeptides range from 150.2 – 408.4 g/mol, tripeptides is 225.3 – 612.6 g/mol everything above 300.5 g/mol is considered an oligopeptide. The values were expressed relative to the total ion-peak abundance B) shows the total abundance of all ion-peaks per protein, C) shows the total amount of unique ion-peaks per protein, D) provides the percentage of WPC overlapping ion peaks (based on charge, mass/charge ratio (±0.003) and retention time (±0.5min)) in contrast to the protein its total amount of ion-peaks and E) shows the abundance of WPC overlapping ion peaks in contrast to the total abundance per protein.

Supplementary figure 2: overview of potential dietary protein tile plots, visualizing peptide overlap and non-overlap with WPC and WPC-matched peptides and their quantity. Unique peptides of WPC were labelled with individual ID numbers (#1-337), if matched with another protein source, matching peptide received WPC ID#. Non overlapping peptides received a new ID# (>338). Abundance of overlapping peptides were plotted based on ID# on the right.

AWPC

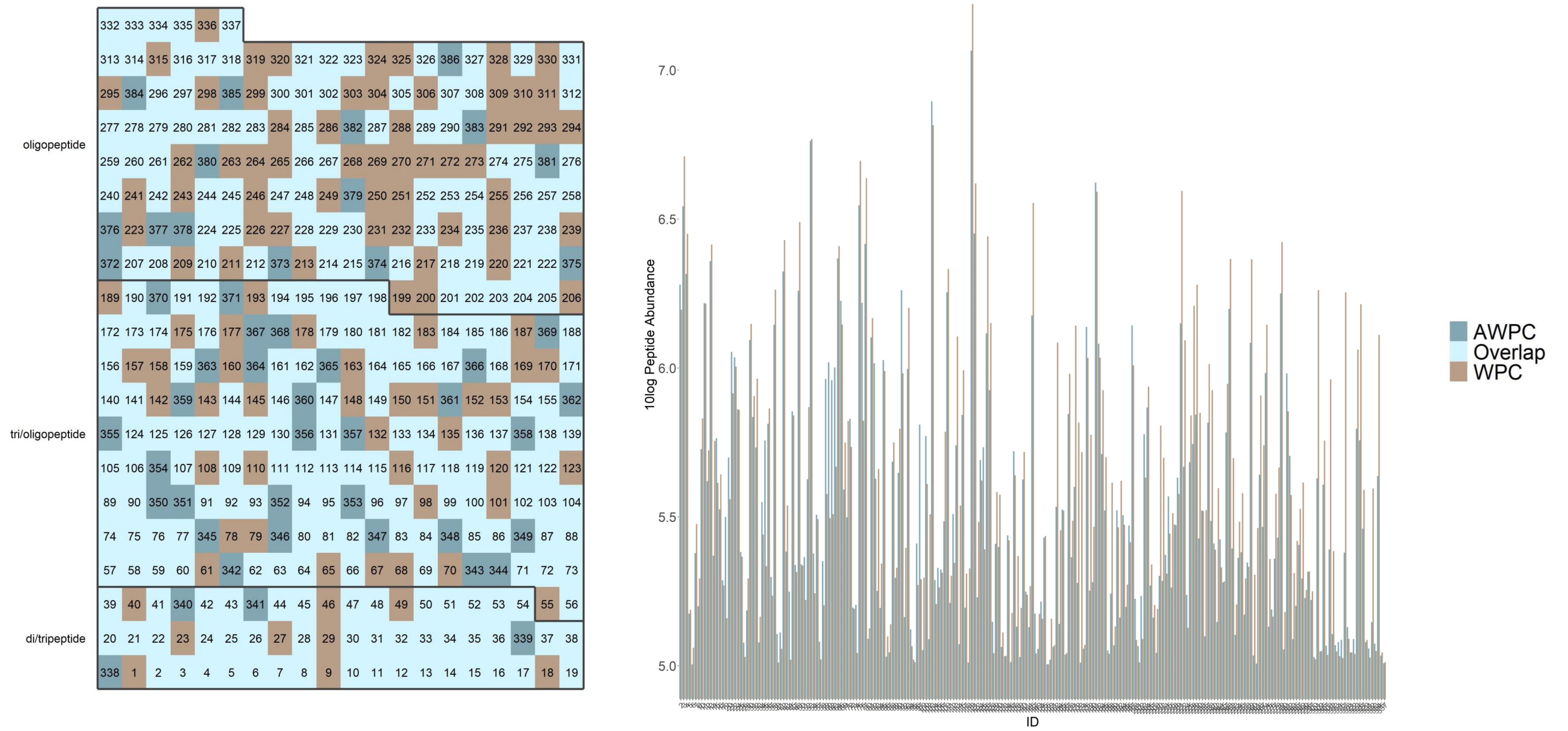

Tile plot of peptide overlap and direct comparison in peptide concentration of AWPC in comparison to WPC. Left shows the tile plot visualizing the amount of overlap, and non-overlapping peptides (ion peaks) sorted by ID number. The right figure visualizes quantities of WPC-matched peptides (ion peaks) sorted by ID number. ID numbers including information can be found in Suppl. Table 1.

Egg

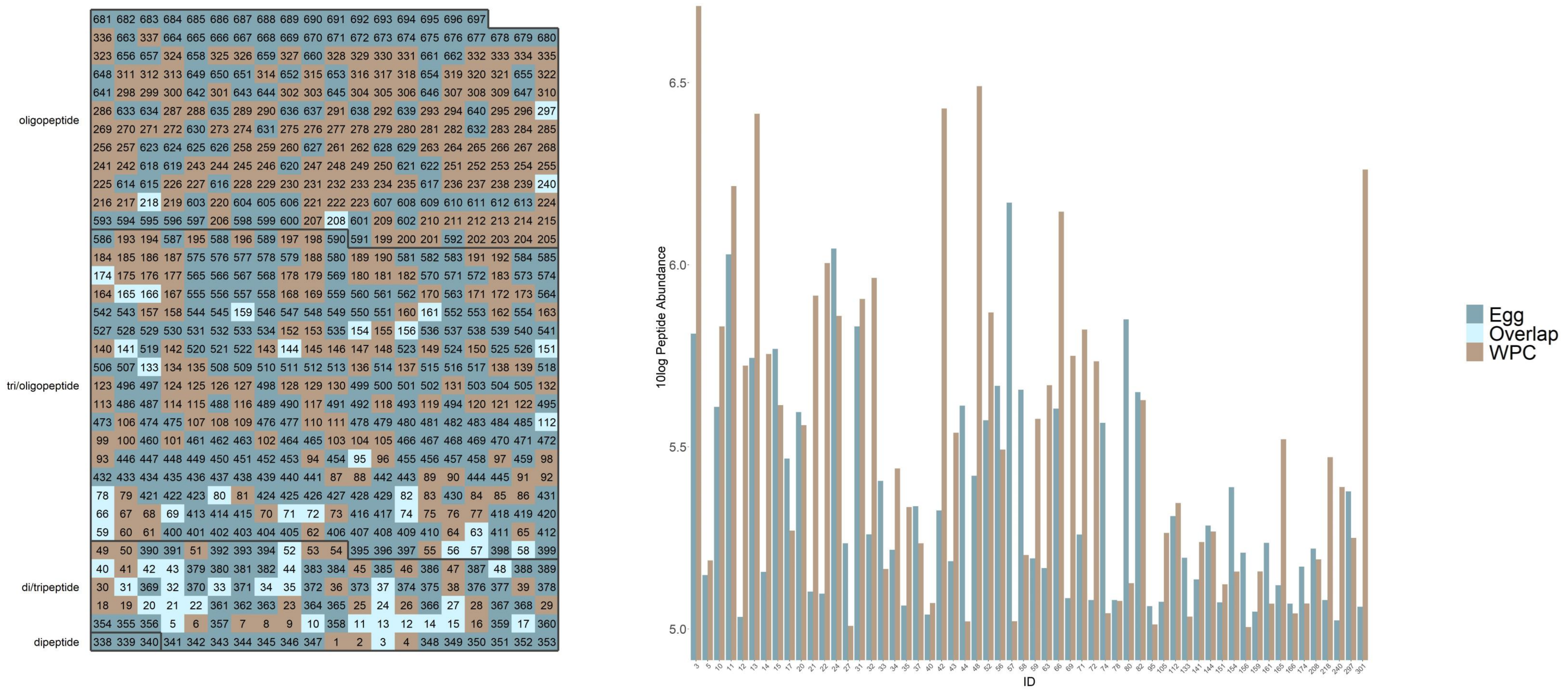

Tile plot of peptide overlap and direct comparison in peptide concentration of Egg in comparison to WPC. Left shows the tile plot visualizing the amount of overlap, and non-overlapping peptides (ion peaks) sorted by ID number. The right figure visualizes quantities of WPC-matched peptides (ion peaks) sorted by ID number. ID numbers including information can be found in Suppl. Table 1.

Soy

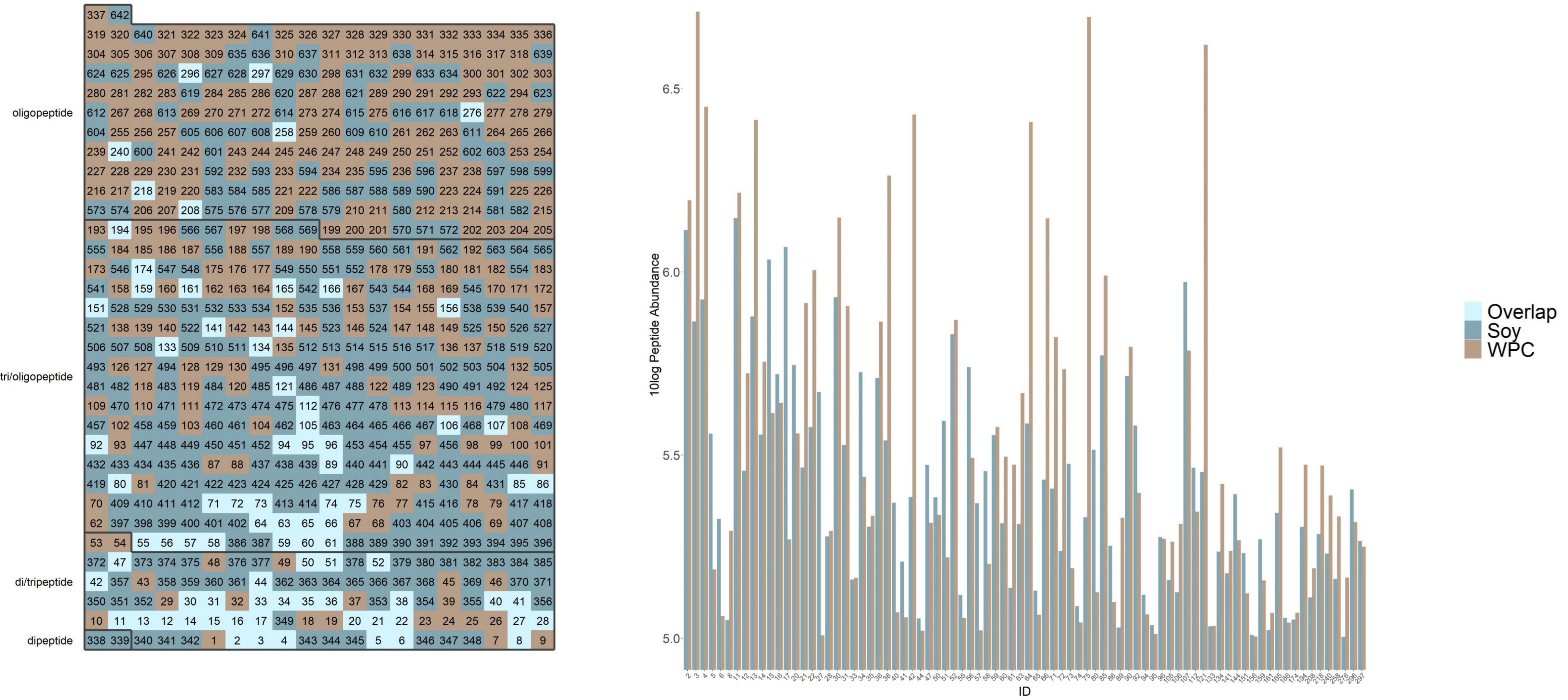

Tile plot of peptide overlap and direct comparison in peptide concentration of Soy in comparison to WPC. Left shows the tile plot visualizing the amount of overlap, and non-overlapping peptides (ion peaks) sorted by ID number. The right figure visualizes quantities of WPC-matched peptides (ion peaks) sorted by ID number. ID numbers including information can be found in Suppl. Table 1.

NPP

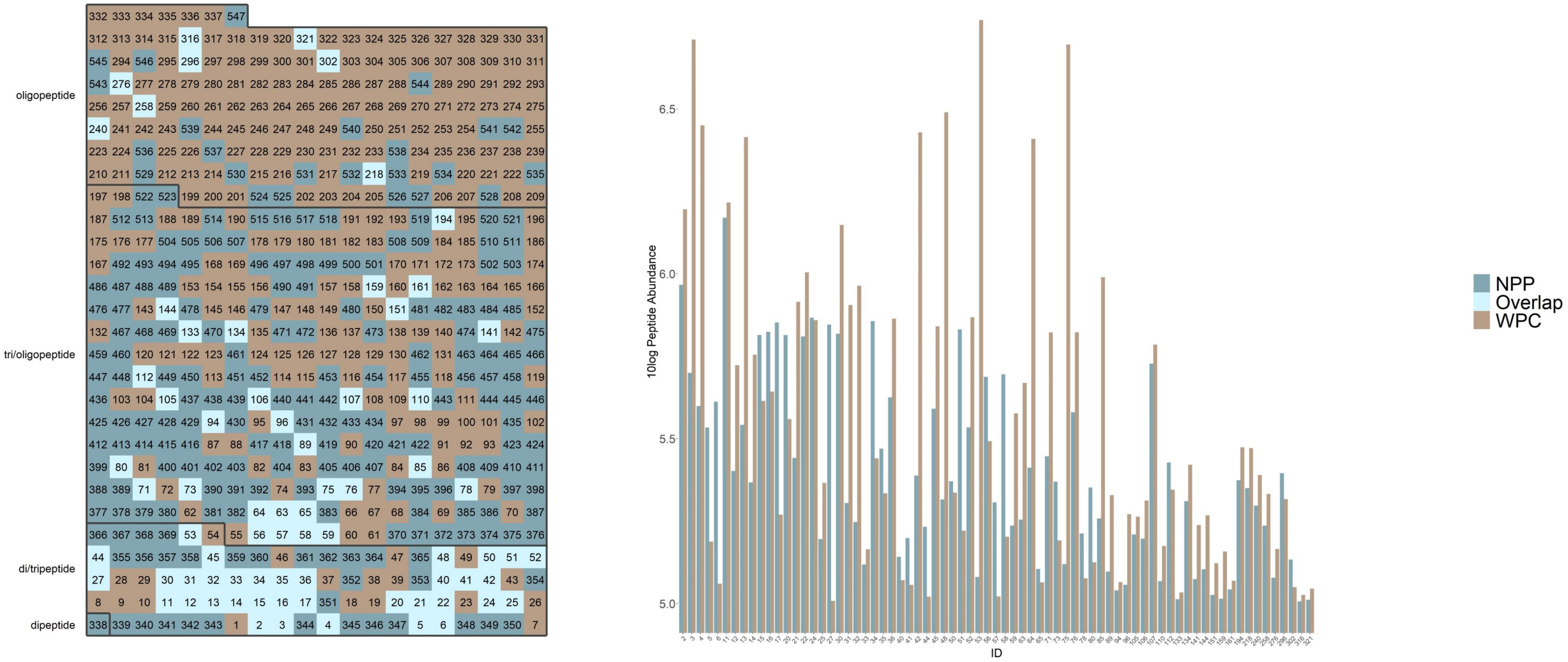

Tile plot of peptide overlap and direct comparison in peptide concentration of NPP in comparison to WPC. Left shows the tile plot visualizing the amount of overlap, and non-overlapping peptides (ion peaks) sorted by ID number. The right figure visualizes quantities of WPC-matched peptides (ion peaks) sorted by ID number. ID numbers including information can be found in Suppl. Table 1.

NWP

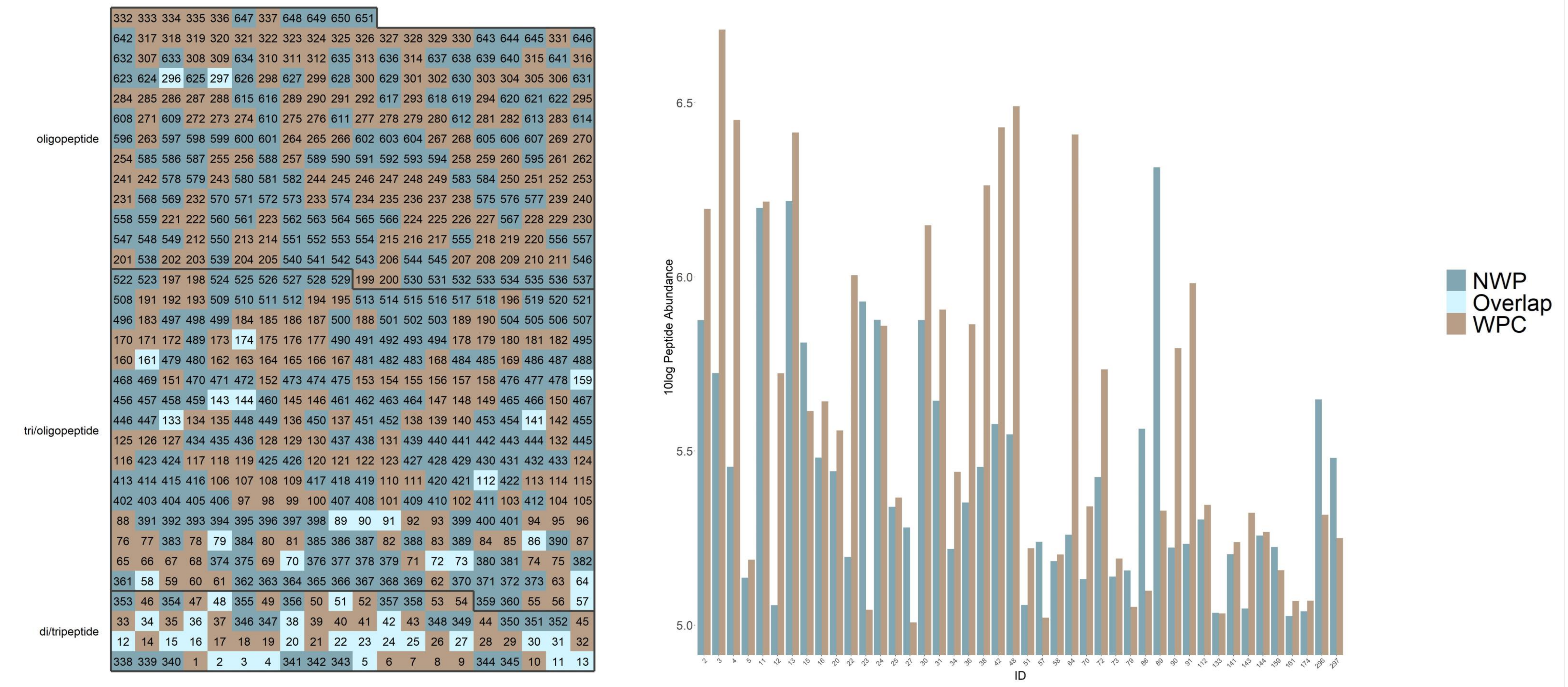

Tile plot of peptide overlap and direct comparison in peptide concentration of NWP in comparison to WPC. Left shows the tile plot visualizing the amount of overlap, and non-overlapping peptides (ion peaks) sorted by ID number. The right figure visualizes quantities of WPC-matched peptides (ion peaks) sorted by ID number. ID numbers including information can be found in Suppl. Table 1.

BP

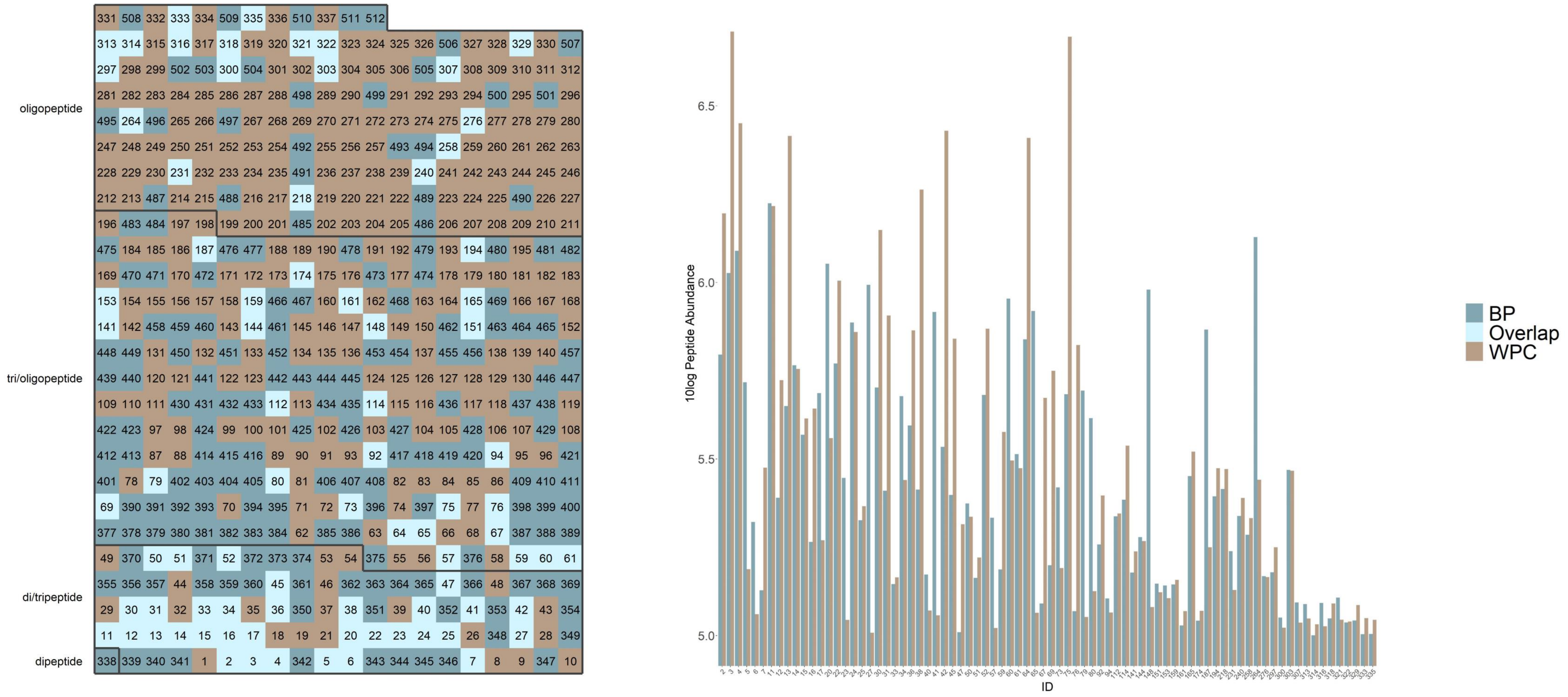

Tile plot of peptide overlap and direct comparison in peptide concentration of BP in comparison to WPC. Left shows the tile plot visualizing the amount of overlap, and non-overlapping peptides (ion peaks) sorted by ID number. The right figure visualizes quantities of WPC-matched peptides (ion peaks) sorted by ID number. ID numbers including information can be found in Suppl. Table 1.

CH

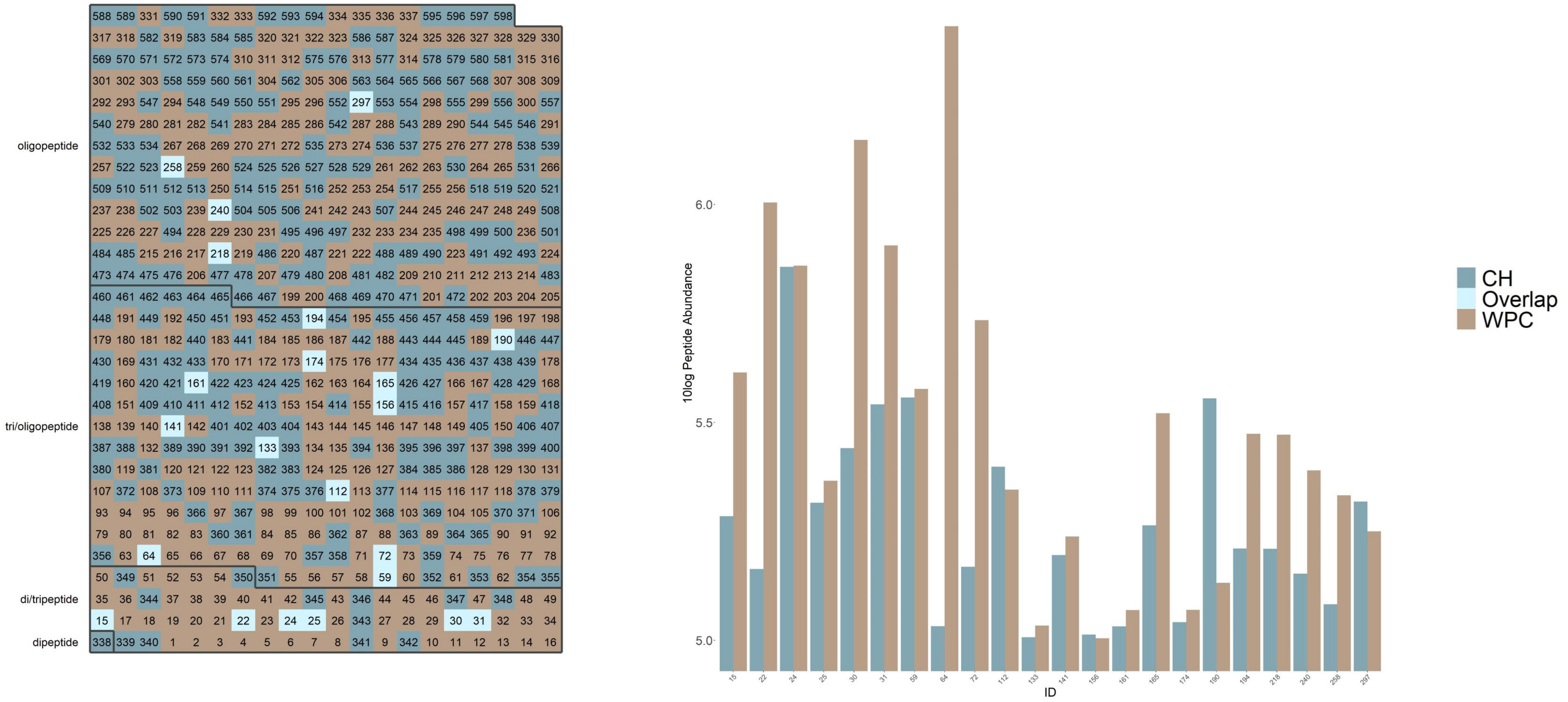

Tile plot of peptide overlap and direct comparison in peptide concentration of CH in comparison to WPC. Left shows the tile plot visualizing the amount of overlap, and non-overlapping peptides (ion peaks) sorted by ID number. The right figure visualizes quantities of WPC-matched peptides (ion peaks) sorted by ID number. ID numbers including information can be found in Suppl. Table 1.

LM

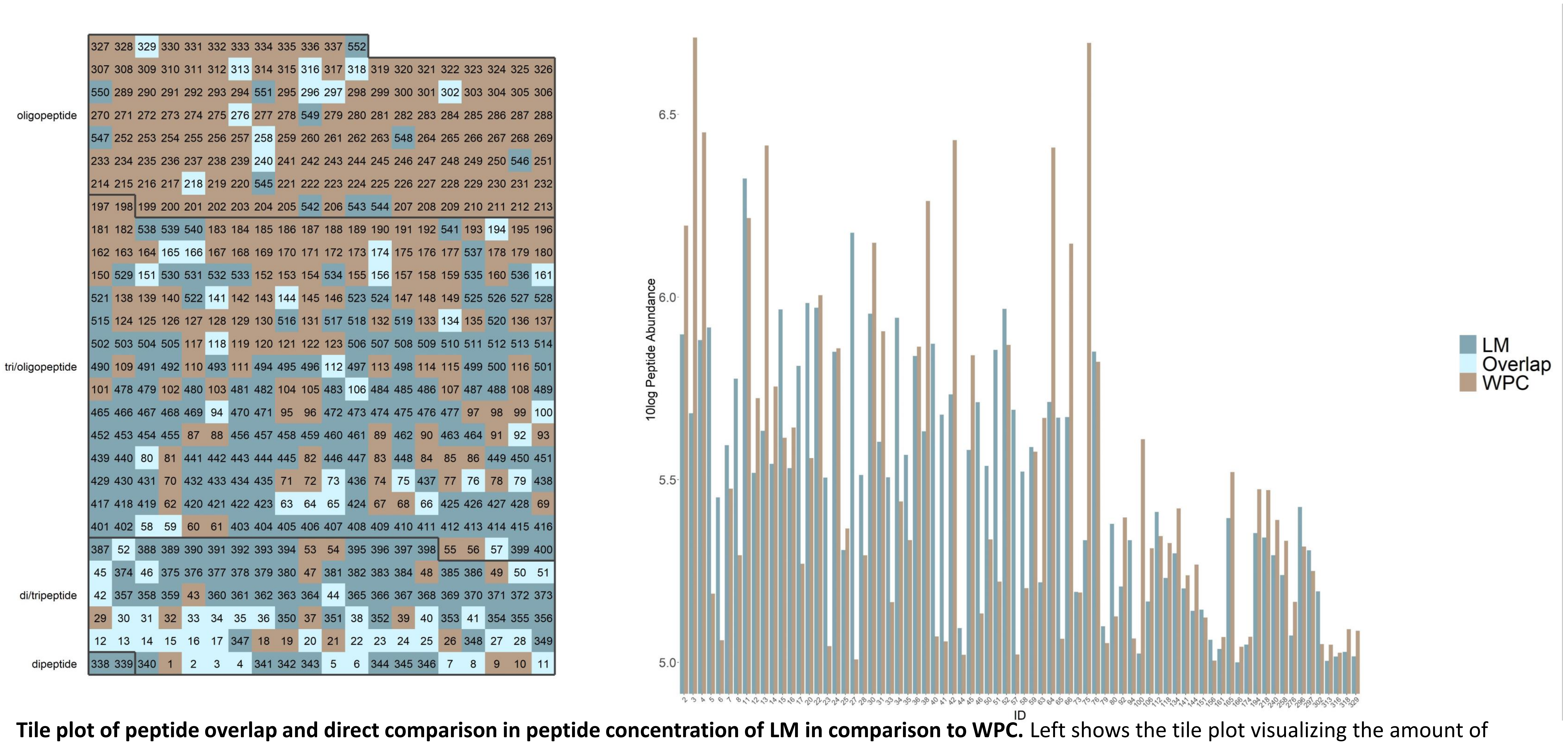

Tile plot of peptide overlap and direct comparison in peptide concentration of LM in comparison to WPC. Left shows the tile plot visualizing the amount of overlap, and non-overlapping peptides (ion peaks) sorted by ID number. The right figure visualizes quantities of WPC-matched peptides (ion peaks) sorted by ID number. ID numbers including information can be found in Suppl. Table 1.

LMC2

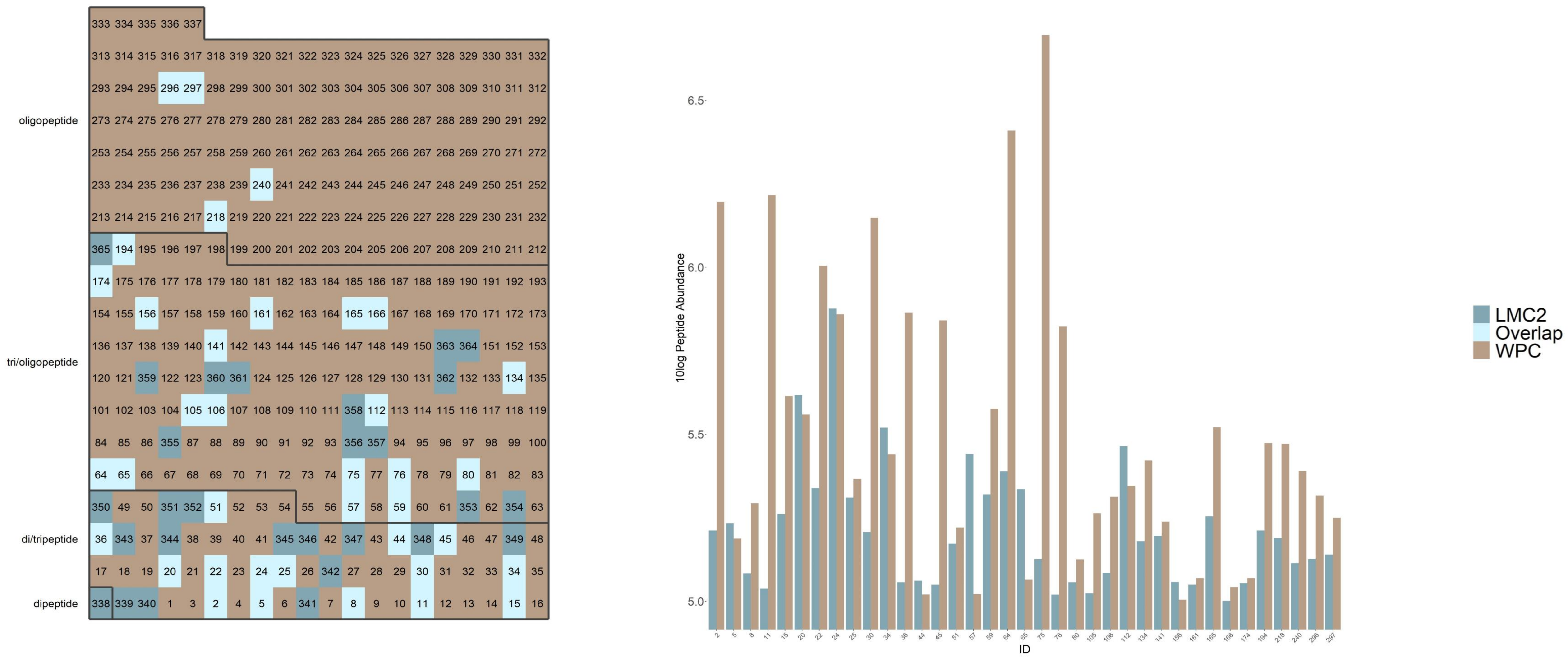

Tile plot of peptide overlap and direct comparison in peptide concentration of LMC2 in comparison to WPC. Left shows the tile plot visualizing the amou[illegible] overlap, and non-overlapping peptides (ion peaks) sorted by ID number. The right figure visualizes quantities of WPC-matched peptides (ion peaks) sorted by ID number. ID numbers including information can be found in Suppl. Table 1.

TPP1

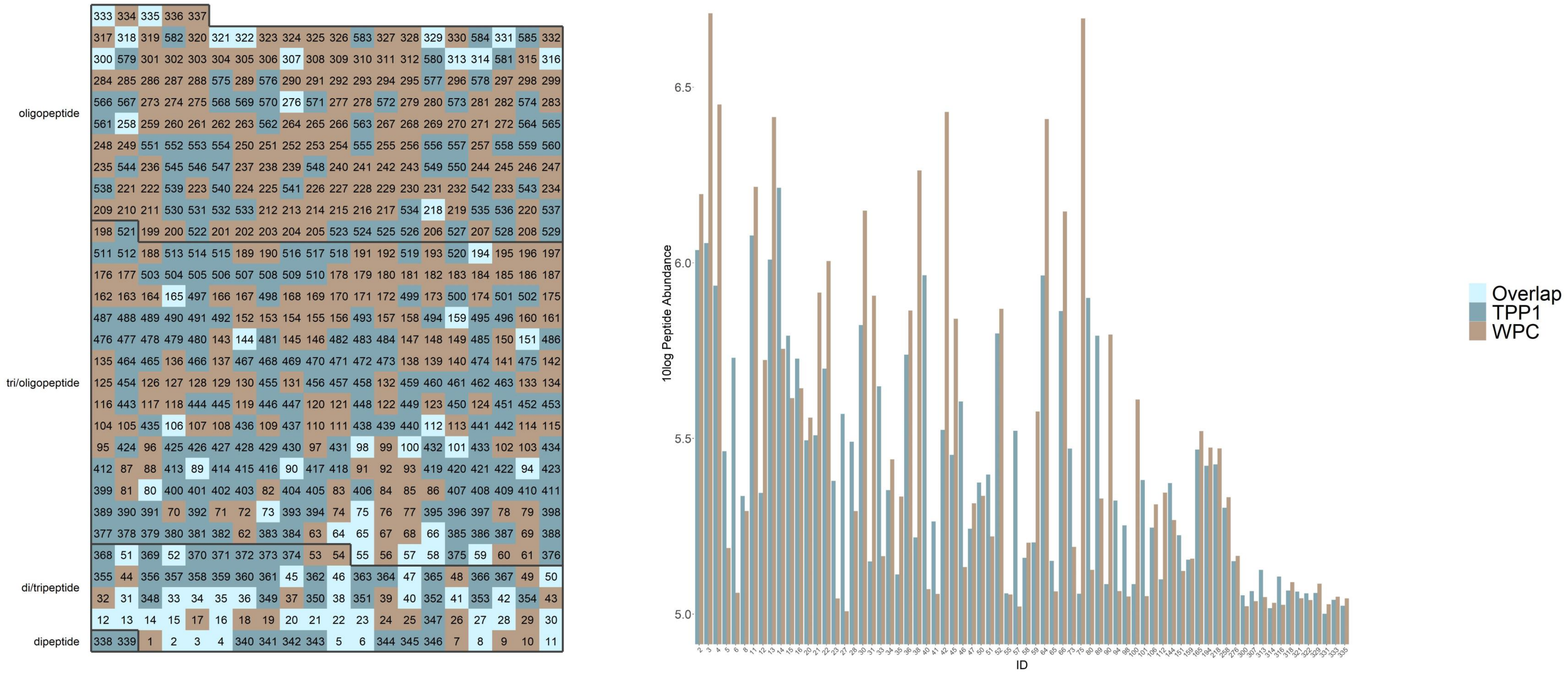

Tile plot of peptide overlap and direct comparison in peptide concentration of TPP1 in comparison to WPC. Left shows the tile plot visualizing the amount of overlap, and non-overlapping peptides (ion peaks) sorted by ID number. The right figure visualizes quantities of WPC-matched peptides (ion peaks) sorted by ID number. ID numbers including information can be found in Suppl. Table 1.

TPP2

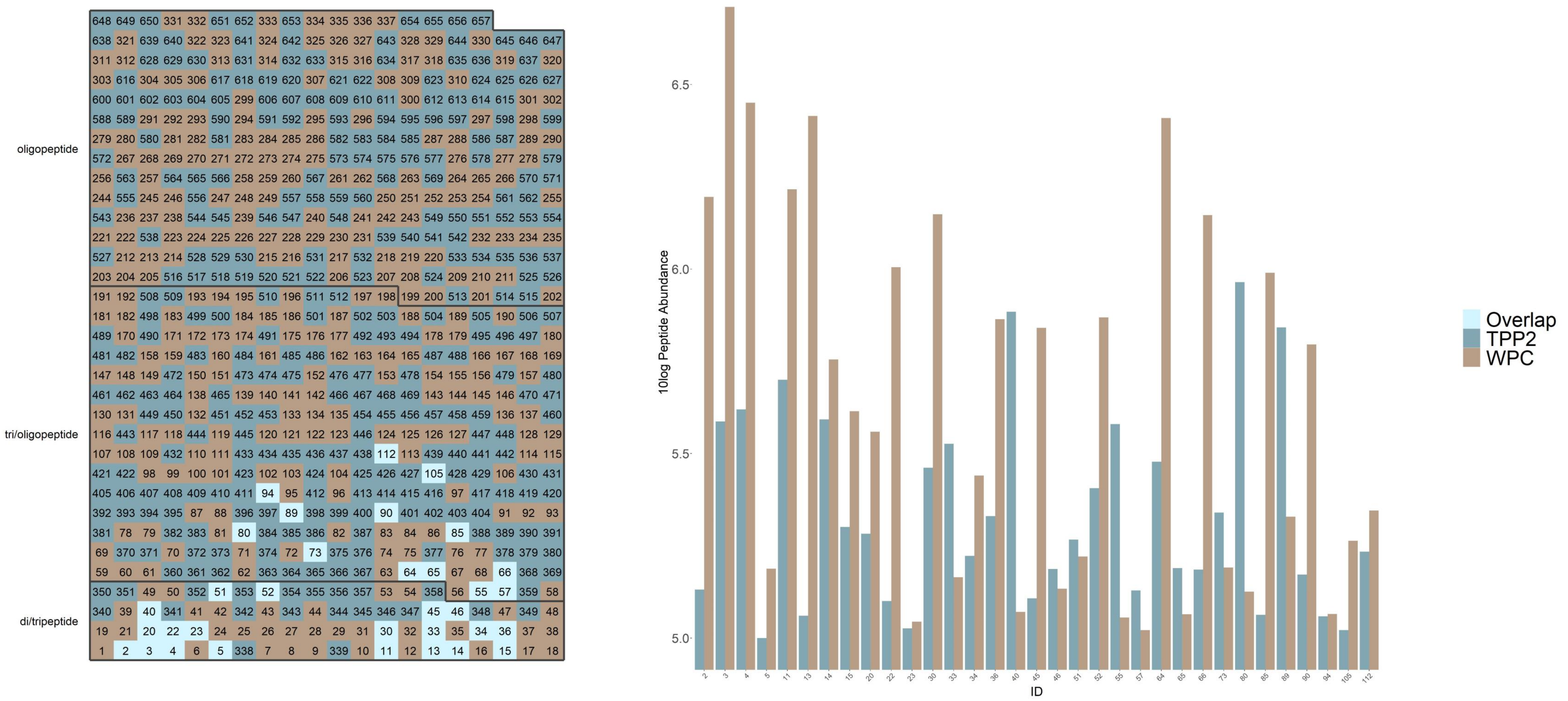

Tile plot of peptide overlap and direct comparison in peptide concentration of TPP2 in comparison to WPC. Left shows the tile plot visualizing the amount of overlap, and non-overlapping peptides (ion peaks) sorted by ID number. The right figure visualizes quantities of WPC-matched peptides (ion peaks) sorted by ID number. ID numbers including information can be found in Suppl. Table 1.

QM

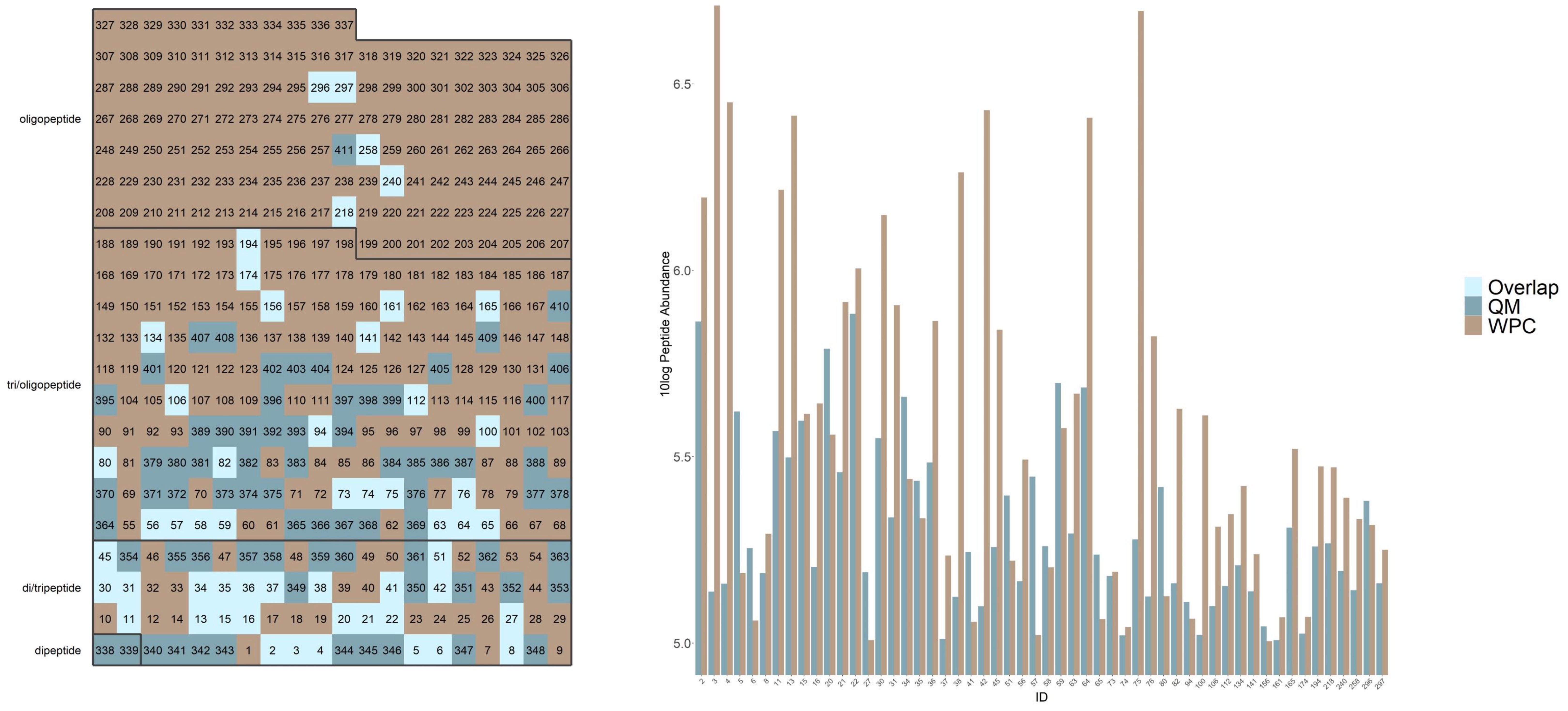

Tile plot of peptide overlap and direct comparison in peptide concentration of QM in comparison to WPC. Left shows the tile plot visualizing the amount of overlap, and non-overlapping peptides (ion peaks) sorted by ID number. The right figure visualizes quantities of WPC-matched peptides (ion peaks) sorted by ID number. ID numbers including information can be found in Suppl. Table 1.

QrtoE

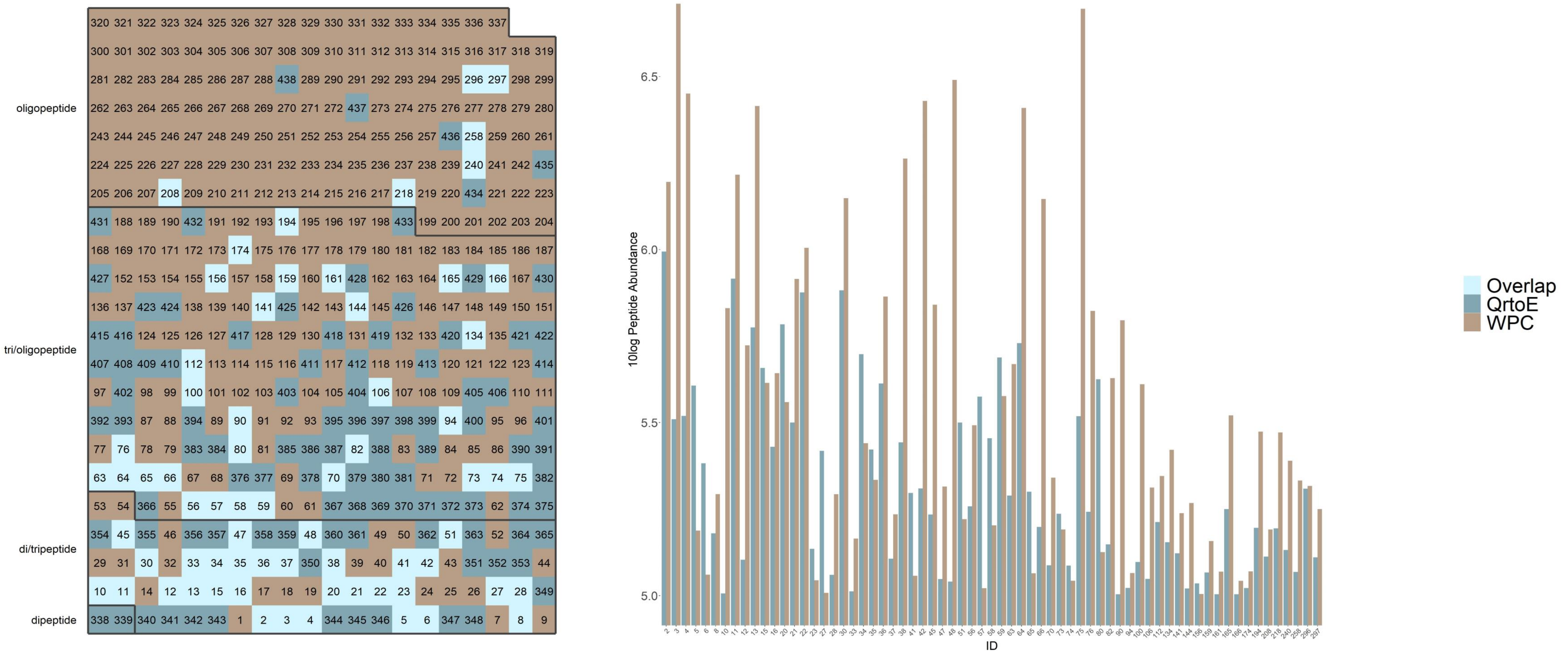

Tile plot of peptide overlap and direct comparison in peptide concentration of QrtoE in comparison to WPC. Left shows the tile plot visualizing the amount of overlap, and non-overlapping peptides (ion peaks) sorted by ID number. The right figure visualizes quantities of WPC-matched peptides (ion peaks) sorted by ID number. ID numbers including information can be found in Suppl. Table 1.

Wheat

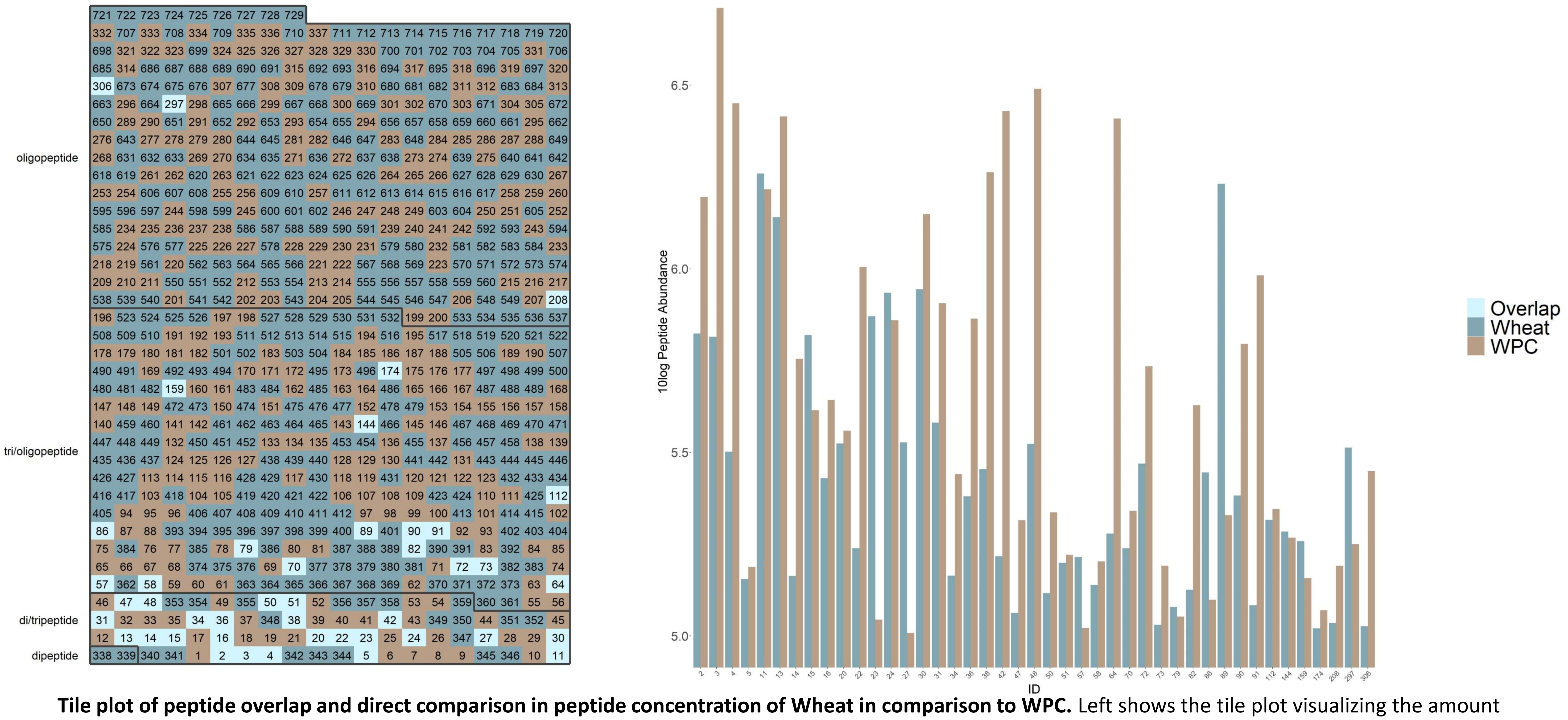

Tile plot of peptide overlap and direct comparison in peptide concentration of Wheat in comparison to WPC. Left shows the tile plot visualizing the amount of overlap, and non-overlapping peptides (ion peaks) sorted by ID number. The right figure visualizes quantities of WPC-matched peptides (ion peaks) sorted by ID number. ID numbers including information can be found in Suppl. Table 1.

Corn

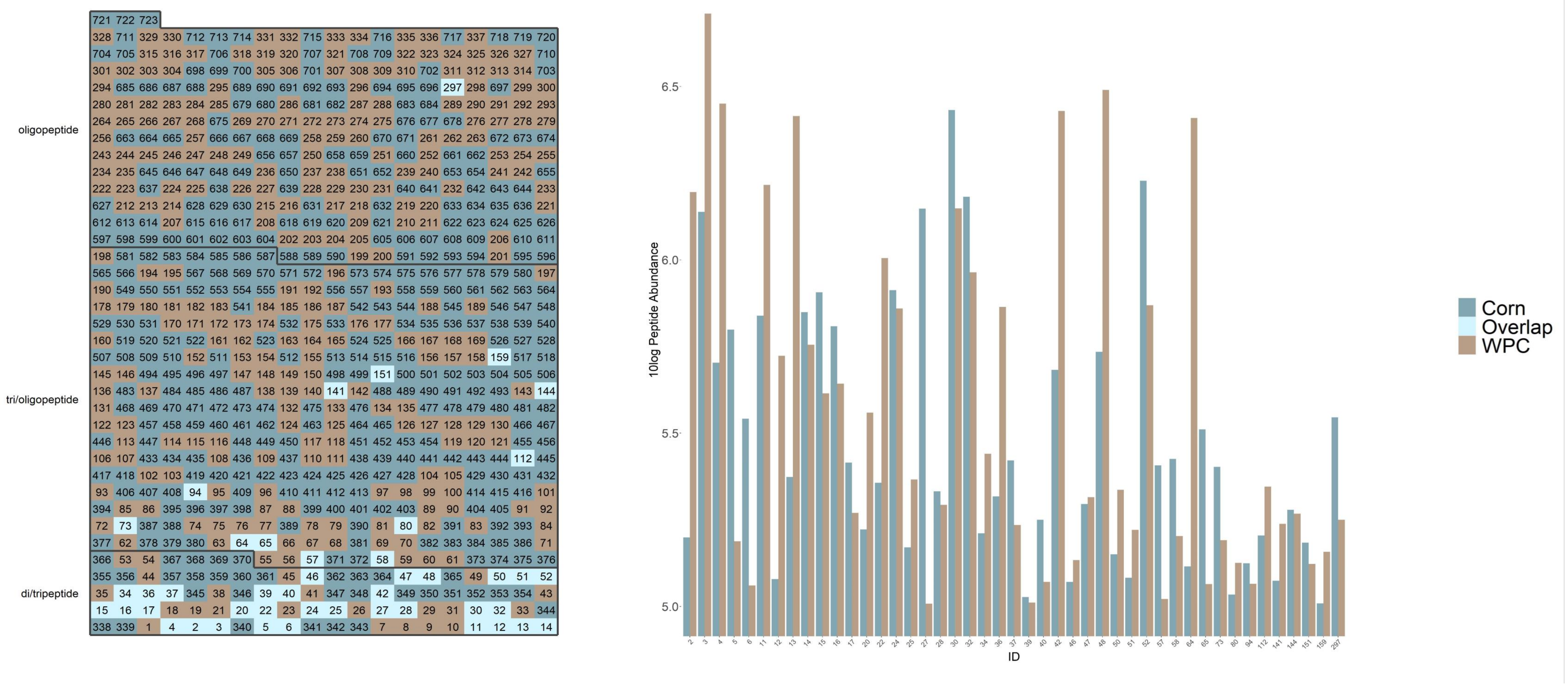

Tile plot of peptide overlap and direct comparison in peptide concentration of Corn in comparison to WPC. Left shows the tile plot visualizing the amount of overlap, and non-overlapping peptides (ion peaks) sorted by ID number. The right figure visualizes quantities of WPC-matched peptides (ion peaks) sorted by ID number. ID numbers including information can be found in Suppl. Table 1.

YE

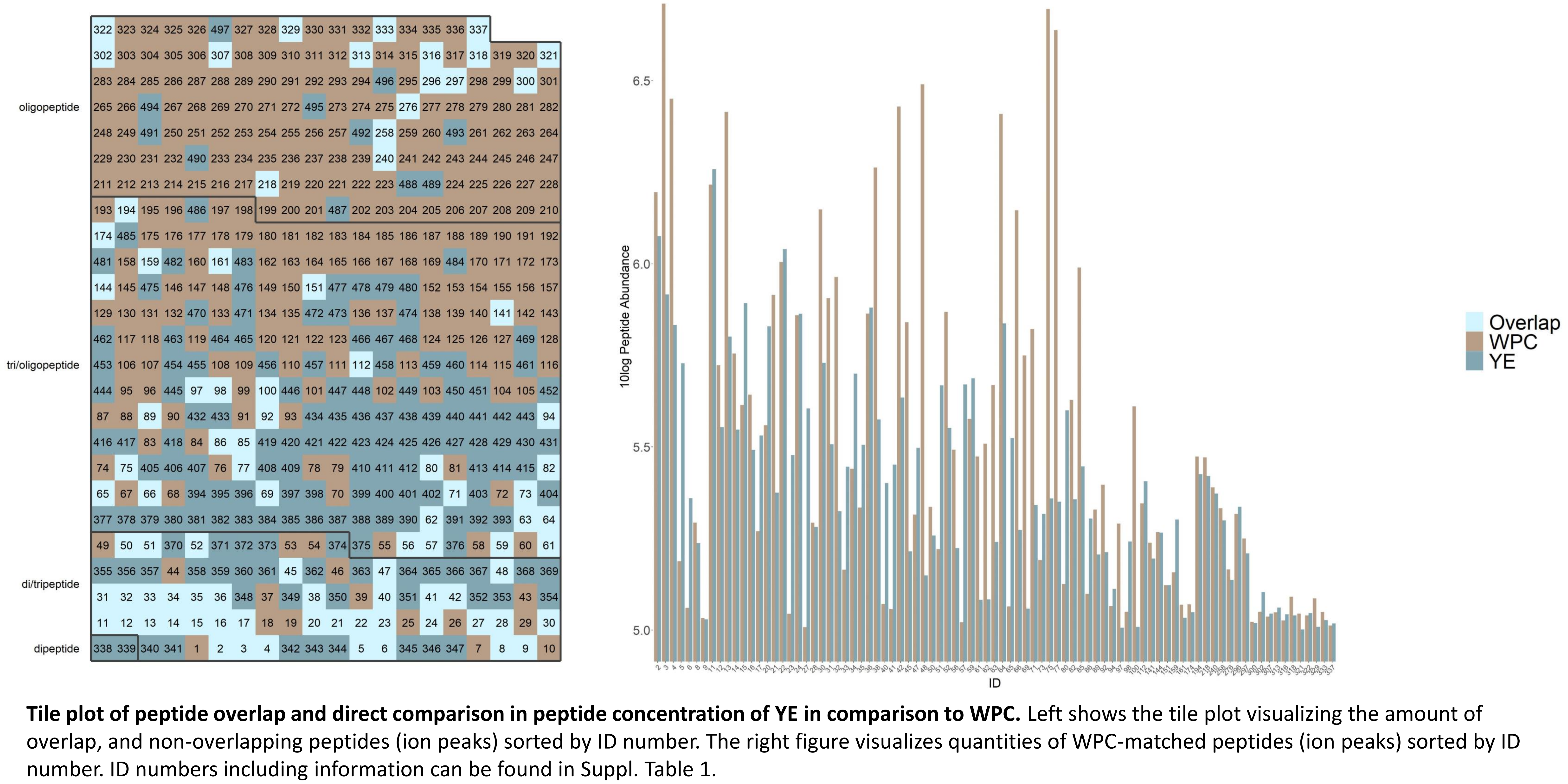

Tile plot of peptide overlap and direct comparison in peptide concentration of YE in comparison to WPC. Left shows the tile plot visualizing the amount of overlap, and non-overlapping peptides (ion peaks) sorted by ID number. The right figure visualizes quantities of WPC-matched peptides (ion peaks) sorted by ID number. ID numbers including information can be found in Suppl. Table 1.

ID	Protein	mz	charge	retentiont	raw.abund	mass	type
1	WPC	230.248	1	36.62458	218546.5	229.2407	di/tripeptide
2	WPC	231.1708	1	5.204583	1568927	230.1635	di/tripeptide
3	WPC	231.1708	1	9.601775	5129673	230.1635	di/tripeptide
4	WPC	231.1708	1	12.02929	2821537	230.1636	di/tripeptide
5	WPC	233.1497	1	2.2007	154251.9	232.1424	di/tripeptide
6	WPC	233.1498	1	2.780942	114979.6	232.1426	di/tripeptide
7	WPC	239.1027	1	2.18985	298924.1	238.0954	di/tripeptide
8	WPC	239.1029	1	2.425233	196607.6	238.0956	di/tripeptide
9	WPC	243.1345	1	5.418808	107826.2	242.1272	di/tripeptide
10	WPC	245.1133	1	1.978642	677213.8	244.106	di/tripeptide
11	WPC	245.1863	1	16.42797	1644069	244.179	di/tripeptide
12	WPC	245.1865	1	15.90611	528585.5	244.1793	di/tripeptide
13	WPC	245.1866	1	14.55145	2597514	244.1793	di/tripeptide
14	WPC	245.1866	1	17.6888	568858.5	244.1793	di/tripeptide
15	WPC	246.145	1	2.123375	411579.4	245.1377	di/tripeptide
16	WPC	246.1451	1	2.6523	439301.4	245.1378	di/tripeptide
17	WPC	246.1453	1	3.364567	186173.6	245.138	di/tripeptide
18	WPC	247.1108	1	20.3645	123176.6	246.1036	di/tripeptide
19	WPC	247.1113	1	2.381492	144495.1	246.1041	di/tripeptide
20	WPC	247.129	1	2.18985	362628.6	246.1217	di/tripeptide
21	WPC	247.1291	1	3.147667	822062.9	246.1218	di/tripeptide
22	WPC	247.1293	1	4.658808	1010908	246.122	di/tripeptide
23	WPC	249.1271	1	2.795658	110783.5	248.1198	di/tripeptide
24	WPC	250.178	1	34.86286	723803.2	249.1707	di/tripeptide
25	WPC	250.1781	1	37.31456	232458	249.1708	di/tripeptide
26	WPC	252.9628	1	4.78395	107139.5	251.9555	di/tripeptide
27	WPC	253.1185	1	2.301917	101901.8	252.1112	di/tripeptide
28	WPC	253.1188	1	9.2948	196395.1	252.1115	di/tripeptide
29	WPC	258.2794	1	37.62385	527017	257.2722	di/tripeptide
30	WPC	260.1607	1	2.18985	1406430	259.1534	di/tripeptide
31	WPC	260.1608	1	2.6523	805315.3	259.1535	di/tripeptide
32	WPC	260.161	1	3.703708	920707.1	259.1537	di/tripeptide
33	WPC	260.1971	1	2.18985	146183.2	259.1898	di/tripeptide
34	WPC	261.1447	1	2.18985	275836.5	260.1375	di/tripeptide
35	WPC	261.1449	1	3.2809	216032.6	260.1376	di/tripeptide
36	WPC	261.145	1	5.410683	731320.4	260.1378	di/tripeptide
37	WPC	262.1399	1	2.093217	171943.3	261.1327	di/tripeptide
38	WPC	263.143	1	11.50558	1830714	262.1357	di/tripeptide
39	WPC	263.1967	1	2.18985	102589.8	262.1895	di/tripeptide
40	WPC	265.1553	1	16.2901	117787.5	264.148	di/tripeptide
41	WPC	267.1343	1	2.404683	114092.9	266.127	di/tripeptide
42	WPC	269.1608	1	2.157517	2686633	268.1536	di/tripeptide
43	WPC	274.1875	1	1.978642	345626.5	273.1802	di/tripeptide
44	WPC	277.1032	1	1.86235	104868	276.096	di/tripeptide
45	WPC	281.1134	1	2.566517	693056.2	280.1062	di/tripeptide
46	WPC	281.1503	1	6.977133	136167.2	280.143	di/tripeptide
47	WPC	288.1925	1	16.08898	206592.3	287.1852	di/tripeptide
48	WPC	290.1714	1	2.712558	3091093	289.1641	di/tripeptide
49	WPC	292.1327	1	2.18985	117891.6	291.1254	di/tripeptide

50	WPC	294.1451	1	2.324467	217038.3	293.1379	di/tripeptide
51	WPC	295.1295	1	3.245917	166335.6	294.1222	di/tripeptide
52	WPC	295.1657	1	16.42797	739234.7	294.1585	di/tripeptide
53	WPC	300.192	1	2.5938	5880434	299.1847	di/tripeptide
54	WPC	300.1928	1	15.14582	175279.4	299.1855	di/tripeptide
55	WPC	302.208	1	16.42797	113690.3	301.2007	tri/oligopeptide
56	WPC	302.2082	1	15.16074	310777.4	301.2009	tri/oligopeptide
57	WPC	302.2084	1	19.87094	105064.9	301.2011	tri/oligopeptide
58	WPC	303.1666	1	2.2007	159566.5	302.1594	tri/oligopeptide
59	WPC	304.1506	1	2.404683	377347	303.1433	tri/oligopeptide
60	WPC	304.1509	1	4.823717	313274.7	303.1437	tri/oligopeptide
61	WPC	304.1664	1	19.00618	297847.9	303.1591	tri/oligopeptide
62	WPC	316.2237	1	13.87745	322898.2	315.2164	tri/oligopeptide
63	WPC	318.1664	1	2.531717	466715	317.1591	tri/oligopeptide
64	WPC	318.1665	1	2.324467	2564465	317.1592	tri/oligopeptide
65	WPC	318.1669	1	9.051517	116011.3	317.1596	tri/oligopeptide
66	WPC	318.1821	1	22.57613	1399162	317.1748	tri/oligopeptide
67	WPC	318.1823	1	24.72373	471190.9	317.175	tri/oligopeptide
68	WPC	318.1824	1	23.73726	113843.7	317.1751	tri/oligopeptide
69	WPC	319.1615	1	2.168392	561926.7	318.1542	tri/oligopeptide
70	WPC	325.1133	1	1.719867	219276.2	324.106	tri/oligopeptide
71	WPC	330.2396	1	18.42354	663790.9	329.2324	tri/oligopeptide
72	WPC	331.1613	1	2.081408	542874.9	330.1541	tri/oligopeptide
73	WPC	331.1657	1	2.837858	155454.8	330.1584	tri/oligopeptide
74	WPC	331.2851	1	38.45748	110434.2	330.2778	tri/oligopeptide
75	WPC	332.1818	1	2.6191	4958838	331.1745	tri/oligopeptide
76	WPC	332.2186	1	3.085625	664880.6	331.2113	tri/oligopeptide
77	WPC	332.2187	1	3.418708	4341485	331.2114	tri/oligopeptide
78	WPC	333.1407	1	1.7666	119427.6	332.1335	tri/oligopeptide
79	WPC	333.1567	1	10.97644	113003.9	332.1494	tri/oligopeptide
80	WPC	334.1612	1	2.21185	133517.7	333.1539	tri/oligopeptide
81	WPC	334.1613	1	3.245917	1468881	333.1541	tri/oligopeptide
82	WPC	342.2399	1	21.82051	424860	341.2326	tri/oligopeptide
83	WPC	343.1239	1	1.719867	457935	342.1167	tri/oligopeptide
84	WPC	344.1826	1	5.567342	220477.6	343.1753	tri/oligopeptide
85	WPC	344.2555	1	21.54997	976111.4	343.2482	tri/oligopeptide
86	WPC	344.2556	1	20.18498	125510.4	343.2483	tri/oligopeptide
87	WPC	349.1723	1	1.86235	137873.8	348.165	tri/oligopeptide
88	WPC	350.1197	1	1.8327	562475.5	349.1125	tri/oligopeptide
89	WPC	358.2712	1	24.02735	213384.7	357.2639	tri/oligopeptide
90	WPC	359.2293	1	2.18985	624779.6	358.2221	tri/oligopeptide
91	WPC	360.196	1	18.73659	959584.1	359.1888	tri/oligopeptide
92	WPC	360.2134	1	2.736492	249087.5	359.2061	tri/oligopeptide
93	WPC	360.2137	1	13.6536	1589841	359.2064	tri/oligopeptide
94	WPC	363.1555	1	2.18985	116238.7	362.1482	tri/oligopeptide
95	WPC	365.1366	1	37.82153	102864.7	364.1293	tri/oligopeptide
96	WPC	366.214	1	2.18985	186795.1	365.2067	tri/oligopeptide
97	WPC	374.2297	1	19.34277	195229.6	373.2224	tri/oligopeptide
98	WPC	375.2239	1	2.2007	112242.4	374.2166	tri/oligopeptide
99	WPC	376.1467	1	1.719867	197899.7	375.1395	tri/oligopeptide

100	WPC	376.1719	1	2.2007	407839.7	375.1646	tri/oligopeptide
101	WPC	376.2276	1	21.63163	112528.8	375.2203	tri/oligopeptide
102	WPC	381.0801	1	1.6744	322600.8	380.0728	tri/oligopeptide
103	WPC	387.2246	1	13.99581	6537951	386.2173	tri/oligopeptide
104	WPC	389.2306	1	13.98086	161396.7	388.2233	tri/oligopeptide
105	WPC	390.1876	1	2.313292	183419.9	389.1803	tri/oligopeptide
106	WPC	395.0394	1	1.572517	205423.7	394.0321	tri/oligopeptide
107	WPC	399.2612	1	18.55638	610178.2	398.254	tri/oligopeptide
108	WPC	401.1786	1	1.510183	107765.2	400.1713	tri/oligopeptide
109	WPC	402.2354	1	11.39669	2149924	401.2281	tri/oligopeptide
110	WPC	405.162	1	1.844392	149490.1	404.1547	tri/oligopeptide
111	WPC	405.199	1	4.178875	200487.1	404.1918	tri/oligopeptide
112	WPC	415.2118	1	36.4732	221664.2	414.2046	tri/oligopeptide
113	WPC	417.2352	1	22.10395	1276204	416.2279	tri/oligopeptide
114	WPC	420.2094	1	2.464725	345128.3	419.2021	tri/oligopeptide
115	WPC	421.1932	1	2.2007	982449.5	420.1859	tri/oligopeptide
116	WPC	423.0647	1	1.697417	151401.7	422.0574	tri/oligopeptide
117	WPC	428.287	1	2.2007	204194.7	427.2797	tri/oligopeptide
118	WPC	429.272	1	18.61096	212246.2	428.2647	tri/oligopeptide
119	WPC	431.2332	1	20.35361	16663423	430.2259	tri/oligopeptide
120	WPC	433.0939	1	1.6744	153852.8	432.0866	tri/oligopeptide
121	WPC	433.1931	1	2.895875	4165481	432.1858	tri/oligopeptide
122	WPC	434.1884	1	2.047242	304482.7	433.1812	tri/oligopeptide
123	WPC	435.199	1	2.889092	122352.1	434.1918	tri/oligopeptide
124	WPC	444.2464	1	10.52273	418633.2	443.2392	tri/oligopeptide
125	WPC	445.2305	1	14.19772	246155.8	444.2232	tri/oligopeptide
126	WPC	445.2662	1	14.23255	2765783	444.2589	tri/oligopeptide
127	WPC	445.3027	1	18.99421	1416299	444.2955	tri/oligopeptide
128	WPC	223.6254	2	2.146283	110258.4	445.2363	tri/oligopeptide
129	WPC	446.2611	1	2.2007	383107.2	445.2538	tri/oligopeptide
130	WPC	223.6342	2	2.21185	375341	445.2539	tri/oligopeptide
131	WPC	449.2078	1	17.60869	129291.1	448.2005	tri/oligopeptide
132	WPC	229.0794	2	2.895875	130998	456.1443	tri/oligopeptide
133	WPC	461.3004	1	37.32523	108090.1	460.2932	tri/oligopeptide
134	WPC	463.0268	1	1.606508	263864.8	462.0195	tri/oligopeptide
135	WPC	463.2038	1	2.2007	112764.9	462.1965	tri/oligopeptide
136	WPC	236.0739	2	2.889092	150618	470.1332	tri/oligopeptide
137	WPC	472.2412	1	11.73022	435935.1	471.234	tri/oligopeptide
138	WPC	476.2906	1	20.35361	233771.2	475.2833	tri/oligopeptide
139	WPC	477.183	1	2.18985	156430.2	476.1758	tri/oligopeptide
140	WPC	478.2307	1	14.86646	522714.3	477.2235	tri/oligopeptide
141	WPC	479.3103	1	37.14109	173144	478.303	tri/oligopeptide
142	WPC	482.2254	1	9.694933	106457.7	481.2182	tri/oligopeptide
143	WPC	488.2466	1	2.18985	210189.7	487.2394	tri/oligopeptide
144	WPC	488.2517	1	19.14056	185115.9	487.2444	tri/oligopeptide
145	WPC	244.6581	2	14.26878	188683.5	487.3017	tri/oligopeptide
146	WPC	489.2559	1	17.18248	3579764	488.2487	tri/oligopeptide
147	WPC	491.2873	1	22.65285	110004.6	490.28	tri/oligopeptide
148	WPC	491.288	1	23.14529	120511.5	490.2807	tri/oligopeptide
149	WPC	492.2304	1	2.2007	149446.9	491.2231	tri/oligopeptide

150	WPC	249.5928	2	2.880642	104121	497.171	tri/oligopeptide
151	WPC	500.2884	1	25.20752	132652.1	499.2811	tri/oligopeptide
152	WPC	255.6069	2	20.35361	125040	509.1992	tri/oligopeptide
153	WPC	513.2676	1	13.79717	127727.1	512.2603	tri/oligopeptide
154	WPC	513.3046	1	19.66069	143752.3	512.2974	tri/oligopeptide
155	WPC	515.2464	1	2.576092	272501.3	514.2391	tri/oligopeptide
156	WPC	515.2914	1	37.12918	101147.7	514.2841	tri/oligopeptide
157	WPC	518.2467	1	4.178875	119791.5	517.2394	tri/oligopeptide
158	WPC	519.2309	1	8.928583	134416.1	518.2236	tri/oligopeptide
159	WPC	520.2781	1	21.18686	143863.4	519.2709	tri/oligopeptide
160	WPC	263.5939	2	15.67608	158150.4	525.1732	tri/oligopeptide
161	WPC	264.1748	2	35.26535	117309	526.335	tri/oligopeptide
162	WPC	265.1712	2	2.511267	1216070	528.3278	tri/oligopeptide
163	WPC	530.3298	1	2.2007	166324.8	529.3225	tri/oligopeptide
164	WPC	265.6687	2	2.2007	285344.6	529.3229	tri/oligopeptide
165	WPC	531.0142	1	1.595175	331868.6	530.0069	tri/oligopeptide
166	WPC	531.3871	1	37.84241	110336.2	530.3798	tri/oligopeptide
167	WPC	532.2251	1	2.2007	956207.7	531.2178	tri/oligopeptide
168	WPC	539.2359	1	8.921633	307519.3	538.2286	tri/oligopeptide
169	WPC	270.6017	2	17.47979	177534	539.1889	tri/oligopeptide
170	WPC	544.318	1	26.99236	103620.7	543.3107	tri/oligopeptide
171	WPC	545.2939	1	18.70931	1387714	544.2866	tri/oligopeptide
172	WPC	273.1507	2	18.69747	655049.1	544.2868	tri/oligopeptide
173	WPC	546.2783	1	22.25869	521518.6	545.271	tri/oligopeptide
174	WPC	274.1677	2	35.94308	117483.5	546.3209	tri/oligopeptide
175	WPC	276.1349	2	17.19903	128044.7	550.2553	tri/oligopeptide
176	WPC	553.3361	1	23.57533	1081491	552.3288	tri/oligopeptide
177	WPC	277.6244	2	17.18858	117237.7	553.2342	tri/oligopeptide
178	WPC	561.3251	1	2.392808	273559.9	560.3178	tri/oligopeptide
179	WPC	562.2734	1	11.38478	596542.6	561.2661	tri/oligopeptide
180	WPC	565.2106	1	2.012825	292933.6	564.2033	tri/oligopeptide
181	WPC	565.2623	1	16.60482	3907241	564.255	tri/oligopeptide
182	WPC	283.1349	2	16.61216	1082928	564.2552	tri/oligopeptide
183	WPC	285.1298	2	18.70931	242415.3	568.245	tri/oligopeptide
184	WPC	286.1582	2	2.179125	842452	570.3018	tri/oligopeptide
185	WPC	571.3091	1	2.18985	502056.4	570.3018	tri/oligopeptide
186	WPC	571.3105	1	16.07581	109766.3	570.3032	tri/oligopeptide
187	WPC	572.2767	1	19.16763	177884.1	571.2694	tri/oligopeptide
188	WPC	578.232	1	9.06615	411848.7	577.2247	tri/oligopeptide
189	WPC	292.1242	2	18.70931	106683.9	582.2339	tri/oligopeptide
190	WPC	584.303	1	2.18985	135506.5	583.2957	tri/oligopeptide
191	WPC	295.114	2	16.61216	291893.9	588.2135	tri/oligopeptide
192	WPC	590.2688	1	19.6081	417765.5	589.2615	tri/oligopeptide
193	WPC	597.2523	1	2.381492	108829.2	596.245	tri/oligopeptide
194	WPC	599.002	1	1.606508	297704.2	597.9947	tri/oligopeptide
195	WPC	300.1609	2	1.424158	187409.8	598.3073	tri/oligopeptide
196	WPC	302.1085	2	16.60482	260133.9	602.2025	tri/oligopeptide
197	WPC	607.3834	1	20.80124	1020570	606.3761	tri/oligopeptide
198	WPC	304.1954	2	20.80863	122171	606.3763	tri/oligopeptide
199	WPC	308.0823	2	2.168392	104088.3	614.15	oligopeptide

200	WPC	615.1576	1	2.18985	106149.4	614.1503	oligopeptide
201	WPC	310.0947	2	16.60482	102695.6	618.1748	oligopeptide
202	WPC	314.1798	2	26.73088	123082.8	626.3451	oligopeptide
203	WPC	627.3524	1	26.71047	428634	626.3451	oligopeptide
204	WPC	628.3317	1	19.14679	865849.2	627.3245	oligopeptide
205	WPC	314.6695	2	19.15264	218800.2	627.3245	oligopeptide
206	WPC	634.2482	1	14.49353	113192.2	633.2409	oligopeptide
207	WPC	640.2975	1	19.9362	159851.2	639.2902	oligopeptide
208	WPC	643.3318	1	20.73993	155273.2	642.3246	oligopeptide
209	WPC	323.1825	2	2.404683	216816.8	644.3504	oligopeptide
210	WPC	646.3048	1	2.279217	640774.8	645.2975	oligopeptide
211	WPC	323.6563	2	2.279217	225924.3	645.298	oligopeptide
212	WPC	652.3209	1	21.66973	499650.8	651.3136	oligopeptide
213	WPC	654.3842	1	21.82051	147026	653.3769	oligopeptide
214	WPC	328.1577	2	15.64802	203884.4	654.3008	oligopeptide
215	WPC	658.3422	1	20.44584	277949	657.3349	oligopeptide
216	WPC	659.2895	1	14.00398	325590.3	658.2822	oligopeptide
217	WPC	660.2839	1	2.18985	102110.3	659.2767	oligopeptide
218	WPC	666.9895	1	1.606508	296182.6	665.9822	oligopeptide
219	WPC	668.3623	1	20.00583	378059.8	667.355	oligopeptide
220	WPC	335.6351	2	2.279217	186496.1	669.2557	oligopeptide
221	WPC	678.347	1	22.66756	3929075	677.3398	oligopeptide
222	WPC	339.6773	2	22.67454	1237910	677.34	oligopeptide
223	WPC	342.701	2	16.28062	126824.3	683.3875	oligopeptide
224	WPC	348.1777	2	2.234092	134224	694.3408	oligopeptide
225	WPC	349.198	2	12.31427	692917.7	696.3814	oligopeptide
226	WPC	350.1688	2	2.168392	102643.7	698.323	oligopeptide
227	WPC	350.6622	2	22.66756	131181.7	699.3098	oligopeptide
228	WPC	701.3478	1	16.54638	1618184	700.3406	oligopeptide
229	WPC	351.1776	2	16.53589	1903700	700.3407	oligopeptide
230	WPC	351.6563	2	22.67454	706602.9	701.298	oligopeptide
231	WPC	702.3688	1	18.63017	134673.1	701.3615	oligopeptide
232	WPC	707.2228	1	1.697417	464747.2	706.2156	oligopeptide
233	WPC	356.6847	2	2.024292	330991.7	711.3549	oligopeptide
234	WPC	239.4362	3	22.67454	205965.4	715.2867	oligopeptide
235	WPC	358.6509	2	22.67454	333158.8	715.2872	oligopeptide
236	WPC	362.1624	2	16.54638	153103.2	722.3102	oligopeptide
237	WPC	363.1464	2	1.955792	1030446	724.2783	oligopeptide
238	WPC	363.1565	2	16.53589	842482.8	724.2985	oligopeptide
239	WPC	366.6369	2	22.67454	131593.4	731.2593	oligopeptide
240	WPC	734.9771	1	1.606508	245470.7	733.9699	oligopeptide
241	WPC	247.1031	3	16.55465	134166.9	738.2874	oligopeptide
242	WPC	370.1511	2	16.53589	394579.1	738.2877	oligopeptide
243	WPC	371.6317	2	18.01268	149229.9	741.2489	oligopeptide
244	WPC	374.6898	2	20.78791	213939.3	747.3651	oligopeptide
245	WPC	376.7003	2	2.222883	192112.4	751.3861	oligopeptide
246	WPC	377.6498	2	21.6631	102812.8	753.2851	oligopeptide
247	WPC	377.7298	2	29.30715	885810.8	753.445	oligopeptide
248	WPC	754.4523	1	29.29283	2322458	753.4451	oligopeptide
249	WPC	378.1371	2	16.53589	143219.4	754.2597	oligopeptide

250	WPC	765.1816	1	1.697417	100309.2	764.1744	oligopeptide
251	WPC	384.6574	2	22.66756	128519.1	767.3002	oligopeptide
252	WPC	769.4105	1	17.81463	498139.5	768.4032	oligopeptide
253	WPC	770.3676	1	2.18985	160709.5	769.3603	oligopeptide
254	WPC	385.6876	2	2.222883	304576.2	769.3606	oligopeptide
255	WPC	778.4486	1	23.56193	121264	777.4413	oligopeptide
256	WPC	389.728	2	23.56193	379864.2	777.4414	oligopeptide
257	WPC	392.6952	2	2.21185	196456.1	783.3758	oligopeptide
258	WPC	802.9646	1	1.595175	215114.1	801.9573	oligopeptide
259	WPC	804.3277	1	13.70475	2317512	803.3204	oligopeptide
260	WPC	402.6675	2	13.71081	202118.7	803.3205	oligopeptide
261	WPC	406.7113	2	14.1609	290124.1	811.408	oligopeptide
262	WPC	406.7115	2	8.7537	113522.6	811.4085	oligopeptide
263	WPC	408.2304	2	14.73601	117665.3	814.4462	oligopeptide
264	WPC	276.1786	3	15.63843	276222.5	825.5138	oligopeptide
265	WPC	414.1595	2	14.04863	124023.7	826.3044	oligopeptide
266	WPC	414.6465	2	13.70475	808464.5	827.2784	oligopeptide
267	WPC	419.7279	2	25.05807	550561.2	837.4413	oligopeptide
268	WPC	838.4487	1	25.05044	134848.3	837.4414	oligopeptide
269	WPC	421.6409	2	13.70475	187017.1	841.2673	oligopeptide
270	WPC	281.4298	3	13.73775	179417.3	841.2676	oligopeptide
271	WPC	422.1383	2	2.482167	105587.4	842.2621	oligopeptide
272	WPC	424.1478	2	2.435583	127803.2	846.281	oligopeptide
273	WPC	853.4408	1	2.234092	101980.4	852.4335	oligopeptide
274	WPC	427.2241	2	2.279217	1398397	852.4337	oligopeptide
275	WPC	428.6348	2	12.11498	228419.1	855.255	oligopeptide
276	WPC	870.9519	1	1.606508	146408.4	869.9446	oligopeptide
277	WPC	438.1863	2	15.55887	378076.6	874.358	oligopeptide
278	WPC	875.3653	1	15.55887	463579.5	874.358	oligopeptide
279	WPC	442.2209	2	16.27213	2648898	882.4272	oligopeptide
280	WPC	442.2492	2	21.53531	151686.5	882.4839	oligopeptide
281	WPC	445.2459	2	30.22242	715547.7	888.4773	oligopeptide
282	WPC	889.485	1	30.21413	374161.1	888.4778	oligopeptide
283	WPC	448.2114	2	2.168392	204759.5	894.4083	oligopeptide
284	WPC	449.2378	2	2.904842	255080.1	896.4611	oligopeptide
285	WPC	450.1653	2	15.55887	262727.3	898.316	oligopeptide
286	WPC	450.6574	2	18.57114	206451.7	899.3003	oligopeptide
287	WPC	457.6376	2	13.04848	336701.2	913.2607	oligopeptide
288	WPC	457.6654	2	19.98653	243660.1	913.3162	oligopeptide
289	WPC	921.3709	1	18.95907	412430.1	920.3636	oligopeptide
290	WPC	462.2141	2	1.432192	179743.4	922.4136	oligopeptide
291	WPC	466.7143	2	2.168392	184586.6	931.4141	oligopeptide
292	WPC	938.9391	1	1.606508	102819.6	937.9318	oligopeptide
293	WPC	471.1978	2	22.17535	124159.9	940.3811	oligopeptide
294	WPC	473.168	2	18.95907	146478.8	944.3214	oligopeptide
295	WPC	485.2353	2	2.21185	118105	968.456	oligopeptide
296	WPC	974.8145	1	1.617742	207403.6	973.8072	oligopeptide
297	WPC	990.789	1	1.628758	177840.7	989.7817	oligopeptide
298	WPC	499.2518	2	16.08898	201061	996.4891	oligopeptide
299	WPC	507.1721	2	17.6531	105729.4	1012.33	oligopeptide

300	WPC	514.958	2	1.595175	105251.2	1027.901	oligopeptide	
301	WPC	521.2777	2	17.21345	1825116	1040.541	oligopeptide	
302	WPC	1042.802	1	1.617742	112199.1	1041.794	oligopeptide	
303	WPC	523.7867	2	23.17221	292908.7	1045.559	oligopeptide	
304	WPC	527.7392	2	18.99943	397963.3	1053.464	oligopeptide	
305	WPC	529.7732	2	15.09943	570236.9	1057.532	oligopeptide	
306	WPC	534.282	2	22.13133	281260.2	1066.549	oligopeptide	
307	WPC	548.9517	2	1.595175	108882.7	1095.889	oligopeptide	
308	WPC	550.7942	2	28.70863	915629.7	1099.574	oligopeptide	
309	WPC	1100.581	1	28.72263	112359	1099.574	oligopeptide	
310	WPC	559.2505	2	20.96831	109252.6	1116.486	oligopeptide	
311	WPC	380.1813	3	28.70863	107459.7	1137.522	oligopeptide	
312	WPC	570.2834	2	14.40415	242813.4	1138.552	oligopeptide	
313	WPC	582.9457	2	1.595175	111749	1163.877	oligopeptide	
314	WPC	590.9329	2	1.606508	107728	1179.851	oligopeptide	
315	WPC	611.3502	2	30.66709	247428.1	1220.686	oligopeptide	
316	WPC	616.9397	2	1.606508	106238.3	1231.865	oligopeptide	
317	WPC	623.2974	2	18.85794	1793319	1244.58	oligopeptide	
318	WPC	624.9266	2	1.606508	123266.4	1247.839	oligopeptide	
319	WPC	423.8535	3	18.85794	117316	1268.539	oligopeptide	
320	WPC	644.7018	2	16.91353	157499.9	1287.389	oligopeptide	
321	WPC	650.9333	2	1.595175	110958.2	1299.852	oligopeptide	
322	WPC	658.9205	2	1.606508	109726.8	1315.826	oligopeptide	
323	WPC	660.8836	2	31.72243	1153159	1319.753	oligopeptide	
324	WPC	671.8743	2	31.72243	128154.4	1341.734	oligopeptide	
325	WPC	453.5738	3	31.72243	111530.4	1357.7	oligopeptide	
326	WPC	683.3317	2	16.83296	1638555	1364.649	oligopeptide	
327	WPC	686.2282	2	16.44577	389863.6	1370.442	oligopeptide	
328	WPC	691.8265	2	14.43147	113440.9	1381.638	oligopeptide	
329	WPC	692.9142	2	1.606508	122083.3	1383.814	oligopeptide	
330	WPC	463.8764	3	16.83296	168314.5	1388.607	oligopeptide	
331	WPC	726.9083	2	1.606508	106652.8	1451.802	oligopeptide	
332	WPC	503.5076	3	14.09834	393668.2	1507.501	oligopeptide	
333	WPC	760.9016	2	1.606508	112147.4	1519.789	oligopeptide	
334	WPC	785.3513	2	18.36856	1290617	1568.688	oligopeptide	
335	WPC	794.8955	2	1.606508	110887.6	1587.776	oligopeptide	
336	WPC	531.8893	3	18.38533	218926.1	1592.646	oligopeptide	
337	WPC	828.8889	2	1.617742	102917.6	1655.763	oligopeptide	
ID	Protein	mz	charge	retentiont	raw.abund	mass	type	Match
2	AWPC	231.1708	1	5.273683	1904546	230.1635	di/tripeptide	Y
3	AWPC	231.1708	1	9.576117	3487293	230.1635	di/tripeptide	Y
4	AWPC	231.1708	1	11.98742	2068211	230.1636	di/tripeptide	Y
5	AWPC	233.1497	1	2.196983	149747.6	232.1424	di/tripeptide	Y
6	AWPC	233.1498	1	2.737975	101116.8	232.1426	di/tripeptide	Y
7	AWPC	239.1027	1	2.185483	238919.6	238.0954	di/tripeptide	Y
8	AWPC	239.1029	1	2.420408	158459.9	238.0956	di/tripeptide	Y
10	AWPC	245.1133	1	1.9787	533983.1	244.106	di/tripeptide	Y
11	AWPC	245.1863	1	16.41699	1651426	244.179	di/tripeptide	Y
12	AWPC	245.1865	1	15.88533	416531.8	244.1793	di/tripeptide	Y
13	AWPC	245.1866	1	14.54718	2284778	244.1793	di/tripeptide	Y

14	AWPC	245.1866	1	17.6797	234215.9	244.1793	di/tripeptide	Y
15	AWPC	246.145	1	2.119825	581353.2	245.1377	di/tripeptide	Y
16	AWPC	246.1451	1	2.628433	335570.8	245.1378	di/tripeptide	Y
17	AWPC	246.1453	1	3.307208	193706.1	245.138	di/tripeptide	Y
19	AWPC	247.1113	1	2.381475	315852.3	246.1041	di/tripeptide	Y
20	AWPC	247.129	1	2.185483	501091.9	246.1217	di/tripeptide	Y
21	AWPC	247.1291	1	3.090867	1133145	246.1218	di/tripeptide	Y
22	AWPC	247.1293	1	4.684425	1086499	246.122	di/tripeptide	Y
24	AWPC	250.178	1	34.8633	727241.7	249.1707	di/tripeptide	Y
25	AWPC	250.1781	1	37.30648	241072.2	249.1708	di/tripeptide	Y
26	AWPC	252.9628	1	4.835875	119741.2	251.9555	di/tripeptide	Y
28	AWPC	253.1188	1	9.2984	153476	252.1115	di/tripeptide	Y
30	AWPC	260.1607	1	2.185483	1241601	259.1534	di/tripeptide	Y
31	AWPC	260.1608	1	2.628433	684324	259.1535	di/tripeptide	Y
32	AWPC	260.161	1	3.647733	540904.4	259.1537	di/tripeptide	Y
33	AWPC	260.1971	1	2.185483	119775.7	259.1898	di/tripeptide	Y
34	AWPC	261.1447	1	2.185483	354452.3	260.1375	di/tripeptide	Y
35	AWPC	261.1449	1	3.221208	570225.9	260.1376	di/tripeptide	Y
36	AWPC	261.145	1	5.452467	650286.2	260.1378	di/tripeptide	Y
37	AWPC	262.1399	1	2.090442	198865.8	261.1327	di/tripeptide	Y
38	AWPC	263.143	1	11.49235	1396537	262.1357	di/tripeptide	Y
39	AWPC	263.1967	1	2.185483	127844.8	262.1895	di/tripeptide	Y
41	AWPC	267.1343	1	2.402025	129472.4	266.127	di/tripeptide	Y
42	AWPC	269.1608	1	2.150667	2107226	268.1536	di/tripeptide	Y
43	AWPC	274.1875	1	1.9787	241761.8	273.1802	di/tripeptide	Y
44	AWPC	277.1032	1	1.857775	177563.1	276.096	di/tripeptide	Y
45	AWPC	281.1134	1	2.551392	715060.2	280.1062	di/tripeptide	Y
47	AWPC	288.1925	1	16.07473	218052.5	287.1852	di/tripeptide	Y
48	AWPC	290.1714	1	2.677217	1816774	289.1641	di/tripeptide	Y
50	AWPC	294.1451	1	2.324908	219033.8	293.1379	di/tripeptide	Y
51	AWPC	295.1295	1	3.184558	231856.9	294.1222	di/tripeptide	Y
52	AWPC	295.1657	1	16.41699	422982.9	294.1585	di/tripeptide	Y
53	AWPC	300.192	1	2.573417	5790506	299.1847	di/tripeptide	Y
54	AWPC	300.1928	1	15.12667	238374.1	299.1855	di/tripeptide	Y
56	AWPC	302.2082	1	15.14083	321660.8	301.2009	tri/oligopeptic	Y
57	AWPC	302.2084	1	19.86491	120421.9	301.2011	tri/oligopeptic	Y
58	AWPC	303.1666	1	2.196983	224317.9	302.1594	tri/oligopeptic	Y
59	AWPC	304.1506	1	2.402025	919383.1	303.1433	tri/oligopeptic	Y
60	AWPC	304.1509	1	4.878617	1044883	303.1437	tri/oligopeptic	Y
62	AWPC	316.2237	1	13.87716	909218.2	315.2164	tri/oligopeptic	Y
63	AWPC	318.1664	1	2.520325	1004240	317.1591	tri/oligopeptic	Y
64	AWPC	318.1665	1	2.324908	2330787	317.1592	tri/oligopeptic	Y
66	AWPC	318.1821	1	22.56183	1680626	317.1748	tri/oligopeptic	Y
69	AWPC	319.1615	1	2.162533	390958	318.1542	tri/oligopeptic	Y
71	AWPC	330.2396	1	18.41157	314976.7	329.2324	tri/oligopeptic	Y
72	AWPC	331.1613	1	2.078558	675098.2	330.1541	tri/oligopeptic	Y
73	AWPC	331.1657	1	2.795458	156774.5	330.1584	tri/oligopeptic	Y
74	AWPC	331.2851	1	38.45992	160207.6	330.2778	tri/oligopeptic	Y
75	AWPC	332.1818	1	2.599758	3512877	331.1745	tri/oligopeptic	Y
76	AWPC	332.2186	1	3.034567	1654703	331.2113	tri/oligopeptic	Y

77	AWPC	332.2187	1	3.361925	2609927	331.2114	tri/oligopeptic	Y
80	AWPC	334.1612	1	2.208867	123167.6	333.1539	tri/oligopeptic	Y
81	AWPC	334.1613	1	3.184558	1265698	333.1541	tri/oligopeptic	Y
82	AWPC	342.2399	1	21.81455	1036480	341.2326	tri/oligopeptic	Y
83	AWPC	343.1239	1	1.720125	178373.7	342.1167	tri/oligopeptic	Y
84	AWPC	344.1826	1	5.57985	156489.5	343.1753	tri/oligopeptic	Y
85	AWPC	344.2555	1	21.54573	1062855	343.2482	tri/oligopeptic	Y
86	AWPC	344.2556	1	20.18665	107348.8	343.2483	tri/oligopeptic	Y
87	AWPC	349.1723	1	1.857775	110858.2	348.165	tri/oligopeptic	Y
88	AWPC	350.1197	1	1.827958	484545.1	349.1125	tri/oligopeptic	Y
89	AWPC	358.2712	1	24.02033	197413.3	357.2639	tri/oligopeptic	Y
90	AWPC	359.2293	1	2.185483	444567.2	358.2221	tri/oligopeptic	Y
91	AWPC	360.196	1	18.72603	1823035	359.1888	tri/oligopeptic	Y
92	AWPC	360.2134	1	2.701133	145799.1	359.2061	tri/oligopeptic	Y
93	AWPC	360.2137	1	13.63674	992379	359.2064	tri/oligopeptic	Y
94	AWPC	363.1555	1	2.185483	132566.2	362.1482	tri/oligopeptic	Y
95	AWPC	365.1366	1	37.81322	104981.3	364.1293	tri/oligopeptic	Y
96	AWPC	366.214	1	2.185483	257472.6	365.2067	tri/oligopeptic	Y
97	AWPC	374.2297	1	19.33054	645905	373.2224	tri/oligopeptic	Y
99	AWPC	376.1467	1	1.720125	112945.9	375.1395	tri/oligopeptic	Y
100	AWPC	376.1719	1	2.196983	591415	375.1646	tri/oligopeptic	Y
102	AWPC	381.0801	1	1.674667	122802.6	380.0728	tri/oligopeptic	Y
103	AWPC	387.2246	1	14.00868	7860160	386.2173	tri/oligopeptic	Y
104	AWPC	389.2306	1	13.9912	194084.4	388.2233	tri/oligopeptic	Y
105	AWPC	390.1876	1	2.313533	213222.5	389.1803	tri/oligopeptic	Y
106	AWPC	395.0394	1	1.574158	210659	394.0321	tri/oligopeptic	Y
107	AWPC	399.2612	1	18.53898	305607.4	398.254	tri/oligopeptic	Y
109	AWPC	402.2354	1	11.35857	1794719	401.2281	tri/oligopeptic	Y
111	AWPC	405.199	1	4.148108	162586.7	404.1918	tri/oligopeptic	Y
112	AWPC	415.2118	1	36.47435	323153.5	414.2046	tri/oligopeptic	Y
113	AWPC	417.2352	1	22.09608	549731.4	416.2279	tri/oligopeptic	Y
114	AWPC	420.2094	1	2.456142	118240	419.2021	tri/oligopeptic	Y
115	AWPC	421.1932	1	2.196983	695644.3	420.1859	tri/oligopeptic	Y
117	AWPC	428.287	1	2.196983	156737.2	427.2797	tri/oligopeptic	Y
118	AWPC	429.272	1	18.59953	102461.8	428.2647	tri/oligopeptic	Y
119	AWPC	431.2332	1	20.34427	11620926	430.2259	tri/oligopeptic	Y
121	AWPC	433.1931	1	2.85885	2829224	432.1858	tri/oligopeptic	Y
122	AWPC	434.1884	1	2.045575	169781.7	433.1812	tri/oligopeptic	Y
124	AWPC	444.2464	1	10.45416	490691.3	443.2392	tri/oligopeptic	Y
125	AWPC	445.2305	1	14.20219	541808.9	444.2232	tri/oligopeptic	Y
126	AWPC	445.2662	1	14.23033	1305963	444.2589	tri/oligopeptic	Y
127	AWPC	445.3027	1	18.98931	842344.3	444.2955	tri/oligopeptic	Y
128	AWPC	223.6254	2	2.138775	140440.6	445.2363	tri/oligopeptic	Y
129	AWPC	446.2611	1	2.196983	256960.4	445.2538	tri/oligopeptic	Y
130	AWPC	223.6342	2	2.208867	250865.4	445.2539	tri/oligopeptic	Y
131	AWPC	449.2078	1	17.59765	115730.9	448.2005	tri/oligopeptic	Y
133	AWPC	461.3004	1	37.31842	107679.2	460.2932	tri/oligopeptic	Y
134	AWPC	463.0268	1	1.607808	274127.4	462.0195	tri/oligopeptic	Y
136	AWPC	236.0739	2	2.852875	102948.4	470.1332	tri/oligopeptic	Y
137	AWPC	472.2412	1	11.76702	525032.6	471.234	tri/oligopeptic	Y

138	AWPC	476.2906	1	20.34427	135478.9	475.2833	tri/oligopeptic	Y
139	AWPC	477.183	1	2.185483	107041.6	476.1758	tri/oligopeptic	Y
140	AWPC	478.2307	1	14.85351	422627.8	477.2235	tri/oligopeptic	Y
141	AWPC	479.3103	1	37.13739	177468.5	478.303	tri/oligopeptic	Y
144	AWPC	488.2517	1	19.13527	134694.2	487.2444	tri/oligopeptic	Y
146	AWPC	489.2559	1	17.17401	1500020	488.2487	tri/oligopeptic	Y
147	AWPC	491.2873	1	22.64707	149780.7	490.28	tri/oligopeptic	Y
149	AWPC	492.2304	1	2.196983	114016.4	491.2231	tri/oligopeptic	Y
154	AWPC	513.3046	1	19.64597	164203.6	512.2974	tri/oligopeptic	Y
155	AWPC	515.2464	1	2.558717	269343.6	514.2391	tri/oligopeptic	Y
156	AWPC	515.2914	1	37.12387	101219.7	514.2841	tri/oligopeptic	Y
159	AWPC	520.2781	1	21.17131	104791	519.2709	tri/oligopeptic	Y
161	AWPC	264.1748	2	35.27389	115760.8	526.335	tri/oligopeptic	Y
162	AWPC	265.1712	2	2.501658	341798.6	528.3278	tri/oligopeptic	Y
164	AWPC	265.6687	2	2.196983	138299.5	529.3229	tri/oligopeptic	Y
165	AWPC	531.0142	1	1.596708	334776.1	530.0069	tri/oligopeptic	Y
166	AWPC	531.3871	1	37.83449	109085.3	530.3798	tri/oligopeptic	Y
167	AWPC	532.2251	1	2.196983	700561.9	531.2178	tri/oligopeptic	Y
168	AWPC	539.2359	1	8.930575	231457	538.2286	tri/oligopeptic	Y
171	AWPC	545.2939	1	18.70098	398680.8	544.2866	tri/oligopeptic	Y
172	AWPC	273.1507	2	18.68868	189785.6	544.2868	tri/oligopeptic	Y
173	AWPC	546.2783	1	22.25314	102459.5	545.271	tri/oligopeptic	Y
174	AWPC	274.1677	2	35.93865	114180.2	546.3209	tri/oligopeptic	Y
176	AWPC	553.3361	1	23.57234	1373638	552.3288	tri/oligopeptic	Y
179	AWPC	562.2734	1	11.34012	178821.2	561.2661	tri/oligopeptic	Y
180	AWPC	565.2106	1	2.011192	191006.5	564.2033	tri/oligopeptic	Y
181	AWPC	565.2623	1	16.59621	4189464	564.255	tri/oligopeptic	Y
182	AWPC	283.1349	2	16.60434	1205912	564.2552	tri/oligopeptic	Y
184	AWPC	286.1582	2	2.174033	514510.7	570.3018	tri/oligopeptic	Y
185	AWPC	571.3091	1	2.185483	332368.2	570.3018	tri/oligopeptic	Y
186	AWPC	571.3105	1	16.06048	112694.3	570.3032	tri/oligopeptic	Y
188	AWPC	578.232	1	9.07675	174934.4	577.2247	tri/oligopeptic	Y
190	AWPC	584.303	1	2.185483	117259	583.2957	tri/oligopeptic	Y
191	AWPC	295.114	2	16.60434	332666.2	588.2135	tri/oligopeptic	Y
192	AWPC	590.2688	1	19.60093	145205.4	589.2615	tri/oligopeptic	Y
194	AWPC	599.002	1	1.607808	320494.4	597.9947	tri/oligopeptic	Y
195	AWPC	300.1609	2	1.423325	157682.3	598.3073	tri/oligopeptic	Y
196	AWPC	302.1085	2	16.59621	295941.2	602.2025	tri/oligopeptic	Y
197	AWPC	607.3834	1	20.79872	1389651	606.3761	tri/oligopeptic	Y
198	AWPC	304.1954	2	20.80518	167911.7	606.3763	tri/oligopeptic	Y
201	AWPC	310.0947	2	16.59621	116601.4	618.1748	oligopeptide	Y
202	AWPC	314.1798	2	26.72738	171703.5	626.3451	oligopeptide	Y
203	AWPC	627.3524	1	26.70038	599130	626.3451	oligopeptide	Y
204	AWPC	628.3317	1	19.14271	736826.5	627.3245	oligopeptide	Y
205	AWPC	314.6695	2	19.15015	186067.7	627.3245	oligopeptide	Y
207	AWPC	640.2975	1	19.93092	145322.3	639.2902	oligopeptide	Y
208	AWPC	643.3318	1	20.73205	110562.9	642.3246	oligopeptide	Y
210	AWPC	646.3048	1	2.279333	200445.1	645.2975	oligopeptide	Y
212	AWPC	652.3209	1	21.66942	192740.3	651.3136	oligopeptide	Y
214	AWPC	328.1577	2	15.62716	235569	654.3008	oligopeptide	Y

215	AWPC	658.3422	1	20.43847	370504.2	657.3349	oligopeptide	Y
216	AWPC	659.2895	1	14.01757	183256.3	658.2822	oligopeptide	Y
218	AWPC	666.9895	1	1.607808	298406.9	665.9822	oligopeptide	Y
219	AWPC	668.3623	1	20.00098	428177.6	667.355	oligopeptide	Y
221	AWPC	678.347	1	22.66536	1410889	677.3398	oligopeptide	Y
222	AWPC	339.6773	2	22.67218	465712.6	677.34	oligopeptide	Y
224	AWPC	348.1777	2	2.232583	173009.7	694.3408	oligopeptide	Y
225	AWPC	349.198	2	12.22493	482964.5	696.3814	oligopeptide	Y
228	AWPC	701.3478	1	16.53653	555605.9	700.3406	oligopeptide	Y
229	AWPC	351.1776	2	16.52618	696887.4	700.3407	oligopeptide	Y
230	AWPC	351.6563	2	22.67218	267837	701.298	oligopeptide	Y
233	AWPC	356.6847	2	2.02265	332281.8	711.3549	oligopeptide	Y
235	AWPC	358.6509	2	22.67218	125669.6	715.2872	oligopeptide	Y
237	AWPC	363.1464	2	1.955233	654970.3	724.2783	oligopeptide	Y
238	AWPC	363.1565	2	16.52618	306530.2	724.2985	oligopeptide	Y
240	AWPC	734.9771	1	1.607808	257477.4	733.9699	oligopeptide	Y
242	AWPC	370.1511	2	16.52618	140387.6	738.2877	oligopeptide	Y
244	AWPC	374.6898	2	20.78355	265838.7	747.3651	oligopeptide	Y
245	AWPC	376.7003	2	2.220583	190545.2	751.3861	oligopeptide	Y
247	AWPC	377.7298	2	29.30544	607690.8	753.445	oligopeptide	Y
248	AWPC	754.4523	1	29.29083	1577483	753.4451	oligopeptide	Y
252	AWPC	769.4105	1	17.80113	247805.7	768.4032	oligopeptide	Y
253	AWPC	770.3676	1	2.185483	127025.5	769.3603	oligopeptide	Y
254	AWPC	385.6876	2	2.220583	230381.3	769.3606	oligopeptide	Y
256	AWPC	389.728	2	23.55654	240793.2	777.4414	oligopeptide	Y
257	AWPC	392.6952	2	2.208867	148575.3	783.3758	oligopeptide	Y
258	AWPC	802.9646	1	1.596708	222248.6	801.9573	oligopeptide	Y
259	AWPC	804.3277	1	13.694	1214111	803.3204	oligopeptide	Y
260	AWPC	402.6675	2	13.7027	108278.5	803.3205	oligopeptide	Y
261	AWPC	406.7113	2	14.17374	101766.7	811.408	oligopeptide	Y
266	AWPC	414.6465	2	13.694	438256	827.2784	oligopeptide	Y
267	AWPC	419.7279	2	25.0595	292635.2	837.4413	oligopeptide	Y
274	AWPC	427.2241	2	2.279333	962064.3	852.4337	oligopeptide	Y
275	AWPC	428.6348	2	12.05813	135454.3	855.255	oligopeptide	Y
276	AWPC	870.9519	1	1.607808	154687	869.9446	oligopeptide	Y
277	AWPC	438.1863	2	15.54144	229065.9	874.358	oligopeptide	Y
278	AWPC	875.3653	1	15.54144	269396	874.358	oligopeptide	Y
279	AWPC	442.2209	2	16.2593	1780225	882.4272	oligopeptide	Y
280	AWPC	442.2492	2	21.52652	113515.2	882.4839	oligopeptide	Y
281	AWPC	445.2459	2	30.22275	959019.8	888.4773	oligopeptide	Y
282	AWPC	889.485	1	30.21368	506720.4	888.4778	oligopeptide	Y
283	AWPC	448.2114	2	2.162533	122875	894.4083	oligopeptide	Y
285	AWPC	450.1653	2	15.54144	159143.8	898.316	oligopeptide	Y
287	AWPC	457.6376	2	12.95815	254773	913.2607	oligopeptide	Y
289	AWPC	921.3709	1	18.95233	196472.6	920.3636	oligopeptide	Y
290	AWPC	462.2141	2	1.431408	168902.6	922.4136	oligopeptide	Y
296	AWPC	974.8145	1	1.618625	207220.9	973.8072	oligopeptide	Y
297	AWPC	990.789	1	1.62945	166308.8	989.7817	oligopeptide	Y
300	AWPC	514.958	2	1.596708	107008.4	1027.901	oligopeptide	Y
301	AWPC	521.2777	2	17.20263	425634.1	1040.541	oligopeptide	Y

302	AWPC	1042.802	1	1.618625	111663.7	1041.794	oligopeptide	Y
305	AWPC	529.7732	2	15.07979	405671.7	1057.532	oligopeptide	Y
307	AWPC	548.9517	2	1.596708	116785.2	1095.889	oligopeptide	Y
308	AWPC	550.7942	2	28.71115	245930.1	1099.574	oligopeptide	Y
312	AWPC	570.2834	2	14.40222	127940.4	1138.552	oligopeptide	Y
313	AWPC	582.9457	2	1.596708	117309	1163.877	oligopeptide	Y
314	AWPC	590.9329	2	1.607808	120091.2	1179.851	oligopeptide	Y
316	AWPC	616.9397	2	1.607808	122188	1231.865	oligopeptide	Y
317	AWPC	623.2974	2	18.84983	239925.4	1244.58	oligopeptide	Y
318	AWPC	624.9266	2	1.607808	134932.7	1247.839	oligopeptide	Y
321	AWPC	650.9333	2	1.596708	110882	1299.852	oligopeptide	Y
322	AWPC	658.9205	2	1.607808	122920.3	1315.826	oligopeptide	Y
323	AWPC	660.8836	2	31.73455	624798.9	1319.753	oligopeptide	Y
326	AWPC	683.3317	2	16.82833	571515.6	1364.649	oligopeptide	Y
327	AWPC	686.2282	2	16.43336	288667.5	1370.442	oligopeptide	Y
329	AWPC	692.9142	2	1.607808	120101.2	1383.814	oligopeptide	Y
331	AWPC	726.9083	2	1.607808	114294.6	1451.802	oligopeptide	Y
332	AWPC	503.5076	3	14.11873	140108.3	1507.501	oligopeptide	Y
333	AWPC	760.9016	2	1.607808	118725	1519.789	oligopeptide	Y
334	AWPC	785.3513	2	18.35963	433492.2	1568.688	oligopeptide	Y
335	AWPC	794.8955	2	1.607808	108313.6	1587.776	oligopeptide	Y
337	AWPC	828.8889	2	1.618625	102009.8	1655.763	oligopeptide	Y
338	AWPC	229.1552	1	11.91688	121366.2	228.148	di/tripeptide	N
339	AWPC	262.1035	1	1.720125	102998.6	261.0963	di/tripeptide	N
340	AWPC	269.1134	1	2.185483	119958.8	268.1061	di/tripeptide	N
341	AWPC	276.1556	1	2.208867	112810.7	275.1483	di/tripeptide	N
342	AWPC	313.176	1	2.185483	117614.6	312.1687	tri/oligopeptic	N
343	AWPC	328.2242	1	15.87005	121495.2	327.217	tri/oligopeptic	N
344	AWPC	328.234	1	15.48093	100780.4	327.2267	tri/oligopeptic	N
345	AWPC	332.219	1	19.44008	153304.3	331.2118	tri/oligopeptic	N
346	AWPC	334.1405	1	13.78616	142047.1	333.1333	tri/oligopeptic	N
347	AWPC	342.24	1	20.11797	163910.2	341.2327	tri/oligopeptic	N
348	AWPC	344.1826	1	4.819033	135656.2	343.1753	tri/oligopeptic	N
349	AWPC	346.1981	1	9.598242	143731.4	345.1908	tri/oligopeptic	N
350	AWPC	359.2298	1	14.24358	158602.9	358.2225	tri/oligopeptic	N
351	AWPC	360.1882	1	2.056958	103364.7	359.1809	tri/oligopeptic	N
352	AWPC	360.2141	1	20.61665	118805.8	359.2068	tri/oligopeptic	N
353	AWPC	366.167	1	11.55632	130143.5	365.1597	tri/oligopeptic	N
354	AWPC	398.1561	1	2.208867	109932.8	397.1489	tri/oligopeptic	N
355	AWPC	443.2139	1	2.196983	113091.1	442.2066	tri/oligopeptic	N
356	AWPC	447.2281	1	13.71706	115276.4	446.2208	tri/oligopeptic	N
357	AWPC	226.1462	2	38.45992	105229.5	450.2778	tri/oligopeptic	N
358	AWPC	475.2236	1	20.62424	117275.7	474.2163	tri/oligopeptic	N
359	AWPC	482.3239	1	37.18689	108301.4	481.3166	tri/oligopeptic	N
360	AWPC	489.2719	1	22.1195	265583.6	488.2646	tri/oligopeptic	N
361	AWPC	505.3036	1	24.77722	154657.1	504.2963	tri/oligopeptic	N
362	AWPC	515.2837	1	15.3988	279580.5	514.2764	tri/oligopeptic	N
363	AWPC	524.3719	1	38.22528	100147.7	523.3646	tri/oligopeptic	N
364	AWPC	526.3143	1	36.96188	169334.5	525.307	tri/oligopeptic	N
365	AWPC	530.299	1	27.48286	240105.7	529.2917	tri/oligopeptic	N

366	AWPC	269.6293	2	2.511142	279813.3	537.2441	tri/oligopeptic	N
367	AWPC	560.2573	1	2.812683	121798.1	559.25	tri/oligopeptic	N
368	AWPC	560.2946	1	16.79592	155692.6	559.2873	tri/oligopeptic	N
369	AWPC	288.6796	2	22.6551	145991	575.3446	tri/oligopeptic	N
370	AWPC	585.326	1	18.40532	100702.6	584.3188	tri/oligopeptic	N
371	AWPC	590.2788	1	2.587317	118720.2	589.2716	tri/oligopeptic	N
372	AWPC	318.117	2	16.59621	106652.5	634.2194	oligopeptide	N
373	AWPC	326.7062	2	20.80518	111565.6	651.3979	oligopeptide	N
374	AWPC	329.6748	2	20.44598	122488.1	657.335	oligopeptide	N
375	AWPC	341.654	2	20.44598	102843.9	681.2933	oligopeptide	N
376	AWPC	342.7006	2	2.196983	167182.1	683.3866	oligopeptide	N
377	AWPC	343.1083	2	16.59621	104361.2	684.2021	oligopeptide	N
378	AWPC	345.2224	2	27.67829	131256.2	688.4302	oligopeptide	N
379	AWPC	379.7094	2	27.11153	109722.3	757.4042	oligopeptide	N
380	AWPC	407.2225	2	13.20707	372110.4	812.4304	oligopeptide	N
381	AWPC	867.3724	1	36.47435	101430.5	866.3651	oligopeptide	N
382	AWPC	457.2248	2	30.20714	114774.5	912.4351	oligopeptide	N
383	AWPC	464.2196	2	30.20714	105427.2	926.4247	oligopeptide	N
384	AWPC	485.2676	2	19.41461	142236.9	968.5207	oligopeptide	N
385	AWPC	503.2729	2	21.74028	105311	1004.531	oligopeptide	N
386	AWPC	684.9273	2	1.607808	109136.6	1367.84	oligopeptide	N

ID	Protein	mz	charge	retentionti	raw.abund	mass	type	Match
3	Egg	231.1708	1	9.488808	647117.7	230.1635	di/tripeptide	Y
5	Egg	233.1499	1	2.459775	140593.6	232.1426	di/tripeptide	Y
10	Egg	245.1136	1	2.113563	407009.6	244.1063	di/tripeptide	Y
11	Egg	245.1864	1	16.87238	354337.9	244.1791	di/tripeptide	Y
11	Egg	245.1865	1	16.65326	713334.5	244.1792	di/tripeptide	Y
12	Egg	245.1868	1	16.14035	107853.7	244.1796	di/tripeptide	Y
13	Egg	245.1865	1	14.88878	555098.5	244.1793	di/tripeptide	Y
14	Egg	245.1867	1	17.88473	143449.7	244.1794	di/tripeptide	Y
15	Egg	246.1452	1	2.351929	587220.1	245.138	di/tripeptide	Y
17	Egg	246.1455	1	3.112496	293727.7	245.1382	di/tripeptide	Y
20	Egg	247.1293	1	2.369725	394111.2	246.122	di/tripeptide	Y
21	Egg	247.1297	1	3.511192	126537.4	246.1224	di/tripeptide	Y
22	Egg	247.1297	1	4.883296	125044.1	246.1224	di/tripeptide	Y
24	Egg	250.1782	1	34.98545	989152.1	249.1709	di/tripeptide	Y
24	Egg	250.1784	1	34.5071	118855.7	249.1711	di/tripeptide	Y
27	Egg	253.1189	1	2.636454	171898.1	252.1116	di/tripeptide	Y
31	Egg	260.161	1	2.441813	370699.2	259.1537	di/tripeptide	Y
31	Egg	260.1611	1	2.851121	306393	259.1539	di/tripeptide	Y
32	Egg	260.1612	1	3.439021	181998	259.1539	di/tripeptide	Y
33	Egg	260.1973	1	2.422696	255141.3	259.19	di/tripeptide	Y
34	Egg	261.1451	1	2.441813	164964.7	260.1378	di/tripeptide	Y
35	Egg	261.1451	1	3.282875	115951.7	260.1379	di/tripeptide	Y
37	Egg	262.1402	1	2.184104	217444.3	261.133	di/tripeptide	Y
40	Egg	265.1556	1	16.49057	109540.5	264.1484	di/tripeptide	Y
42	Egg	269.1612	1	2.422696	211595.8	268.154	di/tripeptide	Y
43	Egg	274.1878	1	2.025771	153394	273.1805	di/tripeptide	Y
44	Egg	277.1035	1	1.948421	410373.7	276.0962	di/tripeptide	Y
48	Egg	290.1717	1	2.680971	104287.9	289.1644	di/tripeptide	Y

48	Egg	290.1718	1	3.156933	159062.1	289.1645	di/tripeptide	Y
52	Egg	295.166	1	16.64235	373969.1	294.1587	di/tripeptide	Y
56	Egg	302.2082	1	15.45535	465214	301.201	tri/oligopeptic	Y
57	Egg	302.2081	1	19.95505	1480326	301.2009	tri/oligopeptic	Y
58	Egg	303.1669	1	2.422696	453670.7	302.1596	tri/oligopeptic	Y
59	Egg	304.1509	1	2.441813	156193.7	303.1437	tri/oligopeptic	Y
63	Egg	318.1669	1	2.680971	146912.9	317.1596	tri/oligopeptic	Y
66	Egg	318.1822	1	22.63143	402724.2	317.175	tri/oligopeptic	Y
69	Egg	319.1619	1	2.141692	121485.3	318.1546	tri/oligopeptic	Y
71	Egg	330.2398	1	18.56749	181715	329.2326	tri/oligopeptic	Y
72	Egg	331.1617	1	2.334708	120000.9	330.1544	tri/oligopeptic	Y
74	Egg	331.2851	1	38.21228	367917.6	330.2778	tri/oligopeptic	Y
78	Egg	333.1411	1	1.97625	120045.3	332.1338	tri/oligopeptic	Y
80	Egg	334.1615	1	2.351929	708292.8	333.1542	tri/oligopeptic	Y
82	Egg	342.2398	1	21.35965	446875.6	341.2325	tri/oligopeptic	Y
95	Egg	365.1368	1	37.61193	115452.8	364.1296	tri/oligopeptic	Y
112	Egg	415.2122	1	36.27107	204098.5	414.2049	tri/oligopeptic	Y
133	Egg	461.3008	1	37.40978	156877.5	460.2935	tri/oligopeptic	Y
141	Egg	479.3108	1	36.86001	136703.6	478.3035	tri/oligopeptic	Y
144	Egg	488.2515	1	19.22683	192323.6	487.2443	tri/oligopeptic	Y
151	Egg	500.2884	1	25.21228	118274.9	499.2812	tri/oligopeptic	Y
154	Egg	513.3045	1	19.78508	245122.1	512.2972	tri/oligopeptic	Y
156	Egg	515.2912	1	36.72567	161844.9	514.2839	tri/oligopeptic	Y
159	Egg	520.2781	1	21.22253	111620.6	519.2708	tri/oligopeptic	Y
161	Egg	264.1749	2	34.94	172257.5	526.3352	tri/oligopeptic	Y
165	Egg	531.0146	1	1.595308	131810.9	530.0074	tri/oligopeptic	Y
166	Egg	531.3875	1	37.63103	117303.9	530.3802	tri/oligopeptic	Y
174	Egg	274.1678	2	35.72245	148287.6	546.3211	tri/oligopeptic	Y
208	Egg	643.3319	1	20.7716	166196.6	642.3246	oligopeptide	Y
218	Egg	666.9901	1	1.582842	120058.7	665.9829	oligopeptide	Y
240	Egg	734.9776	1	1.582842	105516.7	733.9703	oligopeptide	Y
297	Egg	990.7898	1	1.607821	238549.1	989.7825	oligopeptide	Y
338	Egg	223.1081	1	9.328596	122462.4	222.1008	dipeptide	N
339	Egg	223.1082	1	8.756054	180595.1	222.1009	dipeptide	N
340	Egg	223.1085	1	6.137638	276546.2	222.1012	dipeptide	N
341	Egg	226.952	1	1.539604	298007.3	225.9447	di/tripeptide	N
342	Egg	228.1961	1	34.98545	2436024	227.1888	di/tripeptide	N
343	Egg	228.1963	1	34.49176	504525.6	227.1891	di/tripeptide	N
344	Egg	229.1552	1	7.86515	117688.6	228.1479	di/tripeptide	N
345	Egg	229.1552	1	8.630525	120074.5	228.1479	di/tripeptide	N
346	Egg	229.1553	1	12.86783	212998.9	228.148	di/tripeptide	N
347	Egg	229.1554	1	6.156467	201637.8	228.1482	di/tripeptide	N
348	Egg	231.1709	1	12.83317	1231873	230.1636	di/tripeptide	N
349	Egg	231.1709	1	11.22379	400662.1	230.1637	di/tripeptide	N
350	Egg	231.1709	1	10.71672	221939.2	230.1637	di/tripeptide	N
351	Egg	231.171	1	13.19696	333681.5	230.1637	di/tripeptide	N
352	Egg	231.1711	1	6.514313	460594.7	230.1639	di/tripeptide	N
353	Egg	232.1295	1	2.422696	487036.3	231.1222	di/tripeptide	N
354	Egg	232.1295	1	1.937763	192601.6	231.1223	di/tripeptide	N
355	Egg	233.1136	1	2.141692	182744.7	232.1063	di/tripeptide	N

356	Egg	233.1136	1	2.459775	1077728	232.1063	di/tripeptide	N
357	Egg	237.0908	1	2.404792	122608.3	236.0835	di/tripeptide	N
358	Egg	245.1516	1	36.32401	101706	244.1443	di/tripeptide	N
359	Egg	246.1453	1	5.439663	165804.7	245.138	di/tripeptide	N
360	Egg	246.1816	1	1.937763	353879.6	245.1744	di/tripeptide	N
361	Egg	247.1296	1	5.723488	204767.8	246.1223	di/tripeptide	N
362	Egg	248.1245	1	2.071563	146734	247.1172	di/tripeptide	N
363	Egg	249.1085	1	1.925296	165941.8	248.1012	di/tripeptide	N
364	Egg	249.1271	1	7.513367	182253.8	248.1198	di/tripeptide	N
365	Egg	249.1274	1	3.304954	180246.7	248.1201	di/tripeptide	N
366	Egg	252.963	1	8.253229	133296.2	251.9557	di/tripeptide	N
367	Egg	253.1188	1	10.20286	233143.5	252.1115	di/tripeptide	N
368	Egg	255.1456	1	1.925296	125452.5	254.1383	di/tripeptide	N
369	Egg	260.1609	1	5.747083	145905.8	259.1537	di/tripeptide	N
370	Egg	260.1611	1	3.191608	170873.6	259.1539	di/tripeptide	N
371	Egg	261.1448	1	8.563958	108526.4	260.1375	di/tripeptide	N
372	Egg	261.145	1	7.744004	115965.5	260.1377	di/tripeptide	N
373	Egg	261.1454	1	6.447513	250603.4	260.1381	di/tripeptide	N
374	Egg	263.1396	1	14.97629	434492.3	262.1323	di/tripeptide	N
375	Egg	263.1398	1	14.78566	416013.8	262.1325	di/tripeptide	N
376	Egg	263.143	1	12.41498	1039331	262.1358	di/tripeptide	N
377	Egg	263.1432	1	13.7246	142990.5	262.1359	di/tripeptide	N
378	Egg	263.1969	1	2.703242	192239.8	262.1896	di/tripeptide	N
379	Egg	276.1196	1	1.843308	119800.1	275.1123	di/tripeptide	N
380	Egg	276.1353	1	17.3745	105052.8	275.128	di/tripeptide	N
381	Egg	276.156	1	2.441813	102746.1	275.1487	di/tripeptide	N
382	Egg	276.1563	1	4.487554	147229.1	275.149	di/tripeptide	N
383	Egg	279.1709	1	20.02722	256947.2	278.1636	di/tripeptide	N
384	Egg	280.1298	1	10.78588	111207.5	279.1226	di/tripeptide	N
385	Egg	281.1501	1	10.02999	182673	280.1429	di/tripeptide	N
386	Egg	281.1506	1	8.721442	167735.5	280.1434	di/tripeptide	N
387	Egg	290.1352	1	2.441813	252845	289.1279	di/tripeptide	N
388	Egg	290.1717	1	7.554021	100872.7	289.1644	di/tripeptide	N
389	Egg	290.1719	1	7.9355	108408.5	289.1646	di/tripeptide	N
390	Egg	295.1292	1	8.524492	132116.7	294.1219	di/tripeptide	N
391	Egg	295.1294	1	8.984521	122862.2	294.1221	di/tripeptide	N
392	Egg	295.1296	1	13.02811	306754.3	294.1224	di/tripeptide	N
393	Egg	295.1299	1	13.48731	100706.1	294.1227	di/tripeptide	N
394	Egg	295.13	1	3.776679	263360.7	294.1227	di/tripeptide	N
395	Egg	302.1715	1	7.657067	139564.3	301.1642	tri/oligopeptic	N
396	Egg	302.1716	1	2.441813	241226.1	301.1643	tri/oligopeptic	N
397	Egg	302.1717	1	6.207183	209817.9	301.1644	tri/oligopeptic	N
398	Egg	302.2084	1	18.89318	623114.8	301.2011	tri/oligopeptic	N
399	Egg	303.1672	1	4.251367	315897.2	302.1599	tri/oligopeptic	N
400	Egg	304.1872	1	6.137638	281816	303.1799	tri/oligopeptic	N
401	Egg	304.1873	1	2.441813	362980.3	303.18	tri/oligopeptic	N
402	Egg	304.1875	1	7.366421	464493.6	303.1802	tri/oligopeptic	N
403	Egg	306.1301	1	1.97625	354910.7	305.1228	tri/oligopeptic	N
404	Egg	310.3111	1	36.70178	240558.4	309.3038	tri/oligopeptic	N
405	Egg	314.2083	1	13.7246	342465.7	313.2011	tri/oligopeptic	N

406	Egg	317.1827	1	2.587379	591801.8	316.1754	tri/oligopeptic	N
407	Egg	317.1827	1	5.395513	287398.7	316.1754	tri/oligopeptic	N
408	Egg	317.1827	1	6.502104	288005.9	316.1754	tri/oligopeptic	N
409	Egg	317.219	1	4.856913	104203.9	316.2118	tri/oligopeptic	N
410	Egg	318.149	1	12.38957	2041942	317.1418	tri/oligopeptic	N
411	Egg	318.1667	1	5.618017	108897.8	317.1594	tri/oligopeptic	N
412	Egg	318.1669	1	10.51695	121197.4	317.1596	tri/oligopeptic	N
413	Egg	320.1459	1	2.351929	145514.5	319.1386	tri/oligopeptic	N
414	Egg	322.1437	1	6.104546	144827	321.1364	tri/oligopeptic	N
415	Egg	322.144	1	2.680971	260315.8	321.1367	tri/oligopeptic	N
416	Egg	331.1657	1	5.730913	314918.9	330.1584	tri/oligopeptic	N
417	Egg	331.1987	1	11.90143	224286.8	330.1914	tri/oligopeptic	N
418	Egg	332.2189	1	15.40528	171466.6	331.2116	tri/oligopeptic	N
419	Egg	332.2191	1	18.77735	122374.4	331.2118	tri/oligopeptic	N
420	Egg	332.2191	1	19.60275	133504	331.2118	tri/oligopeptic	N
421	Egg	333.1775	1	2.422696	547044.5	332.1702	tri/oligopeptic	N
422	Egg	333.1777	1	2.96645	334210.3	332.1704	tri/oligopeptic	N
423	Egg	334.1406	1	14.24543	804958.7	333.1333	tri/oligopeptic	N
424	Egg	334.1804	1	15.85984	351842.5	333.1731	tri/oligopeptic	N
425	Egg	334.1805	1	16.07056	142089.7	333.1732	tri/oligopeptic	N
426	Egg	335.1567	1	1.960788	190202.8	334.1495	tri/oligopeptic	N
427	Egg	338.3423	1	36.4099	8711581	337.3351	tri/oligopeptic	N
428	Egg	338.3427	1	38.97075	3861406	337.3354	tri/oligopeptic	N
429	Egg	340.3582	1	36.4099	108478.9	339.3509	tri/oligopeptic	N
430	Egg	343.2964	1	35.28562	106005.2	342.2892	tri/oligopeptic	N
431	Egg	345.2252	1	2.254725	120071.1	344.218	tri/oligopeptic	N
432	Egg	345.2505	1	2.441813	122538.1	344.2432	tri/oligopeptic	N
433	Egg	347.1932	1	9.957804	159357.2	346.1859	tri/oligopeptic	N
434	Egg	347.1935	1	6.170188	144576.4	346.1862	tri/oligopeptic	N
435	Egg	347.1936	1	4.457533	472819.4	346.1863	tri/oligopeptic	N
436	Egg	347.2297	1	2.098179	127201.4	346.2224	tri/oligopeptic	N
437	Egg	348.1409	1	2.254725	168049.3	347.1337	tri/oligopeptic	N
438	Egg	348.1772	1	5.889554	218477.7	347.1699	tri/oligopeptic	N
439	Egg	348.1773	1	6.799275	221076.6	347.17	tri/oligopeptic	N
440	Egg	348.1774	1	2.561363	441361.6	347.1701	tri/oligopeptic	N
441	Egg	348.1777	1	3.08725	100367.1	347.1704	tri/oligopeptic	N
442	Egg	352.1515	1	12.65745	133403	351.1442	tri/oligopeptic	N
443	Egg	358.1984	1	15.88562	1020130	357.1911	tri/oligopeptic	N
444	Egg	359.2298	1	9.589308	170369.7	358.2225	tri/oligopeptic	N
445	Egg	359.2301	1	3.050596	147851.1	358.2228	tri/oligopeptic	N
446	Egg	360.3243	1	36.4099	244299.3	359.317	tri/oligopeptic	N
447	Egg	360.3247	1	38.97075	226332.1	359.3175	tri/oligopeptic	N
448	Egg	361.1724	1	5.114638	208020.7	360.1652	tri/oligopeptic	N
449	Egg	361.1726	1	2.441813	261223.3	360.1653	tri/oligopeptic	N
450	Egg	361.1727	1	2.808975	168548.7	360.1654	tri/oligopeptic	N
451	Egg	361.1728	1	10.22242	217711.9	360.1656	tri/oligopeptic	N
452	Egg	361.2357	1	37.11418	154780.4	360.2284	tri/oligopeptic	N
453	Egg	363.1554	1	3.021488	155745.4	362.1481	tri/oligopeptic	N
454	Egg	364.1358	1	2.141692	245839.6	363.1285	tri/oligopeptic	N
455	Egg	366.3739	1	36.71975	137774.2	365.3666	tri/oligopeptic	N

456	Egg	368.3532	1	39.04783	210097.1	367.346	tri/oligopeptic	N
457	Egg	374.204	1	3.430229	144672.5	373.1967	tri/oligopeptic	N
458	Egg	374.2041	1	2.441813	206944.4	373.1969	tri/oligopeptic	N
459	Egg	374.2297	1	14.87214	191768.9	373.2224	tri/oligopeptic	N
460	Egg	376.2244	1	25.40078	264727	375.2171	tri/oligopeptic	N
461	Egg	378.17	1	8.837529	126590.6	377.1627	tri/oligopeptic	N
462	Egg	378.1705	1	2.939775	135346.2	377.1632	tri/oligopeptic	N
463	Egg	380.1828	1	20.58785	250338.7	379.1755	tri/oligopeptic	N
464	Egg	383.4005	1	36.00488	262363.1	382.3932	tri/oligopeptic	N
465	Egg	384.3116	1	37.12183	114065.6	383.3043	tri/oligopeptic	N
466	Egg	390.199	1	2.441813	118680.3	389.1917	tri/oligopeptic	N
467	Egg	391.1623	1	14.86355	176056.9	390.155	tri/oligopeptic	N
468	Egg	391.183	1	5.470129	141462.8	390.1757	tri/oligopeptic	N
469	Egg	391.1836	1	3.220267	138385.8	390.1763	tri/oligopeptic	N
470	Egg	391.2023	1	18.982	186990.6	390.195	tri/oligopeptic	N
471	Egg	391.2194	1	7.799954	109173.6	390.2121	tri/oligopeptic	N
472	Egg	391.2856	1	0.957508	337660.6	390.2783	tri/oligopeptic	N
473	Egg	394.1809	1	23.55302	407135.5	393.1736	tri/oligopeptic	N
474	Egg	398.2337	1	39.47645	145393.2	397.2265	tri/oligopeptic	N
475	Egg	399.225	1	14.86355	153885.2	398.2177	tri/oligopeptic	N
476	Egg	403.2307	1	5.843033	146328.3	402.2234	tri/oligopeptic	N
477	Egg	403.2308	1	2.441813	324037.8	402.2235	tri/oligopeptic	N
478	Egg	405.2145	1	19.02448	286818.3	404.2072	tri/oligopeptic	N
479	Egg	405.2619	1	37.0692	353579.3	404.2546	tri/oligopeptic	N
480	Egg	407.1415	1	2.025771	138737.6	406.1342	tri/oligopeptic	N
481	Egg	407.178	1	2.404792	159043	406.1707	tri/oligopeptic	N
482	Egg	407.2699	1	35.73519	165218.9	406.2626	tri/oligopeptic	N
483	Egg	409.1728	1	12.38957	317617.3	408.1655	tri/oligopeptic	N
484	Egg	413.267	1	37.84227	898769.7	412.2597	tri/oligopeptic	N
485	Egg	413.2676	1	38.24858	513518.7	412.2603	tri/oligopeptic	N
486	Egg	417.2356	1	14.87955	112847	416.2283	tri/oligopeptic	N
487	Egg	419.2149	1	12.58989	125277.2	418.2076	tri/oligopeptic	N
488	Egg	421.213	1	16.38858	115701.4	420.2057	tri/oligopeptic	N
489	Egg	423.236	1	36.00488	112415.3	422.2288	tri/oligopeptic	N
490	Egg	428.2516	1	17.0312	351348.2	427.2443	tri/oligopeptic	N
491	Egg	428.3378	1	37.0692	175869.9	427.3305	tri/oligopeptic	N
492	Egg	429.2416	1	38.30464	123396.8	428.2343	tri/oligopeptic	N
493	Egg	430.2673	1	20.64273	197912.9	429.26	tri/oligopeptic	N
494	Egg	432.2573	1	2.141692	281067.7	431.25	tri/oligopeptic	N
495	Egg	435.1888	1	19.8693	125170.1	434.1815	tri/oligopeptic	N
496	Egg	440.4109	1	39.08271	156417.8	439.4036	tri/oligopeptic	N
497	Egg	441.2981	1	37.15278	153152	440.2909	tri/oligopeptic	N
498	Egg	446.2257	1	15.75597	454547.7	445.2184	tri/oligopeptic	N
499	Egg	446.2614	1	11.90143	217496.7	445.2541	tri/oligopeptic	N
500	Egg	446.262	1	15.35666	629321.4	445.2547	tri/oligopeptic	N
501	Egg	446.2626	1	9.92875	131778.6	445.2553	tri/oligopeptic	N
502	Egg	447.3467	1	36.38005	209128.6	446.3394	tri/oligopeptic	N
503	Egg	449.288	1	37.0323	266715.4	448.2808	tri/oligopeptic	N
504	Egg	451.2662	1	37.15278	264204.5	450.2589	tri/oligopeptic	N
505	Egg	456.2827	1	21.93832	558774.5	455.2755	tri/oligopeptic	N

506	Egg	459.2645	1	20.85927	1013701	458.2573	tri/oligopeptic	N
507	Egg	461.2618	1	20.1633	103922.2	460.2545	tri/oligopeptic	N
508	Egg	465.2182	1	24.48658	230504.1	464.2109	tri/oligopeptic	N
509	Egg	465.2359	1	21.67026	119933.6	464.2286	tri/oligopeptic	N
510	Egg	466.3167	1	33.26533	2330749	465.3095	tri/oligopeptic	N
511	Egg	467.3486	1	34.0478	106236.9	466.3413	tri/oligopeptic	N
512	Egg	468.4418	1	37.48591	262781	467.4346	tri/oligopeptic	N
513	Egg	235.5955	2	15.75597	131240.6	469.1765	tri/oligopeptic	N
514	Egg	471.2829	1	24.6175	472895.6	470.2756	tri/oligopeptic	N
515	Egg	472.364	1	37.0323	115984.4	471.3567	tri/oligopeptic	N
516	Egg	473.2253	1	14.1332	333433	472.218	tri/oligopeptic	N
517	Egg	476.2518	1	19.70328	304537.2	475.2445	tri/oligopeptic	N
518	Egg	477.3666	1	34.98545	124092.4	476.3594	tri/oligopeptic	N
519	Egg	480.2464	1	17.74115	583411.4	479.2391	tri/oligopeptic	N
520	Egg	242.5901	2	15.75597	115155.2	483.1656	tri/oligopeptic	N
521	Egg	484.3142	1	23.72195	489893	483.3069	tri/oligopeptic	N
522	Egg	484.3144	1	20.22308	119378.4	483.3072	tri/oligopeptic	N
523	Egg	491.2944	1	37.0602	113982.6	490.2871	tri/oligopeptic	N
524	Egg	493.3142	1	36.99063	100240.1	492.3069	tri/oligopeptic	N
525	Egg	498.2893	1	33.01051	192722.1	497.2821	tri/oligopeptic	N
526	Egg	250.1884	2	38.98545	110931.3	498.3622	tri/oligopeptic	N
527	Egg	500.305	1	32.42	715467.5	499.2978	tri/oligopeptic	N
528	Egg	251.6661	2	13.40645	220406.2	501.3177	tri/oligopeptic	N
529	Egg	503.2836	1	15.5226	121719	502.2764	tri/oligopeptic	N
530	Egg	504.2673	1	17.87733	992139.1	503.26	tri/oligopeptic	N
531	Egg	252.6427	2	2.404792	121225.8	503.2709	tri/oligopeptic	N
532	Egg	504.2781	1	2.422696	128437	503.2709	tri/oligopeptic	N
533	Egg	505.2262	1	2.351929	130222.3	504.219	tri/oligopeptic	N
534	Egg	505.2674	1	27.08945	100582.4	504.2602	tri/oligopeptic	N
535	Egg	513.3043	1	17.53155	117628.3	512.297	tri/oligopeptic	N
536	Egg	515.3198	1	19.39674	365826.5	514.3125	tri/oligopeptic	N
537	Egg	258.1691	2	7.649746	157780.5	514.3237	tri/oligopeptic	N
538	Egg	516.2676	1	13.98917	566958.9	515.2603	tri/oligopeptic	N
539	Egg	516.3003	1	31.51498	239272.6	515.293	tri/oligopeptic	N
540	Egg	258.6614	2	17.74902	225449.7	515.3083	tri/oligopeptic	N
541	Egg	516.3245	1	36.18276	100996.6	515.3172	tri/oligopeptic	N
542	Egg	517.2629	1	16.59175	108324.6	516.2557	tri/oligopeptic	N
543	Egg	517.2992	1	19.21868	210229.2	516.2919	tri/oligopeptic	N
544	Egg	519.4391	1	37.16177	118129	518.4318	tri/oligopeptic	N
545	Egg	520.2416	1	16.9723	266532.3	519.2343	tri/oligopeptic	N
546	Egg	520.3342	1	21.53453	101054.1	519.3269	tri/oligopeptic	N
547	Egg	521.2572	1	10.66618	128232.9	520.2499	tri/oligopeptic	N
548	Egg	521.2579	1	3.08725	128464.8	520.2506	tri/oligopeptic	N
549	Egg	522.2412	1	7.701329	154630.2	521.2339	tri/oligopeptic	N
550	Egg	522.2415	1	2.516542	118105.2	521.2342	tri/oligopeptic	N
551	Egg	261.6504	2	17.51057	268021.7	521.2862	tri/oligopeptic	N
552	Egg	265.1532	2	11.84195	357926.2	528.2919	tri/oligopeptic	N
553	Egg	265.1535	2	3.059763	348810.4	528.2925	tri/oligopeptic	N
554	Egg	265.6565	2	4.933442	102853.5	529.2984	tri/oligopeptic	N
555	Egg	533.4546	1	37.15278	262247.3	532.4473	tri/oligopeptic	N

556	Egg	536.2397	1	16.91682	184967.9	535.2325	tri/oligopeptic	N
557	Egg	536.2804	1	33.26533	162763.7	535.2731	tri/oligopeptic	N
558	Egg	538.252	1	32.42	142233.1	537.2447	tri/oligopeptic	N
559	Egg	542.302	1	22.05201	133293.9	541.2948	tri/oligopeptic	N
560	Egg	543.3148	1	13.67203	175121.9	542.3076	tri/oligopeptic	N
561	Egg	543.3154	1	13.07962	161642.2	542.3082	tri/oligopeptic	N
562	Egg	544.2992	1	18.76391	127291.6	543.292	tri/oligopeptic	N
563	Egg	273.1488	2	33.26533	139690.4	544.283	tri/oligopeptic	N
564	Egg	547.2016	1	15.37784	326834.9	546.1943	tri/oligopeptic	N
565	Egg	557.2949	1	17.35413	242803.1	556.2877	tri/oligopeptic	N
566	Egg	559.3097	1	2.441813	198202.3	558.3024	tri/oligopeptic	N
567	Egg	280.1586	2	2.459775	656934.5	558.3026	tri/oligopeptic	N
568	Egg	280.1588	2	7.3065	503211.3	558.3031	tri/oligopeptic	N
569	Egg	564.3609	1	22.45108	106341	563.3536	tri/oligopeptic	N
570	Egg	284.1427	2	10.15414	172469.2	566.2709	tri/oligopeptic	N
571	Egg	568.3353	1	22.08695	1481416	567.328	tri/oligopeptic	N
572	Egg	284.6714	2	22.08695	123487.1	567.3283	tri/oligopeptic	N
573	Egg	285.1931	2	18.83526	152499.4	568.3716	tri/oligopeptic	N
574	Egg	285.6733	2	33.26533	125595.3	569.3321	tri/oligopeptic	N
575	Egg	572.2944	1	19.75581	931701.7	571.2871	tri/oligopeptic	N
576	Egg	286.6742	2	7.716142	273353.9	571.3338	tri/oligopeptic	N
577	Egg	286.6743	2	2.441813	335114.1	571.334	tri/oligopeptic	N
578	Egg	575.2683	1	11.82405	127492	574.2611	tri/oligopeptic	N
579	Egg	575.305	1	16.74948	868334.1	574.2977	tri/oligopeptic	N
580	Egg	579.2788	1	15.42431	733518	578.2715	tri/oligopeptic	N
581	Egg	293.6622	2	33.26533	228263	585.3098	tri/oligopeptic	N
582	Egg	293.6877	2	2.992263	119026.3	585.3609	tri/oligopeptic	N
583	Egg	293.6878	2	2.156538	156086.3	585.3611	tri/oligopeptic	N
584	Egg	298.1515	2	2.351929	202505.4	594.2885	tri/oligopeptic	N
585	Egg	298.1516	2	2.071563	364081	594.2886	tri/oligopeptic	N
586	Egg	298.1517	2	3.021488	531673.6	594.2888	tri/oligopeptic	N
587	Egg	300.1352	2	16.70318	143783.7	598.2558	tri/oligopeptic	N
588	Egg	300.1796	2	10.59145	164199.7	598.3447	tri/oligopeptic	N
589	Egg	304.1329	2	2.025771	165420.6	606.2512	tri/oligopeptic	N
590	Egg	307.1878	2	19.32281	910238.4	612.3611	tri/oligopeptic	N
591	Egg	307.6203	2	18.06362	115795	613.2261	oligopeptide	N
592	Egg	312.1745	2	16.87238	215749.2	622.3344	oligopeptide	N
593	Egg	315.1672	2	13.22776	124543	628.3198	oligopeptide	N
594	Egg	632.3371	1	2.369725	140628.1	631.3299	oligopeptide	N
595	Egg	316.6724	2	2.369725	426256.4	631.3302	oligopeptide	N
596	Egg	316.6727	2	3.905454	293071.8	631.3309	oligopeptide	N
597	Egg	316.6749	2	21.7642	281843.5	631.3353	oligopeptide	N
598	Egg	635.309	1	22.49933	367774.7	634.3017	oligopeptide	N
599	Egg	637.3072	1	37.69898	135266.9	636.2999	oligopeptide	N
600	Egg	319.1882	2	17.2146	106366.5	636.3619	oligopeptide	N
601	Egg	645.3447	1	31.4738	705616.3	644.3374	oligopeptide	N
602	Egg	323.1882	2	2.992263	107681.7	644.3618	oligopeptide	N
603	Egg	335.2371	2	13.04052	119501.1	668.4596	oligopeptide	N
604	Egg	675.151	1	36.86903	100302.5	674.1437	oligopeptide	N
605	Egg	338.688	2	22.05201	128615.6	675.3615	oligopeptide	N

606	Egg	339.1855	2	19.3695	1575424	676.3564	oligopeptide	N
607	Egg	343.139	2	17.50146	110792.6	684.2635	oligopeptide	N
608	Egg	685.3791	1	22.69245	228722.3	684.3718	oligopeptide	N
609	Egg	344.1879	2	7.898871	151151.8	686.3612	oligopeptide	N
610	Egg	344.1882	2	2.921871	130016.1	686.3618	oligopeptide	N
611	Egg	344.1882	2	2.459775	113770.7	686.3619	oligopeptide	N
612	Egg	692.2771	1	26.96676	132420.2	691.2698	oligopeptide	N
613	Egg	347.6807	2	14.78566	140540.5	693.3469	oligopeptide	N
614	Egg	697.6587	1	38.9788	154584	696.6514	oligopeptide	N
615	Egg	349.7146	2	14.49884	580888.2	697.4146	oligopeptide	N
616	Egg	350.686	2	14.1332	341851	699.3574	oligopeptide	N
617	Egg	359.6914	2	2.269517	150158.4	717.3683	oligopeptide	N
618	Egg	741.4268	1	20.06393	124249.8	740.4195	oligopeptide	N
619	Egg	371.2173	2	20.06393	4673409	740.4201	oligopeptide	N
620	Egg	377.6669	2	10.68464	255160.9	753.3193	oligopeptide	N
621	Egg	383.1961	2	20.06393	175847.4	764.3777	oligopeptide	N
622	Egg	384.65	2	14.25139	108431.6	767.2854	oligopeptide	N
623	Egg	392.7273	2	26.6692	396587.1	783.44	oligopeptide	N
624	Egg	784.4474	1	26.6692	1044673	783.4401	oligopeptide	N
625	Egg	394.2147	2	14.47958	123025.6	786.4149	oligopeptide	N
626	Egg	797.352	1	19.23475	199749.3	796.3448	oligopeptide	N
627	Egg	404.7063	2	26.6692	131692.2	807.3981	oligopeptide	N
628	Egg	407.7249	2	10.38225	775374.5	813.4353	oligopeptide	N
629	Egg	407.7255	2	2.459775	264847	813.4364	oligopeptide	N
630	Egg	424.2203	2	21.19993	354728.3	846.4261	oligopeptide	N
631	Egg	428.1659	2	13.76779	233381	854.3173	oligopeptide	N
632	Egg	298.8403	3	10.83385	180157.5	893.4991	oligopeptide	N
633	Egg	452.749	2	22.37599	978595.2	903.4835	oligopeptide	N
634	Egg	303.8065	3	2.318379	126301.4	908.3977	oligopeptide	N
635	Egg	460.6668	2	14.75074	178302.9	919.319	oligopeptide	N
636	Egg	466.2413	2	17.52313	798509.8	930.468	oligopeptide	N
637	Egg	931.4755	1	17.52313	267121.1	930.4682	oligopeptide	N
638	Egg	468.2286	2	21.73675	176876.5	934.4427	oligopeptide	N
639	Egg	471.1939	2	14.26156	110055	940.3733	oligopeptide	N
640	Egg	478.2202	2	17.53155	130352.6	954.4259	oligopeptide	N
641	Egg	498.902	2	1.582842	248956.6	995.7894	oligopeptide	N
642	Egg	515.2654	2	15.98212	113812.4	1028.516	oligopeptide	N
643	Egg	521.2914	2	17.62739	115067.1	1040.568	oligopeptide	N
644	Egg	347.8636	3	17.64046	543905	1040.569	oligopeptide	N
645	Egg	524.771	2	26.35684	156793.7	1047.527	oligopeptide	N
646	Egg	548.8698	2	1.630292	124112.6	1095.725	oligopeptide	N
647	Egg	556.7309	2	17.72337	109053.3	1111.447	oligopeptide	N
648	Egg	1126.764	1	1.619417	127620.2	1125.756	oligopeptide	N
649	Egg	587.2569	2	16.61947	129361.5	1172.499	oligopeptide	N
650	Egg	587.2571	2	18.31643	135368.1	1172.5	oligopeptide	N
651	Egg	589.8189	2	21.18589	136695.8	1177.623	oligopeptide	N
652	Egg	608.8164	2	24.66908	378238.8	1215.618	oligopeptide	N
653	Egg	615.8062	2	27.45297	121203.7	1229.598	oligopeptide	N
654	Egg	418.988	3	36.76753	107804.4	1253.942	oligopeptide	N
655	Egg	439.5545	3	21.45928	353139.6	1315.642	oligopeptide	N

656	Egg	663.2923	2	17.4836	115361.2	1324.57	oligopeptide	N
657	Egg	664.7945	2	26.2486	1064544	1327.574	oligopeptide	N
658	Egg	451.5182	3	26.2486	166497.5	1351.533	oligopeptide	N
659	Egg	456.1811	3	26.2486	108505.9	1365.521	oligopeptide	N
660	Egg	686.4669	2	34.08	212931.8	1370.919	oligopeptide	N
661	Egg	502.9077	3	17.1089	141705	1505.701	oligopeptide	N
662	Egg	377.4328	4	17.1089	163403.9	1505.702	oligopeptide	N
663	Egg	552.8916	3	20.29598	127708.9	1655.653	oligopeptide	N
664	Egg	586.5745	3	19.79107	245005	1756.702	oligopeptide	N
665	Egg	1024.782	2	1.607821	130112.8	2047.55	oligopeptide	N
666	Egg	1058.777	2	1.607821	197013.4	2115.54	oligopeptide	N
667	Egg	1092.77	2	1.607821	116870.8	2183.525	oligopeptide	N
668	Egg	1031.87	3	33.14594	1117333	3092.589	oligopeptide	N
669	Egg	779.8988	4	33.13924	132849.4	3115.566	oligopeptide	N
670	Egg	636.5268	5	31.70659	195539.7	3177.598	oligopeptide	N
671	Egg	1069.564	3	33.711	179431.8	3205.67	oligopeptide	N
672	Egg	1069.568	3	34.17494	126781.8	3205.684	oligopeptide	N
673	Egg	872.8746	4	12.65745	125238.4	3487.469	oligopeptide	N
674	Egg	935.2255	4	33.0191	2579990	3736.873	oligopeptide	N
675	Egg	748.3819	5	33.01051	7303922	3736.873	oligopeptide	N
676	Egg	623.8197	6	33.01051	920190.7	3736.874	oligopeptide	N
677	Egg	748.3827	5	40.04883	128365.7	3736.877	oligopeptide	N
678	Egg	752.7764	5	33.0191	312760.2	3758.846	oligopeptide	N
679	Egg	627.4816	6	33.05928	145900.4	3758.846	oligopeptide	N
680	Egg	630.1441	6	33.00094	217420	3774.821	oligopeptide	N
681	Egg	755.9715	5	33.01051	339460.3	3774.821	oligopeptide	N
682	Egg	952.9845	4	33.01051	115642.4	3807.909	oligopeptide	N
683	Egg	762.5901	5	33.04512	570495.8	3807.914	oligopeptide	N
684	Egg	970.7438	4	33.0191	2757822	3878.946	oligopeptide	N
685	Egg	776.7965	5	33.0191	10391855	3878.946	oligopeptide	N
686	Egg	647.4985	6	33.01051	1932310	3878.947	oligopeptide	N
687	Egg	776.7979	5	40.07121	193215.7	3878.953	oligopeptide	N
688	Egg	651.1607	6	33.04512	277217.9	3900.921	oligopeptide	N
689	Egg	781.3915	5	33.03768	415596.7	3901.921	oligopeptide	N
690	Egg	653.823	6	33.01051	379093.2	3916.894	oligopeptide	N
691	Egg	784.388	5	33.16486	485295	3916.904	oligopeptide	N
692	Egg	839.4286	5	34.34394	225926.9	4192.107	oligopeptide	N
693	Egg	1142.905	5	23.49596	575832.8	5709.487	oligopeptide	N
694	Egg	952.5892	6	23.47803	953732.3	5709.491	oligopeptide	N
695	Egg	955.5912	6	24.45528	433373.7	5727.503	oligopeptide	N
696	Egg	1146.509	5	24.53197	132042.5	5727.511	oligopeptide	N
697	Egg	819.3654	7	24.41442	138041.7	5728.507	oligopeptide	N
ID	Protein	mz	charge	retentiont	raw.abund	mass	type	Match
2	Soy	231.1707	1	5.410937	1300555	230.1635	di/tripeptide	Y
3	Soy	231.1708	1	9.712147	732405.9	230.1635	di/tripeptide	Y
4	Soy	231.1708	1	12.0754	841262.9	230.1635	di/tripeptide	Y
5	Soy	233.1497	1	2.19034	362064.6	232.1424	di/tripeptide	Y
6	Soy	233.1498	1	2.804483	211873.9	232.1425	di/tripeptide	Y
8	Soy	239.1028	1	2.453827	112055.1	238.0956	di/tripeptide	Y
11	Soy	245.1862	1	16.43531	1401550	244.1789	di/tripeptide	Y

12	Soy	245.1864	1	15.90536	286577.3	244.1791	di/tripeptide	Y
13	Soy	245.1862	1	14.55055	755014.6	244.1789	di/tripeptide	Y
14	Soy	245.1865	1	17.69164	359748.6	244.1792	di/tripeptide	Y
15	Soy	246.145	1	2.19034	957325.6	245.1377	di/tripeptide	Y
15	Soy	246.145	1	1.96049	122597.2	245.1377	di/tripeptide	Y
16	Soy	246.145	1	2.67671	525933.2	245.1378	di/tripeptide	Y
17	Soy	246.1452	1	3.407643	1168265	245.1379	di/tripeptide	Y
20	Soy	247.1289	1	2.134193	557202.4	246.1217	di/tripeptide	Y
21	Soy	247.1293	1	3.186757	292606.6	246.122	di/tripeptide	Y
22	Soy	247.1293	1	4.693403	376982.4	246.122	di/tripeptide	Y
27	Soy	253.1185	1	2.299983	360570	252.1112	di/tripeptide	Y
27	Soy	253.1186	1	2.510303	109239.3	252.1113	di/tripeptide	Y
28	Soy	253.1188	1	9.330253	189787.8	252.1115	di/tripeptide	Y
30	Soy	260.1607	1	2.19034	853381.7	259.1534	di/tripeptide	Y
31	Soy	260.1609	1	2.68468	336825.5	259.1536	di/tripeptide	Y
33	Soy	260.197	1	1.857653	144687.2	259.1897	di/tripeptide	Y
34	Soy	261.1449	1	2.201593	532283.9	260.1376	di/tripeptide	Y
35	Soy	261.145	1	3.31379	201665.5	260.1377	di/tripeptide	Y
36	Soy	261.145	1	5.432833	513078.5	260.1377	di/tripeptide	Y
38	Soy	263.1429	1	11.53434	346796.2	262.1357	di/tripeptide	Y
40	Soy	265.1552	1	16.28741	234461.4	264.1479	di/tripeptide	Y
41	Soy	267.1343	1	2.41733	161881.6	266.127	di/tripeptide	Y
42	Soy	269.1609	1	2.179287	242856.1	268.1536	di/tripeptide	Y
44	Soy	277.1032	1	1.86743	113426.3	276.0959	di/tripeptide	Y
47	Soy	288.1924	1	16.08951	297451.1	287.1851	di/tripeptide	Y
50	Soy	294.1451	1	2.34546	242460.4	293.1378	di/tripeptide	Y
51	Soy	295.1295	1	3.292053	391894.4	294.1222	di/tripeptide	Y
52	Soy	295.1657	1	16.43531	675524.7	294.1584	di/tripeptide	Y
55	Soy	302.208	1	16.4289	131435.8	301.2007	tri/oligopeptic	Y
56	Soy	302.2081	1	15.16414	549487.3	301.2008	tri/oligopeptic	Y
57	Soy	302.2081	1	19.87241	233654.2	301.2008	tri/oligopeptic	Y
58	Soy	303.1665	1	2.19034	285780.6	302.1593	tri/oligopeptic	Y
59	Soy	304.1507	1	2.22438	358797.1	303.1434	tri/oligopeptic	Y
60	Soy	304.151	1	4.902313	205994	303.1437	tri/oligopeptic	Y
61	Soy	304.1663	1	19.00177	137380	303.159	tri/oligopeptic	Y
63	Soy	318.1665	1	2.769463	204777.1	317.1593	tri/oligopeptic	Y
64	Soy	318.1663	1	2.201593	385805.6	317.1591	tri/oligopeptic	Y
65	Soy	318.1668	1	9.139763	134922.6	317.1596	tri/oligopeptic	Y
66	Soy	318.1821	1	22.57565	271068.8	317.1748	tri/oligopeptic	Y
71	Soy	330.2395	1	18.13061	256233.3	329.2323	tri/oligopeptic	Y
72	Soy	331.1615	1	1.902557	173004.9	330.1542	tri/oligopeptic	Y
73	Soy	331.1657	1	2.86022	299527.6	330.1584	tri/oligopeptic	Y
74	Soy	331.2851	1	38.46329	122344.4	330.2778	tri/oligopeptic	Y
75	Soy	332.1822	1	2.58934	213983.8	331.1749	tri/oligopeptic	Y
80	Soy	334.1613	1	2.334193	326816.8	333.154	tri/oligopeptic	Y
85	Soy	344.2554	1	21.64955	298270.3	343.2481	tri/oligopeptic	Y
85	Soy	344.2554	1	21.3753	293471.4	343.2482	tri/oligopeptic	Y
86	Soy	344.2555	1	20.19351	178863.5	343.2482	tri/oligopeptic	Y
89	Soy	358.2712	1	23.5953	106994.3	357.2639	tri/oligopeptic	Y
90	Soy	359.2293	1	2.22438	520378.8	358.222	tri/oligopeptic	Y

92	Soy	360.2136	1	2.769463	380848.6	359.2063	tri/oligopeptic	Y
94	Soy	363.1555	1	2.19034	131465.9	362.1482	tri/oligopeptic	Y
95	Soy	365.1366	1	37.81734	108596.2	364.1293	tri/oligopeptic	Y
96	Soy	366.2139	1	2.201593	189125.8	365.2066	tri/oligopeptic	Y
105	Soy	390.1876	1	2.356947	144443.4	389.1804	tri/oligopeptic	Y
106	Soy	395.0393	1	1.573627	133555.9	394.032	tri/oligopeptic	Y
107	Soy	399.2608	1	18.79712	937735.7	398.2535	tri/oligopeptic	Y
112	Soy	415.2118	1	36.47641	292091.4	414.2045	tri/oligopeptic	Y
121	Soy	433.1935	1	2.49893	284382.9	432.1862	tri/oligopeptic	Y
133	Soy	461.3005	1	37.39671	107878.8	460.2932	tri/oligopeptic	Y
134	Soy	463.0267	1	1.58502	172385.9	462.0194	tri/oligopeptic	Y
141	Soy	479.3102	1	37.13019	150483.5	478.303	tri/oligopeptic	Y
144	Soy	488.2516	1	19.13773	247176.3	487.2443	tri/oligopeptic	Y
151	Soy	500.2882	1	25.20616	170945.1	499.2809	tri/oligopeptic	Y
156	Soy	515.2914	1	37.09338	102154.3	514.2842	tri/oligopeptic	Y
159	Soy	520.278	1	21.18518	186427.3	519.2707	tri/oligopeptic	Y
161	Soy	264.1747	2	35.30401	105257.5	526.3349	tri/oligopeptic	Y
165	Soy	531.014	1	1.596133	219946	530.0068	tri/oligopeptic	Y
166	Soy	531.3871	1	37.83629	113701.2	530.3798	tri/oligopeptic	Y
174	Soy	274.1676	2	35.93114	112584.8	546.3207	tri/oligopeptic	Y
194	Soy	599.0019	1	1.58502	201379.3	597.9946	tri/oligopeptic	Y
208	Soy	643.3318	1	20.74867	129363.5	642.3245	oligopeptide	Y
218	Soy	666.9893	1	1.596133	192468.4	665.982	oligopeptide	Y
240	Soy	734.9768	1	1.596133	170379.3	733.9696	oligopeptide	Y
258	Soy	802.9643	1	1.58502	145325.8	801.9571	oligopeptide	Y
276	Soy	870.9516	1	1.596133	101006.3	869.9443	oligopeptide	Y
296	Soy	974.814	1	1.60749	254589.3	973.8067	oligopeptide	Y
297	Soy	990.7886	1	1.60749	184364	989.7813	oligopeptide	Y
338	Soy	223.1081	1	5.138677	292885.1	222.1008	dipeptide	N
339	Soy	223.1081	1	10.45349	152375.2	222.1009	dipeptide	N
340	Soy	226.9517	1	1.54202	159697.9	225.9444	di/tripeptide	N
341	Soy	226.9521	1	1.664157	160373.7	225.9448	di/tripeptide	N
342	Soy	229.1552	1	9.020453	201268.8	228.1479	di/tripeptide	N
343	Soy	231.1708	1	8.981633	549444.1	230.1635	di/tripeptide	N
344	Soy	232.1292	1	2.19034	145900	231.122	di/tripeptide	N
345	Soy	233.1133	1	2.19034	146559.5	232.106	di/tripeptide	N
346	Soy	233.15	1	4.093763	381285.4	232.1427	di/tripeptide	N
347	Soy	237.1238	1	4.64821	230371.4	236.1165	di/tripeptide	N
348	Soy	237.1238	1	11.22803	287221.3	236.1165	di/tripeptide	N
349	Soy	246.1453	1	5.35951	483600.5	245.138	di/tripeptide	N
350	Soy	253.1295	1	2.19034	111593	252.1222	di/tripeptide	N
351	Soy	255.0658	1	30.4133	244336.4	254.0586	di/tripeptide	N
352	Soy	258.1103	1	1.73406	146112.3	257.103	di/tripeptide	N
353	Soy	263.1398	1	14.45582	265925	262.1325	di/tripeptide	N
354	Soy	263.143	1	13.13653	136218.1	262.1357	di/tripeptide	N
355	Soy	265.1551	1	14.76493	265680.2	264.1478	di/tripeptide	N
356	Soy	269.1134	1	2.34546	135641.7	268.1062	di/tripeptide	N
357	Soy	271.0602	1	33.8803	167278.8	270.053	di/tripeptide	N
358	Soy	276.1192	1	1.803487	133444.1	275.1119	di/tripeptide	N
359	Soy	276.1349	1	17.19951	217938.5	275.1276	di/tripeptide	N

360	Soy	276.1558	1	2.201593	196380.4	275.1485	di/tripeptide	N
361	Soy	276.156	1	3.383567	187150.3	275.1487	di/tripeptide	N
362	Soy	278.1171	1	2.145577	112890.7	277.1098	di/tripeptide	N
363	Soy	279.1709	1	19.91176	442501.4	278.1637	di/tripeptide	N
364	Soy	279.171	1	18.99481	126435.1	278.1637	di/tripeptide	N
365	Soy	279.1711	1	21.03812	154404.5	278.1638	di/tripeptide	N
366	Soy	279.2321	1	37.01965	588390.2	278.2248	di/tripeptide	N
367	Soy	280.1294	1	2.22438	120614.4	279.1221	di/tripeptide	N
368	Soy	280.1299	1	10.05288	105144.5	279.1226	di/tripeptide	N
369	Soy	281.1137	1	11.59336	397016.3	280.1065	di/tripeptide	N
370	Soy	281.1502	1	6.15241	263167.2	280.143	di/tripeptide	N
371	Soy	286.1768	1	13.45593	456719.6	285.1695	di/tripeptide	N
372	Soy	288.192	1	2.247413	372573.7	287.1847	di/tripeptide	N
373	Soy	288.2032	1	1.99523	129169.2	287.1959	di/tripeptide	N
374	Soy	289.1509	1	2.02972	132674.3	288.1436	di/tripeptide	N
375	Soy	290.1348	1	2.02972	113681.5	289.1275	di/tripeptide	N
376	Soy	290.1716	1	2.201593	577191.9	289.1643	di/tripeptide	N
377	Soy	292.1296	1	16.43531	222800.7	291.1223	di/tripeptide	N
378	Soy	295.1297	1	12.65641	191504	294.1224	di/tripeptide	N
379	Soy	295.1657	1	14.61802	289237.6	294.1585	di/tripeptide	N
380	Soy	295.166	1	13.42547	759601.5	294.1587	di/tripeptide	N
381	Soy	295.2271	1	36.28755	121969.6	294.2198	di/tripeptide	N
382	Soy	295.2272	1	37.38631	317811.2	294.2199	di/tripeptide	N
383	Soy	296.1243	1	2.19034	119462.3	295.1171	di/tripeptide	N
384	Soy	297.1086	1	2.719777	109351.4	296.1013	di/tripeptide	N
385	Soy	297.2427	1	36.15032	180915.9	296.2354	di/tripeptide	N
386	Soy	303.1669	1	5.837807	130791	302.1597	tri/oligopeptic	N
387	Soy	304.133	1	2.66673	102250.4	303.1257	tri/oligopeptic	N
388	Soy	304.1869	1	2.201593	114677.2	303.1796	tri/oligopeptic	N
389	Soy	304.1871	1	2.41733	132742.1	303.1799	tri/oligopeptic	N
390	Soy	306.1457	1	10.7608	546120.8	305.1385	tri/oligopeptic	N
391	Soy	306.1662	1	2.19034	192813.9	305.1589	tri/oligopeptic	N
392	Soy	310.1402	1	2.58934	190464.3	309.1329	tri/oligopeptic	N
393	Soy	311.1243	1	2.77728	121001	310.117	tri/oligopeptic	N
394	Soy	313.2376	1	35.81307	184336.3	312.2303	tri/oligopeptic	N
395	Soy	314.2081	1	13.67871	1045387	313.2008	tri/oligopeptic	N
396	Soy	315.2533	1	36.15032	389994.9	314.246	tri/oligopeptic	N
397	Soy	316.2239	1	14.83729	101454.9	315.2167	tri/oligopeptic	N
398	Soy	316.224	1	19.60118	250725.8	315.2167	tri/oligopeptic	N
399	Soy	317.1823	1	2.201593	299447.4	316.175	tri/oligopeptic	N
400	Soy	317.1826	1	2.48644	723417.7	316.1753	tri/oligopeptic	N
401	Soy	317.2091	1	37.38631	194403.6	316.2018	tri/oligopeptic	N
402	Soy	318.149	1	3.69555	144784.2	317.1417	tri/oligopeptic	N
403	Soy	318.2027	1	2.356947	190941.7	317.1954	tri/oligopeptic	N
404	Soy	318.203	1	14.61802	211426.2	317.1957	tri/oligopeptic	N
405	Soy	318.2032	1	15.91184	133152.6	317.1959	tri/oligopeptic	N
406	Soy	319.141	1	8.144863	194093	318.1337	tri/oligopeptic	N
407	Soy	320.125	1	17.67373	145644.5	319.1177	tri/oligopeptic	N
408	Soy	320.1456	1	2.19034	122527.5	319.1383	tri/oligopeptic	N
409	Soy	327.0519	1	1.61864	168343.6	326.0446	tri/oligopeptic	N

410	Soy	328.224	1	16.87933	146216.7	327.2168	tri/oligopeptic	N
411	Soy	328.2339	1	15.50291	102345.1	327.2266	tri/oligopeptic	N
412	Soy	330.2033	1	13.98454	106322.9	329.196	tri/oligopeptic	N
413	Soy	331.1981	1	2.41733	225651.2	330.1908	tri/oligopeptic	N
414	Soy	331.2484	1	35.42013	105464.4	330.2412	tri/oligopeptic	N
415	Soy	332.2188	1	15.84154	152830.2	331.2115	tri/oligopeptic	N
416	Soy	332.2188	1	18.60684	344494.4	331.2115	tri/oligopeptic	N
417	Soy	333.1771	1	2.212967	262662	332.1699	tri/oligopeptic	N
418	Soy	334.1248	1	1.93733	116368.2	333.1175	tri/oligopeptic	N
419	Soy	334.1406	1	17.97144	313258.7	333.1333	tri/oligopeptic	N
420	Soy	334.1616	1	5.14789	106534	333.1543	tri/oligopeptic	N
421	Soy	334.1771	1	18.03891	107605.3	333.1698	tri/oligopeptic	N
422	Soy	335.2196	1	36.73052	101886.2	334.2123	tri/oligopeptic	N
423	Soy	335.2196	1	36.43542	154959.3	334.2123	tri/oligopeptic	N
424	Soy	336.1386	1	21.04663	177225.4	335.1313	tri/oligopeptic	N
425	Soy	336.1927	1	23.05415	198179.7	335.1854	tri/oligopeptic	N
426	Soy	337.2352	1	36.15032	169785.6	336.2279	tri/oligopeptic	N
427	Soy	340.1508	1	2.311637	114430.8	339.1435	tri/oligopeptic	N
428	Soy	340.3579	1	37.43446	145976.5	339.3506	tri/oligopeptic	N
429	Soy	342.2398	1	19.8871	126855.6	341.2325	tri/oligopeptic	N
430	Soy	343.1982	1	2.41733	136709.1	342.1909	tri/oligopeptic	N
431	Soy	344.1827	1	12.45655	179565.1	343.1754	tri/oligopeptic	N
432	Soy	346.1613	1	2.19034	100429.1	345.154	tri/oligopeptic	N
433	Soy	346.2345	1	14.85206	177085.4	345.2272	tri/oligopeptic	N
434	Soy	346.2346	1	19.19143	156126	345.2274	tri/oligopeptic	N
435	Soy	348.177	1	2.201593	274275.9	347.1697	tri/oligopeptic	N
436	Soy	348.1772	1	2.567123	127681.7	347.1699	tri/oligopeptic	N
437	Soy	350.1721	1	12.71654	150800.9	349.1649	tri/oligopeptic	N
438	Soy	352.1513	1	11.9056	210962.2	351.144	tri/oligopeptic	N
439	Soy	358.1725	1	1.92573	403893	357.1652	tri/oligopeptic	N
440	Soy	358.2955	1	36.28755	151609	357.2882	tri/oligopeptic	N
441	Soy	358.2955	1	36.73052	107532	357.2882	tri/oligopeptic	N
442	Soy	359.2297	1	16.18152	352539	358.2224	tri/oligopeptic	N
443	Soy	359.2298	1	15.84824	309432.8	358.2225	tri/oligopeptic	N
444	Soy	359.2298	1	14.2936	340451.7	358.2225	tri/oligopeptic	N
445	Soy	359.2299	1	19.47349	166400.9	358.2226	tri/oligopeptic	N
446	Soy	360.1929	1	19.62243	119914.8	359.1857	tri/oligopeptic	N
447	Soy	360.214	1	20.62003	278899.7	359.2067	tri/oligopeptic	N
448	Soy	361.1721	1	2.201593	397165.1	360.1648	tri/oligopeptic	N
449	Soy	361.2085	1	2.201593	167876.5	360.2012	tri/oligopeptic	N
450	Soy	361.2353	1	37.32485	100079.1	360.228	tri/oligopeptic	N
451	Soy	362.1564	1	2.567123	116495.4	361.1491	tri/oligopeptic	N
452	Soy	362.1927	1	2.286927	898104.6	361.1854	tri/oligopeptic	N
453	Soy	367.1616	1	2.273767	158443.3	366.1543	tri/oligopeptic	N
454	Soy	374.2036	1	2.19034	297330.1	373.1963	tri/oligopeptic	N
455	Soy	374.2293	1	14.3877	559318.7	373.2221	tri/oligopeptic	N
456	Soy	374.2298	1	21.19854	124354.5	373.2225	tri/oligopeptic	N
457	Soy	377.183	1	17.7734	334161.5	376.1757	tri/oligopeptic	N
458	Soy	383.4	1	36.25822	101096.4	382.3927	tri/oligopeptic	N
459	Soy	387.2243	1	2.334193	150867.7	386.217	tri/oligopeptic	N

460	Soy	387.2246	1	14.80274	113448.3	386.2173	tri/oligopeptic	N
461	Soy	389.2034	1	2.201593	176955.5	388.1961	tri/oligopeptic	N
462	Soy	389.2403	1	13.26386	359099.5	388.2331	tri/oligopeptic	N
463	Soy	391.1986	1	16.68167	400316.2	390.1913	tri/oligopeptic	N
464	Soy	391.2854	1	0.017617	807060.3	390.2781	tri/oligopeptic	N
465	Soy	392.1859	1	11.57734	284448.3	391.1786	tri/oligopeptic	N
466	Soy	392.3138	1	37.0313	111502.9	391.3065	tri/oligopeptic	N
467	Soy	393.2142	1	22.46718	288010.4	392.2069	tri/oligopeptic	N
468	Soy	399.2607	1	14.52741	981027.9	398.2535	tri/oligopeptic	N
469	Soy	401.2405	1	13.81713	165767.6	400.2332	tri/oligopeptic	N
470	Soy	403.2193	1	2.48644	106255.9	402.212	tri/oligopeptic	N
471	Soy	405.1983	1	2.19034	119892.9	404.191	tri/oligopeptic	N
472	Soy	405.2614	1	37.28021	233736.8	404.2542	tri/oligopeptic	N
473	Soy	407.2138	1	2.201593	106349.9	406.2066	tri/oligopeptic	N
474	Soy	413.2665	1	36.81405	392413.7	412.2592	tri/oligopeptic	N
475	Soy	413.2673	1	38.08873	531728.3	412.26	tri/oligopeptic	N
476	Soy	416.2514	1	15.70977	140963.3	415.2441	tri/oligopeptic	N
477	Soy	417.119	1	22.56133	183226.8	416.1117	tri/oligopeptic	N
478	Soy	417.2176	1	19.10422	120585.2	416.2103	tri/oligopeptic	N
479	Soy	423.2356	1	36.2179	102304.5	422.2283	tri/oligopeptic	N
480	Soy	424.183	1	2.322907	229124	423.1757	tri/oligopeptic	N
481	Soy	428.3373	1	37.28021	109656.8	427.3301	tri/oligopeptic	N
482	Soy	429.2415	1	38.08873	130363.9	428.2342	tri/oligopeptic	N
483	Soy	429.3082	1	21.62759	229280.4	428.3009	tri/oligopeptic	N
484	Soy	432.2091	1	2.19034	176859.1	431.2019	tri/oligopeptic	N
485	Soy	433.1139	1	26.54081	317023.3	432.1066	tri/oligopeptic	N
486	Soy	433.1939	1	10.45349	343244.4	432.1866	tri/oligopeptic	N
487	Soy	433.1939	1	11.57734	289947	432.1867	tri/oligopeptic	N
488	Soy	433.2305	1	12.88126	118103	432.2232	tri/oligopeptic	N
489	Soy	434.2251	1	2.567123	141076.8	433.2178	tri/oligopeptic	N
490	Soy	435.2248	1	18.38731	441463	434.2175	tri/oligopeptic	N
491	Soy	441.2982	1	37.37668	191929.6	440.2909	tri/oligopeptic	N
492	Soy	442.2661	1	2.247413	361273	441.2588	tri/oligopeptic	N
493	Soy	445.2403	1	2.19034	175364.7	444.233	tri/oligopeptic	N
494	Soy	446.2248	1	2.19034	117723.3	445.2176	tri/oligopeptic	N
495	Soy	447.246	1	12.42705	204378.3	446.2388	tri/oligopeptic	N
496	Soy	448.2405	1	2.322907	144010.1	447.2332	tri/oligopeptic	N
497	Soy	449.2043	1	17.99405	302649.6	448.197	tri/oligopeptic	N
498	Soy	449.2876	1	37.2412	172281.6	448.2803	tri/oligopeptic	N
499	Soy	450.2358	1	19.51463	137181.5	449.2285	tri/oligopeptic	N
500	Soy	451.2018	1	21.67102	410648.7	450.1946	tri/oligopeptic	N
501	Soy	453.2458	1	2.19034	285198.2	452.2385	tri/oligopeptic	N
502	Soy	456.2826	1	16.64312	342760.5	455.2753	tri/oligopeptic	N
503	Soy	456.2827	1	14.08747	390447.1	455.2754	tri/oligopeptic	N
504	Soy	228.645	2	10.50599	113324.1	455.2755	tri/oligopeptic	N
505	Soy	458.1996	1	2.156933	258021.6	457.1923	tri/oligopeptic	N
506	Soy	458.2613	1	2.393167	204806.7	457.254	tri/oligopeptic	N
507	Soy	460.2776	1	12.85695	263461.1	459.2703	tri/oligopeptic	N
508	Soy	461.2615	1	13.84445	466241.1	460.2542	tri/oligopeptic	N
509	Soy	462.1992	1	15.95769	103097.8	461.1919	tri/oligopeptic	N

510	Soy	462.2197	1	2.201593	157100.1	461.2124	tri/oligopeptic	N
511	Soy	462.2205	1	12.2992	225950.9	461.2132	tri/oligopeptic	N
512	Soy	463.2197	1	16.26486	216110.2	462.2124	tri/oligopeptic	N
513	Soy	463.2198	1	15.12985	141590.3	462.2125	tri/oligopeptic	N
514	Soy	464.3738	1	37.3567	197899.8	463.3665	tri/oligopeptic	N
515	Soy	467.2148	1	18.19269	144028.4	466.2075	tri/oligopeptic	N
516	Soy	468.4415	1	37.6771	119760	467.4342	tri/oligopeptic	N
517	Soy	235.1239	2	2.19034	125070.9	468.2333	tri/oligopeptic	N
518	Soy	472.2774	1	16.0576	126734.6	471.2701	tri/oligopeptic	N
519	Soy	474.2569	1	15.74701	165848.5	473.2496	tri/oligopeptic	N
520	Soy	237.6499	2	2.311637	119506.2	473.2853	tri/oligopeptic	N
521	Soy	476.2353	1	2.19034	106308.7	475.228	tri/oligopeptic	N
522	Soy	240.1528	2	14.76493	739608	478.2911	tri/oligopeptic	N
523	Soy	489.2305	1	2.247413	185123.2	488.2232	tri/oligopeptic	N
524	Soy	490.1976	1	13.43423	184135.9	489.1904	tri/oligopeptic	N
525	Soy	249.1453	2	2.02972	200106.4	496.276	tri/oligopeptic	N
526	Soy	250.1534	2	2.659453	123786.6	498.2921	tri/oligopeptic	N
527	Soy	250.6456	2	10.72989	186060.5	499.2766	tri/oligopeptic	N
528	Soy	500.3083	1	18.87005	2235992	499.3011	tri/oligopeptic	N
529	Soy	501.2306	1	2.156933	164785.3	500.2233	tri/oligopeptic	N
530	Soy	503.1197	1	27.02633	129593.9	502.1124	tri/oligopeptic	N
531	Soy	504.2302	1	2.212967	109880.6	503.2229	tri/oligopeptic	N
532	Soy	504.2669	1	14.52741	116079.8	503.2596	tri/oligopeptic	N
533	Soy	504.2671	1	16.66691	162236.9	503.2598	tri/oligopeptic	N
534	Soy	504.2824	1	23.7609	766611.4	503.2751	tri/oligopeptic	N
535	Soy	510.2566	1	17.70748	147006.4	509.2493	tri/oligopeptic	N
536	Soy	512.3447	1	26.08693	1336486	511.3374	tri/oligopeptic	N
537	Soy	257.1451	2	16.68167	166009.7	512.2757	tri/oligopeptic	N
538	Soy	516.2674	1	13.32147	218364	515.2601	tri/oligopeptic	N
539	Soy	258.6507	2	14.77512	132502.6	515.2868	tri/oligopeptic	N
540	Soy	518.2465	1	14.77512	636732.5	517.2392	tri/oligopeptic	N
541	Soy	519.1146	1	29.53444	322883.2	518.1073	tri/oligopeptic	N
542	Soy	531.2784	1	12.59817	350125.9	530.2711	tri/oligopeptic	N
543	Soy	533.2204	1	2.26061	132444.3	532.2131	tri/oligopeptic	N
544	Soy	538.2518	1	14.27157	160234.2	537.2445	tri/oligopeptic	N
545	Soy	544.236	1	2.19034	128038.5	543.2287	tri/oligopeptic	N
546	Soy	546.3298	1	25.88982	238637.3	545.3225	tri/oligopeptic	N
547	Soy	549.268	1	17.97144	283095.8	548.2607	tri/oligopeptic	N
548	Soy	275.6716	2	18.93769	182808.9	549.3287	tri/oligopeptic	N
549	Soy	278.1793	2	19.2791	141690.6	554.3441	tri/oligopeptic	N
550	Soy	555.3515	1	19.2791	229247.9	554.3442	tri/oligopeptic	N
551	Soy	558.2783	1	15.92095	127781.5	557.271	tri/oligopeptic	N
552	Soy	560.2681	1	2.356947	160404.4	559.2608	tri/oligopeptic	N
553	Soy	563.2108	1	16.50597	132138.5	562.2035	tri/oligopeptic	N
554	Soy	566.2471	1	16.11219	212987	565.2398	tri/oligopeptic	N
555	Soy	569.3675	1	20.84264	116682.5	568.3602	tri/oligopeptic	N
556	Soy	575.2635	1	30.02411	142309	574.2562	tri/oligopeptic	N
557	Soy	289.6873	2	23.36966	224559.8	577.36	tri/oligopeptic	N
558	Soy	292.6562	2	13.76391	115389.9	583.2979	tri/oligopeptic	N
559	Soy	293.6635	2	2.179287	147953.9	585.3124	tri/oligopeptic	N

560	Soy	294.1617	2	12.047	125124.6	586.3088	tri/oligopeptic	N
561	Soy	588.2892	1	13.99992	127536.8	587.2819	tri/oligopeptic	N
562	Soy	295.1511	2	2.632187	338823	588.2877	tri/oligopeptic	N
563	Soy	591.2624	1	2.19034	170070.5	590.2552	tri/oligopeptic	N
564	Soy	591.2636	1	11.15505	159559.8	590.2563	tri/oligopeptic	N
565	Soy	594.2786	1	17.15513	114437.6	593.2713	tri/oligopeptic	N
566	Soy	605.2689	1	18.87751	183191.5	604.2616	tri/oligopeptic	N
567	Soy	607.274	1	18.06415	116464.8	606.2667	tri/oligopeptic	N
568	Soy	611.2325	1	14.11505	120093.8	610.2252	tri/oligopeptic	N
569	Soy	307.1771	2	19.22292	658131.1	612.3397	tri/oligopeptic	N
570	Soy	619.2948	1	15.82979	154375.6	618.2876	oligopeptide	N
571	Soy	310.1643	2	15.70977	173655.7	618.3141	oligopeptide	N
572	Soy	626.284	1	20.48573	126675.3	625.2768	oligopeptide	N
573	Soy	315.6954	2	2.659453	658334	629.3762	oligopeptide	N
574	Soy	632.2902	1	15.15773	207929.8	631.2829	oligopeptide	N
575	Soy	644.363	1	17.35972	112491.3	643.3558	oligopeptide	N
576	Soy	322.6852	2	17.35111	152611.8	643.3559	oligopeptide	N
577	Soy	323.1723	2	15.95175	181219.8	644.33	oligopeptide	N
578	Soy	646.2982	1	2.19034	119220.6	645.2909	oligopeptide	N
579	Soy	323.6529	2	2.19034	342555.7	645.2913	oligopeptide	N
580	Soy	325.1459	2	15.97963	187684.6	648.2772	oligopeptide	N
581	Soy	328.6777	2	2.006527	123850.5	655.3409	oligopeptide	N
582	Soy	329.1815	2	2.212967	256298.4	656.3484	oligopeptide	N
583	Soy	672.3212	1	16.68854	481535.7	671.3139	oligopeptide	N
584	Soy	336.6644	2	16.68854	122183.8	671.3142	oligopeptide	N
585	Soy	672.3327	1	15.74701	102210	671.3254	oligopeptide	N
586	Soy	678.4204	1	23.16424	767609.8	677.4132	oligopeptide	N
587	Soy	339.714	2	23.17124	869239.5	677.4134	oligopeptide	N
588	Soy	679.332	1	24.3967	190237.6	678.3248	oligopeptide	N
589	Soy	682.3788	1	16.85962	204501.4	681.3715	oligopeptide	N
590	Soy	341.6932	2	16.85121	255372.7	681.3719	oligopeptide	N
591	Soy	348.6434	2	16.68854	168099.8	695.2722	oligopeptide	N
592	Soy	704.3481	1	18.20143	118567.9	703.3409	oligopeptide	N
593	Soy	356.1569	2	2.099537	157231.6	710.2992	oligopeptide	N
594	Soy	357.2199	2	16.84442	114286.2	712.4252	oligopeptide	N
595	Soy	358.6955	2	2.212967	141567.5	715.3764	oligopeptide	N
596	Soy	362.7218	2	2.273767	164424.6	723.429	oligopeptide	N
597	Soy	365.2172	2	16.6275	206718.9	728.4199	oligopeptide	N
598	Soy	365.6779	2	2.12261	137861.5	729.3413	oligopeptide	N
599	Soy	731.3222	1	13.28589	118213.4	730.3149	oligopeptide	N
600	Soy	368.686	2	19.13773	377577.8	735.3574	oligopeptide	N
601	Soy	371.2067	2	17.236	220506.3	740.3989	oligopeptide	N
602	Soy	769.4106	1	16.14967	612208.9	768.4034	oligopeptide	N
603	Soy	385.209	2	16.13606	758456	768.4035	oligopeptide	N
604	Soy	386.2037	2	2.168163	195971.6	770.3929	oligopeptide	N
605	Soy	788.344	1	13.742	127096.5	787.3367	oligopeptide	N
606	Soy	396.2254	2	20.99711	108812.2	790.4363	oligopeptide	N
607	Soy	397.1884	2	16.14281	112376.4	792.3622	oligopeptide	N
608	Soy	398.1863	2	13.02295	479441.3	794.358	oligopeptide	N
609	Soy	402.7173	2	14.01437	213871.5	803.42	oligopeptide	N

610	Soy	403.6898	2	2.356947	318453.5	805.365	oligopeptide	N
611	Soy	409.1783	2	16.60027	117503.2	816.342	oligopeptide	N
612	Soy	415.741	2	16.7314	253593.5	829.4674	oligopeptide	N
613	Soy	419.7332	2	19.34892	258548.6	837.4518	oligopeptide	N
614	Soy	283.158	3	17.1861	131216.6	846.452	oligopeptide	N
615	Soy	427.723	2	15.7727	135753	853.4315	oligopeptide	N
616	Soy	429.2385	2	27.05431	170344.6	856.4625	oligopeptide	N
617	Soy	430.2181	2	13.69893	186728.4	858.4216	oligopeptide	N
618	Soy	435.2176	2	16.49981	271066.6	868.4207	oligopeptide	N
619	Soy	449.1968	2	14.52741	260827.5	896.3791	oligopeptide	N
620	Soy	913.5166	1	35.22043	329495.8	912.5093	oligopeptide	N
621	Soy	460.6665	2	14.27896	199116.5	919.3185	oligopeptide	N
622	Soy	943.5273	1	34.96775	1035471	942.5201	oligopeptide	N
623	Soy	476.2074	2	17.26986	238983.3	950.4002	oligopeptide	N
624	Soy	478.7365	2	26.97019	118655.1	955.4584	oligopeptide	N
625	Soy	959.5215	1	34.81903	110070.8	958.5143	oligopeptide	N
626	Soy	487.2237	2	20.67789	171989	972.4328	oligopeptide	N
627	Soy	493.2648	2	17.72728	118773.4	984.515	oligopeptide	N
628	Soy	493.268	2	27.34778	148044.3	984.5214	oligopeptide	N
629	Soy	332.1811	3	23.50908	381719.1	993.5215	oligopeptide	N
630	Soy	332.1917	3	16.21439	284855.6	993.5533	oligopeptide	N
631	Soy	501.7313	2	17.51042	172569.1	1001.448	oligopeptide	N
632	Soy	503.7652	2	34.96118	123913.1	1005.516	oligopeptide	N
633	Soy	511.754	2	34.96118	265755	1021.493	oligopeptide	N
634	Soy	513.7778	2	16.12065	302429.8	1025.541	oligopeptide	N
635	Soy	554.7502	2	16.26486	133913.6	1107.486	oligopeptide	N
636	Soy	557.2945	2	17.33522	614842.3	1112.574	oligopeptide	N
637	Soy	374.8675	3	24.06618	449052.1	1121.581	oligopeptide	N
638	Soy	590.2686	2	16.41307	176978.4	1178.523	oligopeptide	N
639	Soy	417.5537	3	23.77648	105552	1249.639	oligopeptide	N
640	Soy	646.811	2	18.76811	141789.4	1291.608	oligopeptide	N
641	Soy	678.8014	2	17.59616	119280.3	1355.588	oligopeptide	N
642	Soy	580.9133	3	19.10422	115189	1739.718	oligopeptide	N
ID	Protein	mz	charge	retentiont	raw.abund	mass	type	Match
2	NPP	231.1708	1	5.5224	925814.9	230.1635	di/tripeptide	Y
3	NPP	231.1708	1	9.348325	500512	230.1635	di/tripeptide	Y
4	NPP	231.1709	1	12.40158	397429.9	230.1636	di/tripeptide	Y
5	NPP	233.1497	1	2.214517	342083.5	232.1424	di/tripeptide	Y
6	NPP	233.1498	1	2.789542	409783.1	232.1426	di/tripeptide	Y
11	NPP	245.1863	1	16.43528	1478909	244.179	di/tripeptide	Y
12	NPP	245.1865	1	15.90576	252386	244.1792	di/tripeptide	Y
13	NPP	245.1864	1	14.57457	348229.1	244.1791	di/tripeptide	Y
14	NPP	245.1866	1	17.68673	232935.9	244.1794	di/tripeptide	Y
15	NPP	246.145	1	1.945083	176859.4	245.1377	di/tripeptide	Y
15	NPP	246.145	1	2.159183	475758.8	245.1377	di/tripeptide	Y
16	NPP	246.145	1	2.656683	666324.4	245.1377	di/tripeptide	Y
17	NPP	246.1453	1	3.326642	711777.3	245.138	di/tripeptide	Y
20	NPP	247.129	1	2.159183	652274.2	246.1217	di/tripeptide	Y
21	NPP	247.1294	1	3.123558	276567	246.1221	di/tripeptide	Y
22	NPP	247.1292	1	4.7321	645292.4	246.1219	di/tripeptide	Y

24	NPP	250.1781	1	34.85937	735968.6	249.1708	di/tripeptide	Y
25	NPP	250.1783	1	37.29403	156985.2	249.171	di/tripeptide	Y
27	NPP	253.1185	1	2.319408	591383	252.1112	di/tripeptide	Y
27	NPP	253.1186	1	2.4988	110173.7	252.1113	di/tripeptide	Y
30	NPP	260.1607	1	2.214517	657745.7	259.1534	di/tripeptide	Y
31	NPP	260.1609	1	2.676583	201798.1	259.1537	di/tripeptide	Y
32	NPP	260.1611	1	3.6485	176622.2	259.1538	di/tripeptide	Y
33	NPP	260.197	1	1.821858	131385.5	259.1898	di/tripeptide	Y
34	NPP	261.1447	1	2.214517	718592.4	260.1375	di/tripeptide	Y
35	NPP	261.1451	1	3.33345	295048.8	260.1378	di/tripeptide	Y
36	NPP	261.145	1	5.47075	422415.2	260.1377	di/tripeptide	Y
40	NPP	265.1554	1	16.28978	138622.4	264.1481	di/tripeptide	Y
41	NPP	267.1343	1	2.413883	158065.2	266.127	di/tripeptide	Y
42	NPP	269.161	1	2.214517	244440.3	268.1537	di/tripeptide	Y
44	NPP	277.1032	1	1.851992	170909.3	276.0959	di/tripeptide	Y
45	NPP	281.1135	1	2.580675	389904.8	280.1062	di/tripeptide	Y
48	NPP	290.1715	1	2.275517	206850.2	289.1642	di/tripeptide	Y
50	NPP	294.1451	1	2.352533	234989.9	293.1378	di/tripeptide	Y
51	NPP	295.1295	1	3.090375	297887.4	294.1222	di/tripeptide	Y
51	NPP	295.1296	1	3.567942	380811.9	294.1223	di/tripeptide	Y
52	NPP	295.1657	1	16.43528	342454.8	294.1585	di/tripeptide	Y
53	NPP	300.1924	1	2.643583	120403.8	299.1851	di/tripeptide	Y
56	NPP	302.2082	1	15.176	486975.9	301.2009	tri/oligopeptic	Y
57	NPP	302.2081	1	19.87011	202786.1	301.2009	tri/oligopeptic	Y
58	NPP	303.1666	1	2.214517	495528.5	302.1593	tri/oligopeptic	Y
59	NPP	304.1506	1	2.203558	172431	303.1433	tri/oligopeptic	Y
63	NPP	318.1666	1	2.724817	179914.7	317.1593	tri/oligopeptic	Y
64	NPP	318.1664	1	2.233433	258174.5	317.1591	tri/oligopeptic	Y
65	NPP	318.1669	1	9.503458	127439.5	317.1596	tri/oligopeptic	Y
71	NPP	330.2396	1	18.12698	279709.7	329.2323	tri/oligopeptic	Y
73	NPP	331.1657	1	2.847225	234291.8	330.1585	tri/oligopeptic	Y
75	NPP	332.1822	1	2.610933	131685	331.1749	tri/oligopeptic	Y
76	NPP	332.2185	1	3.004592	380566.4	331.2113	tri/oligopeptic	Y
78	NPP	333.1408	1	1.933725	163254.5	332.1335	tri/oligopeptic	Y
80	NPP	334.1612	1	2.214517	224821.6	333.154	tri/oligopeptic	Y
85	NPP	344.2556	1	21.55005	181303.4	343.2483	tri/oligopeptic	Y
89	NPP	358.2714	1	24.01751	125004.9	357.2641	tri/oligopeptic	Y
94	NPP	363.1553	1	2.159183	109651.7	362.1481	tri/oligopeptic	Y
96	NPP	366.214	1	2.214517	114078.2	365.2067	tri/oligopeptic	Y
105	NPP	390.1877	1	2.341342	162132.8	389.1804	tri/oligopeptic	Y
106	NPP	395.0394	1	1.581992	157303.3	394.0321	tri/oligopeptic	Y
107	NPP	399.2611	1	18.79762	534116.3	398.2539	tri/oligopeptic	Y
110	NPP	405.162	1	2.000675	116935.5	404.1547	tri/oligopeptic	Y
112	NPP	415.2119	1	36.47743	267874.9	414.2047	tri/oligopeptic	Y
133	NPP	461.3006	1	37.42824	103192.1	460.2933	tri/oligopeptic	Y
134	NPP	463.0268	1	1.592733	204366.9	462.0195	tri/oligopeptic	Y
141	NPP	479.3104	1	37.26442	118683.5	478.3032	tri/oligopeptic	Y
144	NPP	488.2517	1	19.12801	127110	487.2444	tri/oligopeptic	Y
151	NPP	500.2883	1	25.20508	106250.9	499.281	tri/oligopeptic	Y
159	NPP	520.2781	1	21.19672	103535.8	519.2708	tri/oligopeptic	Y

161	NPP	264.1748	2	35.27954	110477.4	526.3351	tri/oligopeptic	Y
194	NPP	599.002	1	1.592733	236659.7	597.9947	tri/oligopeptic	Y
218	NPP	666.9894	1	1.581992	224109	665.9821	oligopeptide	Y
240	NPP	734.9769	1	1.581992	198281.4	733.9697	oligopeptide	Y
258	NPP	802.9644	1	1.592733	172265.1	801.9571	oligopeptide	Y
276	NPP	870.9519	1	1.581992	119744	869.9446	oligopeptide	Y
296	NPP	974.8142	1	1.613858	248703	973.8069	oligopeptide	Y
302	NPP	1042.802	1	1.613858	136091	1041.794	oligopeptide	Y
316	NPP	616.9397	2	1.592733	101600.1	1231.865	oligopeptide	Y
321	NPP	650.9331	2	1.581992	102766.2	1299.852	oligopeptide	Y
338	NPP	223.1081	1	4.822233	573005.9	222.1008	dipeptide	N
339	NPP	226.9522	1	1.656242	134215.1	225.9449	di/tripeptide	N
340	NPP	229.1552	1	5.048558	180415.7	228.1479	di/tripeptide	N
341	NPP	229.1552	1	8.661092	150759.9	228.1479	di/tripeptide	N
342	NPP	229.1552	1	9.457333	123757.8	228.148	di/tripeptide	N
343	NPP	229.1553	1	11.41065	170061.5	228.148	di/tripeptide	N
344	NPP	231.1708	1	8.661092	170487.8	230.1635	di/tripeptide	N
345	NPP	231.1709	1	10.10248	416923.4	230.1636	di/tripeptide	N
346	NPP	231.1709	1	11.44895	261051.2	230.1636	di/tripeptide	N
347	NPP	233.1133	1	2.012142	143444.7	232.1061	di/tripeptide	N
348	NPP	233.1501	1	4.025867	158470.9	232.1428	di/tripeptide	N
349	NPP	237.1238	1	4.778367	110194.2	236.1165	di/tripeptide	N
350	NPP	237.1238	1	11.74028	116487.9	236.1165	di/tripeptide	N
351	NPP	246.1453	1	5.393658	183508.7	245.1381	di/tripeptide	N
352	NPP	263.1396	1	14.49822	237083.7	262.1323	di/tripeptide	N
353	NPP	265.1552	1	14.78038	283113.3	264.1479	di/tripeptide	N
354	NPP	276.1557	1	2.214517	247855	275.1484	di/tripeptide	N
355	NPP	279.171	1	18.99064	155352.1	278.1637	di/tripeptide	N
356	NPP	279.1711	1	19.90661	1355695	278.1638	di/tripeptide	N
357	NPP	279.1711	1	21.03887	110532.2	278.1639	di/tripeptide	N
358	NPP	279.2322	1	37.01387	205207.9	278.2249	di/tripeptide	N
359	NPP	281.1139	1	10.9666	146937.9	280.1066	di/tripeptide	N
360	NPP	281.1139	1	11.97882	161146.8	280.1066	di/tripeptide	N
361	NPP	281.1503	1	5.7536	342297.6	280.143	di/tripeptide	N
362	NPP	286.1769	1	13.56565	368459	285.1696	di/tripeptide	N
363	NPP	288.1921	1	2.24205	176637.4	287.1848	di/tripeptide	N
364	NPP	288.1923	1	14.68328	186485.6	287.185	di/tripeptide	N
365	NPP	288.2032	1	2.012142	100636.3	287.1959	di/tripeptide	N
366	NPP	295.1659	1	14.63796	158823.8	294.1586	di/tripeptide	N
367	NPP	295.2273	1	37.37973	145816.2	294.22	di/tripeptide	N
368	NPP	296.1244	1	2.203558	121325.8	295.1171	di/tripeptide	N
369	NPP	297.2429	1	36.14133	154761.1	296.2356	di/tripeptide	N
370	NPP	304.187	1	2.258733	139478.5	303.1797	tri/oligopeptic	N
371	NPP	304.1872	1	2.413883	139444.4	303.1799	tri/oligopeptic	N
372	NPP	306.1299	1	1.871108	127969.8	305.1226	tri/oligopeptic	N
373	NPP	306.1455	1	11.3374	184357.3	305.1383	tri/oligopeptic	N
374	NPP	306.1458	1	10.27468	140783.2	305.1385	tri/oligopeptic	N
375	NPP	310.1402	1	2.558808	157761.6	309.1329	tri/oligopeptic	N
376	NPP	311.124	1	2.203558	137512.8	310.1167	tri/oligopeptic	N
377	NPP	311.1243	1	2.73465	119447.1	310.117	tri/oligopeptic	N

378	NPP	313.2377	1	35.81377	240179.3	312.2304	tri/oligopeptic	N
379	NPP	315.2534	1	36.14133	388041	314.2461	tri/oligopeptic	N
380	NPP	316.1874	1	2.676583	114408	315.1801	tri/oligopeptic	N
381	NPP	316.2241	1	19.59533	367978.4	315.2168	tri/oligopeptic	N
382	NPP	317.1823	1	2.214517	279079.5	316.175	tri/oligopeptic	N
383	NPP	318.1669	1	3.655775	150139	317.1596	tri/oligopeptic	N
384	NPP	318.2027	1	2.233433	175418.3	317.1954	tri/oligopeptic	N
385	NPP	320.1456	1	2.159183	109366.4	319.1383	tri/oligopeptic	N
386	NPP	320.1616	1	15.75373	122174.9	319.1544	tri/oligopeptic	N
387	NPP	328.2241	1	18.06699	156967.8	327.2168	tri/oligopeptic	N
388	NPP	328.2241	1	16.89194	193542.4	327.2169	tri/oligopeptic	N
389	NPP	330.2395	1	17.42544	409874.7	329.2322	tri/oligopeptic	N
390	NPP	331.1984	1	3.521267	255888.6	330.1911	tri/oligopeptic	N
391	NPP	331.1985	1	4.31605	240899.8	330.1912	tri/oligopeptic	N
392	NPP	331.2485	1	35.41337	125834	330.2413	tri/oligopeptic	N
393	NPP	332.1454	1	2.203558	129576.8	331.1381	tri/oligopeptic	N
394	NPP	332.2188	1	19.44977	169414.7	331.2115	tri/oligopeptic	N
395	NPP	332.2188	1	4.1195	287292.4	331.2115	tri/oligopeptic	N
396	NPP	332.219	1	18.60003	140655.3	331.2118	tri/oligopeptic	N
397	NPP	333.1772	1	2.24205	260765	332.1699	tri/oligopeptic	N
398	NPP	334.1248	1	1.989408	108680.9	333.1176	tri/oligopeptic	N
399	NPP	334.1408	1	17.96907	117929.4	333.1335	tri/oligopeptic	N
400	NPP	334.1617	1	5.219917	284525.8	333.1544	tri/oligopeptic	N
401	NPP	337.2354	1	36.14133	141996.7	336.2281	tri/oligopeptic	N
402	NPP	338.1355	1	12.75843	164656.8	337.1282	tri/oligopeptic	N
403	NPP	338.1356	1	12.10909	134556.1	337.1283	tri/oligopeptic	N
404	NPP	342.2399	1	21.27832	185948.1	341.2326	tri/oligopeptic	N
405	NPP	343.1983	1	2.404158	390137.4	342.191	tri/oligopeptic	N
406	NPP	343.1986	1	11.59091	161705.7	342.1913	tri/oligopeptic	N
407	NPP	343.1987	1	10.6629	150370.6	342.1914	tri/oligopeptic	N
408	NPP	345.1773	1	2.159183	125277.4	344.1701	tri/oligopeptic	N
409	NPP	345.2138	1	2.214517	136423.2	344.2065	tri/oligopeptic	N
410	NPP	345.2141	1	14.79059	430468.8	344.2068	tri/oligopeptic	N
411	NPP	345.2142	1	5.338983	102076.7	344.2069	tri/oligopeptic	N
412	NPP	346.1614	1	2.192475	141596	345.1541	tri/oligopeptic	N
413	NPP	346.1979	1	2.48815	256947.1	345.1907	tri/oligopeptic	N
414	NPP	346.1983	1	4.309633	302834.1	345.191	tri/oligopeptic	N
415	NPP	348.177	1	2.233433	358886.9	347.1697	tri/oligopeptic	N
416	NPP	348.1775	1	8.07045	290161.4	347.1702	tri/oligopeptic	N
417	NPP	351.0962	1	30.84738	148087.3	350.0889	tri/oligopeptic	N
418	NPP	357.2139	1	2.580675	593755.4	356.2067	tri/oligopeptic	N
419	NPP	358.2957	1	35.81377	112096.8	357.2885	tri/oligopeptic	N
420	NPP	359.2298	1	19.46658	126842.1	358.2225	tri/oligopeptic	N
421	NPP	359.2298	1	15.846	423796.8	358.2225	tri/oligopeptic	N
422	NPP	359.23	1	17.04905	213773.4	358.2227	tri/oligopeptic	N
423	NPP	360.2139	1	19.86357	180714.5	359.2066	tri/oligopeptic	N
424	NPP	360.214	1	8.901842	138842.7	359.2067	tri/oligopeptic	N
425	NPP	360.214	1	20.61621	395895.7	359.2067	tri/oligopeptic	N
426	NPP	360.2141	1	21.2568	144794.6	359.2068	tri/oligopeptic	N
427	NPP	361.1722	1	2.192475	309366	360.1649	tri/oligopeptic	N

428	NPP	362.1564	1	2.558808	155972.1	361.1491	tri/oligopeptic	N
429	NPP	362.1926	1	2.267058	315772.7	361.1854	tri/oligopeptic	N
430	NPP	364.1355	1	1.945083	107756.7	363.1283	tri/oligopeptic	N
431	NPP	367.0665	1	33.76821	105714.3	366.0592	tri/oligopeptic	N
432	NPP	373.2088	1	2.546567	120962.2	372.2016	tri/oligopeptic	N
433	NPP	374.2034	1	2.214517	117959.6	373.1961	tri/oligopeptic	N
434	NPP	374.2296	1	14.40951	188521.4	373.2223	tri/oligopeptic	N
435	NPP	377.1831	1	18.24088	152523.4	376.1758	tri/oligopeptic	N
436	NPP	385.2454	1	2.789542	139191.5	384.2381	tri/oligopeptic	N
437	NPP	390.1986	1	2.181333	136877.2	389.1914	tri/oligopeptic	N
438	NPP	391.199	1	16.65275	146071	390.1917	tri/oligopeptic	N
439	NPP	391.2855	1	0.018708	835320.4	390.2782	tri/oligopeptic	N
440	NPP	397.1728	1	8.315958	152357.9	396.1655	tri/oligopeptic	N
441	NPP	399.2609	1	14.51828	940991.5	398.2536	tri/oligopeptic	N
442	NPP	399.2611	1	14.92188	451703.6	398.2538	tri/oligopeptic	N
443	NPP	405.1984	1	2.203558	151710.8	404.1911	tri/oligopeptic	N
444	NPP	405.2615	1	37.28352	190199.4	404.2542	tri/oligopeptic	N
445	NPP	407.1937	1	18.20436	100391	406.1864	tri/oligopeptic	N
446	NPP	408.2137	1	17.57364	639059.9	407.2064	tri/oligopeptic	N
447	NPP	409.2197	1	2.214517	295795.8	408.2124	tri/oligopeptic	N
448	NPP	413.2669	1	36.77545	1086933	412.2596	tri/oligopeptic	N
449	NPP	415.2355	1	25.41622	121823.8	414.2282	tri/oligopeptic	N
450	NPP	416.2514	1	14.95294	163368.5	415.2442	tri/oligopeptic	N
451	NPP	417.2352	1	14.64766	203759.6	416.2279	tri/oligopeptic	N
452	NPP	418.1935	1	2.192475	193121.8	417.1863	tri/oligopeptic	N
453	NPP	422.2044	1	9.223883	100012.8	421.1972	tri/oligopeptic	N
454	NPP	427.2925	1	20.13953	368281.7	426.2853	tri/oligopeptic	N
455	NPP	428.3374	1	37.28352	104845.1	427.3301	tri/oligopeptic	N
456	NPP	430.2669	1	3.068025	156809.3	429.2596	tri/oligopeptic	N
457	NPP	430.267	1	5.219917	100515.8	429.2598	tri/oligopeptic	N
458	NPP	431.1888	1	2.181333	111780.4	430.1815	tri/oligopeptic	N
459	NPP	432.2094	1	2.446667	151089.9	431.2021	tri/oligopeptic	N
460	NPP	432.2464	1	9.907817	324841.3	431.2392	tri/oligopeptic	N
461	NPP	436.3434	1	0.018708	298578.4	435.3362	tri/oligopeptic	N
462	NPP	447.2097	1	9.354242	218565.6	446.2024	tri/oligopeptic	N
463	NPP	449.2877	1	37.24133	131601.7	448.2804	tri/oligopeptic	N
464	NPP	450.2356	1	19.50183	311694.9	449.2283	tri/oligopeptic	N
465	NPP	456.283	1	16.62268	120075.5	455.2757	tri/oligopeptic	N
466	NPP	456.2911	1	37.29403	264485.2	455.2839	tri/oligopeptic	N
467	NPP	457.2418	1	15.01802	184433.8	456.2345	tri/oligopeptic	N
468	NPP	458.262	1	13.15438	490052.3	457.2548	tri/oligopeptic	N
469	NPP	459.2454	1	2.6187	165151.8	458.2382	tri/oligopeptic	N
470	NPP	462.2198	1	2.24205	152674.6	461.2125	tri/oligopeptic	N
471	NPP	467.1784	1	13.2788	112598.8	466.1712	tri/oligopeptic	N
472	NPP	470.2622	1	13.70957	232321.3	469.2549	tri/oligopeptic	N
473	NPP	475.215	1	2.203558	100646.1	474.2078	tri/oligopeptic	N
474	NPP	240.1529	2	14.76152	222281.9	478.2913	tri/oligopeptic	N
475	NPP	483.2564	1	2.214517	163196.4	482.2492	tri/oligopeptic	N
476	NPP	487.2523	1	10.66908	359080	486.245	tri/oligopeptic	N
477	NPP	488.235	1	2.297392	194798.4	487.2277	tri/oligopeptic	N

478	NPP	488.2726	1	18.359	278304.9	487.2653	tri/oligopeptic	N
479	NPP	490.215	1	2.527783	238089.4	489.2077	tri/oligopeptic	N
480	NPP	249.1314	2	12.98966	115672	496.2482	tri/oligopeptic	N
481	NPP	502.2676	1	27.86498	139026.4	501.2603	tri/oligopeptic	N
482	NPP	251.6531	2	2.341342	115824.5	501.2917	tri/oligopeptic	N
483	NPP	504.2824	1	23.76127	1011205	503.2752	tri/oligopeptic	N
484	NPP	504.2829	1	18.97255	117518.7	503.2756	tri/oligopeptic	N
485	NPP	505.2673	1	23.13577	165374.1	504.26	tri/oligopeptic	N
486	NPP	510.3302	1	22.55003	109858.9	509.3229	tri/oligopeptic	N
487	NPP	512.3447	1	26.09214	2170926	511.3375	tri/oligopeptic	N
488	NPP	512.3453	1	21.66011	534415.1	511.338	tri/oligopeptic	N
489	NPP	512.3456	1	28.24036	458048.5	511.3383	tri/oligopeptic	N
490	NPP	518.2462	1	2.527783	254438.8	517.2389	tri/oligopeptic	N
491	NPP	518.2466	1	14.67538	529240.8	517.2393	tri/oligopeptic	N
492	NPP	532.2627	1	14.21914	171160.4	531.2555	tri/oligopeptic	N
493	NPP	267.1504	2	2.0919	170877.8	532.2862	tri/oligopeptic	N
494	NPP	534.2932	1	23.85065	978209.1	533.2859	tri/oligopeptic	N
495	NPP	536.2729	1	16.95713	106731.9	535.2656	tri/oligopeptic	N
496	NPP	540.3769	1	27.65308	395416.2	539.3696	tri/oligopeptic	N
497	NPP	541.335	1	16.44463	516269.7	540.3278	tri/oligopeptic	N
498	NPP	271.1713	2	16.43528	273191.9	540.328	tri/oligopeptic	N
499	NPP	271.177	2	14.63796	270021.9	540.3394	tri/oligopeptic	N
500	NPP	271.1898	2	19.28166	143106.6	540.365	tri/oligopeptic	N
501	NPP	542.3564	1	26.25365	171235.8	541.3492	tri/oligopeptic	N
502	NPP	546.3294	1	25.88028	1167098	545.3222	tri/oligopeptic	N
503	NPP	546.3303	1	25.61011	238227.8	545.3231	tri/oligopeptic	N
504	NPP	279.169	2	18.07656	274599.2	556.3235	tri/oligopeptic	N
505	NPP	557.331	1	18.07656	283676.3	556.3237	tri/oligopeptic	N
506	NPP	559.0208	1	1.634867	109159.1	558.0135	tri/oligopeptic	N
507	NPP	559.2731	1	2.352533	154160.8	558.2658	tri/oligopeptic	N
508	NPP	570.3266	1	22.46247	241477.5	569.3193	tri/oligopeptic	N
509	NPP	286.1512	2	1.967492	171795.4	570.2879	tri/oligopeptic	N
510	NPP	286.1587	2	17.53155	191578.2	570.3028	tri/oligopeptic	N
511	NPP	571.3102	1	17.55228	254391.4	570.3029	tri/oligopeptic	N
512	NPP	573.3255	1	14.71003	160235	572.3183	tri/oligopeptic	N
513	NPP	577.2847	1	14.09738	120113.4	576.2774	tri/oligopeptic	N
514	NPP	583.3471	1	22.23785	198922.4	582.3399	tri/oligopeptic	N
515	NPP	292.6922	2	2.2504	409023.4	583.3699	tri/oligopeptic	N
516	NPP	585.3627	1	21.46353	183291.9	584.3554	tri/oligopeptic	N
517	NPP	587.3051	1	18.30486	589218.5	586.2978	tri/oligopeptic	N
518	NPP	588.2893	1	13.91764	362424.8	587.282	tri/oligopeptic	N
519	NPP	299.7008	2	19.13617	133797.2	597.387	tri/oligopeptic	N
520	NPP	300.1796	2	2.267058	389473.2	598.3446	tri/oligopeptic	N
521	NPP	601.321	1	18.46895	336360.1	600.3137	tri/oligopeptic	N
522	NPP	306.6954	2	2.685492	388367.8	611.3763	tri/oligopeptic	N
523	NPP	306.6959	2	10.97906	381492.9	611.3772	tri/oligopeptic	N
524	NPP	623.2687	1	19.32909	107817	622.2615	oligopeptide	N
525	NPP	312.1802	2	20.4447	188449	622.3459	oligopeptide	N
526	NPP	314.6878	2	18.94773	104729.3	627.3609	oligopeptide	N
527	NPP	633.3259	1	19.30373	123859.9	632.3187	oligopeptide	N

528	NPP	320.7241	2	19.0305	459697.2	639.4335	oligopeptide	N
529	NPP	649.3217	1	24.80836	118904.1	648.3144	oligopeptide	N
530	NPP	655.3681	1	19.0305	226003	654.3608	oligopeptide	N
531	NPP	330.1621	2	14.19271	124572.5	658.3097	oligopeptide	N
532	NPP	331.6502	2	2.114667	109071	661.2859	oligopeptide	N
533	NPP	667.2745	1	23.93218	128357	666.2672	oligopeptide	N
534	NPP	334.7273	2	25.85133	372809.3	667.4401	oligopeptide	N
535	NPP	341.1643	2	2.224983	125860.4	680.314	oligopeptide	N
536	NPP	348.1909	2	15.2375	125829.6	694.3673	oligopeptide	N
537	NPP	350.2245	2	19.4093	1439564	698.4344	oligopeptide	N
538	NPP	358.1752	2	16.32931	196984.5	714.3359	oligopeptide	N
539	NPP	742.4007	1	21.43413	140661.6	741.3935	oligopeptide	N
540	NPP	382.2407	2	26.09214	103907.2	762.4669	oligopeptide	N
541	NPP	387.2125	2	17.10144	135714.3	772.4105	oligopeptide	N
542	NPP	389.2487	2	27.78449	160054.2	776.4828	oligopeptide	N
543	NPP	434.7078	2	14.01948	710701.6	867.401	oligopeptide	N
544	NPP	460.6668	2	14.27085	141060.2	919.319	oligopeptide	N
545	NPP	943.5277	1	34.95814	258867.9	942.5205	oligopeptide	N
546	NPP	476.2075	2	17.43158	108242.8	950.4005	oligopeptide	N
547	NPP	1058.776	2	1.613858	107629.3	2115.538	oligopeptide	N
ID	Protein	mz	charge	retentiont	raw.abund	mass	type	Match
2	NWP	231.1709	1	5.269683	751074.9	230.1636	di/tripeptide	Y
3	NWP	231.1709	1	9.647	529367.1	230.1637	di/tripeptide	Y
4	NWP	231.171	1	12.05013	285199	230.1637	di/tripeptide	Y
5	NWP	233.1499	1	2.19015	136905.2	232.1426	di/tripeptide	Y
11	NWP	245.1864	1	16.43062	1580142	244.1791	di/tripeptide	Y
12	NWP	245.1867	1	15.89729	114196.4	244.1794	di/tripeptide	Y
13	NWP	245.1864	1	14.53345	1650914	244.1791	di/tripeptide	Y
15	NWP	246.1452	1	1.949417	338951.9	245.1379	di/tripeptide	Y
15	NWP	246.1452	1	2.19015	308511.7	245.1379	di/tripeptide	Y
16	NWP	246.1453	1	2.646058	302860.7	245.1381	di/tripeptide	Y
20	NWP	247.1292	1	2.2005	276591.7	246.1219	di/tripeptide	Y
22	NWP	247.1295	1	4.70545	156973.5	246.1222	di/tripeptide	Y
23	NWP	249.1272	1	2.755942	849790.5	248.1199	di/tripeptide	Y
24	NWP	250.1781	1	34.86262	753131.7	249.1708	di/tripeptide	Y
25	NWP	250.1782	1	37.29748	218958.9	249.1709	di/tripeptide	Y
27	NWP	253.1187	1	2.297892	190787.6	252.1114	di/tripeptide	Y
30	NWP	260.1609	1	2.19015	751120.5	259.1536	di/tripeptide	Y
31	NWP	260.161	1	2.65545	440965.2	259.1537	di/tripeptide	Y
34	NWP	261.145	1	2.19015	165593.1	260.1377	di/tripeptide	Y
36	NWP	261.1452	1	5.452742	225128.2	260.1379	di/tripeptide	Y
38	NWP	263.1431	1	11.51343	284676.6	262.1358	di/tripeptide	Y
42	NWP	269.1612	1	2.169475	378214.4	268.1539	di/tripeptide	Y
48	NWP	290.1716	1	2.375042	352940.6	289.1643	di/tripeptide	Y
51	NWP	295.1298	1	3.294067	114341.8	294.1225	di/tripeptide	Y
57	NWP	302.2083	1	19.86438	173567.1	301.201	tri/oligopeptic	Y
58	NWP	303.1668	1	2.19015	152696.7	302.1596	tri/oligopeptic	Y
64	NWP	318.1667	1	2.2005	181957.6	317.1594	tri/oligopeptic	Y
70	NWP	325.1136	1	1.727833	135564.3	324.1063	tri/oligopeptic	Y
72	NWP	331.1618	1	1.900858	266162.3	330.1545	tri/oligopeptic	Y

73	NWP	331.1659	1	2.82415	137988.6	330.1587	tri/oligopeptic	Y
79	NWP	333.1568	1	11.03227	143539.4	332.1495	tri/oligopeptic	Y
86	NWP	344.2553	1	20.1807	366655.9	343.248	tri/oligopeptic	Y
89	NWP	358.271	1	24.01123	2064993	357.2638	tri/oligopeptic	Y
90	NWP	359.2298	1	2.474208	167034.9	358.2225	tri/oligopeptic	Y
91	NWP	360.1962	1	18.77164	171093.6	359.1889	tri/oligopeptic	Y
112	NWP	415.212	1	36.46915	201092.2	414.2047	tri/oligopeptic	Y
133	NWP	461.3007	1	37.26621	108545.3	460.2934	tri/oligopeptic	Y
141	NWP	479.3105	1	37.15253	159819	478.3032	tri/oligopeptic	Y
143	NWP	488.2471	1	2.064292	111729.5	487.2398	tri/oligopeptic	Y
144	NWP	488.2516	1	19.13203	180810.9	487.2444	tri/oligopeptic	Y
159	NWP	520.2781	1	21.12729	167614	519.2708	tri/oligopeptic	Y
161	NWP	264.1749	2	35.30065	106303.3	526.3353	tri/oligopeptic	Y
174	NWP	274.1678	2	35.95148	109706.4	546.3211	tri/oligopeptic	Y
296	NWP	974.8151	1	1.611425	444833.4	973.8078	oligopeptide	Y
297	NWP	990.7889	1	1.611425	302196.3	989.7817	oligopeptide	Y
338	NWP	229.1553	1	8.991208	510421.5	228.148	di/tripeptide	N
339	NWP	229.1553	1	4.962333	112471.8	228.148	di/tripeptide	N
340	NWP	229.1554	1	11.87278	274090.7	228.1481	di/tripeptide	N
341	NWP	231.1709	1	8.911225	159299.2	230.1636	di/tripeptide	N
342	NWP	232.1295	1	2.19015	203188.7	231.1223	di/tripeptide	N
343	NWP	233.1135	1	2.2005	133331.9	232.1062	di/tripeptide	N
344	NWP	244.1295	1	1.804642	285480.1	243.1223	di/tripeptide	N
345	NWP	244.1296	1	2.075975	211139.7	243.1223	di/tripeptide	N
346	NWP	263.1396	1	14.44101	795432.5	262.1323	di/tripeptide	N
347	NWP	263.1398	1	16.45443	119597.3	262.1325	di/tripeptide	N
348	NWP	275.1355	1	1.704283	315861.6	274.1282	di/tripeptide	N
349	NWP	276.1559	1	2.2005	149380.4	275.1486	di/tripeptide	N
350	NWP	279.1347	1	3.675558	120866.5	278.1274	di/tripeptide	N
351	NWP	279.1711	1	19.91464	262903.3	278.1638	di/tripeptide	N
352	NWP	279.2323	1	37.02085	294317.1	278.225	di/tripeptide	N
353	NWP	281.114	1	11.57606	106631.8	280.1067	di/tripeptide	N
354	NWP	281.1504	1	5.970525	119847.8	280.1431	di/tripeptide	N
355	NWP	290.1715	1	2.19015	106110	289.1642	di/tripeptide	N
356	NWP	292.1508	1	1.925175	177428.2	291.1435	di/tripeptide	N
357	NWP	297.2419	1	37.78043	108275.1	296.2346	di/tripeptide	N
358	NWP	297.2429	1	36.15063	114381.4	296.2356	di/tripeptide	N
359	NWP	302.1717	1	2.264992	212497.3	301.1644	tri/oligopeptic	N
360	NWP	302.1717	1	2.482642	333843	301.1645	tri/oligopeptic	N
361	NWP	302.2084	1	7.07575	268760.6	301.2011	tri/oligopeptic	N
362	NWP	304.1876	1	6.53155	228898.2	303.1803	tri/oligopeptic	N
363	NWP	311.1243	1	2.19015	103475.2	310.117	tri/oligopeptic	N
364	NWP	313.1757	1	2.19015	109224.1	312.1684	tri/oligopeptic	N
365	NWP	313.2378	1	35.80482	172678.5	312.2305	tri/oligopeptic	N
366	NWP	314.2084	1	12.98423	205828.3	313.2011	tri/oligopeptic	N
367	NWP	315.1668	1	2.075975	129256.7	314.1595	tri/oligopeptic	N
368	NWP	315.2535	1	36.14357	323371.9	314.2462	tri/oligopeptic	N
369	NWP	316.1878	1	13.01736	106577.7	315.1805	tri/oligopeptic	N
370	NWP	316.224	1	19.08468	242339.8	315.2167	tri/oligopeptic	N
371	NWP	317.1826	1	2.2005	221057.4	316.1753	tri/oligopeptic	N

372	NWP	317.1827	1	2.413875	359351.9	316.1754	tri/oligopeptic	N
373	NWP	318.1492	1	3.58605	352480.6	317.1419	tri/oligopeptic	N
374	NWP	318.203	1	2.330867	157161.6	317.1957	tri/oligopeptic	N
375	NWP	318.203	1	14.60249	176700.2	317.1958	tri/oligopeptic	N
376	NWP	328.2241	1	17.60873	464718	327.2169	tri/oligopeptic	N
377	NWP	328.2242	1	16.87166	212391	327.2169	tri/oligopeptic	N
378	NWP	329.1828	1	2.523975	234015.4	328.1755	tri/oligopeptic	N
379	NWP	330.2034	1	11.84241	538802.9	329.1962	tri/oligopeptic	N
380	NWP	331.1982	1	2.38605	561522.3	330.191	tri/oligopeptic	N
381	NWP	331.1986	1	6.315492	148326.8	330.1913	tri/oligopeptic	N
382	NWP	332.1825	1	9.681408	696536.4	331.1753	tri/oligopeptic	N
383	NWP	332.2189	1	18.59788	447388.8	331.2116	tri/oligopeptic	N
384	NWP	333.1774	1	2.2005	226025.9	332.1702	tri/oligopeptic	N
385	NWP	334.177	1	18.04588	765373.2	333.1697	tri/oligopeptic	N
386	NWP	337.2354	1	36.15063	115993.7	336.2282	tri/oligopeptic	N
387	NWP	342.2398	1	18.77164	323650.8	341.2325	tri/oligopeptic	N
388	NWP	342.2399	1	21.29517	195697.3	341.2326	tri/oligopeptic	N
389	NWP	343.1984	1	2.2005	127222.6	342.1911	tri/oligopeptic	N
390	NWP	345.2142	1	14.60249	100216.2	344.2069	tri/oligopeptic	N
391	NWP	350.1722	1	17.51015	274494.1	349.1649	tri/oligopeptic	N
392	NWP	351.0963	1	30.8487	138658.8	350.089	tri/oligopeptic	N
393	NWP	357.214	1	2.2218	345269.4	356.2068	tri/oligopeptic	N
394	NWP	357.2142	1	2.523975	705901.3	356.2069	tri/oligopeptic	N
395	NWP	357.2145	1	13.23047	159492.2	356.2072	tri/oligopeptic	N
396	NWP	357.2146	1	12.47918	202494.3	356.2073	tri/oligopeptic	N
397	NWP	357.2146	1	14.9649	156374.6	356.2073	tri/oligopeptic	N
398	NWP	358.1729	1	1.925175	150544.4	357.1656	tri/oligopeptic	N
399	NWP	361.1725	1	2.158175	102200.6	360.1653	tri/oligopeptic	N
400	NWP	361.2089	1	2.2005	106352.9	360.2016	tri/oligopeptic	N
401	NWP	362.212	1	18.18833	107241	361.2047	tri/oligopeptic	N
402	NWP	372.1885	1	1.949417	453826.5	371.1812	tri/oligopeptic	N
403	NWP	372.1885	1	2.19015	890112.7	371.1812	tri/oligopeptic	N
404	NWP	373.2454	1	18.82214	254642.8	372.2381	tri/oligopeptic	N
405	NWP	373.2458	1	3.017792	177284.4	372.2385	tri/oligopeptic	N
406	NWP	374.2041	1	2.19015	228258.7	373.1968	tri/oligopeptic	N
407	NWP	376.1834	1	1.859958	110089.9	375.1762	tri/oligopeptic	N
408	NWP	376.2271	1	20.34811	1202813	375.2198	tri/oligopeptic	N
409	NWP	378.1706	1	10.52969	345752	377.1633	tri/oligopeptic	N
410	NWP	380.1829	1	11.33939	801394.6	379.1756	tri/oligopeptic	N
411	NWP	385.2459	1	19.1687	133336.3	384.2386	tri/oligopeptic	N
412	NWP	389.2038	1	2.2218	153646.9	388.1965	tri/oligopeptic	N
413	NWP	391.1988	1	14.57108	659070.6	390.1916	tri/oligopeptic	N
414	NWP	391.1989	1	17.28833	377210.3	390.1916	tri/oligopeptic	N
415	NWP	391.2198	1	2.96565	706201.7	390.2125	tri/oligopeptic	N
416	NWP	391.2855	1	1.009117	252947.3	390.2782	tri/oligopeptic	N
417	NWP	403.22	1	11.78285	138062.2	402.2128	tri/oligopeptic	N
418	NWP	404.1783	1	1.813833	116245.6	403.171	tri/oligopeptic	N
419	NWP	404.2144	1	2.19015	102649.8	403.2071	tri/oligopeptic	N
420	NWP	411.2614	1	18.2323	185600.2	410.2542	tri/oligopeptic	N
421	NWP	413.267	1	36.76962	1022574	412.2597	tri/oligopeptic	N

422	NWP	415.2354	1	27.25199	270543.3	414.2281	tri/oligopeptic	N
423	NWP	428.2517	1	16.89563	419112.4	427.2444	tri/oligopeptic	N
424	NWP	428.2518	1	15.45257	151864	427.2445	tri/oligopeptic	N
425	NWP	431.2508	1	14.21524	1262631	430.2435	tri/oligopeptic	N
426	NWP	432.2466	1	10.72683	100416.2	431.2393	tri/oligopeptic	N
427	NWP	436.343	1	35.95148	510377.1	435.3357	tri/oligopeptic	N
428	NWP	220.119	2	2.028933	135454.3	438.2234	tri/oligopeptic	N
429	NWP	441.3088	1	24.44906	107153.1	440.3015	tri/oligopeptic	N
430	NWP	442.2668	1	19.02848	850859.8	441.2595	tri/oligopeptic	N
431	NWP	443.2256	1	2.19015	144791.4	442.2183	tri/oligopeptic	N
432	NWP	443.2875	1	17.37	388847.8	442.2803	tri/oligopeptic	N
433	NWP	444.2458	1	2.2005	260733.1	443.2385	tri/oligopeptic	N
434	NWP	445.3037	1	25.33028	113413.6	444.2964	tri/oligopeptic	N
435	NWP	446.2048	1	20.64898	153557.9	445.1975	tri/oligopeptic	N
436	NWP	446.2076	1	2.297892	514094.4	445.2004	tri/oligopeptic	N
437	NWP	447.2253	1	17.09128	128961.4	446.218	tri/oligopeptic	N
438	NWP	447.2617	1	23.54789	130328.4	446.2544	tri/oligopeptic	N
439	NWP	449.2407	1	22.39833	224462.6	448.2335	tri/oligopeptic	N
440	NWP	455.3243	1	26.10715	233596.2	454.3171	tri/oligopeptic	N
441	NWP	455.3246	1	26.81373	105464.9	454.3173	tri/oligopeptic	N
442	NWP	456.2831	1	13.74552	358893.3	455.2758	tri/oligopeptic	N
443	NWP	456.2831	1	12.8648	119815.7	455.2758	tri/oligopeptic	N
444	NWP	456.2913	1	37.38384	202614.5	455.2841	tri/oligopeptic	N
445	NWP	459.2205	1	2.028933	342042.8	458.2132	tri/oligopeptic	N
446	NWP	460.2778	1	2.950692	100326.1	459.2705	tri/oligopeptic	N
447	NWP	461.2438	1	20.25896	275795.4	460.2365	tri/oligopeptic	N
448	NWP	469.2412	1	2.19015	860719.4	468.2339	tri/oligopeptic	N
449	NWP	235.6586	2	14.86473	155458.8	469.3027	tri/oligopeptic	N
450	NWP	471.2571	1	2.264992	818021.1	470.2498	tri/oligopeptic	N
451	NWP	472.2416	1	13.00084	318947.6	471.2343	tri/oligopeptic	N
452	NWP	475.2517	1	2.2005	312247.6	474.2444	tri/oligopeptic	N
453	NWP	478.2311	1	10.69933	126827.5	477.2238	tri/oligopeptic	N
454	NWP	478.2311	1	18.57301	324956.8	477.2238	tri/oligopeptic	N
455	NWP	485.2724	1	2.19015	272840.2	484.2651	tri/oligopeptic	N
456	NWP	485.2732	1	15.45257	628347.3	484.2659	tri/oligopeptic	N
457	NWP	485.2732	1	12.66652	204823	484.266	tri/oligopeptic	N
458	NWP	485.2733	1	13.71265	355684.9	484.266	tri/oligopeptic	N
459	NWP	243.1403	2	2.710892	133855	484.266	tri/oligopeptic	N
460	NWP	488.2518	1	20.35511	266815.1	487.2445	tri/oligopeptic	N
461	NWP	489.2674	1	2.2005	135946.9	488.2601	tri/oligopeptic	N
462	NWP	489.2681	1	13.76726	108955.9	488.2608	tri/oligopeptic	N
463	NWP	489.2721	1	19.69391	330110.7	488.2648	tri/oligopeptic	N
464	NWP	489.2721	1	17.63446	444219.6	488.2649	tri/oligopeptic	N
465	NWP	494.2726	1	2.19015	200213.6	493.2653	tri/oligopeptic	N
466	NWP	247.6401	2	2.2005	624962.3	493.2657	tri/oligopeptic	N
467	NWP	249.6744	2	16.04743	138490.1	497.3342	tri/oligopeptic	N
468	NWP	500.247	1	2.064292	672762.4	499.2397	tri/oligopeptic	N
469	NWP	250.6272	2	2.064292	102314.7	499.2399	tri/oligopeptic	N
470	NWP	502.263	1	2.19015	153553.5	501.2557	tri/oligopeptic	N
471	NWP	504.2679	1	12.7932	273340.1	503.2606	tri/oligopeptic	N

472	NWP	504.2831	1	22.72198	215678	503.2758	tri/oligopeptic	N
473	NWP	510.2679	1	2.19015	134438.4	509.2606	tri/oligopeptic	N
474	NWP	255.6376	2	2.2005	259336.9	509.2606	tri/oligopeptic	N
475	NWP	512.3459	1	26.21453	176957.6	511.3386	tri/oligopeptic	N
476	NWP	519.2574	1	16.43062	1329228	518.2502	tri/oligopeptic	N
477	NWP	519.2575	1	17.98209	161708.1	518.2502	tri/oligopeptic	N
478	NWP	260.1325	2	16.25144	343839.8	518.2505	tri/oligopeptic	N
479	NWP	529.2625	1	2.19015	114254.1	528.2552	tri/oligopeptic	N
480	NWP	529.2995	1	15.53021	328388.6	528.2922	tri/oligopeptic	N
481	NWP	533.2395	1	2.2218	175348.8	532.2322	tri/oligopeptic	N
482	NWP	535.2525	1	14.71921	226206.2	534.2452	tri/oligopeptic	N
483	NWP	535.2526	1	13.17164	127245.6	534.2453	tri/oligopeptic	N
484	NWP	539.3202	1	15.54673	229502	538.3129	tri/oligopeptic	N
485	NWP	270.1638	2	15.54673	192434.1	538.3131	tri/oligopeptic	N
486	NWP	541.335	1	22.83464	1646362	540.3277	tri/oligopeptic	N
487	NWP	543.2463	1	16.70842	706404.7	542.239	tri/oligopeptic	N
488	NWP	543.2942	1	26.16703	197894.8	542.2869	tri/oligopeptic	N
489	NWP	545.2943	1	14.20503	244934.2	544.287	tri/oligopeptic	N
490	NWP	556.2736	1	2.19015	402217.2	555.2663	tri/oligopeptic	N
491	NWP	278.6591	2	13.34393	318717.4	555.3036	tri/oligopeptic	N
492	NWP	556.311	1	13.33695	211487.6	555.3037	tri/oligopeptic	N
493	NWP	558.2903	1	2.2005	155702.9	557.283	tri/oligopeptic	N
494	NWP	559.3104	1	15.86916	283326.9	558.3031	tri/oligopeptic	N
495	NWP	566.2943	1	2.2005	235565.9	565.287	tri/oligopeptic	N
496	NWP	567.8931	1	1.611425	137066.8	566.8859	tri/oligopeptic	N
497	NWP	285.1873	2	22.06907	249262.4	568.36	tri/oligopeptic	N
498	NWP	569.3675	1	22.06215	349285.9	568.3602	tri/oligopeptic	N
499	NWP	285.193	2	18.41222	126282.3	568.3715	tri/oligopeptic	N
500	NWP	576.3	1	2.232533	107064.6	575.2927	tri/oligopeptic	N
501	NWP	290.1526	2	15.20923	100682.7	578.2907	tri/oligopeptic	N
502	NWP	582.3264	1	15.94964	100376	581.3192	tri/oligopeptic	N
503	NWP	582.3265	1	17.3345	124469.1	581.3192	tri/oligopeptic	N
504	NWP	584.3423	1	19.22847	222144.7	583.3351	tri/oligopeptic	N
505	NWP	585.3257	1	20.26458	632390.3	584.3184	tri/oligopeptic	N
506	NWP	587.2794	1	2.169475	192696.5	586.2721	tri/oligopeptic	N
507	NWP	294.18	2	2.700767	146388.3	586.3455	tri/oligopeptic	N
508	NWP	588.3414	1	20.60566	354722.6	587.3341	tri/oligopeptic	N
509	NWP	597.3001	1	2.2005	528834.9	596.2928	tri/oligopeptic	N
510	NWP	299.1538	2	2.232533	120356	596.2931	tri/oligopeptic	N
511	NWP	598.3573	1	14.6702	198320.1	597.35	tri/oligopeptic	N
512	NWP	299.6823	2	14.6702	297616.2	597.3501	tri/oligopeptic	N
513	NWP	599.3156	1	2.297892	196184.4	598.3083	tri/oligopeptic	N
514	NWP	300.1619	2	2.319817	107435	598.3092	tri/oligopeptic	N
515	NWP	600.2678	1	18.87613	319562.7	599.2605	tri/oligopeptic	N
516	NWP	601.3314	1	2.2005	142552.6	600.3241	tri/oligopeptic	N
517	NWP	601.3364	1	23.88409	113896.2	600.3291	tri/oligopeptic	N
518	NWP	301.1719	2	23.90773	101328.3	600.3293	tri/oligopeptic	N
519	NWP	605.3157	1	14.23519	122040.8	604.3085	tri/oligopeptic	N
520	NWP	606.2898	1	18.92618	116397.4	605.2825	tri/oligopeptic	N
521	NWP	606.2899	1	16.27453	453198.3	605.2826	tri/oligopeptic	N

522	NWP	303.6486	2	16.27453	558224.6	605.2826	tri/oligopeptic	N
523	NWP	303.654	2	2.0994	109159.5	605.2934	tri/oligopeptic	N
524	NWP	306.1125	2	2.2005	158836.2	610.2104	tri/oligopeptic	N
525	NWP	307.1697	2	14.33174	353744.9	612.3248	tri/oligopeptic	N
526	NWP	613.3321	1	14.34803	404762.2	612.3248	tri/oligopeptic	N
527	NWP	307.1699	2	12.01208	113021	612.3253	tri/oligopeptic	N
528	NWP	307.1699	2	13.50965	200912.6	612.3253	tri/oligopeptic	N
529	NWP	613.3327	1	13.51762	111071.6	612.3254	tri/oligopeptic	N
530	NWP	615.312	1	2.19015	150820.7	614.3047	oligopeptide	N
531	NWP	616.3106	1	20.35511	821727.1	615.3033	oligopeptide	N
532	NWP	308.6591	2	18.17968	304023	615.3036	oligopeptide	N
533	NWP	616.311	1	18.19462	297451.6	615.3037	oligopeptide	N
534	NWP	308.6591	2	17.21655	299114	615.3037	oligopeptide	N
535	NWP	616.311	1	17.29663	230491.6	615.3038	oligopeptide	N
536	NWP	309.6669	2	15.63978	136600.8	617.3193	oligopeptide	N
537	NWP	618.3266	1	15.63978	103019.2	617.3194	oligopeptide	N
538	NWP	622.2845	1	14.66008	171773.1	621.2773	oligopeptide	N
539	NWP	628.306	1	2.19015	378700.8	627.2987	oligopeptide	N
540	NWP	315.1564	2	14.57108	973137.8	628.2982	oligopeptide	N
541	NWP	315.6278	2	16.41447	113579.2	629.241	oligopeptide	N
542	NWP	632.3427	1	24.74464	128906.4	631.3354	oligopeptide	N
543	NWP	633.3221	1	2.846517	120153.1	632.3148	oligopeptide	N
544	NWP	635.8805	1	1.611425	147780.1	634.8733	oligopeptide	N
545	NWP	319.1488	2	14.34803	150296.9	636.283	oligopeptide	N
546	NWP	647.3165	1	16.38483	453203.2	646.3092	oligopeptide	N
547	NWP	324.1619	2	16.37593	1234482	646.3093	oligopeptide	N
548	NWP	324.6543	2	17.07485	120537.4	647.294	oligopeptide	N
549	NWP	651.3373	1	23.47418	128337.3	650.33	oligopeptide	N
550	NWP	326.7244	2	15.49975	187273.2	651.4343	oligopeptide	N
551	NWP	328.6927	2	26.44001	762778.1	655.3709	oligopeptide	N
552	NWP	656.3783	1	26.44001	739295.2	655.371	oligopeptide	N
553	NWP	329.1723	2	21.8573	640695.4	656.3301	oligopeptide	N
554	NWP	657.3376	1	21.84747	187091.5	656.3304	oligopeptide	N
555	NWP	333.7196	2	21.11093	343420.2	665.4246	oligopeptide	N
556	NWP	670.3532	1	2.2005	148134.2	669.3459	oligopeptide	N
557	NWP	336.1409	2	16.39481	308816.3	670.2673	oligopeptide	N
558	NWP	672.3375	1	26.08537	295510.3	671.3302	oligopeptide	N
559	NWP	336.6725	2	26.07833	139952.1	671.3303	oligopeptide	N
560	NWP	341.2014	2	15.90666	128862.2	680.3883	oligopeptide	N
561	NWP	684.3323	1	2.2005	194210.7	683.325	oligopeptide	N
562	NWP	343.1356	2	16.39481	147285.8	684.2567	oligopeptide	N
563	NWP	344.178	2	18.22415	103400	686.3415	oligopeptide	N
564	NWP	692.3267	1	14.6702	205917	691.3194	oligopeptide	N
565	NWP	346.6672	2	14.69342	115339.3	691.3199	oligopeptide	N
566	NWP	346.691	2	2.474208	124477.4	691.3675	oligopeptide	N
567	NWP	350.6936	2	22.41069	291141.7	699.3726	oligopeptide	N
568	NWP	703.3427	1	18.79297	1076866	702.3354	oligopeptide	N
569	NWP	352.1752	2	18.80085	211254.6	702.3358	oligopeptide	N
570	NWP	355.6963	2	14.47028	121122.7	709.3781	oligopeptide	N
571	NWP	355.6965	2	17.51015	204575.8	709.3784	oligopeptide	N

572	NWP	355.6966	2	16.81488	231828.2	709.3786	oligopeptide	N
573	NWP	356.147	2	16.93706	154370.5	710.2795	oligopeptide	N
574	NWP	357.1858	2	21.11093	175085.8	712.3571	oligopeptide	N
575	NWP	364.1541	2	18.80085	195265.8	726.2936	oligopeptide	N
576	NWP	728.327	1	19.08468	206668.9	727.3198	oligopeptide	N
577	NWP	365.2014	2	20.36851	189717.1	728.3882	oligopeptide	N
578	NWP	371.1991	2	14.61663	299800.7	740.3837	oligopeptide	N
579	NWP	371.1995	2	13.86817	292414.4	740.3844	oligopeptide	N
580	NWP	372.6885	2	20.31109	321381	743.3625	oligopeptide	N
581	NWP	372.6887	2	19.49727	227885.4	743.3628	oligopeptide	N
582	NWP	372.6887	2	18.17968	208512.8	743.3628	oligopeptide	N
583	NWP	756.3908	1	14.69342	115790.6	755.3835	oligopeptide	N
584	NWP	378.6992	2	14.69342	208459.7	755.3838	oligopeptide	N
585	NWP	386.7101	2	16.62428	125776.4	771.4056	oligopeptide	N
586	NWP	388.1916	2	16.54954	142845.8	774.3686	oligopeptide	N
587	NWP	389.7277	2	18.79297	118152.4	777.4409	oligopeptide	N
588	NWP	392.2043	2	2.297892	149014.6	782.394	oligopeptide	N
589	NWP	396.7358	2	19.83368	117401.5	791.457	oligopeptide	N
590	NWP	397.7487	2	19.01612	818930.6	793.4829	oligopeptide	N
591	NWP	399.2126	2	15.70601	642094	796.4106	oligopeptide	N
592	NWP	400.7016	2	19.62132	660788.2	799.3886	oligopeptide	N
593	NWP	800.396	1	19.62132	264939.7	799.3888	oligopeptide	N
594	NWP	400.7021	2	24.41042	112998.3	799.3897	oligopeptide	N
595	NWP	406.2382	2	20.34154	183220.6	810.4619	oligopeptide	N
596	NWP	408.2073	2	18.52414	226239	814.4001	oligopeptide	N
597	NWP	408.7176	2	20.59078	170978.6	815.4207	oligopeptide	N
598	NWP	410.7207	2	14.18893	172006.1	819.4268	oligopeptide	N
599	NWP	411.1915	2	15.71728	117821.3	820.3684	oligopeptide	N
600	NWP	412.2383	2	22.52834	588853.1	822.4621	oligopeptide	N
601	NWP	412.6805	2	19.62132	131954.3	823.3464	oligopeptide	N
602	NWP	416.2044	2	18.86353	379605.7	830.3942	oligopeptide	N
603	NWP	416.2045	2	20.20967	234126.9	830.3945	oligopeptide	N
604	NWP	419.7258	2	13.97034	509019.6	837.4371	oligopeptide	N
605	NWP	420.1762	2	16.45443	159415.6	838.3379	oligopeptide	N
606	NWP	421.2151	2	19.49727	202516.5	840.4156	oligopeptide	N
607	NWP	421.2153	2	21.28819	113195.4	840.4161	oligopeptide	N
608	NWP	421.7254	2	20.59078	171976.1	841.4363	oligopeptide	N
609	NWP	423.2125	2	18.28506	278587.8	844.4105	oligopeptide	N
610	NWP	428.1992	2	17.95548	231417.9	854.3838	oligopeptide	N
611	NWP	436.7178	2	17.95548	684329.7	871.4211	oligopeptide	N
612	NWP	444.218	2	23.83657	309303	886.4215	oligopeptide	N
613	NWP	447.7571	2	17.47709	600760.7	893.4996	oligopeptide	N
614	NWP	449.1792	2	16.84325	102093.9	896.3438	oligopeptide	N
615	NWP	459.7204	2	18.79297	631684.4	917.4262	oligopeptide	N
616	NWP	460.6667	2	14.24767	135359	919.3188	oligopeptide	N
617	NWP	470.2519	2	22.13375	284625.9	938.4892	oligopeptide	N
618	NWP	471.6993	2	18.80085	128073.3	941.384	oligopeptide	N
619	NWP	471.7391	2	21.02236	132745.1	941.4637	oligopeptide	N
620	NWP	473.742	2	17.49389	103122.9	945.4695	oligopeptide	N
621	NWP	477.7574	2	23.07018	454000.1	953.5002	oligopeptide	N

622	NWP	480.2339	2	20.1807	207742.7	958.4533	oligopeptide	N
623	NWP	485.2445	2	19.29148	327623.6	968.4745	oligopeptide	N
624	NWP	487.242	2	19.85073	210287.9	972.4695	oligopeptide	N
625	NWP	491.7418	2	16.28235	671745.3	981.4691	oligopeptide	N
626	NWP	498.2006	2	2.243275	107806.6	994.3866	oligopeptide	N
627	NWP	506.7786	2	24.44232	169244.8	1011.543	oligopeptide	N
628	NWP	513.7865	2	25.33028	136125.3	1025.558	oligopeptide	N
629	NWP	516.2633	2	23.3991	259884.9	1030.512	oligopeptide	N
630	NWP	523.7497	2	18.80085	386368.1	1045.485	oligopeptide	N
631	NWP	541.787	2	21.65751	142699.3	1081.559	oligopeptide	N
632	NWP	548.8698	2	1.611425	109984.8	1095.725	oligopeptide	N
633	NWP	549.2737	2	19.60824	114048	1096.533	oligopeptide	N
634	NWP	557.2901	2	21.80879	106646.6	1112.566	oligopeptide	N
635	NWP	574.2824	2	23.24356	175230.1	1146.55	oligopeptide	N
636	NWP	588.3136	2	16.43062	135743.3	1174.613	oligopeptide	N
637	NWP	596.3108	2	17.52669	151909.4	1190.607	oligopeptide	N
638	NWP	597.8005	2	20.20967	220996.5	1193.586	oligopeptide	N
639	NWP	597.8008	2	19.82558	127525.6	1193.587	oligopeptide	N
640	NWP	599.7982	2	19.46579	409012.9	1197.582	oligopeptide	N
641	NWP	613.3033	2	18.79297	293047.1	1224.592	oligopeptide	N
642	NWP	619.8193	2	16.25813	107512.6	1237.624	oligopeptide	N
643	NWP	696.8494	2	14.95572	296758.7	1391.684	oligopeptide	N
644	NWP	705.3465	2	20.17156	228969.5	1408.678	oligopeptide	N
645	NWP	718.3726	2	21.8573	177517	1434.731	oligopeptide	N
646	NWP	732.3757	2	17.03131	161694.8	1462.737	oligopeptide	N
647	NWP	804.4043	2	26.43388	100900.9	1606.794	oligopeptide	N
648	NWP	1024.783	2	1.611425	164844.3	2047.551	oligopeptide	N
649	NWP	1058.777	2	1.611425	240714.6	2115.539	oligopeptide	N
650	NWP	717.0233	3	24.47841	302909.2	2148.048	oligopeptide	N
651	NWP	1092.77	2	1.622642	121529.2	2183.525	oligopeptide	N

ID	Protein	mz	charge	retentionti	raw.abund	mass	type	Match
2	BP	231.171	1	5.378708	625083.8	230.1637	di/tripeptide	Y
3	BP	231.1711	1	9.670667	1063120	230.1638	di/tripeptide	Y
4	BP	231.1711	1	12.04521	1229779	230.1638	di/tripeptide	Y
5	BP	233.15	1	2.201208	521386	232.1427	di/tripeptide	Y
6	BP	233.1502	1	2.786958	209835.1	232.143	di/tripeptide	Y
7	BP	239.103	1	2.190508	134390.2	238.0957	di/tripeptide	Y
11	BP	245.1867	1	16.42313	1676322	244.1794	di/tripeptide	Y
12	BP	245.1868	1	15.8879	245695.2	244.1795	di/tripeptide	Y
13	BP	245.1868	1	14.51483	446650.9	244.1795	di/tripeptide	Y
14	BP	245.1868	1	17.67486	582972.2	244.1795	di/tripeptide	Y
15	BP	246.1453	1	2.201208	370495.7	245.138	di/tripeptide	Y
16	BP	246.1455	1	2.631358	184281.4	245.1382	di/tripeptide	Y
17	BP	246.1456	1	3.405142	485877.8	245.1384	di/tripeptide	Y
20	BP	247.1293	1	2.126558	1128987	246.122	di/tripeptide	Y
22	BP	247.1296	1	4.721192	589508.2	246.1223	di/tripeptide	Y
23	BP	249.1275	1	2.725833	279589	248.1202	di/tripeptide	Y
24	BP	250.1784	1	34.86318	770470.4	249.1711	di/tripeptide	Y
25	BP	250.1784	1	37.2597	212118.8	249.1711	di/tripeptide	Y
27	BP	253.1188	1	2.2919	984714.8	252.1115	di/tripeptide	Y

30	BP	260.161	1	2.201208	503863	259.1538	di/tripeptide	Y
31	BP	260.1611	1	2.551008	257285.7	259.1539	di/tripeptide	Y
33	BP	260.1974	1	1.844667	139829.3	259.1901	di/tripeptide	Y
34	BP	261.145	1	2.190508	476441.1	260.1378	di/tripeptide	Y
36	BP	261.1453	1	5.476758	393311.7	260.1381	di/tripeptide	Y
38	BP	263.1433	1	11.5175	259251.8	262.136	di/tripeptide	Y
40	BP	265.1556	1	16.25883	148965.3	264.1483	di/tripeptide	Y
41	BP	267.1345	1	2.401483	824444.5	266.1273	di/tripeptide	Y
42	BP	269.1613	1	2.190508	342425.3	268.1541	di/tripeptide	Y
45	BP	281.1139	1	2.558442	250075.5	280.1066	di/tripeptide	Y
47	BP	288.1928	1	15.93067	102285.5	287.1855	di/tripeptide	Y
50	BP	294.1455	1	2.317767	236668.6	293.1382	di/tripeptide	Y
51	BP	295.13	1	3.348983	145668.1	294.1227	di/tripeptide	Y
52	BP	295.1661	1	16.40204	480260.4	294.1588	di/tripeptide	Y
57	BP	302.2086	1	19.86203	215639.2	301.2013	tri/oligopeptic	Y
59	BP	304.151	1	2.222792	154050	303.1437	tri/oligopeptic	Y
60	BP	304.1513	1	4.90775	899493.5	303.144	tri/oligopeptic	Y
61	BP	304.1667	1	18.98487	326614.3	303.1595	tri/oligopeptic	Y
64	BP	318.1668	1	2.244567	690080.8	317.1595	tri/oligopeptic	Y
65	BP	318.1671	1	9.108958	829494.7	317.1598	tri/oligopeptic	Y
67	BP	318.1827	1	24.7141	123280.9	317.1754	tri/oligopeptic	Y
69	BP	319.1619	1	1.959117	158112.7	318.1546	tri/oligopeptic	Y
73	BP	331.1663	1	2.770708	262811.4	330.159	tri/oligopeptic	Y
75	BP	332.1825	1	2.576742	482236.1	331.1752	tri/oligopeptic	Y
76	BP	332.2192	1	2.818683	117212.3	331.2119	tri/oligopeptic	Y
79	BP	333.157	1	11.04885	494257	332.1497	tri/oligopeptic	Y
80	BP	334.1615	1	2.201208	412958.1	333.1543	tri/oligopeptic	Y
92	BP	360.2142	1	2.643	181142.9	359.2069	tri/oligopeptic	Y
94	BP	363.1559	1	2.190508	127436.2	362.1486	tri/oligopeptic	Y
112	BP	415.2123	1	36.47223	217662.5	414.2051	tri/oligopeptic	Y
114	BP	420.2096	1	2.2919	242467.8	419.2024	tri/oligopeptic	Y
141	BP	479.3109	1	36.99012	150898.5	478.3036	tri/oligopeptic	Y
144	BP	488.2522	1	19.13919	190180.2	487.245	tri/oligopeptic	Y
148	BP	491.2879	1	23.13454	953820	490.2807	tri/oligopeptic	Y
151	BP	500.2889	1	25.19403	140298.3	499.2816	tri/oligopeptic	Y
153	BP	513.2685	1	13.73743	138772.4	512.2612	tri/oligopeptic	Y
159	BP	520.2785	1	21.16339	139516.9	519.2712	tri/oligopeptic	Y
161	BP	264.1752	2	35.28835	106889	526.3358	tri/oligopeptic	Y
165	BP	531.0146	1	1.596683	282829	530.0074	tri/oligopeptic	Y
174	BP	274.1681	2	35.94048	110173.1	546.3217	tri/oligopeptic	Y
187	BP	572.2772	1	19.16912	734901	571.2699	tri/oligopeptic	Y
194	BP	599.0026	1	1.596683	248062.2	597.9953	tri/oligopeptic	Y
218	BP	666.9901	1	1.608117	259936.9	665.9828	oligopeptide	Y
231	BP	702.37	1	18.61288	173453	701.3627	oligopeptide	Y
240	BP	734.9776	1	1.596683	218189.9	733.9704	oligopeptide	Y
258	BP	802.9652	1	1.585458	193038.1	801.958	oligopeptide	Y
264	BP	276.1789	3	15.52703	1343296	825.5148	oligopeptide	Y
276	BP	870.9526	1	1.585458	147370.5	869.9453	oligopeptide	Y
297	BP	990.79	1	1.596683	151081.8	989.7827	oligopeptide	Y
300	BP	514.9584	2	1.596683	112449.6	1027.902	oligopeptide	Y

303	BP	523.7873	2	23.1578	294597.8	1045.56	oligopeptide	Y
307	BP	548.9522	2	1.608117	124133.1	1095.89	oligopeptide	Y
313	BP	582.9463	2	1.596683	122831.9	1163.878	oligopeptide	Y
314	BP	590.9332	2	1.608117	100215.8	1179.852	oligopeptide	Y
316	BP	616.9403	2	1.608117	123697.1	1231.866	oligopeptide	Y
318	BP	624.9269	2	1.596683	111869	1247.839	oligopeptide	Y
321	BP	650.9337	2	1.608117	128075.4	1299.853	oligopeptide	Y
322	BP	658.9207	2	1.608117	108972	1315.827	oligopeptide	Y
329	BP	692.9147	2	1.596683	110335.3	1383.815	oligopeptide	Y
333	BP	760.902	2	1.596683	101012.9	1519.789	oligopeptide	Y
335	BP	794.896	2	1.608117	101060.5	1587.777	oligopeptide	Y
338	BP	223.1084	1	10.38173	165635.6	222.1011	dipeptide	N
339	BP	226.9525	1	1.677442	166223.7	225.9452	di/tripeptide	N
340	BP	229.1191	1	10.25577	151794.7	228.1118	di/tripeptide	N
341	BP	229.1554	1	8.98885	227353.2	228.1481	di/tripeptide	N
342	BP	233.1136	1	1.994208	144599.5	232.1063	di/tripeptide	N
343	BP	233.1504	1	4.1048	496718.7	232.1431	di/tripeptide	N
344	BP	237.0908	1	2.190508	120608.2	236.0835	di/tripeptide	N
345	BP	237.1241	1	4.611867	110915.9	236.1168	di/tripeptide	N
346	BP	237.1241	1	11.19204	327781.6	236.1168	di/tripeptide	N
347	BP	244.1296	1	2.1048	147406.2	243.1223	di/tripeptide	N
348	BP	252.9631	1	9.934192	152778.4	251.9558	di/tripeptide	N
349	BP	255.1456	1	1.783425	205768.8	254.1383	di/tripeptide	N
350	BP	262.1195	1	14.4752	580140	261.1122	di/tripeptide	N
351	BP	263.1433	1	13.09528	173396.8	262.136	di/tripeptide	N
352	BP	265.1556	1	14.74059	265548.2	264.1483	di/tripeptide	N
353	BP	269.1138	1	2.190508	513803.3	268.1065	di/tripeptide	N
354	BP	276.1353	1	14.32788	116733.3	275.128	di/tripeptide	N
355	BP	276.1559	1	2.0604	372575.9	275.1487	di/tripeptide	N
356	BP	276.156	1	2.190508	699997.4	275.1487	di/tripeptide	N
357	BP	276.156	1	2.389858	785000.6	275.1487	di/tripeptide	N
358	BP	279.1714	1	19.89973	394200.4	278.1641	di/tripeptide	N
359	BP	279.1715	1	21.03043	219766.6	278.1642	di/tripeptide	N
360	BP	279.2324	1	37.0111	165050.5	278.2252	di/tripeptide	N
361	BP	281.1142	1	11.55735	160981.5	280.1069	di/tripeptide	N
362	BP	281.1505	1	5.510233	138349.7	280.1432	di/tripeptide	N
363	BP	281.1506	1	6.053717	300770.9	280.1433	di/tripeptide	N
364	BP	283.1293	1	2.201208	145615.9	282.1221	di/tripeptide	N
365	BP	288.1925	1	2.666167	426513.1	287.1853	di/tripeptide	N
366	BP	289.1513	1	2.190508	321953.2	288.144	di/tripeptide	N
367	BP	290.1716	1	2.201208	385068.5	289.1644	di/tripeptide	N
368	BP	292.1301	1	16.41637	466011.2	291.1228	di/tripeptide	N
369	BP	292.1301	1	9.455833	366045.7	291.1228	di/tripeptide	N
370	BP	292.1508	1	2.038392	214025.1	291.1435	di/tripeptide	N
371	BP	295.13	1	12.62893	167975.7	294.1227	di/tripeptide	N
372	BP	295.1663	1	14.5967	143747.1	294.1591	di/tripeptide	N
373	BP	295.1664	1	13.3899	340748.7	294.1591	di/tripeptide	N
374	BP	295.2275	1	37.38036	110241.7	294.2202	di/tripeptide	N
375	BP	302.1717	1	2.222792	127980.7	301.1644	tri/oligopeptic	N
376	BP	302.2086	1	11.18408	118989.6	301.2013	tri/oligopeptic	N

377	BP	304.1668	1	19.9139	173946.5	303.1595	tri/oligopeptic	N
378	BP	304.1874	1	2.190508	590437.2	303.1801	tri/oligopeptic	N
379	BP	304.1879	1	11.34206	130644.5	303.1806	tri/oligopeptic	N
380	BP	306.1302	1	1.856525	101759.4	305.1229	tri/oligopeptic	N
381	BP	306.1668	1	2.190508	780396.8	305.1595	tri/oligopeptic	N
382	BP	311.1244	1	2.190508	478811	310.1171	tri/oligopeptic	N
383	BP	316.1875	1	2.355467	810914.2	315.1802	tri/oligopeptic	N
384	BP	316.188	1	12.97118	243580.9	315.1807	tri/oligopeptic	N
385	BP	316.2244	1	15.40928	119463.5	315.2171	tri/oligopeptic	N
386	BP	316.2244	1	14.80839	111623.3	315.2171	tri/oligopeptic	N
387	BP	318.2031	1	2.317767	117046.6	317.1958	tri/oligopeptic	N
388	BP	318.2037	1	12.38566	162850.9	317.1964	tri/oligopeptic	N
389	BP	319.1413	1	16.81207	100676	318.134	tri/oligopeptic	N
390	BP	320.1253	1	11.79946	119970.6	319.1181	tri/oligopeptic	N
391	BP	320.1254	1	17.65223	166494.3	319.1181	tri/oligopeptic	N
392	BP	320.1459	1	1.925067	153394.6	319.1387	tri/oligopeptic	N
393	BP	324.1298	1	2.190508	569218	323.1225	tri/oligopeptic	N
394	BP	328.2245	1	17.09425	500267.3	327.2172	tri/oligopeptic	N
395	BP	328.2343	1	15.48075	138814.4	327.227	tri/oligopeptic	N
396	BP	331.1984	1	2.201208	149211	330.1911	tri/oligopeptic	N
397	BP	332.146	1	2.038392	151662.4	331.1387	tri/oligopeptic	N
398	BP	332.2192	1	4.856167	582582.7	331.2119	tri/oligopeptic	N
399	BP	332.2193	1	8.98005	532498.8	331.212	tri/oligopeptic	N
400	BP	332.2193	1	15.89443	103267.4	331.212	tri/oligopeptic	N
401	BP	332.2195	1	19.44302	129266.8	331.2122	tri/oligopeptic	N
402	BP	333.1776	1	2.201208	237375.1	332.1704	tri/oligopeptic	N
403	BP	334.1252	1	1.936058	151593.4	333.1179	tri/oligopeptic	N
404	BP	334.1409	1	13.73743	119216.2	333.1336	tri/oligopeptic	N
405	BP	334.1412	1	17.95638	174562.5	333.1339	tri/oligopeptic	N
406	BP	334.1771	1	16.38625	143868.8	333.1698	tri/oligopeptic	N
407	BP	334.1775	1	18.02637	101370.8	333.1702	tri/oligopeptic	N
408	BP	334.1979	1	2.201208	153225.3	333.1906	tri/oligopeptic	N
409	BP	345.2146	1	12.60298	304639.9	344.2074	tri/oligopeptic	N
410	BP	346.1986	1	12.74334	641039.9	345.1914	tri/oligopeptic	N
411	BP	346.2353	1	19.18507	103314.3	345.228	tri/oligopeptic	N
412	BP	347.1569	1	2.038392	119756.8	346.1496	tri/oligopeptic	N
413	BP	348.1774	1	2.201208	370341.1	347.1701	tri/oligopeptic	N
414	BP	350.1752	1	10.06618	124806.7	349.1679	tri/oligopeptic	N
415	BP	352.1517	1	11.26378	956510.1	351.1444	tri/oligopeptic	N
416	BP	357.2141	1	2.190508	425379.9	356.2069	tri/oligopeptic	N
417	BP	360.2144	1	8.789833	503433.4	359.2071	tri/oligopeptic	N
418	BP	361.1726	1	2.201208	391053.9	360.1653	tri/oligopeptic	N
419	BP	362.1931	1	2.244567	1206515	361.1858	tri/oligopeptic	N
420	BP	363.1518	1	1.891675	136193.7	362.1445	tri/oligopeptic	N
421	BP	372.1887	1	2.169517	199660.8	371.1814	tri/oligopeptic	N
422	BP	373.1729	1	2.071533	287741.2	372.1657	tri/oligopeptic	N
423	BP	374.109	1	2.190508	447175.6	373.1017	tri/oligopeptic	N
424	BP	375.2253	1	12.79243	139692.6	374.218	tri/oligopeptic	N
425	BP	378.2036	1	15.08798	564407.1	377.1964	tri/oligopeptic	N
426	BP	382.1619	1	2.522775	347352.5	381.1547	tri/oligopeptic	N

427	BP	389.2039	1	2.201208	105874.6	388.1966	tri/oligopeptic	N
428	BP	393.1988	1	2.190508	131623.9	392.1915	tri/oligopeptic	N
429	BP	399.2617	1	17.65223	233847.6	398.2544	tri/oligopeptic	N
430	BP	405.2352	1	2.212083	375180.7	404.228	tri/oligopeptic	N
431	BP	409.0201	1	2.201208	207427.5	408.0129	tri/oligopeptic	N
432	BP	409.1886	1	23.70576	439877.6	408.1814	tri/oligopeptic	N
433	BP	413.2673	1	36.71189	936003.1	412.26	tri/oligopeptic	N
434	BP	419.2152	1	2.978092	643183.1	418.2079	tri/oligopeptic	N
435	BP	419.2513	1	14.38211	639094.7	418.244	tri/oligopeptic	N
436	BP	424.2093	1	14.50823	158932.6	423.202	tri/oligopeptic	N
437	BP	429.2726	1	20.71539	142548.4	428.2653	tri/oligopeptic	N
438	BP	430.2676	1	14.89067	107990.1	429.2603	tri/oligopeptic	N
439	BP	431.2517	1	14.26715	138983.2	430.2444	tri/oligopeptic	N
440	BP	432.2096	1	2.027433	154209.9	431.2024	tri/oligopeptic	N
441	BP	433.2675	1	19.19297	103897.9	432.2602	tri/oligopeptic	N
442	BP	435.2256	1	17.70002	519512.3	434.2183	tri/oligopeptic	N
443	BP	436.3438	1	1.366417	284646.2	435.3365	tri/oligopeptic	N
444	BP	439.2935	1	23.49402	121088.3	438.2862	tri/oligopeptic	N
445	BP	440.252	1	15.60512	119187.1	439.2448	tri/oligopeptic	N
446	BP	447.2003	1	13.90281	269554.4	446.1931	tri/oligopeptic	N
447	BP	447.2256	1	16.43152	174362.3	446.2183	tri/oligopeptic	N
448	BP	447.2461	1	15.45893	956666	446.2388	tri/oligopeptic	N
449	BP	448.1841	1	18.69215	500190.7	447.1769	tri/oligopeptic	N
450	BP	455.3248	1	25.64467	182157.9	454.3176	tri/oligopeptic	N
451	BP	230.1322	2	1.959117	102921.1	458.2498	tri/oligopeptic	N
452	BP	462.2202	1	2.179992	135761.7	461.213	tri/oligopeptic	N
453	BP	471.2933	1	2.190508	140971.5	470.286	tri/oligopeptic	N
454	BP	236.1504	2	2.201208	203556.6	470.2862	tri/oligopeptic	N
455	BP	472.2782	1	15.58155	288057.5	471.2709	tri/oligopeptic	N
456	BP	474.2403	1	19.4263	162394	473.233	tri/oligopeptic	N
457	BP	479.2151	1	2.676858	475729.1	478.2078	tri/oligopeptic	N
458	BP	486.2569	1	13.51828	281074.5	485.2497	tri/oligopeptic	N
459	BP	486.2938	1	16.25883	407640.2	485.2865	tri/oligopeptic	N
460	BP	487.2892	1	12.74992	201596	486.2819	tri/oligopeptic	N
461	BP	488.2734	1	13.92928	211040.2	487.2661	tri/oligopeptic	N
462	BP	249.6745	2	16.84591	190639.2	497.3345	tri/oligopeptic	N
463	BP	504.2668	1	2.190508	167640.1	503.2595	tri/oligopeptic	N
464	BP	253.6528	2	2.190508	187111.1	505.2911	tri/oligopeptic	N
465	BP	255.1642	2	1.389167	256353.9	508.3139	tri/oligopeptic	N
466	BP	522.2577	1	17.08049	108857.1	521.2504	tri/oligopeptic	N
467	BP	261.6508	2	17.34193	443166.2	521.2871	tri/oligopeptic	N
468	BP	530.2996	1	26.96137	137845.2	529.2924	tri/oligopeptic	N
469	BP	531.2617	1	18.36313	155544.9	530.2544	tri/oligopeptic	N
470	BP	543.2779	1	2.190508	157185	542.2706	tri/oligopeptic	N
471	BP	544.2641	1	15.62132	428683.4	543.2568	tri/oligopeptic	N
472	BP	545.2578	1	2.607225	122451.4	544.2505	tri/oligopeptic	N
473	BP	553.3365	1	24.37516	2044654	552.3292	tri/oligopeptic	N
474	BP	279.1877	2	18.42107	147591.5	556.3609	tri/oligopeptic	N
475	BP	285.6853	2	13.73743	148085.4	569.356	tri/oligopeptic	N
476	BP	286.6461	2	15.20957	102092.4	571.2777	tri/oligopeptic	N

477	BP	573.2537	1	12.05268	246675.7	572.2464	tri/oligopeptic	N
478	BP	587.3057	1	12.07098	102389.9	586.2984	tri/oligopeptic	N
479	BP	592.3006	1	21.88585	300123.6	591.2933	tri/oligopeptic	N
480	BP	300.0945	2	1.959117	175317.6	598.1745	tri/oligopeptic	N
481	BP	600.3359	1	2.179992	178292.6	599.3287	tri/oligopeptic	N
482	BP	300.6718	2	2.190508	424340.8	599.329	tri/oligopeptic	N
483	BP	302.6694	2	2.049308	259618.9	603.3242	tri/oligopeptic	N
484	BP	304.177	2	2.389858	667040.6	606.3394	tri/oligopeptic	N
485	BP	623.3054	1	16.43152	413242.9	622.2981	oligopeptide	N
486	BP	316.6543	2	2.027433	163041	631.2941	oligopeptide	N
487	BP	328.1257	2	2.622217	136347.5	654.2369	oligopeptide	N
488	BP	330.1314	2	17.51987	145801.2	658.2483	oligopeptide	N
489	BP	342.1902	2	2.212083	279651.1	682.3658	oligopeptide	N
490	BP	349.7165	2	19.68709	129234.2	697.4185	oligopeptide	N
491	BP	716.3856	1	18.95381	407255.8	715.3783	oligopeptide	N
492	BP	387.1884	2	2.179992	167695.7	772.3623	oligopeptide	N
493	BP	395.1865	2	14.72253	103120.1	788.3585	oligopeptide	N
494	BP	798.3708	1	24.10095	106066.9	797.3635	oligopeptide	N
495	BP	412.2077	2	2.169517	111907.7	822.4009	oligopeptide	N
496	BP	413.7646	2	16.43152	308034.3	825.5146	oligopeptide	N
497	BP	419.2104	2	14.89067	389338.6	836.4063	oligopeptide	N
498	BP	460.6672	2	14.26049	109760.8	919.3198	oligopeptide	N
499	BP	463.747	2	16.09563	200564.7	925.4794	oligopeptide	N
500	BP	478.7759	2	16.53578	265114.6	955.5373	oligopeptide	N
501	BP	325.4923	3	15.3074	115392.1	973.455	oligopeptide	N
502	BP	338.8432	3	13.47607	331977.2	1013.508	oligopeptide	N
503	BP	510.2557	2	16.24487	137621.1	1018.497	oligopeptide	N
504	BP	520.2896	2	21.83461	184232.1	1038.565	oligopeptide	N
505	BP	542.7745	2	15.65519	106576.6	1083.534	oligopeptide	N
506	BP	684.9277	2	1.608117	120038.9	1367.841	oligopeptide	N
507	BP	718.9215	2	1.608117	119749.2	1435.828	oligopeptide	N
508	BP	752.9154	2	1.608117	102845	1503.816	oligopeptide	N
509	BP	786.9083	2	1.608117	102210.9	1571.802	oligopeptide	N
510	BP	820.903	2	1.596683	101779	1639.791	oligopeptide	N
511	BP	922.802	2	1.608117	216151.5	1843.589	oligopeptide	N
512	BP	1058.777	2	1.608117	110486.7	2115.54	oligopeptide	N
ID	Protein	mz	charge	retentiont	raw.abund	mass	type	Match
15	CH	246.1453	1	2.173233	192720.5	245.138	di/tripeptide	Y
22	CH	247.1296	1	4.710933	145785	246.1223	di/tripeptide	Y
24	CH	250.1784	1	34.8638	719351.8	249.1711	di/tripeptide	Y
25	CH	250.1784	1	37.26694	206849.7	249.1711	di/tripeptide	Y
30	CH	260.161	1	2.173233	276150.4	259.1538	di/tripeptide	Y
31	CH	260.1611	1	2.509258	348136.1	259.1539	di/tripeptide	Y
59	CH	304.151	1	2.19435	360622.1	303.1437	tri/oligopeptic	Y
64	CH	318.1668	1	2.2156	107753	317.1595	tri/oligopeptic	Y
72	CH	331.1619	1	2.119542	147552.8	330.1546	tri/oligopeptic	Y
112	CH	415.2123	1	36.47483	250209.1	414.2051	tri/oligopeptic	Y
133	CH	461.301	1	37.42613	101667.5	460.2937	tri/oligopeptic	Y
141	CH	479.3109	1	36.99213	156959.8	478.3036	tri/oligopeptic	Y
156	CH	515.292	1	37.11854	103130.9	514.2847	tri/oligopeptic	Y

161	CH	264.1752	2	35.28911	107658.7	526.3358	tri/oligopeptic	Y
165	CH	531.0146	1	1.581025	183538.5	530.0074	tri/oligopeptic	Y
174	CH	274.1681	2	35.93957	110090	546.3217	tri/oligopeptic	Y
190	CH	584.3056	1	2.2263	359131	583.2983	tri/oligopeptic	Y
194	CH	599.0026	1	1.581025	162461.9	597.9953	tri/oligopeptic	Y
218	CH	666.9901	1	1.593725	162240	665.9828	oligopeptide	Y
240	CH	734.9776	1	1.581025	142238.8	733.9704	oligopeptide	Y
258	CH	802.9652	1	1.569142	121084.7	801.958	oligopeptide	Y
297	CH	990.79	1	1.581025	208202.4	989.7827	oligopeptide	Y
338	CH	223.1084	1	10.46356	431339.6	222.1011	dipeptide	N
339	CH	226.9522	1	1.520492	147888.7	225.945	di/tripeptide	N
340	CH	226.9525	1	1.667042	427105.9	225.9452	di/tripeptide	N
341	CH	242.9266	1	1.667042	130247.8	241.9193	di/tripeptide	N
342	CH	244.1296	1	2.075283	730378.6	243.1223	di/tripeptide	N
343	CH	252.9631	1	10.01325	131336.5	251.9558	di/tripeptide	N
344	CH	262.1039	1	1.877517	172495	261.0966	di/tripeptide	N
345	CH	272.161	1	2.282258	522589.9	271.1538	di/tripeptide	N
346	CH	276.1195	1	1.877517	251645.4	275.1122	di/tripeptide	N
347	CH	286.1771	1	10.93833	370508	285.1698	di/tripeptide	N
348	CH	290.1352	1	2.19435	245617.3	289.128	di/tripeptide	N
349	CH	294.9405	1	1.667042	174204.7	293.9332	di/tripeptide	N
350	CH	301.1512	1	2.097825	269156.6	300.144	di/tripeptide	N
351	CH	302.1717	1	2.19435	139941.5	301.1644	tri/oligopeptic	N
352	CH	304.1513	1	8.269217	149441.1	303.144	tri/oligopeptic	N
353	CH	315.1669	1	2.19435	572662.5	314.1596	tri/oligopeptic	N
354	CH	317.1462	1	1.855583	118774.5	316.139	tri/oligopeptic	N
355	CH	317.1827	1	2.19435	127270.9	316.1754	tri/oligopeptic	N
356	CH	318.1302	1	1.918883	129756.1	317.123	tri/oligopeptic	N
357	CH	328.2343	1	15.48488	112942.9	327.227	tri/oligopeptic	N
358	CH	329.1941	1	1.7503	153476.7	328.1868	tri/oligopeptic	N
359	CH	331.1986	1	2.663867	158001.9	330.1913	tri/oligopeptic	N
360	CH	343.1987	1	2.259725	742589.9	342.1914	tri/oligopeptic	N
361	CH	343.1989	1	13.68614	109833.7	342.1917	tri/oligopeptic	N
362	CH	345.1777	1	2.19435	383970.5	344.1704	tri/oligopeptic	N
363	CH	357.2146	1	12.53406	250950.2	356.2073	tri/oligopeptic	N
364	CH	359.157	1	2.19435	141461.4	358.1497	tri/oligopeptic	N
365	CH	359.1933	1	2.534867	1979714	358.1861	tri/oligopeptic	N
366	CH	374.2044	1	2.582608	130314.9	373.1971	tri/oligopeptic	N
367	CH	375.1518	1	1.907467	150953.7	374.1445	tri/oligopeptic	N
368	CH	385.2461	1	17.78398	211270.1	384.2388	tri/oligopeptic	N
369	CH	388.1838	1	2.183825	355345.2	387.1765	tri/oligopeptic	N
370	CH	393.1783	1	12.84225	187525.3	392.171	tri/oligopeptic	N
371	CH	393.181	1	2.19435	621497.5	392.1737	tri/oligopeptic	N
372	CH	400.2202	1	2.4053	158853.8	399.2129	tri/oligopeptic	N
373	CH	401.2044	1	8.483533	114881.4	400.1971	tri/oligopeptic	N
374	CH	405.2617	1	37.28453	142718.3	404.2544	tri/oligopeptic	N
375	CH	413.2673	1	36.71656	978885.2	412.26	tri/oligopeptic	N
376	CH	414.2357	1	2.552767	646775.2	413.2284	tri/oligopeptic	N
377	CH	420.1733	1	2.097825	373386.6	419.166	tri/oligopeptic	N
378	CH	430.194	1	2.19435	756565.4	429.1867	tri/oligopeptic	N

379	CH	430.231	1	2.978375	419766.5	429.2237	tri/oligopeptic	N
380	CH	430.2311	1	13.83522	887748.8	429.2238	tri/oligopeptic	N
381	CH	432.1732	1	2.009375	457393.8	431.1659	tri/oligopeptic	N
382	CH	436.3438	1	1.350842	256414.5	435.3365	tri/oligopeptic	N
383	CH	443.2256	1	2.19435	273234.1	442.2183	tri/oligopeptic	N
384	CH	446.1888	1	2.020658	133835.1	445.1816	tri/oligopeptic	N
385	CH	446.189	1	2.19435	277523.6	445.1817	tri/oligopeptic	N
386	CH	446.2258	1	2.789092	776291.4	445.2186	tri/oligopeptic	N
387	CH	449.2878	1	37.23838	106847.8	448.2806	tri/oligopeptic	N
388	CH	450.1997	1	13.63806	355891	449.1924	tri/oligopeptic	N
389	CH	458.2255	1	2.552767	119466.6	457.2182	tri/oligopeptic	N
390	CH	458.2624	1	15.79448	610472.2	457.2552	tri/oligopeptic	N
391	CH	458.2624	1	19.21448	551908.1	457.2552	tri/oligopeptic	N
392	CH	460.2415	1	14.50872	1148174	459.2342	tri/oligopeptic	N
393	CH	462.1838	1	2.031942	211890.4	461.1765	tri/oligopeptic	N
394	CH	464.2156	1	16.85857	500541.3	463.2083	tri/oligopeptic	N
395	CH	471.2572	1	2.2156	155177.1	470.2499	tri/oligopeptic	N
396	CH	471.2576	1	3.840367	185450.4	470.2503	tri/oligopeptic	N
397	CH	471.2576	1	9.174592	232146	470.2504	tri/oligopeptic	N
398	CH	472.2416	1	15.9233	270997.8	471.2343	tri/oligopeptic	N
399	CH	473.236	1	2.19435	294020.6	472.2287	tri/oligopeptic	N
400	CH	238.6182	2	2.009375	298913.2	475.2219	tri/oligopeptic	N
401	CH	485.2363	1	2.19435	486540.5	484.229	tri/oligopeptic	N
402	CH	487.2154	1	2.19435	177247.1	486.2082	tri/oligopeptic	N
403	CH	488.2363	1	13.10863	1719919	487.229	tri/oligopeptic	N
404	CH	488.2367	1	12.19323	566408.7	487.2294	tri/oligopeptic	N
405	CH	495.2572	1	2.41845	294763.8	494.2499	tri/oligopeptic	N
406	CH	499.2521	1	2.19435	265676.6	498.2448	tri/oligopeptic	N
407	CH	499.2884	1	2.489342	313494.7	498.2812	tri/oligopeptic	N
408	CH	500.273	1	12.52728	197977.2	499.2657	tri/oligopeptic	N
409	CH	501.2311	1	2.19435	473175.8	500.2238	tri/oligopeptic	N
410	CH	501.2681	1	13.67786	148357.1	500.2608	tri/oligopeptic	N
411	CH	501.2681	1	13.3276	123584.1	500.2609	tri/oligopeptic	N
412	CH	501.2683	1	11.84976	109217.7	500.261	tri/oligopeptic	N
413	CH	511.2519	1	2.19435	104664.7	510.2446	tri/oligopeptic	N
414	CH	257.1614	2	2.19435	135827.8	512.3082	tri/oligopeptic	N
415	CH	516.242	1	2.19435	521926.4	515.2347	tri/oligopeptic	N
416	CH	517.226	1	2.19435	187104.1	516.2188	tri/oligopeptic	N
417	CH	519.2053	1	2.151417	310570.1	518.198	tri/oligopeptic	N
418	CH	522.1873	1	2.19435	309975.1	521.18	tri/oligopeptic	N
419	CH	522.2214	1	16.36224	944899.5	521.2141	tri/oligopeptic	N
420	CH	527.2839	1	15.90679	682689.2	526.2767	tri/oligopeptic	N
421	CH	264.1458	2	15.89866	187400.8	526.277	tri/oligopeptic	N
422	CH	264.6612	2	2.19435	183872.6	527.3079	tri/oligopeptic	N
423	CH	529.2632	1	11.16034	100220.2	528.256	tri/oligopeptic	N
424	CH	529.2996	1	18.52648	216362.5	528.2924	tri/oligopeptic	N
425	CH	529.2999	1	20.04718	313331.8	528.2926	tri/oligopeptic	N
426	CH	531.2416	1	2.19435	390523.3	530.2344	tri/oligopeptic	N
427	CH	531.2787	1	14.02315	368265	530.2714	tri/oligopeptic	N
428	CH	535.2527	1	16.5157	577423.4	534.2454	tri/oligopeptic	N

429	CH	537.3049	1	18.63977	167008.2	536.2976	tri/oligopeptic	N
430	CH	539.3198	1	20.53473	1368667	538.3125	tri/oligopeptic	N
431	CH	542.2575	1	2.19435	187149.5	541.2502	tri/oligopeptic	N
432	CH	543.2418	1	2.19435	714348.8	542.2345	tri/oligopeptic	N
433	CH	272.6405	2	1.975583	451345.6	543.2664	tri/oligopeptic	N
434	CH	555.3158	1	18.75917	218094.3	554.3085	tri/oligopeptic	N
435	CH	556.3213	1	2.183825	113748.2	555.314	tri/oligopeptic	N
436	CH	278.6644	2	2.19435	440759.6	555.3142	tri/oligopeptic	N
437	CH	557.2954	1	20.14087	121956.4	556.2881	tri/oligopeptic	N
438	CH	557.3318	1	18.81414	288308.9	556.3245	tri/oligopeptic	N
439	CH	559.3109	1	18.91823	167669.4	558.3036	tri/oligopeptic	N
440	CH	568.2737	1	2.19435	333537.7	567.2664	tri/oligopeptic	N
441	CH	285.6539	2	2.19435	337252.2	569.2933	tri/oligopeptic	N
442	CH	574.2478	1	2.19435	524010.5	573.2405	tri/oligopeptic	N
443	CH	579.2431	1	15.93778	205410.2	578.2358	tri/oligopeptic	N
444	CH	579.2453	1	2.19435	182733.9	578.238	tri/oligopeptic	N
445	CH	582.3278	1	17.23125	108949.4	581.3205	tri/oligopeptic	N
446	CH	292.6743	2	2.2263	148248.7	583.3341	tri/oligopeptic	N
447	CH	586.2845	1	2.183825	266234	585.2772	tri/oligopeptic	N
448	CH	588.2645	1	2.19435	224927.6	587.2572	tri/oligopeptic	N
449	CH	295.1605	2	2.19435	158698.1	588.3065	tri/oligopeptic	N
450	CH	592.2746	1	16.24601	669490.9	591.2674	tri/oligopeptic	N
451	CH	596.3428	1	19.58732	126738.6	595.3355	tri/oligopeptic	N
452	CH	299.1721	2	2.19435	179496.5	596.3296	tri/oligopeptic	N
453	CH	598.3218	1	15.11926	340932.4	597.3145	tri/oligopeptic	N
454	CH	599.2797	1	2.183825	113623.1	598.2724	tri/oligopeptic	N
455	CH	600.2629	1	2.183825	146870.6	599.2557	tri/oligopeptic	N
456	CH	600.3375	1	19.33392	110860.1	599.3303	tri/oligopeptic	N
457	CH	602.2801	1	13.31931	131868.2	601.2728	tri/oligopeptic	N
458	CH	602.2802	1	12.59656	175580.5	601.273	tri/oligopeptic	N
459	CH	301.6696	2	16.15702	215899	601.3245	tri/oligopeptic	N
460	CH	608.342	1	18.576	432048.9	607.3348	tri/oligopeptic	N
461	CH	610.2844	1	2.19435	579525	609.2771	tri/oligopeptic	N
462	CH	611.3166	1	2.645558	124350.6	610.3093	tri/oligopeptic	N
463	CH	612.3005	1	2.205033	270210.1	611.2932	tri/oligopeptic	N
464	CH	612.337	1	18.56745	343667.9	611.3298	tri/oligopeptic	N
465	CH	612.3376	1	17.97792	142989.4	611.3303	tri/oligopeptic	N
466	CH	307.6489	2	2.141275	183514.6	613.2833	oligopeptide	N
467	CH	614.3534	1	20.22931	111193.9	613.3461	oligopeptide	N
468	CH	615.3118	1	13.20163	238197.6	614.3045	oligopeptide	N
469	CH	308.62	2	16.24601	144086.5	615.2254	oligopeptide	N
470	CH	616.2955	1	13.79248	152923.4	615.2882	oligopeptide	N
471	CH	618.2743	1	2.19435	118047.9	617.267	oligopeptide	N
472	CH	624.3376	1	17.49828	109342.2	623.3303	oligopeptide	N
473	CH	629.2901	1	2.183825	296983.7	628.2829	oligopeptide	N
474	CH	629.2905	1	2.552767	157274.6	628.2832	oligopeptide	N
475	CH	315.1489	2	2.552767	143646.1	628.2833	oligopeptide	N
476	CH	630.2721	1	2.19435	141897	629.2648	oligopeptide	N
477	CH	634.2853	1	16.3534	103200.1	633.278	oligopeptide	N
478	CH	317.6646	2	13.10863	347925.4	633.3146	oligopeptide	N

479	CH	321.1726	2	2.19435	1140137	640.3307	oligopeptide	N
480	CH	641.3387	1	2.19435	233258.1	640.3314	oligopeptide	N
481	CH	644.3276	1	17.28434	204689.5	643.3203	oligopeptide	N
482	CH	644.329	1	20.51143	130085.6	643.3217	oligopeptide	N
483	CH	655.3433	1	16.50208	198194.6	654.336	oligopeptide	N
484	CH	329.1638	2	2.183825	148471.1	656.3131	oligopeptide	N
485	CH	329.1827	2	2.19435	217249.3	656.3509	oligopeptide	N
486	CH	669.3215	1	2.183825	173620.1	668.3143	oligopeptide	N
487	CH	339.6654	2	14.49849	246522	677.3162	oligopeptide	N
488	CH	342.1902	2	2.183825	113788.4	682.3658	oligopeptide	N
489	CH	683.3748	1	18.90156	171739	682.3676	oligopeptide	N
490	CH	342.1913	2	18.89199	111561.2	682.368	oligopeptide	N
491	CH	686.3749	1	19.66499	165073.1	685.3676	oligopeptide	N
492	CH	687.2952	1	2.183825	105754.2	686.2879	oligopeptide	N
493	CH	689.275	1	2.19435	141832	688.2677	oligopeptide	N
494	CH	701.3115	1	2.19435	109879.3	700.3042	oligopeptide	N
495	CH	352.6651	2	2.054608	155487.1	703.3156	oligopeptide	N
496	CH	705.3227	1	18.54459	781857.2	704.3155	oligopeptide	N
497	CH	353.1652	2	18.55301	200011	704.3159	oligopeptide	N
498	CH	358.6876	2	19.41008	110945.1	715.3606	oligopeptide	N
499	CH	360.689	2	15.27584	114598.4	719.3634	oligopeptide	N
500	CH	721.3177	1	15.98494	179717.7	720.3105	oligopeptide	N
501	CH	362.7095	2	2.205033	333002.6	723.4045	oligopeptide	N
502	CH	365.1441	2	18.54459	175315.7	728.2736	oligopeptide	N
503	CH	366.2096	2	19.28442	341454.4	730.4046	oligopeptide	N
504	CH	735.3339	1	21.19383	246976.8	734.3266	oligopeptide	N
505	CH	736.3649	1	13.20163	146647.9	735.3577	oligopeptide	N
506	CH	368.6863	2	13.16188	243450.7	735.358	oligopeptide	N
507	CH	744.3553	1	14.19074	128410	743.3481	oligopeptide	N
508	CH	755.3598	1	16.80773	211687.3	754.3526	oligopeptide	N
509	CH	378.1837	2	16.78675	475024.9	754.3528	oligopeptide	N
510	CH	378.1885	2	2.020658	116676.3	754.3625	oligopeptide	N
511	CH	756.355	1	12.43064	103233.6	755.3477	oligopeptide	N
512	CH	378.7051	2	12.63975	170538.2	755.3955	oligopeptide	N
513	CH	764.4334	1	22.33383	176564.1	763.4261	oligopeptide	N
514	CH	384.207	2	2.19435	519927.3	766.3994	oligopeptide	N
515	CH	767.4067	1	2.19435	105256.9	766.3994	oligopeptide	N
516	CH	385.1962	2	2.19435	271912	768.3778	oligopeptide	N
517	CH	387.1884	2	2.151417	272091.9	772.3623	oligopeptide	N
518	CH	390.1626	2	16.76308	108439.8	778.3106	oligopeptide	N
519	CH	390.2125	2	2.183825	292430.9	778.4105	oligopeptide	N
520	CH	391.2023	2	2.19435	379589.5	780.39	oligopeptide	N
521	CH	392.2229	2	13.89069	1723643	782.4312	oligopeptide	N
522	CH	398.2104	2	2.19435	202081.9	794.4062	oligopeptide	N
523	CH	797.3808	1	2.2156	108466.9	796.3735	oligopeptide	N
524	CH	404.1724	2	17.50731	156210.7	806.3302	oligopeptide	N
525	CH	405.2129	2	16.8015	125147.4	808.4112	oligopeptide	N
526	CH	810.3648	1	2.248483	113621.5	809.3575	oligopeptide	N
527	CH	405.6861	2	2.350883	518761.9	809.3577	oligopeptide	N
528	CH	811.397	1	13.72504	187092.2	810.3897	oligopeptide	N

529	CH	406.2076	2	2.19435	417788.4	810.4006	oligopeptide	N
530	CH	411.2366	2	16.61754	243252.3	820.4586	oligopeptide	N
531	CH	414.2237	2	12.6025	113616.7	826.4328	oligopeptide	N
532	CH	417.6651	2	2.350883	178014.3	833.3155	oligopeptide	N
533	CH	418.7232	2	2.19435	716155.3	835.4318	oligopeptide	N
534	CH	419.2144	2	2.2156	117241.7	836.4143	oligopeptide	N
535	CH	424.2025	2	17.69002	115569	846.3905	oligopeptide	N
536	CH	427.2314	2	14.88533	1247744	852.4483	oligopeptide	N
537	CH	427.7103	2	2.19435	476992.5	853.406	oligopeptide	N
538	CH	439.7285	2	2.19435	212269.7	877.4424	oligopeptide	N
539	CH	440.2337	2	18.6336	213419.7	878.4528	oligopeptide	N
540	CH	441.7392	2	16.99826	145301.9	881.4639	oligopeptide	N
541	CH	448.2043	2	14.57429	161881.3	894.394	oligopeptide	N
542	CH	453.721	2	14.21893	176388.2	905.4274	oligopeptide	N
543	CH	458.2174	2	14.89539	116681.1	914.4202	oligopeptide	N
544	CH	462.2573	2	2.19435	115100.6	922.5	oligopeptide	N
545	CH	466.2316	2	22.88008	140673	930.4486	oligopeptide	N
546	CH	931.4559	1	22.86679	137522.3	930.4487	oligopeptide	N
547	CH	472.2289	2	2.205033	112778.6	942.4433	oligopeptide	N
548	CH	947.4505	1	21.39534	148314.1	946.4432	oligopeptide	N
549	CH	474.2289	2	21.41241	160573.5	946.4433	oligopeptide	N
550	CH	483.7364	2	2.205033	151617.6	965.4582	oligopeptide	N
551	CH	484.2475	2	16.50208	188629	966.4804	oligopeptide	N
552	CH	490.7396	2	17.98568	180272.6	979.4646	oligopeptide	N
553	CH	496.2662	2	19.67261	285733.1	990.5178	oligopeptide	N
554	CH	496.7759	2	21.38751	687699.7	991.5372	oligopeptide	N
555	CH	504.2631	2	18.61848	467836.8	1006.512	oligopeptide	N
556	CH	339.5059	3	21.38147	122766.3	1015.496	oligopeptide	N
557	CH	517.2712	2	19.86586	137550.9	1032.528	oligopeptide	N
558	CH	524.763	2	2.205033	177180.3	1047.512	oligopeptide	N
559	CH	524.7816	2	2.534867	227953.4	1047.549	oligopeptide	N
560	CH	524.7819	2	13.56627	185409.4	1047.549	oligopeptide	N
561	CH	526.2574	2	2.183825	411992.2	1050.5	oligopeptide	N
562	CH	528.7708	2	2.282258	102308.2	1055.527	oligopeptide	N
563	CH	538.2877	2	15.23566	135271.9	1074.561	oligopeptide	N
564	CH	538.774	2	18.80704	273179.7	1075.533	oligopeptide	N
565	CH	542.7745	2	15.65792	142697.5	1083.534	oligopeptide	N
566	CH	544.7738	2	18.55301	565250.1	1087.533	oligopeptide	N
567	CH	544.774	2	18.96904	232217.3	1087.533	oligopeptide	N
568	CH	546.7715	2	17.67602	591132.4	1091.528	oligopeptide	N
569	CH	552.2564	2	21.61837	327806.3	1102.498	oligopeptide	N
570	CH	553.2874	2	19.44127	128949.5	1104.56	oligopeptide	N
571	CH	553.2928	2	14.76489	997932	1104.571	oligopeptide	N
572	CH	553.785	2	15.47732	253368.9	1105.555	oligopeptide	N
573	CH	554.7695	2	18.33628	116911.4	1107.524	oligopeptide	N
574	CH	558.253	2	2.19435	292871.7	1114.491	oligopeptide	N
575	CH	577.787	2	15.98494	281444.9	1153.559	oligopeptide	N
576	CH	581.8169	2	18.90156	281946.9	1161.619	oligopeptide	N
577	CH	390.1738	3	2.19435	128946.9	1167.5	oligopeptide	N
578	CH	600.7829	2	24.44879	265575.8	1199.551	oligopeptide	N

579	CH	601.2986	2	19.85294	258730.6	1200.583	oligopeptide	N
580	CH	601.7906	2	20.28936	290048.6	1201.567	oligopeptide	N
581	CH	608.7804	2	23.55998	133776.6	1215.546	oligopeptide	N
582	CH	634.3295	2	19.86586	176545.5	1266.644	oligopeptide	N
583	CH	638.8331	2	24.10134	144711.2	1275.652	oligopeptide	N
584	CH	638.8384	2	18.99985	252228.4	1275.662	oligopeptide	N
585	CH	639.3306	2	19.55983	233092.8	1276.647	oligopeptide	N
586	CH	666.8323	2	2.205033	442765.4	1331.65	oligopeptide	N
587	CH	667.3249	2	14.59383	124226.6	1332.635	oligopeptide	N
588	CH	695.3443	2	16.45014	118178.2	1388.674	oligopeptide	N
589	CH	715.8759	2	19.96082	107797.6	1429.737	oligopeptide	N
590	CH	731.8551	2	23.3877	239104.8	1461.696	oligopeptide	N
591	CH	749.3736	2	22.9781	100094.5	1496.733	oligopeptide	N
592	CH	764.3584	2	16.61754	257337.9	1526.702	oligopeptide	N
593	CH	767.3737	2	23.94483	189770.8	1532.733	oligopeptide	N
594	CH	781.8979	2	22.81726	152390.1	1561.781	oligopeptide	N
595	CH	843.3949	2	18.576	136310.5	1684.775	oligopeptide	N
596	CH	600.6066	3	15.31625	100602.6	1798.798	oligopeptide	N
597	CH	922.802	2	1.593725	253244.3	1843.589	oligopeptide	N
598	CH	1058.777	2	1.593725	148045.5	2115.54	oligopeptide	N
ID	Protein	mz	charge	retentiont	raw.abund	mass	type	Match
2	LM	231.171	1	5.400792	790372.3	230.1637	di/tripeptide	Y
3	LM	231.171	1	9.743442	481015.2	230.1637	di/tripeptide	Y
4	LM	231.1711	1	12.08171	762393.3	230.1638	di/tripeptide	Y
5	LM	233.1499	1	2.187992	825371.5	232.1426	di/tripeptide	Y
6	LM	233.1501	1	2.806692	282953.3	232.1428	di/tripeptide	Y
7	LM	239.1029	1	2.187992	392966.5	238.0956	di/tripeptide	Y
8	LM	239.103	1	2.447792	597528.1	238.0958	di/tripeptide	Y
11	LM	245.1867	1	16.43414	2109532	244.1794	di/tripeptide	Y
12	LM	245.1868	1	15.91003	330362.2	244.1795	di/tripeptide	Y
13	LM	245.1867	1	14.55766	430262.7	244.1794	di/tripeptide	Y
14	LM	245.1868	1	17.69159	349448	244.1795	di/tripeptide	Y
15	LM	246.1452	1	2.187992	674200.9	245.1379	di/tripeptide	Y
15	LM	246.1452	1	1.959917	251259.4	245.1379	di/tripeptide	Y
16	LM	246.1454	1	2.66985	340039.6	245.1381	di/tripeptide	Y
17	LM	246.1455	1	3.434625	647613.3	245.1382	di/tripeptide	Y
20	LM	247.1292	1	2.187992	962733.7	246.1219	di/tripeptide	Y
22	LM	247.1296	1	4.767342	934677.6	246.1223	di/tripeptide	Y
23	LM	249.1274	1	2.822408	320545.5	248.1201	di/tripeptide	Y
24	LM	250.1783	1	34.86093	708119.4	249.1711	di/tripeptide	Y
25	LM	250.1784	1	37.25158	203109.1	249.1711	di/tripeptide	Y
27	LM	253.1187	1	2.198958	777943.1	252.1114	di/tripeptide	Y
27	LM	253.1187	1	2.513058	721125.8	252.1115	di/tripeptide	Y
28	LM	253.119	1	9.369508	325801.9	252.1117	di/tripeptide	Y
30	LM	260.1609	1	2.198958	900233	259.1536	di/tripeptide	Y
31	LM	260.1611	1	2.679042	401502.1	259.1539	di/tripeptide	Y
33	LM	260.1973	1	2.187992	177406.3	259.19	di/tripeptide	Y
33	LM	260.1973	1	1.815142	143541.9	259.19	di/tripeptide	Y
34	LM	261.1449	1	2.198958	877421.8	260.1377	di/tripeptide	Y
35	LM	261.1453	1	3.349892	369742.4	260.138	di/tripeptide	Y

36	LM	261.1453	1	5.518942	689477.6	260.138	di/tripeptide	Y
38	LM	263.1432	1	11.57159	428758.1	262.1359	di/tripeptide	Y
40	LM	265.1556	1	16.28167	745277.5	264.1484	di/tripeptide	Y
41	LM	267.1345	1	2.424883	476588.9	266.1272	di/tripeptide	Y
42	LM	269.1612	1	2.187992	541533.6	268.1539	di/tripeptide	Y
44	LM	277.1035	1	1.863258	124110.4	276.0962	di/tripeptide	Y
45	LM	281.1138	1	2.586833	381695.1	280.1065	di/tripeptide	Y
46	LM	281.1505	1	7.14775	515517	280.1432	di/tripeptide	Y
50	LM	294.1454	1	2.345083	345050.9	293.1381	di/tripeptide	Y
51	LM	295.1298	1	3.385825	716262.6	294.1225	di/tripeptide	Y
52	LM	295.1661	1	16.43414	927847.8	294.1588	di/tripeptide	Y
57	LM	302.2084	1	19.87893	491743	301.2011	tri/oligopeptic	Y
58	LM	303.1669	1	2.198958	164850.2	302.1596	tri/oligopeptic	Y
58	LM	303.1673	1	2.649925	167910.7	302.16	tri/oligopeptic	Y
59	LM	304.1509	1	2.210133	388629.5	303.1436	tri/oligopeptic	Y
63	LM	318.1669	1	2.66985	165596.4	317.1596	tri/oligopeptic	Y
64	LM	318.1666	1	2.210133	516146.7	317.1594	tri/oligopeptic	Y
65	LM	318.167	1	9.162492	467533.8	317.1597	tri/oligopeptic	Y
66	LM	318.1826	1	22.57528	349225.5	317.1753	tri/oligopeptic	Y
66	LM	318.1826	1	22.90584	120259.8	317.1753	tri/oligopeptic	Y
73	LM	331.1662	1	2.85575	156018.8	330.1589	tri/oligopeptic	Y
75	LM	332.1825	1	2.598342	215947.3	331.1752	tri/oligopeptic	Y
76	LM	332.2191	1	3.113358	708843.7	331.2118	tri/oligopeptic	Y
79	LM	333.1569	1	11.09138	125614.9	332.1496	tri/oligopeptic	Y
80	LM	334.1615	1	2.198958	239286.5	333.1542	tri/oligopeptic	Y
92	LM	360.2139	1	2.791033	161325.4	359.2066	tri/oligopeptic	Y
94	LM	363.1558	1	2.187992	215902.2	362.1486	tri/oligopeptic	Y
100	LM	376.1722	1	2.210133	105742.6	375.1649	tri/oligopeptic	Y
106	LM	395.0398	1	1.598842	146851.5	394.0325	tri/oligopeptic	Y
112	LM	415.2123	1	36.4673	258007.9	414.205	tri/oligopeptic	Y
118	LM	429.2723	1	18.85342	170343.8	428.2651	tri/oligopeptic	Y
134	LM	463.0273	1	1.621117	198866	462.02	tri/oligopeptic	Y
141	LM	479.3109	1	37.16047	159411.4	478.3036	tri/oligopeptic	Y
144	LM	488.2522	1	19.14933	138432.2	487.2449	tri/oligopeptic	Y
151	LM	500.2888	1	25.19285	139476.2	499.2815	tri/oligopeptic	Y
156	LM	515.292	1	37.08576	115294.3	514.2847	tri/oligopeptic	Y
161	LM	264.1751	2	35.26698	108830.5	526.3357	tri/oligopeptic	Y
165	LM	531.0146	1	1.610017	248139.9	530.0073	tri/oligopeptic	Y
166	LM	531.3877	1	37.84633	100068	530.3804	tri/oligopeptic	Y
174	LM	274.1681	2	35.92648	111895.7	546.3216	tri/oligopeptic	Y
194	LM	599.0024	1	1.610017	226038.2	597.9952	tri/oligopeptic	Y
218	LM	666.99	1	1.610017	219741.3	665.9827	oligopeptide	Y
240	LM	734.9775	1	1.610017	196535.2	733.9702	oligopeptide	Y
258	LM	802.9651	1	1.610017	173428.3	801.9578	oligopeptide	Y
276	LM	870.9528	1	1.598842	118476.4	869.9455	oligopeptide	Y
296	LM	974.8153	1	1.621117	266358	973.808	oligopeptide	Y
297	LM	990.7896	1	1.621117	202825.3	989.7823	oligopeptide	Y
302	LM	1042.802	1	1.621117	156478.3	1041.795	oligopeptide	Y
313	LM	582.9462	2	1.610017	101013.2	1163.878	oligopeptide	Y
316	LM	616.9401	2	1.621117	103805.2	1231.866	oligopeptide	Y

318	LM	624.9268	2	1.610017	106833.9	1247.839	oligopeptide	Y
329	LM	692.9145	2	1.621117	103839.1	1383.814	oligopeptide	Y
338	LM	223.1083	1	5.106542	483354.7	222.1011	dipeptide	N
339	LM	223.1084	1	10.47817	431659.3	222.1011	dipeptide	N
340	LM	229.1554	1	5.049692	153906.4	228.1481	di/tripeptide	N
341	LM	231.171	1	9.006225	291605.9	230.1637	di/tripeptide	N
342	LM	233.1135	1	2.198958	120423.1	232.1062	di/tripeptide	N
343	LM	233.1136	1	2.005575	198786.9	232.1063	di/tripeptide	N
344	LM	233.1503	1	4.133825	378099.2	232.143	di/tripeptide	N
345	LM	237.124	1	11.23058	456998.2	236.1167	di/tripeptide	N
346	LM	237.124	1	4.676475	235116.5	236.1168	di/tripeptide	N
347	LM	246.1456	1	5.425042	235592.3	245.1383	di/tripeptide	N
348	LM	252.963	1	8.455267	138554	251.9558	di/tripeptide	N
349	LM	258.1108	1	1.724358	134427.6	257.1036	di/tripeptide	N
350	LM	262.1196	1	17.00185	100435.3	261.1124	di/tripeptide	N
351	LM	263.1399	1	14.47133	166826.9	262.1326	di/tripeptide	N
352	LM	263.1433	1	13.14811	178151.8	262.136	di/tripeptide	N
353	LM	265.1556	1	14.76838	368743.1	264.1483	di/tripeptide	N
354	LM	269.0884	1	2.198958	186821.6	268.0811	di/tripeptide	N
355	LM	269.1135	1	2.187992	386596	268.1062	di/tripeptide	N
356	LM	269.1137	1	2.345083	382219.4	268.1065	di/tripeptide	N
357	LM	274.1401	1	2.187992	109126.7	273.1328	di/tripeptide	N
358	LM	274.1766	1	2.27715	178221.5	273.1693	di/tripeptide	N
359	LM	274.1767	1	2.501892	133789.1	273.1694	di/tripeptide	N
360	LM	276.1353	1	14.35843	164860.9	275.128	di/tripeptide	N
361	LM	276.1354	1	17.19731	115912.2	275.1281	di/tripeptide	N
362	LM	276.1559	1	2.198958	249873.7	275.1486	di/tripeptide	N
363	LM	276.1561	1	2.413558	148626.7	275.1488	di/tripeptide	N
364	LM	276.1563	1	5.971225	130143.3	275.1491	di/tripeptide	N
365	LM	277.1194	1	20.44814	296245.7	276.1121	di/tripeptide	N
366	LM	279.1348	1	9.507417	148050.9	278.1276	di/tripeptide	N
367	LM	279.1348	1	3.796183	265994.3	278.1276	di/tripeptide	N
368	LM	279.1713	1	19.91518	944712.8	278.164	di/tripeptide	N
369	LM	279.1714	1	19.00699	140545.5	278.1641	di/tripeptide	N
370	LM	279.1714	1	21.04403	229202.6	278.1641	di/tripeptide	N
371	LM	279.2325	1	37.01129	233685.7	278.2252	di/tripeptide	N
372	LM	280.1296	1	2.221225	220320.3	279.1223	di/tripeptide	N
373	LM	280.1302	1	10.05645	105074.3	279.1229	di/tripeptide	N
374	LM	281.1141	1	11.60508	401405.3	280.1068	di/tripeptide	N
375	LM	281.1505	1	6.1342	623305.1	280.1432	di/tripeptide	N
376	LM	283.1293	1	2.198958	277670.6	282.122	di/tripeptide	N
377	LM	286.1771	1	13.53491	103690	285.1699	di/tripeptide	N
378	LM	288.1557	1	2.17705	125772.7	287.1485	di/tripeptide	N
379	LM	288.1922	1	2.243617	206569.4	287.185	di/tripeptide	N
380	LM	288.1924	1	2.402158	122764.8	287.1851	di/tripeptide	N
381	LM	288.1926	1	9.516258	243417.8	287.1854	di/tripeptide	N
382	LM	288.1927	1	10.74696	116072.6	287.1854	di/tripeptide	N
383	LM	288.2035	1	1.9941	102758.4	287.1962	di/tripeptide	N
384	LM	290.1351	1	2.039967	114042.1	289.1278	di/tripeptide	N
385	LM	290.1715	1	2.198958	572393.6	289.1643	di/tripeptide	N

386	LM	292.1301	1	16.43414	110485.1	291.1228	di/tripeptide	N
387	LM	295.1299	1	12.67196	381692.4	294.1227	di/tripeptide	N
388	LM	295.1662	1	14.63307	409113.1	294.159	di/tripeptide	N
389	LM	295.1663	1	13.44093	918679.1	294.159	di/tripeptide	N
390	LM	295.2275	1	37.37008	124380.9	294.2202	di/tripeptide	N
391	LM	296.1246	1	2.198958	278559	295.1174	di/tripeptide	N
392	LM	296.1248	1	2.413558	128894.9	295.1176	di/tripeptide	N
393	LM	297.1086	1	2.198958	140735.9	296.1013	di/tripeptide	N
394	LM	297.1089	1	2.717092	383503.5	296.1016	di/tripeptide	N
395	LM	300.1928	1	4.074342	122548.7	299.1855	di/tripeptide	N
396	LM	300.1929	1	13.41257	380115.2	299.1856	di/tripeptide	N
397	LM	300.1929	1	12.08773	122686.8	299.1856	di/tripeptide	N
398	LM	300.1929	1	13.69647	188393.1	299.1857	di/tripeptide	N
399	LM	302.2084	1	11.18603	130543	301.2012	tri/oligopeptic	N
400	LM	302.2084	1	13.14811	125786.2	301.2012	tri/oligopeptic	N
401	LM	302.2085	1	6.428992	163427.7	301.2012	tri/oligopeptic	N
402	LM	302.2086	1	15.83846	138480.4	301.2013	tri/oligopeptic	N
403	LM	304.1665	1	19.92085	484931.5	303.1592	tri/oligopeptic	N
404	LM	304.1872	1	2.198958	141633.7	303.1799	tri/oligopeptic	N
405	LM	304.1874	1	2.424883	104670.7	303.1801	tri/oligopeptic	N
406	LM	304.1877	1	11.40626	128839	303.1804	tri/oligopeptic	N
407	LM	306.1462	1	10.8124	103856.2	305.139	tri/oligopeptic	N
408	LM	306.1665	1	2.187992	174350.2	305.1592	tri/oligopeptic	N
409	LM	310.1402	1	2.210133	673439.5	309.133	tri/oligopeptic	N
410	LM	310.1404	1	2.447792	157134.1	309.1332	tri/oligopeptic	N
411	LM	310.1408	1	9.923342	119633.8	309.1335	tri/oligopeptic	N
412	LM	310.1767	1	1.97135	101008.2	309.1694	tri/oligopeptic	N
413	LM	311.1242	1	2.198958	363040.9	310.117	tri/oligopeptic	N
414	LM	311.1246	1	2.799667	310704.6	310.1173	tri/oligopeptic	N
415	LM	313.1227	1	11.82952	284120.4	312.1154	tri/oligopeptic	N
416	LM	314.2085	1	13.68121	242073.4	313.2012	tri/oligopeptic	N
417	LM	314.2085	1	13.03123	125922.9	313.2012	tri/oligopeptic	N
418	LM	316.1879	1	13.06332	139702.9	315.1807	tri/oligopeptic	N
419	LM	316.188	1	14.58649	176512	315.1807	tri/oligopeptic	N
420	LM	316.2243	1	14.84319	143387.7	315.2171	tri/oligopeptic	N
421	LM	316.2244	1	19.60503	194943.7	315.2171	tri/oligopeptic	N
422	LM	317.1825	1	2.198958	222620.1	316.1753	tri/oligopeptic	N
423	LM	317.1831	1	3.529967	212051.2	316.1758	tri/oligopeptic	N
424	LM	318.1671	1	5.5851	182424	317.1598	tri/oligopeptic	N
425	LM	318.2029	1	2.210133	225729	317.1956	tri/oligopeptic	N
426	LM	318.2035	1	8.1336	133853.8	317.1963	tri/oligopeptic	N
427	LM	318.2037	1	16.1012	107512.2	317.1964	tri/oligopeptic	N
428	LM	319.1414	1	16.83336	156950.2	318.1341	tri/oligopeptic	N
429	LM	320.1254	1	17.66704	116637.4	319.1181	tri/oligopeptic	N
430	LM	320.1458	1	2.039967	130254.6	319.1386	tri/oligopeptic	N
431	LM	320.1619	1	18.3665	194554.8	319.1546	tri/oligopeptic	N
432	LM	328.2244	1	16.88842	137668.4	327.2172	tri/oligopeptic	N
433	LM	328.2244	1	17.11261	189201	327.2172	tri/oligopeptic	N
434	LM	328.2245	1	17.80768	102801.6	327.2172	tri/oligopeptic	N
435	LM	329.1825	1	2.198958	627822.8	328.1752	tri/oligopeptic	N

436	LM	331.1982	1	2.198958	159919.4	330.191	tri/oligopeptic	N
437	LM	332.1828	1	11.64338	108197.6	331.1755	tri/oligopeptic	N
438	LM	333.1774	1	2.187992	382225.8	332.1701	tri/oligopeptic	N
439	LM	333.1778	1	2.658675	388816.9	332.1705	tri/oligopeptic	N
440	LM	334.141	1	13.77377	213315.8	333.1337	tri/oligopeptic	N
441	LM	334.162	1	5.175725	116668.8	333.1547	tri/oligopeptic	N
442	LM	334.1775	1	18.0527	204287.5	333.1702	tri/oligopeptic	N
443	LM	336.1566	1	12.89045	131672.7	335.1493	tri/oligopeptic	N
444	LM	338.1719	1	2.490767	106418.2	337.1646	tri/oligopeptic	N
445	LM	340.151	1	2.27715	108871.3	339.1438	tri/oligopeptic	N
446	LM	342.2403	1	21.30493	121678.6	341.233	tri/oligopeptic	N
447	LM	342.2403	1	20.75707	112204.1	341.233	tri/oligopeptic	N
448	LM	343.1985	1	2.221225	235806.6	342.1912	tri/oligopeptic	N
449	LM	345.1459	1	14.0599	112968.2	344.1386	tri/oligopeptic	N
450	LM	345.214	1	2.198958	108736	344.2068	tri/oligopeptic	N
451	LM	346.1808	1	14.51786	128588.1	345.1735	tri/oligopeptic	N
452	LM	346.1982	1	2.479592	135395.3	345.191	tri/oligopeptic	N
453	LM	346.2349	1	14.60074	132960.4	345.2276	tri/oligopeptic	N
454	LM	346.2351	1	13.57153	128729.3	345.2278	tri/oligopeptic	N
455	LM	348.1772	1	2.198958	392067.3	347.17	tri/oligopeptic	N
456	LM	350.1723	1	12.41783	397774.7	349.165	tri/oligopeptic	N
457	LM	352.1879	1	11.18603	112614.8	351.1807	tri/oligopeptic	N
458	LM	357.2141	1	2.288542	738446	356.2068	tri/oligopeptic	N
459	LM	357.2142	1	2.791033	2133665	356.2069	tri/oligopeptic	N
460	LM	357.2145	1	11.2235	194576.1	356.2072	tri/oligopeptic	N
461	LM	357.2146	1	12.91559	112820.5	356.2073	tri/oligopeptic	N
462	LM	358.296	1	36.28283	224752.3	357.2887	tri/oligopeptic	N
463	LM	359.2302	1	7.407167	157784.5	358.223	tri/oligopeptic	N
464	LM	359.2303	1	16.17923	126130.2	358.2231	tri/oligopeptic	N
465	LM	360.2145	1	20.46971	147907.7	359.2072	tri/oligopeptic	N
466	LM	361.1725	1	2.187992	334741.3	360.1652	tri/oligopeptic	N
467	LM	361.2088	1	2.210133	117349.3	360.2015	tri/oligopeptic	N
468	LM	362.193	1	2.27715	357634.7	361.1858	tri/oligopeptic	N
469	LM	362.209	1	21.35968	265200.1	361.2017	tri/oligopeptic	N
470	LM	364.066	1	2.210133	152598.2	363.0587	tri/oligopeptic	N
471	LM	364.1883	1	18.27964	107759.6	363.181	tri/oligopeptic	N
472	LM	371.2301	1	11.47663	599971.6	370.2229	tri/oligopeptic	N
473	LM	371.2301	1	14.64038	482636.6	370.2229	tri/oligopeptic	N
474	LM	371.2302	1	14.208	383515.1	370.2229	tri/oligopeptic	N
475	LM	371.2302	1	12.52073	486803.2	370.223	tri/oligopeptic	N
476	LM	373.2461	1	18.83558	161186.7	372.2389	tri/oligopeptic	N
477	LM	374.2039	1	2.187992	144360.8	373.1966	tri/oligopeptic	N
478	LM	377.2038	1	2.243617	114531.1	376.1965	tri/oligopeptic	N
479	LM	378.2039	1	15.12353	176720.4	377.1966	tri/oligopeptic	N
480	LM	385.2461	1	15.47318	121649.8	384.2388	tri/oligopeptic	N
481	LM	387.2247	1	2.775042	102517.1	386.2174	tri/oligopeptic	N
482	LM	389.2037	1	2.198958	171793.5	388.1965	tri/oligopeptic	N
483	LM	391.1999	1	14.98866	161478.2	390.1927	tri/oligopeptic	N
484	LM	396.1775	1	2.66985	200787.6	395.1702	tri/oligopeptic	N
485	LM	397.1726	1	2.345083	105775.7	396.1653	tri/oligopeptic	N

486	LM	399.2612	1	16.42042	139763.6	398.2539	tri/oligopeptic	N
487	LM	399.2617	1	16.76551	116559.9	398.2545	tri/oligopeptic	N
488	LM	400.2197	1	2.187992	153912.8	399.2124	tri/oligopeptic	N
489	LM	401.2408	1	10.94893	147305.7	400.2336	tri/oligopeptic	N
490	LM	402.1673	1	12.61625	105480.4	401.1601	tri/oligopeptic	N
491	LM	403.2567	1	16.91779	147257.6	402.2494	tri/oligopeptic	N
492	LM	403.2567	1	18.06443	187279.4	402.2494	tri/oligopeptic	N
493	LM	405.1987	1	2.187992	108081.6	404.1914	tri/oligopeptic	N
494	LM	408.1779	1	11.54782	155927	407.1706	tri/oligopeptic	N
495	LM	412.1723	1	2.311033	110393.8	411.165	tri/oligopeptic	N
496	LM	414.236	1	11.06041	173266.3	413.2287	tri/oligopeptic	N
497	LM	415.2202	1	14.49839	110830	414.2129	tri/oligopeptic	N
498	LM	419.1779	1	2.198958	102442.4	418.1706	tri/oligopeptic	N
499	LM	421.2095	1	14.79268	129399.4	420.2023	tri/oligopeptic	N
500	LM	421.2096	1	13.92144	130000.2	420.2024	tri/oligopeptic	N
501	LM	423.2356	1	2.187992	166871.9	422.2283	tri/oligopeptic	N
502	LM	426.204	1	23.88436	232113.6	425.1968	tri/oligopeptic	N
503	LM	428.2513	1	2.557658	678014	427.244	tri/oligopeptic	N
504	LM	428.2517	1	11.47663	231734.8	427.2444	tri/oligopeptic	N
505	LM	428.2518	1	13.63509	204905.2	427.2445	tri/oligopeptic	N
506	LM	436.3433	1	35.94393	457356.2	435.336	tri/oligopeptic	N
507	LM	436.3437	1	0.020917	262032	435.3364	tri/oligopeptic	N
508	LM	437.2045	1	12.55126	116842.6	436.1973	tri/oligopeptic	N
509	LM	439.268	1	14.29792	125572	438.2607	tri/oligopeptic	N
510	LM	439.2934	1	22.64638	190586.6	438.2861	tri/oligopeptic	N
511	LM	442.1989	1	17.61672	326654	441.1916	tri/oligopeptic	N
512	LM	442.2673	1	13.37642	334991.6	441.2601	tri/oligopeptic	N
513	LM	442.2674	1	14.4488	311248.1	441.2602	tri/oligopeptic	N
514	LM	442.2675	1	15.86203	123991.1	441.2602	tri/oligopeptic	N
515	LM	444.2461	1	2.221225	185431	443.2388	tri/oligopeptic	N
516	LM	446.2624	1	16.72344	199736.1	445.2552	tri/oligopeptic	N
517	LM	450.1626	1	2.187992	259521.9	449.1553	tri/oligopeptic	N
518	LM	456.2829	1	15.94793	575865.5	455.2756	tri/oligopeptic	N
519	LM	458.2623	1	11.12248	100197.8	457.2551	tri/oligopeptic	N
520	LM	463.2989	1	37.55269	106520.1	462.2916	tri/oligopeptic	N
521	LM	472.2781	1	16.56067	500892.6	471.2708	tri/oligopeptic	N
522	LM	478.231	1	11.19344	184813.5	477.2238	tri/oligopeptic	N
523	LM	491.2516	1	19.99671	320056.8	490.2443	tri/oligopeptic	N
524	LM	491.2519	1	24.47185	110392	490.2447	tri/oligopeptic	N
525	LM	492.2468	1	13.71196	227828.6	491.2395	tri/oligopeptic	N
526	LM	494.2726	1	2.198958	551168.6	493.2653	tri/oligopeptic	N
527	LM	247.64	2	2.187992	204242.6	493.2655	tri/oligopeptic	N
528	LM	496.2413	1	16.38859	148665.9	495.234	tri/oligopeptic	N
529	LM	498.902	1	1.610017	598312.4	497.8947	tri/oligopeptic	N
530	LM	502.2712	1	18.72871	135789.4	501.2639	tri/oligopeptic	N
531	LM	504.2835	1	21.433	134856.5	503.2762	tri/oligopeptic	N
532	LM	508.2892	1	11.17733	239454.9	507.282	tri/oligopeptic	N
533	LM	254.6484	2	11.2078	155440.3	507.2822	tri/oligopeptic	N
534	LM	257.6537	2	11.85296	109268.5	513.2928	tri/oligopeptic	N
535	LM	520.2784	1	18.28602	272917.5	519.2711	tri/oligopeptic	N

536	LM	526.3256	1	23.384	173721.8	525.3183	tri/oligopeptic	N
537	LM	554.332	1	15.341	164807.8	553.3247	tri/oligopeptic	N
538	LM	283.1586	2	2.198958	123969.8	564.3027	tri/oligopeptic	N
539	LM	566.3316	1	16.3722	143936.8	565.3243	tri/oligopeptic	N
540	LM	283.6694	2	16.38113	151513.6	565.3243	tri/oligopeptic	N
541	LM	595.2901	1	24.75063	128487.8	594.2828	tri/oligopeptic	N
542	LM	316.1875	2	2.379283	104989.3	630.3604	oligopeptide	N
543	LM	639.3738	1	19.38297	161934.5	638.3665	oligopeptide	N
544	LM	320.1907	2	19.36935	109235.1	638.3668	oligopeptide	N
545	LM	336.6725	2	15.77554	129132.9	671.3305	oligopeptide	N
546	LM	384.1789	2	17.32999	107188.3	766.3433	oligopeptide	N
547	LM	384.7172	2	2.557658	156029.5	767.4198	oligopeptide	N
548	LM	820.8202	1	1.632142	116464.2	819.8129	oligopeptide	N
549	LM	439.226	2	14.84319	207156.7	876.4375	oligopeptide	N
550	LM	460.6672	2	14.28904	111083.6	919.3199	oligopeptide	N
551	LM	475.7421	2	23.89188	170404.7	949.4696	oligopeptide	N
552	LM	1058.777	2	1.621117	139747.5	2115.539	oligopeptide	N
ID	Protein	mz	charge	retentiont	raw.abund	mass	type	Match
2	LMC2	231.171	1	5.308483	162965.6	230.1637	di/tripeptide	Y
5	LMC2	233.1499	1	2.178042	171408.1	232.1426	di/tripeptide	Y
8	LMC2	239.103	1	2.447683	121182.2	238.0958	di/tripeptide	Y
11	LMC2	245.1867	1	16.4214	109168.8	244.1794	di/tripeptide	Y
15	LMC2	246.1452	1	2.178042	182636.1	245.1379	di/tripeptide	Y
20	LMC2	247.1292	1	2.178042	414327.4	246.1219	di/tripeptide	Y
22	LMC2	247.1296	1	4.667183	218239.9	246.1223	di/tripeptide	Y
24	LMC2	250.1783	1	34.86174	753175.9	249.1711	di/tripeptide	Y
25	LMC2	250.1784	1	37.25502	204283.7	249.1711	di/tripeptide	Y
30	LMC2	260.1609	1	2.189583	161266.1	259.1536	di/tripeptide	Y
34	LMC2	261.1449	1	2.189583	331013.5	260.1377	di/tripeptide	Y
36	LMC2	261.1453	1	5.425975	114040.5	260.138	di/tripeptide	Y
44	LMC2	277.1035	1	1.8456	115215.8	276.0962	di/tripeptide	Y
45	LMC2	281.1138	1	2.592508	112078.4	280.1065	di/tripeptide	Y
51	LMC2	295.1298	1	3.259108	148779.1	294.1225	di/tripeptide	Y
57	LMC2	302.2084	1	19.87918	276345.1	301.2011	tri/oligopeptic	Y
59	LMC2	304.1509	1	2.201183	208868.4	303.1436	tri/oligopeptic	Y
64	LMC2	318.1666	1	2.201183	244948.6	317.1594	tri/oligopeptic	Y
65	LMC2	318.167	1	9.080292	216788.3	317.1597	tri/oligopeptic	Y
75	LMC2	332.1825	1	2.602717	133828.6	331.1752	tri/oligopeptic	Y
76	LMC2	332.2191	1	3.025442	104777.8	331.2118	tri/oligopeptic	Y
80	LMC2	334.1615	1	2.189583	113988.3	333.1542	tri/oligopeptic	Y
105	LMC2	390.188	1	2.340608	105600.9	389.1807	tri/oligopeptic	Y
106	LMC2	395.0398	1	1.577917	121743.3	394.0325	tri/oligopeptic	Y
112	LMC2	415.2123	1	36.46694	291695.3	414.205	tri/oligopeptic	Y
134	LMC2	463.0273	1	1.59865	151387.5	462.02	tri/oligopeptic	Y
141	LMC2	479.3109	1	37.15848	157109.3	478.3036	tri/oligopeptic	Y
156	LMC2	515.292	1	37.08083	114310.3	514.2847	tri/oligopeptic	Y
161	LMC2	264.1751	2	35.26117	112157.7	526.3357	tri/oligopeptic	Y
165	LMC2	531.0146	1	1.588292	179670.1	530.0073	tri/oligopeptic	Y
166	LMC2	531.3877	1	37.83429	100276.1	530.3804	tri/oligopeptic	Y
174	LMC2	274.1681	2	35.92845	113243.4	546.3216	tri/oligopeptic	Y

194	LMC2	599.0024	1	1.588292	162989.7	597.9952	tri/oligopeptic	Y
218	LMC2	666.99	1	1.588292	154789	665.9827	oligopeptide	Y
240	LMC2	734.9775	1	1.588292	130103.4	733.9702	oligopeptide	Y
296	LMC2	974.8153	1	1.59865	133878.8	973.808	oligopeptide	Y
297	LMC2	990.7896	1	1.59865	138053.7	989.7823	oligopeptide	Y
338	LMC2	223.1084	1	10.40951	106602.9	222.1011	dipeptide	N
339	LMC2	226.9526	1	1.650042	195448.4	225.9453	di/tripeptide	N
340	LMC2	229.1554	1	11.88037	238370.6	228.1482	di/tripeptide	N
341	LMC2	235.1654	1	1.7832	368201.7	234.1581	di/tripeptide	N
342	LMC2	252.963	1	8.379542	146697.2	251.9558	di/tripeptide	N
343	LMC2	261.1454	1	10.77103	140602.1	260.1381	di/tripeptide	N
344	LMC2	263.14	1	16.82774	194641.6	262.1328	di/tripeptide	N
345	LMC2	268.1408	1	2.189583	100466.3	267.1335	di/tripeptide	N
346	LMC2	269.0884	1	2.189583	293937.8	268.0811	di/tripeptide	N
347	LMC2	274.1299	1	2.189583	184041.3	273.1227	di/tripeptide	N
348	LMC2	279.1348	1	9.432442	301840.2	278.1276	di/tripeptide	N
349	LMC2	290.1351	1	2.028083	117307.3	289.1278	di/tripeptide	N
350	LMC2	290.1715	1	2.189583	149052	289.1643	di/tripeptide	N
351	LMC2	294.9405	1	1.650042	120607.9	293.9333	di/tripeptide	N
352	LMC2	295.0609	1	2.154708	118228.2	294.0536	di/tripeptide	N
353	LMC2	311.1242	1	2.189583	162549.5	310.117	tri/oligopeptic	N
354	LMC2	318.1203	1	2.189583	102168.1	317.113	tri/oligopeptic	N
355	LMC2	348.1772	1	2.189583	181931.6	347.17	tri/oligopeptic	N
356	LMC2	361.1725	1	2.178042	147074	360.1652	tri/oligopeptic	N
357	LMC2	362.193	1	2.272383	109095.4	361.1858	tri/oligopeptic	N
358	LMC2	406.1728	1	2.201183	128053	405.1656	tri/oligopeptic	N
359	LMC2	433.2464	1	22.54095	223442.7	432.2391	tri/oligopeptic	N
360	LMC2	436.3433	1	35.9459	450263.6	435.336	tri/oligopeptic	N
361	LMC2	436.3437	1	0.020983	248379.4	435.3364	tri/oligopeptic	N
362	LMC2	450.1626	1	2.178042	113922.9	449.1553	tri/oligopeptic	N
363	LMC2	498.902	1	1.588292	243353.3	497.8947	tri/oligopeptic	N
364	LMC2	498.9028	1	1.650042	126213.5	497.8955	tri/oligopeptic	N
365	LMC2	598.838	1	1.619058	108531.4	597.8308	tri/oligopeptic	N

ID	Protein	mz	charge	retentionti	raw.abund	mass	type	Match
2	TPP1	231.1712	1	4.993583	1087549	230.1639	di/tripeptide	Y
3	TPP1	231.1712	1	9.508383	1138185	230.1639	di/tripeptide	Y
4	TPP1	231.1712	1	11.98594	860501.2	230.1639	di/tripeptide	Y
5	TPP1	233.1502	1	2.194608	290728.5	232.1429	di/tripeptide	Y
6	TPP1	233.1504	1	2.719742	536815.3	232.1431	di/tripeptide	Y
8	TPP1	239.1034	1	2.397617	216850.3	238.0961	di/tripeptide	Y
11	TPP1	245.1868	1	16.41001	1196334	244.1795	di/tripeptide	Y
12	TPP1	245.1869	1	15.86598	221307.8	244.1796	di/tripeptide	Y
13	TPP1	245.1869	1	14.49528	1021074	244.1796	di/tripeptide	Y
14	TPP1	245.1868	1	17.66004	1634066	244.1796	di/tripeptide	Y
15	TPP1	246.1455	1	2.183042	620093.7	245.1382	di/tripeptide	Y
16	TPP1	246.1456	1	2.606783	533341.4	245.1384	di/tripeptide	Y
20	TPP1	247.1296	1	2.183042	312523.4	246.1223	di/tripeptide	Y
21	TPP1	247.1298	1	3.0391	322793.5	246.1225	di/tripeptide	Y
22	TPP1	247.1297	1	4.590683	499708.3	246.1224	di/tripeptide	Y
23	TPP1	249.1285	1	2.730717	239298.4	248.1213	di/tripeptide	Y

27	TPP1	253.119	1	2.27365	249564.1	252.1117	di/tripeptide	Y
27	TPP1	253.1191	1	2.455017	121967.1	252.1118	di/tripeptide	Y
28	TPP1	253.1191	1	9.2369	309478.6	252.1119	di/tripeptide	Y
30	TPP1	260.1613	1	2.194608	665058.6	259.154	di/tripeptide	Y
31	TPP1	260.1615	1	2.618408	141134.3	259.1542	di/tripeptide	Y
33	TPP1	260.1976	1	2.194608	314673.4	259.1904	di/tripeptide	Y
33	TPP1	260.1976	1	1.860925	130507.8	259.1904	di/tripeptide	Y
34	TPP1	261.1453	1	2.194608	225404.4	260.138	di/tripeptide	Y
35	TPP1	261.1456	1	3.151317	129631.5	260.1383	di/tripeptide	Y
36	TPP1	261.1455	1	5.290575	547601.2	260.1382	di/tripeptide	Y
38	TPP1	263.1434	1	11.43265	165336.1	262.1362	di/tripeptide	Y
40	TPP1	265.1557	1	16.25203	920959.6	264.1484	di/tripeptide	Y
41	TPP1	267.1349	1	2.374933	183594.6	266.1276	di/tripeptide	Y
42	TPP1	269.1616	1	2.183042	334475.7	268.1543	di/tripeptide	Y
45	TPP1	281.1142	1	2.5323	284112.9	280.1069	di/tripeptide	Y
46	TPP1	281.1506	1	6.639933	402843.7	280.1434	di/tripeptide	Y
47	TPP1	288.1929	1	15.91368	175040.6	287.1856	di/tripeptide	Y
50	TPP1	294.1456	1	2.307033	236906.7	293.1383	di/tripeptide	Y
51	TPP1	295.1301	1	3.078567	249463.5	294.1228	di/tripeptide	Y
52	TPP1	295.1662	1	16.41001	629202.1	294.1589	di/tripeptide	Y
55	TPP1	302.2084	1	16.39781	114550.2	301.2012	tri/oligopeptic	Y
57	TPP1	302.2087	1	19.85245	332471.6	301.2014	tri/oligopeptic	Y
58	TPP1	303.1673	1	2.194608	144609.3	302.1601	tri/oligopeptic	Y
59	TPP1	304.1513	1	2.194608	159978.2	303.144	tri/oligopeptic	Y
64	TPP1	318.1671	1	2.183042	920231.2	317.1598	tri/oligopeptic	Y
65	TPP1	318.1673	1	8.99935	141819.4	317.16	tri/oligopeptic	Y
66	TPP1	318.1827	1	22.88427	206560.8	317.1754	tri/oligopeptic	Y
66	TPP1	318.1828	1	22.561	521525.6	317.1755	tri/oligopeptic	Y
73	TPP1	331.1663	1	2.78065	295841.2	330.159	tri/oligopeptic	Y
75	TPP1	332.1829	1	2.5323	114262	331.1756	tri/oligopeptic	Y
80	TPP1	334.1619	1	2.194608	794391.5	333.1547	tri/oligopeptic	Y
89	TPP1	358.2715	1	24.00949	619761.4	357.2642	tri/oligopeptic	Y
90	TPP1	359.2301	1	2.229358	121687.7	358.2228	tri/oligopeptic	Y
94	TPP1	363.1563	1	2.183042	210459.1	362.149	tri/oligopeptic	Y
98	TPP1	375.2248	1	2.183042	178762.3	374.2175	tri/oligopeptic	Y
100	TPP1	376.1729	1	2.420517	121769.4	375.1656	tri/oligopeptic	Y
101	TPP1	376.2255	1	21.16854	240797.6	375.2182	tri/oligopeptic	Y
106	TPP1	395.0399	1	1.596875	176177.1	394.0327	tri/oligopeptic	Y
112	TPP1	415.2127	1	36.47063	125494.4	414.2054	tri/oligopeptic	Y
144	TPP1	488.2524	1	19.12113	236100.8	487.2451	tri/oligopeptic	Y
151	TPP1	500.2889	1	25.19041	167677.9	499.2817	tri/oligopeptic	Y
159	TPP1	520.2785	1	21.16854	142919.4	519.2712	tri/oligopeptic	Y
165	TPP1	531.0149	1	1.596875	293853.4	530.0076	tri/oligopeptic	Y
194	TPP1	599.0028	1	1.596875	264403.2	597.9955	tri/oligopeptic	Y
218	TPP1	666.9904	1	1.596875	266644.2	665.9831	oligopeptide	Y
258	TPP1	802.9655	1	1.596875	200713.7	801.9582	oligopeptide	Y
276	TPP1	870.953	1	1.585625	141502.7	869.9457	oligopeptide	Y
300	TPP1	514.9588	2	1.607775	113160.6	1027.903	oligopeptide	Y
307	TPP1	548.9524	2	1.596875	116258.5	1095.89	oligopeptide	Y
313	TPP1	582.9463	2	1.596875	133610.7	1163.878	oligopeptide	Y

314	TPP1	590.9336	2	1.596875	104025.5	1179.853	oligopeptide	Y
316	TPP1	616.9405	2	1.596875	127818.5	1231.866	oligopeptide	Y
318	TPP1	624.9273	2	1.607775	116638.9	1247.84	oligopeptide	Y
321	TPP1	650.9336	2	1.607775	115941.6	1299.853	oligopeptide	Y
322	TPP1	658.9208	2	1.596875	114580.1	1315.827	oligopeptide	Y
329	TPP1	692.9146	2	1.596875	114866.5	1383.815	oligopeptide	Y
331	TPP1	726.9088	2	1.607775	100252.8	1451.803	oligopeptide	Y
333	TPP1	760.9023	2	1.607775	109802.8	1519.79	oligopeptide	Y
335	TPP1	794.8962	2	1.618825	105588.5	1587.778	oligopeptide	Y
338	TPP1	223.1085	1	4.728142	281913	222.1012	dipeptide	N
339	TPP1	223.1085	1	10.36156	112491.1	222.1012	dipeptide	N
340	TPP1	231.1712	1	8.785917	298049.9	230.1639	di/tripeptide	N
341	TPP1	232.1298	1	2.1716	219129.1	231.1225	di/tripeptide	N
342	TPP1	232.1298	1	1.902508	110235.1	231.1225	di/tripeptide	N
343	TPP1	233.1139	1	2.194608	214165.9	232.1066	di/tripeptide	N
344	TPP1	233.1505	1	3.9329	316939.6	232.1432	di/tripeptide	N
345	TPP1	237.1242	1	4.278833	885188.7	236.1169	di/tripeptide	N
346	TPP1	237.1242	1	11.13268	141139.1	236.1169	di/tripeptide	N
347	TPP1	251.1066	1	2.183042	183472	250.0994	di/tripeptide	N
348	TPP1	260.1615	1	6.117333	400916.2	259.1542	di/tripeptide	N
349	TPP1	262.1196	1	14.46567	103324.5	261.1123	di/tripeptide	N
350	TPP1	263.14	1	14.41424	170894.9	262.1327	di/tripeptide	N
351	TPP1	263.1434	1	13.05917	256073.9	262.1361	di/tripeptide	N
352	TPP1	265.1557	1	14.71689	645284.2	264.1484	di/tripeptide	N
353	TPP1	269.1139	1	2.307033	156469.8	268.1067	di/tripeptide	N
354	TPP1	274.1771	1	10.33156	506105.6	273.1698	di/tripeptide	N
355	TPP1	276.1563	1	2.194608	433463.1	275.149	di/tripeptide	N
356	TPP1	277.2169	1	36.75047	537316.4	276.2096	di/tripeptide	N
357	TPP1	278.1177	1	2.194608	241226.9	277.1104	di/tripeptide	N
358	TPP1	279.1714	1	21.01918	519795	278.1641	di/tripeptide	N
359	TPP1	279.1714	1	19.89113	1210250	278.1641	di/tripeptide	N
360	TPP1	279.1715	1	18.96737	140174	278.1642	di/tripeptide	N
361	TPP1	279.2326	1	37.01238	988600	278.2253	di/tripeptide	N
362	TPP1	281.1143	1	11.51086	181335.1	280.107	di/tripeptide	N
363	TPP1	281.1506	1	5.606542	141636.6	280.1434	di/tripeptide	N
364	TPP1	286.1772	1	13.43262	1104791	285.1699	di/tripeptide	N
365	TPP1	290.1354	1	2.183042	149517.3	289.1282	di/tripeptide	N
366	TPP1	290.1719	1	2.194608	770765.1	289.1646	di/tripeptide	N
367	TPP1	292.1302	1	16.41001	109017.2	291.123	di/tripeptide	N
368	TPP1	294.9403	1	1.675225	220749	293.933	di/tripeptide	N
369	TPP1	295.13	1	12.58608	915728.5	294.1228	di/tripeptide	N
370	TPP1	295.1664	1	14.56998	286538.5	294.1592	di/tripeptide	N
371	TPP1	295.1665	1	13.35489	164127.1	294.1592	di/tripeptide	N
372	TPP1	295.2277	1	37.377	527949	294.2204	di/tripeptide	N
373	TPP1	297.1092	1	2.636492	317476.2	296.1019	di/tripeptide	N
374	TPP1	297.2423	1	37.76648	118587	296.235	di/tripeptide	N
375	TPP1	304.1336	1	2.606783	335980.6	303.1264	tri/oligopeptic	N
376	TPP1	304.1667	1	19.90886	538756.4	303.1594	tri/oligopeptic	N
377	TPP1	304.1878	1	2.576733	173810.5	303.1805	tri/oligopeptic	N
378	TPP1	306.1461	1	10.63	113663.2	305.1388	tri/oligopeptic	N

379	TPP1	306.1669	1	2.194608	291205.6	305.1597	tri/oligopeptic	N
380	TPP1	306.167	1	2.618408	121176.2	305.1597	tri/oligopeptic	N
381	TPP1	313.1767	1	2.183042	107032.1	312.1694	tri/oligopeptic	N
382	TPP1	316.1878	1	2.32975	107982.3	315.1806	tri/oligopeptic	N
383	TPP1	316.2245	1	14.89203	165784.8	315.2172	tri/oligopeptic	N
384	TPP1	317.2096	1	37.377	240170.9	316.2023	tri/oligopeptic	N
385	TPP1	318.2034	1	2.194608	213587.4	317.1961	tri/oligopeptic	N
386	TPP1	318.2037	1	12.28745	669389	317.1964	tri/oligopeptic	N
387	TPP1	318.2037	1	14.56098	149961.9	317.1964	tri/oligopeptic	N
388	TPP1	319.2254	1	37.01238	103592.2	318.2181	tri/oligopeptic	N
389	TPP1	320.1464	1	2.116342	170700.5	319.1391	tri/oligopeptic	N
390	TPP1	322.144	1	2.295758	320150.5	321.1368	tri/oligopeptic	N
391	TPP1	324.1566	1	2.509608	151630.1	323.1494	tri/oligopeptic	N
392	TPP1	328.2245	1	15.41343	219502.2	327.2172	tri/oligopeptic	N
393	TPP1	331.1988	1	2.194608	259363.8	330.1915	tri/oligopeptic	N
394	TPP1	331.2349	1	2.194608	109864	330.2276	tri/oligopeptic	N
395	TPP1	332.2193	1	4.9847	300802.9	331.212	tri/oligopeptic	N
396	TPP1	332.2193	1	9.5437	313352.6	331.212	tri/oligopeptic	N
397	TPP1	332.2194	1	19.42713	108549.1	331.2121	tri/oligopeptic	N
398	TPP1	333.1571	1	17.05924	453391.8	332.1498	tri/oligopeptic	N
399	TPP1	333.1779	1	2.194608	280643.1	332.1706	tri/oligopeptic	N
400	TPP1	336.1236	1	2.262583	135855	335.1163	tri/oligopeptic	N
401	TPP1	336.139	1	21.01918	214250.6	335.1317	tri/oligopeptic	N
402	TPP1	336.1933	1	23.03313	177137.8	335.1861	tri/oligopeptic	N
403	TPP1	340.2858	1	37.377	142454.5	339.2785	tri/oligopeptic	N
404	TPP1	342.2402	1	19.86153	225628.4	341.2329	tri/oligopeptic	N
405	TPP1	342.2402	1	20.73378	1035432	341.233	tri/oligopeptic	N
406	TPP1	343.1991	1	13.31236	889164.8	342.1918	tri/oligopeptic	N
407	TPP1	345.2147	1	15.7986	117227.4	344.2075	tri/oligopeptic	N
408	TPP1	346.2352	1	19.17043	396054.6	345.2279	tri/oligopeptic	N
409	TPP1	347.1573	1	2.194608	219918.5	346.15	tri/oligopeptic	N
410	TPP1	348.1414	1	2.116342	267898.4	347.1342	tri/oligopeptic	N
411	TPP1	348.1778	1	2.262583	609053.7	347.1705	tri/oligopeptic	N
412	TPP1	348.178	1	9.995033	281076.6	347.1708	tri/oligopeptic	N
413	TPP1	354.1307	1	2.194608	218057.1	353.1234	tri/oligopeptic	N
414	TPP1	358.2962	1	36.42645	160834.5	357.2889	tri/oligopeptic	N
415	TPP1	359.1573	1	2.194608	152782.7	358.15	tri/oligopeptic	N
416	TPP1	359.1934	1	2.183042	293574.8	358.1861	tri/oligopeptic	N
417	TPP1	359.2303	1	16.81008	115106.2	358.223	tri/oligopeptic	N
418	TPP1	359.2663	1	2.2403	129574.8	358.259	tri/oligopeptic	N
419	TPP1	360.2146	1	20.4456	112145.6	359.2073	tri/oligopeptic	N
420	TPP1	361.173	1	2.183042	197819.5	360.1657	tri/oligopeptic	N
421	TPP1	361.1732	1	2.819	128837.8	360.166	tri/oligopeptic	N
422	TPP1	362.2088	1	21.3447	145341.1	361.2016	tri/oligopeptic	N
423	TPP1	363.1886	1	2.5545	102944	362.1813	tri/oligopeptic	N
424	TPP1	366.134	1	2.194608	225905.9	365.1268	tri/oligopeptic	N
425	TPP1	368.1468	1	8.852467	108384.5	367.1395	tri/oligopeptic	N
426	TPP1	371.2304	1	14.11968	666059.9	370.2231	tri/oligopeptic	N
427	TPP1	373.2092	1	2.576733	2070175	372.2019	tri/oligopeptic	N
428	TPP1	373.2459	1	16.47554	992004.9	372.2386	tri/oligopeptic	N

429	TPP1	373.2462	1	18.80608	124517.1	372.2389	tri/oligopeptic	N
430	TPP1	374.2045	1	2.183042	581604.1	373.1972	tri/oligopeptic	N
431	TPP1	375.189	1	8.367283	115244.1	374.1817	tri/oligopeptic	N
432	TPP1	376.2252	1	23.89678	353426.4	375.2179	tri/oligopeptic	N
433	TPP1	377.2042	1	2.194608	184855	376.1969	tri/oligopeptic	N
434	TPP1	389.2048	1	10.81154	276222	388.1975	tri/oligopeptic	N
435	TPP1	391.2198	1	2.21825	107812.6	390.2125	tri/oligopeptic	N
436	TPP1	401.2412	1	15.06663	158445.6	400.2339	tri/oligopeptic	N
437	TPP1	404.2152	1	2.475942	424661.2	403.2079	tri/oligopeptic	N
438	TPP1	405.1992	1	2.206367	131816.3	404.192	tri/oligopeptic	N
439	TPP1	408.1449	1	2.5323	112779.5	407.1376	tri/oligopeptic	N
440	TPP1	408.2145	1	17.54296	152335.9	407.2073	tri/oligopeptic	N
441	TPP1	419.2148	1	2.27365	1126873	418.2075	tri/oligopeptic	N
442	TPP1	419.2304	1	24.53998	1536043	418.2232	tri/oligopeptic	N
443	TPP1	423.2255	1	15.8946	179781.4	422.2182	tri/oligopeptic	N
444	TPP1	429.2725	1	23.96479	246307.3	428.2652	tri/oligopeptic	N
445	TPP1	431.1942	1	19.96136	199353.9	430.1869	tri/oligopeptic	N
446	TPP1	431.2517	1	17.46008	150160.9	430.2445	tri/oligopeptic	N
447	TPP1	432.2463	1	2.183042	147062.7	431.239	tri/oligopeptic	N
448	TPP1	433.2672	1	12.17632	926242.1	432.26	tri/oligopeptic	N
449	TPP1	434.2263	1	10.68503	404689.4	433.219	tri/oligopeptic	N
450	TPP1	443.2515	1	19.61331	739667.1	442.2443	tri/oligopeptic	N
451	TPP1	444.2467	1	2.5323	724110.8	443.2395	tri/oligopeptic	N
452	TPP1	222.627	2	2.5545	110728.3	443.2395	tri/oligopeptic	N
453	TPP1	444.247	1	13.44047	493649.5	443.2397	tri/oligopeptic	N
454	TPP1	445.2308	1	16.27382	431831.6	444.2235	tri/oligopeptic	N
455	TPP1	224.6321	2	2.5323	280258.3	447.2496	tri/oligopeptic	N
456	TPP1	450.2001	1	13.51618	656103.1	449.1928	tri/oligopeptic	N
457	TPP1	450.2204	1	2.194608	232753	449.2131	tri/oligopeptic	N
458	TPP1	456.2835	1	20.99268	317198.1	455.2762	tri/oligopeptic	N
459	TPP1	457.2423	1	14.17213	124050.4	456.235	tri/oligopeptic	N
460	TPP1	458.2257	1	2.194608	137205.2	457.2184	tri/oligopeptic	N
461	TPP1	458.2263	1	14.87784	235457.1	457.219	tri/oligopeptic	N
462	TPP1	460.2417	1	2.645608	119808	459.2344	tri/oligopeptic	N
463	TPP1	461.2621	1	15.59184	187757.4	460.2549	tri/oligopeptic	N
464	TPP1	467.1437	1	23.24796	410426.1	466.1364	tri/oligopeptic	N
465	TPP1	470.2987	1	21.43569	2544861	469.2914	tri/oligopeptic	N
466	TPP1	236.1324	2	2.082508	128431	470.2503	tri/oligopeptic	N
467	TPP1	472.2569	1	27.18286	1135490	471.2496	tri/oligopeptic	N
468	TPP1	473.2735	1	13.07853	134950.7	472.2662	tri/oligopeptic	N
469	TPP1	473.2735	1	14.8516	385024.8	472.2663	tri/oligopeptic	N
470	TPP1	474.2316	1	2.183042	396415.1	473.2243	tri/oligopeptic	N
471	TPP1	475.2778	1	17.69141	256807	474.2705	tri/oligopeptic	N
472	TPP1	475.2927	1	26.25843	442448	474.2854	tri/oligopeptic	N
473	TPP1	238.64	2	2.262583	276071.2	475.2655	tri/oligopeptic	N
474	TPP1	478.2518	1	2.194608	105857.4	477.2446	tri/oligopeptic	N
475	TPP1	481.231	1	24.36167	303312.7	480.2237	tri/oligopeptic	N
476	TPP1	483.2464	1	12.64023	161178.3	482.2392	tri/oligopeptic	N
477	TPP1	486.294	1	18.44501	401282.4	485.2867	tri/oligopeptic	N
478	TPP1	487.289	1	16.27382	166294.7	486.2817	tri/oligopeptic	N

479	TPP1	487.2894	1	16.56902	119307.2	486.2821	tri/oligopeptic	N
480	TPP1	487.3142	1	21.45246	145912.5	486.307	tri/oligopeptic	N
481	TPP1	488.2734	1	15.25283	154767.3	487.2661	tri/oligopeptic	N
482	TPP1	489.3091	1	27.88488	1169345	488.3018	tri/oligopeptic	N
483	TPP1	489.358	1	36.4528	100684.9	488.3507	tri/oligopeptic	N
484	TPP1	491.2472	1	2.386317	202749.8	490.24	tri/oligopeptic	N
485	TPP1	497.1898	1	13.61781	276881.4	496.1825	tri/oligopeptic	N
486	TPP1	502.3253	1	21.57278	461516.7	501.318	tri/oligopeptic	N
487	TPP1	503.2845	1	15.18874	101914.8	502.2772	tri/oligopeptic	N
488	TPP1	504.2424	1	2.194608	141417.8	503.2352	tri/oligopeptic	N
489	TPP1	504.2681	1	15.51249	439063	503.2608	tri/oligopeptic	N
490	TPP1	506.1546	1	25.23488	326865.5	505.1473	tri/oligopeptic	N
491	TPP1	506.2296	1	20.4456	173914.1	505.2223	tri/oligopeptic	N
492	TPP1	506.266	1	17.27622	457793	505.2588	tri/oligopeptic	N
493	TPP1	517.2627	1	2.183042	119857.6	516.2554	tri/oligopeptic	N
494	TPP1	519.2421	1	2.183042	177645.2	518.2348	tri/oligopeptic	N
495	TPP1	522.2576	1	17.89618	368137.7	521.2503	tri/oligopeptic	N
496	TPP1	262.6643	2	13.98698	100722	523.314	tri/oligopeptic	N
497	TPP1	531.2792	1	3.180542	169647.2	530.2719	tri/oligopeptic	N
498	TPP1	535.274	1	2.956392	191172.9	534.2667	tri/oligopeptic	N
499	TPP1	545.2944	1	2.465533	324481.3	544.2871	tri/oligopeptic	N
500	TPP1	547.2897	1	23.46378	105415.2	546.2825	tri/oligopeptic	N
501	TPP1	548.2207	1	2.206367	162265.1	547.2134	tri/oligopeptic	N
502	TPP1	550.256	1	18.96737	268721	549.2487	tri/oligopeptic	N
503	TPP1	556.3372	1	22.40913	189092.5	555.3299	tri/oligopeptic	N
504	TPP1	557.332	1	23.30199	286429.1	556.3247	tri/oligopeptic	N
505	TPP1	558.326	1	2.194608	239764.8	557.3187	tri/oligopeptic	N
506	TPP1	279.6667	2	2.183042	576478.5	557.3188	tri/oligopeptic	N
507	TPP1	559.2742	1	2.618408	147979.1	558.2669	tri/oligopeptic	N
508	TPP1	559.2746	1	11.78493	228333.5	558.2673	tri/oligopeptic	N
509	TPP1	560.269	1	2.194608	105902.1	559.2617	tri/oligopeptic	N
510	TPP1	561.29	1	14.92539	446654.5	560.2828	tri/oligopeptic	N
511	TPP1	574.3263	1	26.24413	100211.7	573.319	tri/oligopeptic	N
512	TPP1	288.1477	2	18.86806	100228.6	574.2809	tri/oligopeptic	N
513	TPP1	289.6511	2	2.262583	238065.7	577.2877	tri/oligopeptic	N
514	TPP1	580.2288	1	28.67756	190994.8	579.2215	tri/oligopeptic	N
515	TPP1	580.2749	1	15.68346	134701.2	579.2676	tri/oligopeptic	N
516	TPP1	586.2857	1	16.66209	161728.2	585.2784	tri/oligopeptic	N
517	TPP1	588.3006	1	18.00173	377762.4	587.2933	tri/oligopeptic	N
518	TPP1	588.3011	1	17.35949	206899.4	587.2939	tri/oligopeptic	N
519	TPP1	590.3208	1	28.20753	912592.7	589.3136	tri/oligopeptic	N
520	TPP1	597.2903	1	15.48333	207511.5	596.283	tri/oligopeptic	N
521	TPP1	305.167	2	18.14001	123063.8	608.3194	tri/oligopeptic	N
522	TPP1	618.312	1	18.49333	280044.1	617.3047	oligopeptide	N
523	TPP1	315.1672	2	2.21825	345843.7	628.3199	oligopeptide	N
524	TPP1	631.2957	1	16.45283	333451.8	630.2884	oligopeptide	N
525	TPP1	631.3118	1	25.7057	140803	630.3045	oligopeptide	N
526	TPP1	632.3275	1	12.47783	269865.6	631.3202	oligopeptide	N
527	TPP1	634.3065	1	2.78905	151227.2	633.2992	oligopeptide	N
528	TPP1	321.6961	2	16.81643	107085.9	641.3777	oligopeptide	N

529	TPP1	322.6858	2	14.00702	213423.2	643.357	oligopeptide	N
530	TPP1	648.3589	1	19.6073	239946.4	647.3516	oligopeptide	N
531	TPP1	649.317	1	2.352442	206437.7	648.3097	oligopeptide	N
532	TPP1	325.1663	2	14.86041	295596.3	648.318	oligopeptide	N
533	TPP1	650.3176	1	21.86493	103292.1	649.3103	oligopeptide	N
534	TPP1	330.662	2	17.2042	3620718	659.3095	oligopeptide	N
535	TPP1	669.4213	1	28.10895	410772.9	668.4141	oligopeptide	N
536	TPP1	335.2143	2	28.10895	166065.1	668.4141	oligopeptide	N
537	TPP1	672.3592	1	18.38946	203388.3	671.352	oligopeptide	N
538	TPP1	676.3357	1	16.39781	199893.1	675.3285	oligopeptide	N
539	TPP1	683.4117	1	22.6982	213255.3	682.4044	oligopeptide	N
540	TPP1	685.3543	1	18.65493	110560.7	684.3471	oligopeptide	N
541	TPP1	349.2356	2	20.64037	215781.7	696.4566	oligopeptide	N
542	TPP1	356.6777	2	16.96288	162705.2	711.3409	oligopeptide	N
543	TPP1	713.3858	1	17.81028	115652.6	712.3785	oligopeptide	N
544	TPP1	720.288	1	30.38458	225304.9	719.2808	oligopeptide	N
545	TPP1	362.1647	2	2.194608	187589.7	722.3149	oligopeptide	N
546	TPP1	241.7791	3	2.194608	228797.9	722.3154	oligopeptide	N
547	TPP1	362.225	2	16.22791	148188.2	722.4355	oligopeptide	N
548	TPP1	733.3956	1	27.89757	106209.9	732.3883	oligopeptide	N
549	TPP1	373.7073	2	12.85807	1160834	745.4001	oligopeptide	N
550	TPP1	747.3912	1	17.34187	119142.5	746.384	oligopeptide	N
551	TPP1	758.3345	1	17.2042	151518	757.3273	oligopeptide	N
552	TPP1	761.3895	1	24.26591	229415.8	760.3822	oligopeptide	N
553	TPP1	381.6863	2	2.363625	131342	761.3581	oligopeptide	N
554	TPP1	762.4025	1	18.76222	108034.6	761.3952	oligopeptide	N
555	TPP1	385.7253	2	2.374933	237100.4	769.436	oligopeptide	N
556	TPP1	390.6871	2	11.09963	223551.1	779.3596	oligopeptide	N
557	TPP1	391.179	2	13.11365	137697.8	780.3433	oligopeptide	N
558	TPP1	395.2304	2	25.15031	150015.8	788.4462	oligopeptide	N
559	TPP1	799.4228	1	19.72483	646522.6	798.4156	oligopeptide	N
560	TPP1	400.2152	2	19.72483	151649.3	798.4158	oligopeptide	N
561	TPP1	400.7127	2	15.82025	280579.4	799.4108	oligopeptide	N
562	TPP1	412.1941	2	19.73385	178540.3	822.3737	oligopeptide	N
563	TPP1	838.3606	1	14.50453	103119.5	837.3533	oligopeptide	N
564	TPP1	425.205	2	24.15548	229580.4	848.3955	oligopeptide	N
565	TPP1	425.205	2	23.21603	146244.9	848.3955	oligopeptide	N
566	TPP1	426.1697	2	12.75703	153879.5	850.3249	oligopeptide	N
567	TPP1	426.7467	2	17.39143	209550.8	851.4789	oligopeptide	N
568	TPP1	429.7128	2	20.27536	183846.2	857.411	oligopeptide	N
569	TPP1	433.7546	2	19.67143	195430.8	865.4947	oligopeptide	N
570	TPP1	435.7309	2	23.58158	582166	869.4472	oligopeptide	N
571	TPP1	436.2368	2	25.44717	103211.5	870.4591	oligopeptide	N
572	TPP1	438.2289	2	16.84127	161612.4	874.4433	oligopeptide	N
573	TPP1	296.1342	3	13.93757	229416.8	885.3807	oligopeptide	N
574	TPP1	890.3927	1	32.64786	132452.7	889.3854	oligopeptide	N
575	TPP1	460.6674	2	14.16565	119015.8	919.3202	oligopeptide	N
576	TPP1	461.6877	2	2.262583	110845.7	921.3609	oligopeptide	N
577	TPP1	487.7631	2	16.19805	154598	973.5116	oligopeptide	N
578	TPP1	488.7527	2	22.72034	103466.6	975.4908	oligopeptide	N

579	TPP1	515.2477	2	18.05342	174117.5	1028.481	oligopeptide	N
580	TPP1	578.2939	2	15.25283	421008.5	1154.573	oligopeptide	N
581	TPP1	596.7805	2	20.16584	180004	1191.547	oligopeptide	N
582	TPP1	643.8141	2	17.72069	196992.9	1285.614	oligopeptide	N
583	TPP1	684.9278	2	1.596875	122133.4	1367.841	oligopeptide	N
584	TPP1	718.9214	2	1.607775	110720.2	1435.828	oligopeptide	N
585	TPP1	752.9153	2	1.607775	105421.8	1503.816	oligopeptide	N
ID	Protein	mz	charge	retentiont	raw.abund	mass	type	Match
2	TPP2	231.1712	1	4.997817	135424.5	230.1639	di/tripeptide	Y
3	TPP2	231.1712	1	9.505967	386322.7	230.1639	di/tripeptide	Y
4	TPP2	231.1712	1	11.9964	416855	230.1639	di/tripeptide	Y
5	TPP2	233.1502	1	2.1884	100058.4	232.1429	di/tripeptide	Y
11	TPP2	245.1868	1	16.40949	500797.7	244.1795	di/tripeptide	Y
13	TPP2	245.1869	1	14.5004	115043.7	244.1796	di/tripeptide	Y
14	TPP2	245.1868	1	17.66532	391293.2	244.1796	di/tripeptide	Y
15	TPP2	246.1455	1	2.177583	200113.4	245.1382	di/tripeptide	Y
20	TPP2	247.1296	1	2.177583	191821.1	246.1223	di/tripeptide	Y
22	TPP2	247.1297	1	4.587892	125962	246.1224	di/tripeptide	Y
23	TPP2	249.1285	1	2.734183	106288.5	248.1213	di/tripeptide	Y
30	TPP2	260.1613	1	2.1884	289570.5	259.154	di/tripeptide	Y
33	TPP2	260.1976	1	2.1884	336150.9	259.1904	di/tripeptide	Y
34	TPP2	261.1453	1	2.1884	166961.9	260.138	di/tripeptide	Y
36	TPP2	261.1455	1	5.296858	213905	260.1382	di/tripeptide	Y
40	TPP2	265.1557	1	16.26598	766152.8	264.1484	di/tripeptide	Y
45	TPP2	281.1142	1	2.536775	128186.6	280.1069	di/tripeptide	Y
46	TPP2	281.1506	1	6.647933	153884.2	280.1434	di/tripeptide	Y
51	TPP2	295.1301	1	3.0816	184850.2	294.1228	di/tripeptide	Y
52	TPP2	295.1662	1	16.40949	254863.8	294.1589	di/tripeptide	Y
55	TPP2	302.2086	1	16.80439	238821.2	301.2014	tri/oligopeptic	Y
55	TPP2	302.2087	1	16.0749	141453.4	301.2014	tri/oligopeptic	Y
57	TPP2	302.2087	1	19.8542	134713.9	301.2014	tri/oligopeptic	Y
64	TPP2	318.1671	1	2.177583	300332	317.1598	tri/oligopeptic	Y
65	TPP2	318.1673	1	9.003108	154665.8	317.16	tri/oligopeptic	Y
66	TPP2	318.1828	1	22.55677	153273.6	317.1755	tri/oligopeptic	Y
73	TPP2	331.1663	1	2.787617	218811	330.159	tri/oligopeptic	Y
80	TPP2	334.1619	1	2.1884	921797.6	333.1547	tri/oligopeptic	Y
85	TPP2	344.2559	1	21.35241	115673.4	343.2486	tri/oligopeptic	Y
89	TPP2	358.2715	1	24.00614	694179.2	357.2642	tri/oligopeptic	Y
90	TPP2	359.2301	1	2.222067	148663.8	358.2228	tri/oligopeptic	Y
94	TPP2	363.1563	1	2.177583	114539.5	362.149	tri/oligopeptic	Y
105	TPP2	390.1883	1	2.313217	105142.4	389.1811	tri/oligopeptic	Y
112	TPP2	415.2127	1	36.47674	171492.8	414.2054	tri/oligopeptic	Y
338	TPP2	237.1242	1	4.276717	502257.7	236.1169	di/tripeptide	N
339	TPP2	244.0936	1	1.898758	156335.2	243.0863	di/tripeptide	N
340	TPP2	263.1434	1	13.07238	142370.9	262.1361	di/tripeptide	N
341	TPP2	265.1557	1	14.72633	208975	264.1484	di/tripeptide	N
342	TPP2	272.9448	1	1.5495	118289.5	271.9376	di/tripeptide	N
343	TPP2	276.1563	1	2.1884	473827.7	275.149	di/tripeptide	N
344	TPP2	279.1714	1	21.02306	170124.9	278.1641	di/tripeptide	N
345	TPP2	279.1714	1	19.89648	230375	278.1641	di/tripeptide	N

346	TPP2	279.2326	1	37.00563	113489.6	278.2253	di/tripeptide	N
347	TPP2	280.1299	1	2.199483	108212.2	279.1227	di/tripeptide	N
348	TPP2	286.1772	1	13.45209	618239.2	285.1699	di/tripeptide	N
349	TPP2	288.2037	1	2.1884	105253.5	287.1965	di/tripeptide	N
350	TPP2	290.1719	1	2.1884	254514.9	289.1646	di/tripeptide	N
351	TPP2	290.1721	1	4.884233	114203.5	289.1648	di/tripeptide	N
352	TPP2	294.9403	1	1.669308	162880.5	293.933	di/tripeptide	N
353	TPP2	295.13	1	12.59933	316486.1	294.1228	di/tripeptide	N
354	TPP2	295.1664	1	14.57049	124749.8	294.1592	di/tripeptide	N
355	TPP2	295.1665	1	13.37085	102192.4	294.1592	di/tripeptide	N
356	TPP2	295.2277	1	37.37093	120066.3	294.2204	di/tripeptide	N
357	TPP2	297.1092	1	2.634942	124889.3	296.1019	di/tripeptide	N
358	TPP2	300.1929	1	14.46062	293045.3	299.1856	di/tripeptide	N
359	TPP2	302.2086	1	11.27234	323010.1	301.2013	tri/oligopeptic	N
360	TPP2	304.1878	1	2.576392	149723.3	303.1805	tri/oligopeptic	N
361	TPP2	306.1669	1	2.1884	671211.7	305.1597	tri/oligopeptic	N
362	TPP2	314.2087	1	13.6132	154489.3	313.2014	tri/oligopeptic	N
363	TPP2	316.2244	1	21.19288	193751.9	315.2171	tri/oligopeptic	N
364	TPP2	316.2244	1	19.58301	159317.7	315.2172	tri/oligopeptic	N
365	TPP2	316.2244	1	20.82078	130570.8	315.2172	tri/oligopeptic	N
366	TPP2	316.2245	1	20.29017	174884.9	315.2172	tri/oligopeptic	N
367	TPP2	317.1833	1	6.438217	160153.5	316.176	tri/oligopeptic	N
368	TPP2	318.2037	1	12.29903	2201521	317.1964	tri/oligopeptic	N
369	TPP2	318.2037	1	16.70711	128254	317.1964	tri/oligopeptic	N
370	TPP2	322.144	1	2.290142	1307407	321.1368	tri/oligopeptic	N
371	TPP2	324.1299	1	2.1884	381735.8	323.1226	tri/oligopeptic	N
372	TPP2	328.2245	1	15.41029	148634.4	327.2172	tri/oligopeptic	N
373	TPP2	328.2245	1	18.28107	199573	327.2172	tri/oligopeptic	N
374	TPP2	330.2402	1	19.17391	158810.1	329.2329	tri/oligopeptic	N
375	TPP2	331.1988	1	2.1884	131297.8	330.1915	tri/oligopeptic	N
376	TPP2	331.2349	1	2.1884	225837.9	330.2276	tri/oligopeptic	N
377	TPP2	332.1829	1	11.14161	850968.6	331.1756	tri/oligopeptic	N
378	TPP2	332.2193	1	21.21669	2022052	331.212	tri/oligopeptic	N
379	TPP2	332.2194	1	15.88318	1064036	331.2121	tri/oligopeptic	N
380	TPP2	332.2194	1	20.46273	183692.4	331.2121	tri/oligopeptic	N
381	TPP2	332.2194	1	19.42958	131997.7	331.2121	tri/oligopeptic	N
382	TPP2	333.1779	1	2.1884	185614.8	332.1706	tri/oligopeptic	N
383	TPP2	333.1783	1	2.787617	149686.3	332.171	tri/oligopeptic	N
384	TPP2	336.139	1	21.02306	123294.9	335.1317	tri/oligopeptic	N
385	TPP2	340.9328	1	1.561442	110119.7	339.9256	tri/oligopeptic	N
386	TPP2	342.2043	1	21.74471	109611.2	341.197	tri/oligopeptic	N
387	TPP2	342.2402	1	20.72298	107263.3	341.233	tri/oligopeptic	N
388	TPP2	344.2559	1	23.29111	580437	343.2486	tri/oligopeptic	N
389	TPP2	344.256	1	22.40665	289134.7	343.2487	tri/oligopeptic	N
390	TPP2	344.256	1	22.17358	176573.2	343.2487	tri/oligopeptic	N
391	TPP2	345.1459	1	14.00087	128385	344.1386	tri/oligopeptic	N
392	TPP2	346.1987	1	16.41489	107450.7	345.1915	tri/oligopeptic	N
393	TPP2	346.2352	1	19.16568	155850.3	345.2279	tri/oligopeptic	N
394	TPP2	348.1778	1	2.25585	195006.6	347.1705	tri/oligopeptic	N
395	TPP2	348.178	1	9.999408	200735.4	347.1708	tri/oligopeptic	N

396	TPP2	350.1757	1	2.65355	124979.2	349.1684	tri/oligopeptic	N
397	TPP2	358.1986	1	15.62301	792640.9	357.1914	tri/oligopeptic	N
398	TPP2	358.2714	1	26.26385	291023.9	357.2642	tri/oligopeptic	N
399	TPP2	358.2715	1	25.32757	332119.5	357.2642	tri/oligopeptic	N
400	TPP2	358.2715	1	24.93741	403529.1	357.2643	tri/oligopeptic	N
401	TPP2	359.2303	1	16.81376	811984.9	358.223	tri/oligopeptic	N
402	TPP2	359.2303	1	15.41029	2364329	358.2231	tri/oligopeptic	N
403	TPP2	359.2304	1	20.53754	103776	358.2232	tri/oligopeptic	N
404	TPP2	359.2411	1	2.1884	103627.9	358.2339	tri/oligopeptic	N
405	TPP2	360.2143	1	17.60134	159553.6	359.2071	tri/oligopeptic	N
406	TPP2	360.2144	1	21.24149	220418.2	359.2071	tri/oligopeptic	N
407	TPP2	360.2146	1	16.06823	354432	359.2073	tri/oligopeptic	N
408	TPP2	360.2146	1	20.4459	125092.6	359.2073	tri/oligopeptic	N
409	TPP2	361.2457	1	2.1884	116487.8	360.2384	tri/oligopeptic	N
410	TPP2	362.1937	1	12.14779	116113.1	361.1864	tri/oligopeptic	N
411	TPP2	362.2088	1	21.33214	1342999	361.2016	tri/oligopeptic	N
412	TPP2	366.134	1	2.1884	309980.6	365.1268	tri/oligopeptic	N
413	TPP2	371.2304	1	14.13379	178210.4	370.2231	tri/oligopeptic	N
414	TPP2	371.2305	1	11.14161	104817.3	370.2232	tri/oligopeptic	N
415	TPP2	373.2092	1	2.576392	121970.6	372.2019	tri/oligopeptic	N
416	TPP2	374.1092	1	2.1884	339847.9	373.102	tri/oligopeptic	N
417	TPP2	374.23	1	12.99975	139222.2	373.2227	tri/oligopeptic	N
418	TPP2	374.23	1	20.64168	440443	373.2228	tri/oligopeptic	N
419	TPP2	374.2301	1	21.18305	190482.7	373.2228	tri/oligopeptic	N
420	TPP2	374.2301	1	22.01933	437700.3	373.2228	tri/oligopeptic	N
421	TPP2	374.2302	1	14.32368	143120.8	373.2229	tri/oligopeptic	N
422	TPP2	375.189	1	8.368875	150048.7	374.1817	tri/oligopeptic	N
423	TPP2	377.2042	1	2.1884	254004.3	376.1969	tri/oligopeptic	N
424	TPP2	388.2201	1	2.1884	106019.3	387.2128	tri/oligopeptic	N
425	TPP2	389.241	1	10.70473	131638.8	388.2337	tri/oligopeptic	N
426	TPP2	389.2411	1	18.8771	100361.3	388.2338	tri/oligopeptic	N
427	TPP2	389.2517	1	2.199483	155264	388.2445	tri/oligopeptic	N
428	TPP2	392.1864	1	20.0029	878533.8	391.1791	tri/oligopeptic	N
429	TPP2	393.2148	1	23.93388	112259.4	392.2075	tri/oligopeptic	N
430	TPP2	398.1576	1	5.231058	509581.4	397.1503	tri/oligopeptic	N
431	TPP2	398.3428	1	34.87829	136017.9	397.3356	tri/oligopeptic	N
432	TPP2	402.2358	1	2.634942	127349	401.2285	tri/oligopeptic	N
433	TPP2	405.1992	1	2.199483	131013.8	404.192	tri/oligopeptic	N
434	TPP2	405.236	1	17.37768	150318.9	404.2287	tri/oligopeptic	N
435	TPP2	408.9202	1	1.561442	121875.9	407.913	tri/oligopeptic	N
436	TPP2	409.0204	1	2.210858	127835.9	408.0131	tri/oligopeptic	N
437	TPP2	410.1937	1	19.54245	353671.8	409.1864	tri/oligopeptic	N
438	TPP2	413.2776	1	21.36654	147092.8	412.2703	tri/oligopeptic	N
439	TPP2	419.2148	1	2.26725	145810.9	418.2075	tri/oligopeptic	N
440	TPP2	419.2304	1	24.53838	237200.4	418.2232	tri/oligopeptic	N
441	TPP2	419.2515	1	17.62796	245265	418.2442	tri/oligopeptic	N
442	TPP2	419.2516	1	13.19333	410168.8	418.2443	tri/oligopeptic	N
443	TPP2	424.2095	1	13.27385	146034.8	423.2022	tri/oligopeptic	N
444	TPP2	429.3091	1	25.69925	162239.8	428.3018	tri/oligopeptic	N
445	TPP2	431.2877	1	20.77187	1243182	430.2805	tri/oligopeptic	N

446	TPP2	440.1674	1	2.222067	156825	439.1601	tri/oligopeptic	N
447	TPP2	446.2256	1	2.1884	186565.4	445.2184	tri/oligopeptic	N
448	TPP2	446.2263	1	10.00723	294015.2	445.219	tri/oligopeptic	N
449	TPP2	449.2081	1	16.65863	240668.7	448.2009	tri/oligopeptic	N
450	TPP2	451.2568	1	18.82551	167728.3	450.2495	tri/oligopeptic	N
451	TPP2	458.2257	1	2.1884	484273	457.2184	tri/oligopeptic	N
452	TPP2	459.257	1	2.1884	111382	458.2498	tri/oligopeptic	N
453	TPP2	230.6482	2	2.348325	137433.7	459.2819	tri/oligopeptic	N
454	TPP2	464.1981	1	22.38317	139024.4	463.1908	tri/oligopeptic	N
455	TPP2	464.218	1	2.1884	107898.5	463.2107	tri/oligopeptic	N
456	TPP2	464.2521	1	16.96685	304705.9	463.2448	tri/oligopeptic	N
457	TPP2	465.1737	1	2.1884	128303.4	464.1664	tri/oligopeptic	N
458	TPP2	467.3488	1	33.8306	104233.7	466.3415	tri/oligopeptic	N
459	TPP2	470.2987	1	21.43092	283038.9	469.2914	tri/oligopeptic	N
460	TPP2	472.2414	1	2.210858	144372.3	471.2341	tri/oligopeptic	N
461	TPP2	472.2569	1	27.18503	1450951	471.2496	tri/oligopeptic	N
462	TPP2	472.3147	1	23.31904	276080.8	471.3074	tri/oligopeptic	N
463	TPP2	475.2927	1	26.24974	3664380	474.2854	tri/oligopeptic	N
464	TPP2	476.273	1	16.28594	165093.6	475.2658	tri/oligopeptic	N
465	TPP2	476.9077	1	1.561442	123538.1	475.9004	tri/oligopeptic	N
466	TPP2	483.2464	1	12.65471	1483696	482.2392	tri/oligopeptic	N
467	TPP2	483.2466	1	14.38507	238287.1	482.2393	tri/oligopeptic	N
468	TPP2	486.294	1	18.44643	392367.9	485.2867	tri/oligopeptic	N
469	TPP2	487.289	1	16.28594	181478	486.2817	tri/oligopeptic	N
470	TPP2	490.2633	1	2.1884	100815.1	489.256	tri/oligopeptic	N
471	TPP2	245.6353	2	2.199483	219784.5	489.256	tri/oligopeptic	N
472	TPP2	246.624	2	18.75618	123195	491.2335	tri/oligopeptic	N
473	TPP2	504.3044	1	21.21066	105244.5	503.2972	tri/oligopeptic	N
474	TPP2	506.2296	1	20.4459	648582.1	505.2223	tri/oligopeptic	N
475	TPP2	509.2779	1	28.34368	578349.6	508.2706	tri/oligopeptic	N
476	TPP2	511.2048	1	2.360142	256324.8	510.1975	tri/oligopeptic	N
477	TPP2	511.3754	1	32.60342	121295.6	510.3681	tri/oligopeptic	N
478	TPP2	257.1473	2	19.73062	108819.3	512.28	tri/oligopeptic	N
479	TPP2	517.2636	1	15.08162	117452.9	516.2563	tri/oligopeptic	N
480	TPP2	518.2827	1	2.1884	109103.2	517.2755	tri/oligopeptic	N
481	TPP2	259.6455	2	2.177583	135074.9	517.2764	tri/oligopeptic	N
482	TPP2	518.3202	1	24.50025	423474.4	517.3129	tri/oligopeptic	N
483	TPP2	522.2576	1	17.90261	119411.4	521.2503	tri/oligopeptic	N
484	TPP2	526.3621	1	27.36036	146501.8	525.3548	tri/oligopeptic	N
485	TPP2	529.1979	1	15.42055	1074035	528.1906	tri/oligopeptic	N
486	TPP2	529.198	1	12.22855	100923.8	528.1908	tri/oligopeptic	N
487	TPP2	531.2899	1	2.15545	430404.1	530.2827	tri/oligopeptic	N
488	TPP2	266.1487	2	2.144192	591018	530.2828	tri/oligopeptic	N
489	TPP2	542.3568	1	22.3421	147563.8	541.3496	tri/oligopeptic	N
490	TPP2	544.8952	1	1.561442	142740.2	543.8879	tri/oligopeptic	N
491	TPP2	550.256	1	18.97212	376966.3	549.2487	tri/oligopeptic	N
492	TPP2	559.2742	1	2.612817	403368.5	558.2669	tri/oligopeptic	N
493	TPP2	559.3105	1	18.2159	1416560	558.3032	tri/oligopeptic	N
494	TPP2	280.1589	2	18.2159	466098.7	558.3032	tri/oligopeptic	N
495	TPP2	281.6642	2	2.199483	1143034	561.3138	tri/oligopeptic	N

496	TPP2	562.3211	1	2.1884	605490.2	561.3139	tri/oligopeptic	N
497	TPP2	562.8071	1	1.6454	113438.9	561.7998	tri/oligopeptic	N
498	TPP2	568.2635	1	17.72558	180515.5	567.2563	tri/oligopeptic	N
499	TPP2	569.2768	1	2.1884	158093	568.2696	tri/oligopeptic	N
500	TPP2	285.1423	2	2.199483	420129.2	568.2701	tri/oligopeptic	N
501	TPP2	571.3109	1	19.11498	333219.4	570.3036	tri/oligopeptic	N
502	TPP2	288.1748	2	3.037492	261617	574.335	tri/oligopeptic	N
503	TPP2	577.2674	1	20.46273	568620.5	576.2601	tri/oligopeptic	N
504	TPP2	582.3167	1	18.77168	246216.8	581.3094	tri/oligopeptic	N
505	TPP2	292.1379	2	18.20898	195339.5	582.2613	tri/oligopeptic	N
506	TPP2	588.3011	1	17.36698	337762.6	587.2939	tri/oligopeptic	N
507	TPP2	588.3782	1	30.70853	120122	587.3709	tri/oligopeptic	N
508	TPP2	590.3208	1	28.20951	702972.3	589.3136	tri/oligopeptic	N
509	TPP2	296.1618	2	15.76422	232533.1	590.309	tri/oligopeptic	N
510	TPP2	300.6799	2	24.35998	599060.1	599.3453	tri/oligopeptic	N
511	TPP2	604.3369	1	27.89078	264414.7	603.3296	tri/oligopeptic	N
512	TPP2	607.3114	1	24.23044	129052.6	606.3041	tri/oligopeptic	N
513	TPP2	309.696	2	14.9813	414391.5	617.3774	oligopeptide	N
514	TPP2	310.6806	2	2.84185	162421.2	619.3467	oligopeptide	N
515	TPP2	311.1909	2	23.5841	113029.5	620.3672	oligopeptide	N
516	TPP2	314.683	2	24.32401	165250.3	627.3514	oligopeptide	N
517	TPP2	630.2747	1	2.177583	179485.2	629.2674	oligopeptide	N
518	TPP2	630.3483	1	17.08539	412169.4	629.341	oligopeptide	N
519	TPP2	315.6961	2	14.69896	127581.3	629.3777	oligopeptide	N
520	TPP2	316.6077	2	15.65833	520010.6	631.2008	oligopeptide	N
521	TPP2	632.3275	1	12.4944	1195900	631.3202	oligopeptide	N
522	TPP2	316.6675	2	12.50832	322312.8	631.3205	oligopeptide	N
523	TPP2	317.7068	2	2.88095	147682	633.3991	oligopeptide	N
524	TPP2	322.6858	2	14.01734	4288565	643.357	oligopeptide	N
525	TPP2	646.3108	1	20.39733	109521.6	645.3035	oligopeptide	N
526	TPP2	323.6992	2	15.89038	131596.2	645.3839	oligopeptide	N
527	TPP2	325.1663	2	14.87298	965231.7	648.318	oligopeptide	N
528	TPP2	328.6464	2	12.4944	224373.9	655.2782	oligopeptide	N
529	TPP2	657.3582	1	2.1884	140223	656.3509	oligopeptide	N
530	TPP2	329.183	2	2.199483	511208.5	656.3515	oligopeptide	N
531	TPP2	330.1912	2	2.210858	237772.3	658.3679	oligopeptide	N
532	TPP2	330.662	2	17.19422	2703659	659.3095	oligopeptide	N
533	TPP2	335.6412	2	12.50083	106958.2	669.2679	oligopeptide	N
534	TPP2	336.1987	2	24.82946	541674.1	670.3829	oligopeptide	N
535	TPP2	336.2095	2	15.24851	156601.6	670.4044	oligopeptide	N
536	TPP2	675.3129	1	23.21875	148625.9	674.3056	oligopeptide	N
537	TPP2	677.3494	1	15.12153	113689.1	676.3422	oligopeptide	N
538	TPP2	341.6848	2	18.63676	436375.2	681.3551	oligopeptide	N
539	TPP2	352.7039	2	24.3723	1540538	703.3932	oligopeptide	N
540	TPP2	235.8192	3	13.80561	103332.7	704.4358	oligopeptide	N
541	TPP2	706.3797	1	25.2859	186193.5	705.3724	oligopeptide	N
542	TPP2	353.6937	2	25.2859	215434.9	705.3728	oligopeptide	N
543	TPP2	359.7118	2	25.08268	613824.1	717.4091	oligopeptide	N
544	TPP2	364.2407	2	22.27803	362345.7	726.4668	oligopeptide	N
545	TPP2	729.3809	1	16.05263	147660.2	728.3737	oligopeptide	N

546	TPP2	366.7175	2	13.78144	265841.1	731.4204	oligopeptide	N
547	TPP2	367.2088	2	15.74576	474945.4	732.4031	oligopeptide	N
548	TPP2	735.37	1	25.08268	164027.5	734.3627	oligopeptide	N
549	TPP2	371.7176	2	24.99795	2414164	741.4207	oligopeptide	N
550	TPP2	373.1499	2	19.8353	228849	744.2852	oligopeptide	N
551	TPP2	745.4117	1	18.00378	135831.2	744.4044	oligopeptide	N
552	TPP2	373.2097	2	17.94768	106266.7	744.4048	oligopeptide	N
553	TPP2	373.7073	2	12.86345	346472.2	745.4001	oligopeptide	N
554	TPP2	373.7151	2	23.68373	113670.8	745.4156	oligopeptide	N
555	TPP2	375.2072	2	14.40998	626744.1	748.3998	oligopeptide	N
556	TPP2	377.6781	2	2.199483	115584.8	753.3416	oligopeptide	N
557	TPP2	378.1825	2	1.4173	118779.1	754.3504	oligopeptide	N
558	TPP2	758.4078	1	21.11267	107950.5	757.4005	oligopeptide	N
559	TPP2	380.2038	2	27.11254	132292	758.393	oligopeptide	N
560	TPP2	380.7206	2	14.90358	160914.6	759.4266	oligopeptide	N
561	TPP2	386.7333	2	17.88735	499411.8	771.452	oligopeptide	N
562	TPP2	386.7334	2	13.91943	160673.6	771.4523	oligopeptide	N
563	TPP2	392.189	2	18.02023	226019.1	782.3635	oligopeptide	N
564	TPP2	395.2304	2	25.14417	815112.3	788.4462	oligopeptide	N
565	TPP2	799.4228	1	19.7228	150267	798.4156	oligopeptide	N
566	TPP2	400.7127	2	15.83037	227359.2	799.4108	oligopeptide	N
567	TPP2	403.1991	2	14.59439	190671.8	804.3836	oligopeptide	N
568	TPP2	406.7389	2	27.92788	245560.7	811.4632	oligopeptide	N
569	TPP2	412.2231	2	26.89203	133382.1	822.4316	oligopeptide	N
570	TPP2	418.2153	2	27.08448	113306	834.416	oligopeptide	N
571	TPP2	418.7077	2	17.20163	129305.9	835.4009	oligopeptide	N
572	TPP2	279.4743	3	17.20163	349664.3	835.4011	oligopeptide	N
573	TPP2	429.7128	2	20.27624	164365.9	857.411	oligopeptide	N
574	TPP2	859.4555	1	20.87527	198767.9	858.4483	oligopeptide	N
575	TPP2	430.7492	2	25.39282	338840.6	859.4839	oligopeptide	N
576	TPP2	868.5095	1	29.96952	179414.6	867.5022	oligopeptide	N
577	TPP2	435.7309	2	23.5841	2333700	869.4472	oligopeptide	N
578	TPP2	436.2368	2	25.44233	1294525	870.4591	oligopeptide	N
579	TPP2	438.7176	2	16.70711	182872.2	875.4207	oligopeptide	N
580	TPP2	884.8336	1	1.573417	100001.1	883.8263	oligopeptide	N
581	TPP2	447.7411	2	27.07788	138304.3	893.4677	oligopeptide	N
582	TPP2	454.2237	2	16.68283	250922.1	906.4329	oligopeptide	N
583	TPP2	907.4403	1	16.68283	193707.2	906.433	oligopeptide	N
584	TPP2	304.4935	3	15.66727	315265.3	910.4587	oligopeptide	N
585	TPP2	304.8687	3	15.53763	105409.6	911.5842	oligopeptide	N
586	TPP2	457.7701	2	2.199483	183452	913.5256	oligopeptide	N
587	TPP2	305.516	3	2.210858	182323.8	913.5261	oligopeptide	N
588	TPP2	465.2316	2	20.72298	101446.2	928.4487	oligopeptide	N
589	TPP2	931.6278	1	32.61258	119629.2	930.6205	oligopeptide	N
590	TPP2	471.2494	2	23.69223	294638.4	940.4843	oligopeptide	N
591	TPP2	477.3021	2	32.61258	111224.7	952.5897	oligopeptide	N
592	TPP2	478.7154	2	2.348325	125372	955.4163	oligopeptide	N
593	TPP2	487.7289	2	16.5951	102209.7	973.4432	oligopeptide	N
594	TPP2	488.7527	2	22.71693	856062.8	975.4908	oligopeptide	N
595	TPP2	976.4985	1	22.71693	751153.2	975.4912	oligopeptide	N

596	TPP2	489.7421	2	19.43706	843621.7	977.4697	oligopeptide	N
597	TPP2	978.4775	1	19.43706	173654.5	977.4702	oligopeptide	N
598	TPP2	496.7581	2	25.98807	114114.7	991.5017	oligopeptide	N
599	TPP2	500.7317	2	22.71693	223680.5	999.4489	oligopeptide	N
600	TPP2	334.157	3	22.71693	252686.7	999.449	oligopeptide	N
601	TPP2	500.7499	2	21.03171	191217.4	999.4852	oligopeptide	N
602	TPP2	501.7211	2	19.43706	104089	1001.428	oligopeptide	N
603	TPP2	334.8166	3	19.44806	198565	1001.428	oligopeptide	N
604	TPP2	506.7347	2	22.09797	190637.3	1011.455	oligopeptide	N
605	TPP2	506.7681	2	24.02238	363455.7	1011.522	oligopeptide	N
606	TPP2	507.7263	2	22.71693	151024.3	1013.438	oligopeptide	N
607	TPP2	338.8203	3	22.71693	179328.5	1013.439	oligopeptide	N
608	TPP2	339.4799	3	19.43706	118235.2	1015.418	oligopeptide	N
609	TPP2	512.1956	2	24.62472	213821.9	1022.377	oligopeptide	N
610	TPP2	514.3124	2	18.05154	385412.4	1026.61	oligopeptide	N
611	TPP2	343.2111	3	18.05154	1310039	1026.611	oligopeptide	N
612	TPP2	515.3028	2	25.69925	112058.8	1028.591	oligopeptide	N
613	TPP2	516.7345	2	2.166583	189218.8	1031.454	oligopeptide	N
614	TPP2	519.7441	2	21.30231	316798.1	1037.474	oligopeptide	N
615	TPP2	1038.481	1	21.31288	134323.5	1037.474	oligopeptide	N
616	TPP2	525.7499	2	14.22944	247002.7	1049.485	oligopeptide	N
617	TPP2	540.7757	2	23.11044	115493.7	1079.537	oligopeptide	N
618	TPP2	542.2865	2	24.37846	139234	1082.558	oligopeptide	N
619	TPP2	545.2947	2	27.21258	552011.1	1088.575	oligopeptide	N
620	TPP2	1089.582	1	27.22069	227187.3	1088.575	oligopeptide	N
621	TPP2	549.7557	2	22.24138	255316.6	1097.497	oligopeptide	N
622	TPP2	366.8398	3	22.24138	967351	1097.497	oligopeptide	N
623	TPP2	371.8518	3	27.22069	133461.5	1112.534	oligopeptide	N
624	TPP2	560.7695	2	17.53639	155707.4	1119.524	oligopeptide	N
625	TPP2	561.2689	2	16.65863	214792.5	1120.523	oligopeptide	N
626	TPP2	563.2771	2	26.13627	969749	1124.54	oligopeptide	N
627	TPP2	563.8299	2	26.89203	200381.9	1125.645	oligopeptide	N
628	TPP2	383.8396	3	26.1419	180621.5	1148.497	oligopeptide	N
629	TPP2	578.2939	2	15.26479	103295.5	1154.573	oligopeptide	N
630	TPP2	388.5028	3	26.1419	140965.5	1162.487	oligopeptide	N
631	TPP2	588.8118	2	23.49098	201467.4	1175.609	oligopeptide	N
632	TPP2	595.8199	2	25.75923	115551.4	1189.625	oligopeptide	N
633	TPP2	599.3483	2	25.91261	807402.2	1196.682	oligopeptide	N
634	TPP2	618.283	2	16.34216	128646.4	1234.551	oligopeptide	N
635	TPP2	625.2908	2	17.23412	705621.6	1248.567	oligopeptide	N
636	TPP2	630.3092	2	21.68146	229687.9	1258.604	oligopeptide	N
637	TPP2	636.3271	2	24.66195	473545.3	1270.64	oligopeptide	N
638	TPP2	649.8724	2	25.57779	257887.6	1297.73	oligopeptide	N
639	TPP2	651.8283	2	20.888	473567.9	1301.642	oligopeptide	N
640	TPP2	655.8907	2	29.23105	192938.9	1309.767	oligopeptide	N
641	TPP2	670.3548	2	18.33473	254607.8	1338.695	oligopeptide	N
642	TPP2	674.8312	2	18.90288	157804.8	1347.648	oligopeptide	N
643	TPP2	687.347	2	20.65529	525009.9	1372.679	oligopeptide	N
644	TPP2	694.3347	2	24.80748	108919.6	1386.655	oligopeptide	N
645	TPP2	705.8735	2	18.64552	171050.3	1409.733	oligopeptide	N

646	TPP2	472.2317	3	24.81514	101790.3	1413.673	oligopeptide	N
647	TPP2	711.841	2	23.28468	121497.1	1421.667	oligopeptide	N
648	TPP2	712.4326	2	30.734	202858.5	1422.851	oligopeptide	N
649	TPP2	717.8593	2	26.12399	123869.6	1433.704	oligopeptide	N
650	TPP2	722.8657	2	21.04044	373114.9	1443.717	oligopeptide	N
651	TPP2	755.9494	2	30.09504	126121.7	1509.884	oligopeptide	N
652	TPP2	758.3836	2	21.31288	223561	1514.753	oligopeptide	N
653	TPP2	520.5717	3	21.39325	107799.1	1558.693	oligopeptide	N
654	TPP2	435.4843	4	17.48428	162278.5	1737.908	oligopeptide	N
655	TPP2	580.3102	3	17.49148	114463.1	1737.909	oligopeptide	N
656	TPP2	586.9664	3	18.92811	188928.6	1757.877	oligopeptide	N
657	TPP2	739.0584	3	28.37018	180560.4	2214.153	oligopeptide	N
ID	Protein	mz	charge	retentiont	raw.abund	mass	type	Match
2	QM	231.1711	1	5.21135	728053.5	230.1638	di/tripeptide	Y
3	QM	231.1711	1	9.658717	137511.5	230.1639	di/tripeptide	Y
4	QM	231.1711	1	12.02454	144395.5	230.1638	di/tripeptide	Y
5	QM	233.15	1	2.18805	418005.5	232.1427	di/tripeptide	Y
6	QM	233.1502	1	2.753658	179755.5	232.143	di/tripeptide	Y
8	QM	239.1032	1	2.42835	153963.7	238.0959	di/tripeptide	Y
11	QM	245.1866	1	16.44068	370144.7	244.1794	di/tripeptide	Y
13	QM	245.1867	1	14.53846	314807.1	244.1794	di/tripeptide	Y
15	QM	246.1453	1	2.18805	395316.6	245.1381	di/tripeptide	Y
16	QM	246.1455	1	2.635392	160203.4	245.1382	di/tripeptide	Y
20	QM	247.1294	1	2.18805	616068.5	246.1221	di/tripeptide	Y
21	QM	247.1297	1	3.080392	287335.6	246.1224	di/tripeptide	Y
22	QM	247.1297	1	4.681792	764079.8	246.1224	di/tripeptide	Y
27	QM	253.1189	1	2.292533	154937	252.1117	di/tripeptide	Y
30	QM	260.1611	1	2.18805	354691.1	259.1538	di/tripeptide	Y
31	QM	260.1613	1	2.42835	217312.7	259.154	di/tripeptide	Y
34	QM	261.1452	1	2.18805	457723.6	260.1379	di/tripeptide	Y
35	QM	261.1455	1	3.245683	272568.2	260.1382	di/tripeptide	Y
36	QM	261.1454	1	5.392858	305189.4	260.1381	di/tripeptide	Y
37	QM	262.1404	1	1.965242	102662.2	261.1331	di/tripeptide	Y
38	QM	263.1434	1	11.50638	133078.8	262.1361	di/tripeptide	Y
41	QM	267.1346	1	2.4006	175573.1	266.1274	di/tripeptide	Y
42	QM	269.1613	1	2.176683	125615.7	268.154	di/tripeptide	Y
45	QM	281.114	1	2.557267	180888.3	280.1068	di/tripeptide	Y
51	QM	295.13	1	3.238992	248941.3	294.1228	di/tripeptide	Y
56	QM	302.2086	1	15.13646	146504.9	301.2013	tri/oligopeptic	Y
57	QM	302.2083	1	19.85459	279609.6	301.201	tri/oligopeptic	Y
58	QM	303.1671	1	2.18805	181954.1	302.1598	tri/oligopeptic	Y
59	QM	304.1511	1	2.18805	498753.9	303.1438	tri/oligopeptic	Y
63	QM	318.167	1	2.664417	196735.3	317.1598	tri/oligopeptic	Y
64	QM	318.1669	1	2.199558	484767	317.1597	tri/oligopeptic	Y
65	QM	318.1672	1	8.984167	172802.6	317.1599	tri/oligopeptic	Y
73	QM	331.1661	1	2.806017	151408.2	330.1589	tri/oligopeptic	Y
74	QM	331.2852	1	38.47418	104876.4	330.2779	tri/oligopeptic	Y
75	QM	332.1827	1	2.557267	189767.4	331.1754	tri/oligopeptic	Y
76	QM	332.2193	1	3.073592	133430.3	331.212	tri/oligopeptic	Y
80	QM	334.1617	1	2.18805	262212.4	333.1544	tri/oligopeptic	Y

82	QM	342.2403	1	21.77958	144714.9	341.233	tri/oligopeptic	Y
94	QM	363.1563	1	2.18805	128936.6	362.149	tri/oligopeptic	Y
100	QM	376.1724	1	2.165425	105204.1	375.1651	tri/oligopeptic	Y
106	QM	395.0398	1	1.600225	125752.6	394.0325	tri/oligopeptic	Y
112	QM	415.2123	1	36.47458	142327.1	414.205	tri/oligopeptic	Y
134	QM	463.0272	1	1.600225	161615.3	462.02	tri/oligopeptic	Y
141	QM	479.3109	1	37.11703	137564.4	478.3036	tri/oligopeptic	Y
156	QM	515.2921	1	37.11703	110832.6	514.2848	tri/oligopeptic	Y
161	QM	264.175	2	35.27874	101924	526.3355	tri/oligopeptic	Y
165	QM	531.0147	1	1.600225	204076.3	530.0074	tri/oligopeptic	Y
174	QM	274.168	2	35.94152	106009	546.3214	tri/oligopeptic	Y
194	QM	599.0026	1	1.600225	181625.2	597.9953	tri/oligopeptic	Y
218	QM	666.9901	1	1.600225	185240.2	665.9828	oligopeptide	Y
240	QM	734.9777	1	1.600225	156251	733.9704	oligopeptide	Y
258	QM	802.9652	1	1.600225	138766.2	801.958	oligopeptide	Y
296	QM	974.8154	1	1.600225	240696.7	973.8082	oligopeptide	Y
297	QM	990.7897	1	1.611008	144750.1	989.7825	oligopeptide	Y
338	QM	223.1084	1	10.38069	188166.8	222.1011	dipeptide	N
339	QM	223.1085	1	4.891675	118922.8	222.1012	dipeptide	N
340	QM	226.9521	1	1.543025	315868	225.9448	di/tripeptide	N
341	QM	229.1555	1	8.956642	323117.9	228.1482	di/tripeptide	N
342	QM	229.1555	1	12.04725	110059.5	228.1482	di/tripeptide	N
343	QM	229.1555	1	4.911475	135928.8	228.1482	di/tripeptide	N
344	QM	231.1711	1	8.888058	257914	230.1638	di/tripeptide	N
345	QM	232.1296	1	2.18805	170234.6	231.1223	di/tripeptide	N
346	QM	233.1137	1	2.18805	196613.4	232.1064	di/tripeptide	N
347	QM	237.1241	1	11.19039	103955.8	236.1169	di/tripeptide	N
348	QM	239.2009	1	36.01476	121683	238.1936	di/tripeptide	N
349	QM	263.14	1	14.44434	135878.1	262.1327	di/tripeptide	N
350	QM	269.0887	1	2.18805	100651.6	268.0814	di/tripeptide	N
351	QM	272.1612	1	2.280725	106808.4	271.1539	di/tripeptide	N
352	QM	276.1561	1	2.18805	301068.4	275.1488	di/tripeptide	N
353	QM	279.2325	1	37.01757	152621.9	278.2252	di/tripeptide	N
354	QM	281.1142	1	11.55701	311625	280.1069	di/tripeptide	N
355	QM	281.1507	1	5.8785	107740.1	280.1434	di/tripeptide	N
356	QM	284.2591	1	36.02242	215580.1	283.2518	di/tripeptide	N
357	QM	288.1929	1	14.6512	105315.8	287.1856	di/tripeptide	N
358	QM	290.1352	1	2.03365	112886.5	289.128	di/tripeptide	N
359	QM	290.1717	1	2.18805	388015.3	289.1644	di/tripeptide	N
360	QM	292.1301	1	16.44068	130928.8	291.1228	di/tripeptide	N
361	QM	294.9403	1	1.676875	422979.1	293.9331	di/tripeptide	N
362	QM	297.1091	1	2.664417	147524.8	296.1018	di/tripeptide	N
363	QM	300.1929	1	13.37495	104527.9	299.1857	di/tripeptide	N
364	QM	302.1719	1	2.18805	204389	301.1647	tri/oligopeptic	N
365	QM	304.1874	1	2.18805	194104.4	303.1801	tri/oligopeptic	N
366	QM	306.1302	1	1.944867	127688.4	305.1229	tri/oligopeptic	N
367	QM	314.2087	1	12.99117	161690.7	313.2014	tri/oligopeptic	N
368	QM	316.188	1	13.04861	113796.7	315.1807	tri/oligopeptic	N
369	QM	317.1828	1	2.245708	184771	316.1755	tri/oligopeptic	N
370	QM	318.2037	1	14.59879	137011.8	317.1964	tri/oligopeptic	N

371	QM	320.1254	1	17.67406	106195.7	319.1182	tri/oligopeptic	N
372	QM	320.146	1	2.176683	111242.8	319.1387	tri/oligopeptic	N
373	QM	328.2245	1	18.05055	258696.2	327.2172	tri/oligopeptic	N
374	QM	328.2245	1	16.88998	231561.1	327.2172	tri/oligopeptic	N
375	QM	330.2037	1	11.44704	102079.5	329.1964	tri/oligopeptic	N
376	QM	332.1829	1	11.55701	101081.8	331.1756	tri/oligopeptic	N
377	QM	333.1776	1	2.18805	180087.7	332.1703	tri/oligopeptic	N
378	QM	334.1411	1	13.74723	131397.3	333.1339	tri/oligopeptic	N
379	QM	334.1775	1	18.0365	134889.1	333.1702	tri/oligopeptic	N
380	QM	338.3429	1	39.28503	181364.7	337.3356	tri/oligopeptic	N
381	QM	342.24	1	21.25604	119712.3	341.2327	tri/oligopeptic	N
382	QM	342.2403	1	20.73765	110676	341.233	tri/oligopeptic	N
383	QM	343.1986	1	2.18805	134521.6	342.1913	tri/oligopeptic	N
384	QM	346.1987	1	17.06241	108670.6	345.1914	tri/oligopeptic	N
385	QM	347.1571	1	2.03365	100337.2	346.1498	tri/oligopeptic	N
386	QM	348.1411	1	2.022317	100446.8	347.1339	tri/oligopeptic	N
387	QM	348.1776	1	2.222733	384782.5	347.1703	tri/oligopeptic	N
388	QM	350.1727	1	17.50589	111000.6	349.1654	tri/oligopeptic	N
389	QM	360.2144	1	16.85426	123735.6	359.2072	tri/oligopeptic	N
390	QM	360.2145	1	20.45567	200705.7	359.2072	tri/oligopeptic	N
391	QM	361.1726	1	2.18805	234145.8	360.1654	tri/oligopeptic	N
392	QM	362.1934	1	2.257225	226954.2	361.1861	tri/oligopeptic	N
393	QM	362.2091	1	21.36968	113933.4	361.2019	tri/oligopeptic	N
394	QM	364.1883	1	16.67554	105968.5	363.181	tri/oligopeptic	N
395	QM	389.2041	1	2.199558	128851.7	388.1968	tri/oligopeptic	N
396	QM	404.2147	1	2.18805	104664.2	403.2075	tri/oligopeptic	N
397	QM	405.1991	1	2.18805	185159.9	404.1919	tri/oligopeptic	N
398	QM	413.267	1	36.75835	489794.6	412.2597	tri/oligopeptic	N
399	QM	413.2676	1	38.05748	312830.3	412.2603	tri/oligopeptic	N
400	QM	425.1776	1	1.852758	161036.4	424.1703	tri/oligopeptic	N
401	QM	432.2099	1	2.199558	109190.1	431.2026	tri/oligopeptic	N
402	QM	436.3433	1	35.94152	557059.9	435.336	tri/oligopeptic	N
403	QM	436.3438	1	0.019108	257556.2	435.3365	tri/oligopeptic	N
404	QM	441.2988	1	37.35428	173848.4	440.2915	tri/oligopeptic	N
405	QM	446.2254	1	2.18805	127273.9	445.2181	tri/oligopeptic	N
406	QM	450.1631	1	2.18805	112960.4	449.1558	tri/oligopeptic	N
407	QM	463.298	1	37.55515	132748.2	462.2907	tri/oligopeptic	N
408	QM	469.3297	1	37.19694	103915.7	468.3225	tri/oligopeptic	N
409	QM	488.8733	1	1.632492	162513.1	487.866	tri/oligopeptic	N
410	QM	533.455	1	37.39097	109600.9	532.4477	tri/oligopeptic	N
411	QM	395.2053	2	16.56945	100381.6	788.396	oligopeptide	N
ID	Protein	mz	charge	retentiont	raw.abund	mass	type	Match
2	QrtoE	231.1711	1	5.220383	986951.6	230.1638	di/tripeptide	Y
3	QrtoE	231.1711	1	9.671092	323583.1	230.1639	di/tripeptide	Y
4	QrtoE	231.1711	1	12.02031	330636.8	230.1638	di/tripeptide	Y
5	QrtoE	233.15	1	2.186908	404703.4	232.1427	di/tripeptide	Y
6	QrtoE	233.1502	1	2.757242	241287.1	232.143	di/tripeptide	Y
8	QrtoE	239.1032	1	2.414267	151311.8	238.0959	di/tripeptide	Y
10	QrtoE	245.1137	1	1.963575	101505.6	244.1064	di/tripeptide	Y
11	QrtoE	245.1866	1	16.42108	823777.3	244.1794	di/tripeptide	Y

12	QrtoE	245.1869	1	15.88466	127010.1	244.1796	di/tripeptide	Y
13	QrtoE	245.1867	1	14.52336	595998.5	244.1794	di/tripeptide	Y
15	QrtoE	246.1453	1	2.186908	454986.8	245.1381	di/tripeptide	Y
16	QrtoE	246.1455	1	2.640517	269079.6	245.1382	di/tripeptide	Y
20	QrtoE	247.1294	1	2.186908	608208.8	246.1221	di/tripeptide	Y
21	QrtoE	247.1297	1	3.08275	316341.4	246.1224	di/tripeptide	Y
22	QrtoE	247.1297	1	4.689058	751819.1	246.1224	di/tripeptide	Y
23	QrtoE	249.1275	1	2.771358	136513.1	248.1202	di/tripeptide	Y
27	QrtoE	253.1189	1	2.285392	262205.9	252.1117	di/tripeptide	Y
28	QrtoE	253.1192	1	9.3319	114916.7	252.1119	di/tripeptide	Y
30	QrtoE	260.1611	1	2.186908	425273	259.1538	di/tripeptide	Y
30	QrtoE	260.1613	1	2.414267	336702	259.154	di/tripeptide	Y
33	QrtoE	260.1975	1	1.809775	102922.7	259.1902	di/tripeptide	Y
34	QrtoE	261.1452	1	2.186908	498519.1	260.1379	di/tripeptide	Y
35	QrtoE	261.1455	1	3.2497	264382.5	260.1382	di/tripeptide	Y
36	QrtoE	261.1454	1	5.402433	410028.5	260.1381	di/tripeptide	Y
37	QrtoE	262.1404	1	1.963575	127956.1	261.1331	di/tripeptide	Y
38	QrtoE	263.1434	1	11.50003	277303.9	262.1361	di/tripeptide	Y
41	QrtoE	267.1346	1	2.386992	198076.6	266.1274	di/tripeptide	Y
42	QrtoE	269.1613	1	2.176208	204018.9	268.154	di/tripeptide	Y
45	QrtoE	281.114	1	2.553442	171609	280.1068	di/tripeptide	Y
47	QrtoE	288.1929	1	15.92202	111615.2	287.1856	di/tripeptide	Y
48	QrtoE	290.1719	1	2.656808	109784	289.1646	di/tripeptide	Y
51	QrtoE	295.13	1	3.2417	316304.3	294.1228	di/tripeptide	Y
56	QrtoE	302.2086	1	15.12705	181077.3	301.2013	tri/oligopeptic	Y
57	QrtoE	302.2083	1	19.84419	375966.9	301.201	tri/oligopeptic	Y
58	QrtoE	303.1671	1	2.186908	285122.8	302.1598	tri/oligopeptic	Y
59	QrtoE	304.1511	1	2.186908	487485	303.1438	tri/oligopeptic	Y
63	QrtoE	318.167	1	2.667942	194647.1	317.1598	tri/oligopeptic	Y
64	QrtoE	318.1669	1	2.1978	536495.2	317.1597	tri/oligopeptic	Y
65	QrtoE	318.1672	1	8.998808	199752	317.1599	tri/oligopeptic	Y
66	QrtoE	318.1824	1	22.57593	158030.6	317.1752	tri/oligopeptic	Y
70	QrtoE	325.1137	1	1.73225	122376.7	324.1064	tri/oligopeptic	Y
73	QrtoE	331.1661	1	2.806233	172415.4	330.1589	tri/oligopeptic	Y
74	QrtoE	331.2852	1	38.45228	122142.8	330.2779	tri/oligopeptic	Y
75	QrtoE	332.1826	1	2.296467	115312.5	331.1753	tri/oligopeptic	Y
75	QrtoE	332.1827	1	2.553442	214580.6	331.1754	tri/oligopeptic	Y
76	QrtoE	332.2193	1	3.075067	174775.1	331.212	tri/oligopeptic	Y
80	QrtoE	334.1617	1	2.186908	421787.9	333.1544	tri/oligopeptic	Y
82	QrtoE	342.2403	1	21.78168	140627.7	341.233	tri/oligopeptic	Y
90	QrtoE	359.2301	1	2.481033	100917.2	358.2228	tri/oligopeptic	Y
94	QrtoE	363.1563	1	2.186908	105259.9	362.149	tri/oligopeptic	Y
100	QrtoE	376.1724	1	2.165617	125082.6	375.1651	tri/oligopeptic	Y
106	QrtoE	395.0398	1	1.606858	111804.3	394.0325	tri/oligopeptic	Y
112	QrtoE	415.2123	1	36.463	163203.2	414.205	tri/oligopeptic	Y
134	QrtoE	463.0272	1	1.606858	142717.4	462.02	tri/oligopeptic	Y
141	QrtoE	479.3109	1	37.12123	132510.4	478.3036	tri/oligopeptic	Y
144	QrtoE	488.252	1	19.10149	105005	487.2447	tri/oligopeptic	Y
156	QrtoE	515.2921	1	37.12123	108499.7	514.2848	tri/oligopeptic	Y
159	QrtoE	520.2784	1	21.12864	116750.3	519.2711	tri/oligopeptic	Y

161	QrtoE	264.175	2	35.27298	100975.5	526.3355	tri/oligopeptic	Y
165	QrtoE	531.0147	1	1.606858	177922	530.0074	tri/oligopeptic	Y
166	QrtoE	531.3876	1	37.84973	100998.8	530.3804	tri/oligopeptic	Y
174	QrtoE	274.168	2	35.93383	105220.2	546.3214	tri/oligopeptic	Y
194	QrtoE	599.0026	1	1.606858	157075.4	597.9953	tri/oligopeptic	Y
208	QrtoE	643.3321	1	20.74675	129722	642.3248	oligopeptide	Y
218	QrtoE	666.9901	1	1.606858	156421.6	665.9828	oligopeptide	Y
240	QrtoE	734.9777	1	1.606858	135488.6	733.9704	oligopeptide	Y
258	QrtoE	802.9652	1	1.606858	117071.7	801.958	oligopeptide	Y
296	QrtoE	974.8154	1	1.606858	203612.6	973.8082	oligopeptide	Y
297	QrtoE	990.7897	1	1.617158	128964	989.7825	oligopeptide	Y
338	QrtoE	223.1084	1	10.38584	169888.3	222.1011	dipeptide	N
339	QrtoE	223.1085	1	4.895408	239736.9	222.1012	dipeptide	N
340	QrtoE	226.9521	1	1.555258	381308.2	225.9448	di/tripeptide	N
341	QrtoE	229.1555	1	8.972033	298663.2	228.1482	di/tripeptide	N
342	QrtoE	229.1555	1	12.04122	123630.9	228.1482	di/tripeptide	N
343	QrtoE	229.1555	1	4.916442	207513.6	228.1482	di/tripeptide	N
344	QrtoE	231.1711	1	8.903342	316963.6	230.1638	di/tripeptide	N
345	QrtoE	232.1296	1	2.186908	252498.3	231.1223	di/tripeptide	N
346	QrtoE	233.1137	1	2.186908	292497.6	232.1064	di/tripeptide	N
347	QrtoE	233.1504	1	4.028742	100192	232.1431	di/tripeptide	N
348	QrtoE	237.1241	1	11.19468	141152	236.1169	di/tripeptide	N
349	QrtoE	258.1106	1	1.755825	269123	257.1033	di/tripeptide	N
350	QrtoE	263.14	1	14.43127	417894.5	262.1327	di/tripeptide	N
351	QrtoE	276.1196	1	1.893792	107920.7	275.1123	di/tripeptide	N
352	QrtoE	276.1353	1	17.17783	105673.8	275.1281	di/tripeptide	N
353	QrtoE	276.1561	1	2.186908	375068.2	275.1488	di/tripeptide	N
354	QrtoE	279.1712	1	19.89998	181279	278.1639	di/tripeptide	N
355	QrtoE	281.1142	1	11.54666	274394.5	280.1069	di/tripeptide	N
356	QrtoE	281.1507	1	5.890842	174932.8	280.1434	di/tripeptide	N
357	QrtoE	288.1925	1	2.370658	120522.2	287.1853	di/tripeptide	N
358	QrtoE	290.1352	1	2.186908	124080.6	289.1279	di/tripeptide	N
359	QrtoE	290.1352	1	2.031792	121327.7	289.128	di/tripeptide	N
360	QrtoE	290.1717	1	2.186908	449165.6	289.1644	di/tripeptide	N
361	QrtoE	292.1301	1	16.42108	138839.2	291.1228	di/tripeptide	N
362	QrtoE	294.9403	1	1.679075	345628.8	293.9331	di/tripeptide	N
363	QrtoE	295.1301	1	12.61662	130893.8	294.1228	di/tripeptide	N
364	QrtoE	295.1665	1	13.38839	142009.7	294.1592	di/tripeptide	N
365	QrtoE	297.1091	1	2.667942	131528	296.1018	di/tripeptide	N
366	QrtoE	302.1719	1	2.186908	248722.7	301.1647	tri/oligopeptic	N
367	QrtoE	304.1874	1	2.186908	289764	303.1801	tri/oligopeptic	N
368	QrtoE	306.1302	1	1.944675	155628.5	305.1229	tri/oligopeptic	N
369	QrtoE	306.1465	1	10.72147	105555.8	305.1392	tri/oligopeptic	N
370	QrtoE	306.1667	1	2.186908	116134	305.1594	tri/oligopeptic	N
371	QrtoE	311.1247	1	2.731008	116586.9	310.1174	tri/oligopeptic	N
372	QrtoE	314.2087	1	12.96946	202064.4	313.2014	tri/oligopeptic	N
373	QrtoE	316.188	1	13.03022	120041.9	315.1807	tri/oligopeptic	N
374	QrtoE	316.2244	1	19.59053	103719.1	315.2171	tri/oligopeptic	N
375	QrtoE	317.1828	1	2.241467	292327.9	316.1755	tri/oligopeptic	N
376	QrtoE	318.2032	1	2.186908	129908.9	317.1959	tri/oligopeptic	N

377	QrtoE	318.2037	1	14.58613	152682	317.1964	tri/oligopeptic	N
378	QrtoE	320.146	1	2.176208	130559.9	319.1387	tri/oligopeptic	N
379	QrtoE	328.2245	1	18.04731	245469.3	327.2172	tri/oligopeptic	N
380	QrtoE	328.2245	1	16.8836	234405.1	327.2172	tri/oligopeptic	N
381	QrtoE	329.1829	1	2.219667	100008.5	328.1756	tri/oligopeptic	N
382	QrtoE	332.1829	1	11.54666	104661.8	331.1756	tri/oligopeptic	N
383	QrtoE	333.1776	1	2.186908	263529.3	332.1703	tri/oligopeptic	N
384	QrtoE	334.1411	1	13.73191	302490.2	333.1339	tri/oligopeptic	N
385	QrtoE	334.1775	1	18.03326	153881.3	333.1702	tri/oligopeptic	N
386	QrtoE	338.3429	1	39.26554	195080.8	337.3356	tri/oligopeptic	N
387	QrtoE	342.24	1	21.24108	192859.8	341.2327	tri/oligopeptic	N
388	QrtoE	342.2403	1	20.7391	109340.3	341.233	tri/oligopeptic	N
389	QrtoE	343.1986	1	2.186908	195990.7	342.1913	tri/oligopeptic	N
390	QrtoE	346.1987	1	17.05514	127823	345.1914	tri/oligopeptic	N
391	QrtoE	347.1571	1	2.031792	107028.5	346.1498	tri/oligopeptic	N
392	QrtoE	348.1411	1	2.020667	131345.5	347.1339	tri/oligopeptic	N
393	QrtoE	348.1776	1	2.219667	554659.6	347.1703	tri/oligopeptic	N
394	QrtoE	350.1727	1	17.49933	110356.7	349.1654	tri/oligopeptic	N
395	QrtoE	360.2144	1	16.84708	137556.9	359.2072	tri/oligopeptic	N
396	QrtoE	360.2145	1	20.45028	220723.1	359.2072	tri/oligopeptic	N
397	QrtoE	361.1726	1	2.186908	286082.5	360.1654	tri/oligopeptic	N
398	QrtoE	361.2091	1	2.1978	128564.8	360.2018	tri/oligopeptic	N
399	QrtoE	362.1934	1	2.252433	277846.9	361.1861	tri/oligopeptic	N
400	QrtoE	364.136	1	1.9346	119552.7	363.1287	tri/oligopeptic	N
401	QrtoE	374.2043	1	2.186908	113500.6	373.197	tri/oligopeptic	N
402	QrtoE	374.2301	1	14.33977	118061.8	373.2228	tri/oligopeptic	N
403	QrtoE	389.2041	1	2.1978	156087.8	388.1968	tri/oligopeptic	N
404	QrtoE	392.2196	1	20.16593	158439.9	391.2124	tri/oligopeptic	N
405	QrtoE	403.2204	1	14.96088	107361.2	402.2131	tri/oligopeptic	N
406	QrtoE	404.2147	1	2.186908	124783	403.2075	tri/oligopeptic	N
407	QrtoE	405.1991	1	2.186908	220434.5	404.1919	tri/oligopeptic	N
408	QrtoE	405.2619	1	37.26945	220961.6	404.2546	tri/oligopeptic	N
409	QrtoE	413.267	1	36.76018	474236.7	412.2597	tri/oligopeptic	N
410	QrtoE	413.2676	1	38.058	309688.2	412.2603	tri/oligopeptic	N
411	QrtoE	425.1776	1	1.857008	249793.5	424.1703	tri/oligopeptic	N
412	QrtoE	428.3378	1	37.27921	142941.9	427.3305	tri/oligopeptic	N
413	QrtoE	432.2099	1	2.1978	125030.2	431.2026	tri/oligopeptic	N
414	QrtoE	436.3433	1	35.93383	565948.6	435.336	tri/oligopeptic	N
415	QrtoE	436.3438	1	0.022758	251182.7	435.3365	tri/oligopeptic	N
416	QrtoE	441.2988	1	37.36306	174956	440.2915	tri/oligopeptic	N
417	QrtoE	446.2254	1	2.186908	161051.9	445.2181	tri/oligopeptic	N
418	QrtoE	446.2626	1	14.91627	112016.4	445.2553	tri/oligopeptic	N
419	QrtoE	449.288	1	37.23882	157316.5	448.2808	tri/oligopeptic	N
420	QrtoE	462.2203	1	2.176208	114986.4	461.213	tri/oligopeptic	N
421	QrtoE	463.298	1	37.55037	124958.7	462.2907	tri/oligopeptic	N
422	QrtoE	469.3297	1	37.19075	105420.9	468.3225	tri/oligopeptic	N
423	QrtoE	473.2259	1	13.4364	102122	472.2186	tri/oligopeptic	N
424	QrtoE	475.2519	1	2.186908	112753.5	474.2447	tri/oligopeptic	N
425	QrtoE	480.2467	1	17.4917	124179.4	479.2394	tri/oligopeptic	N
426	QrtoE	488.8733	1	1.6376	156457.9	487.866	tri/oligopeptic	N

427	QrtoE	252.1404	2	15.80574	100104.3	502.2662	tri/oligopeptic	N
428	QrtoE	265.1535	2	2.533567	151443.1	528.2924	tri/oligopeptic	N
429	QrtoE	531.2792	1	10.39986	103791.4	530.272	tri/oligopeptic	N
430	QrtoE	533.455	1	37.39205	109131	532.4477	tri/oligopeptic	N
431	QrtoE	572.2947	1	19.65016	157831	571.2874	tri/oligopeptic	N
432	QrtoE	585.3268	1	22.37813	217780.6	584.3195	tri/oligopeptic	N
433	QrtoE	307.1881	2	19.22523	269361.7	612.3616	tri/oligopeptic	N
434	QrtoE	339.1857	2	19.30112	368105.5	676.3569	oligopeptide	N
435	QrtoE	371.2175	2	20.00975	160071.8	740.4204	oligopeptide	N
436	QrtoE	395.2053	2	16.55079	120378.5	788.396	oligopeptide	N
437	QrtoE	424.2204	2	21.15673	103873.6	846.4263	oligopeptide	N
438	QrtoE	460.6674	2	14.23063	171688	919.3202	oligopeptide	N
ID	Protein	mz	charge	retentionti	raw.abund	mass	type	Match
2	Wheat	231.1711	1	5.370767	666071.8	230.1638	di/tripeptide	Y
3	Wheat	231.1711	1	9.6898	652493.9	230.1638	di/tripeptide	Y
4	Wheat	231.1712	1	12.05777	317444.2	230.1639	di/tripeptide	Y
5	Wheat	233.1501	1	2.189133	143073.8	232.1428	di/tripeptide	Y
11	Wheat	245.1867	1	16.43059	1817012	244.1794	di/tripeptide	Y
13	Wheat	245.1866	1	14.54314	1382481	244.1793	di/tripeptide	Y
14	Wheat	245.1869	1	17.68958	145748.2	244.1796	di/tripeptide	Y
15	Wheat	246.1454	1	1.952483	373811.4	245.1381	di/tripeptide	Y
15	Wheat	246.1454	1	2.189133	285700.7	245.1382	di/tripeptide	Y
16	Wheat	246.1456	1	2.661	269029	245.1383	di/tripeptide	Y
20	Wheat	247.1294	1	2.189133	334347.9	246.1222	di/tripeptide	Y
22	Wheat	247.1298	1	4.715917	173441	246.1225	di/tripeptide	Y
23	Wheat	249.1274	1	2.803825	742508.1	248.1201	di/tripeptide	Y
24	Wheat	250.1784	1	34.86167	860455.5	249.1711	di/tripeptide	Y
27	Wheat	253.1189	1	2.306058	336752.7	252.1116	di/tripeptide	Y
30	Wheat	260.1611	1	2.189133	879771.8	259.1539	di/tripeptide	Y
31	Wheat	260.1613	1	2.661	381089.1	259.154	di/tripeptide	Y
34	Wheat	261.1452	1	2.189133	146087.8	260.1379	di/tripeptide	Y
36	Wheat	261.1455	1	5.448867	240124.4	260.1382	di/tripeptide	Y
38	Wheat	263.1434	1	11.52553	284511.4	262.1361	di/tripeptide	Y
42	Wheat	269.1614	1	2.08405	164839.5	268.1541	di/tripeptide	Y
47	Wheat	288.1928	1	16.07955	115631.9	287.1855	di/tripeptide	Y
48	Wheat	290.1718	1	2.389692	333863.8	289.1645	di/tripeptide	Y
50	Wheat	294.1456	1	2.341733	130713	293.1383	di/tripeptide	Y
51	Wheat	295.1301	1	3.341692	158206.3	294.1228	di/tripeptide	Y
57	Wheat	302.2085	1	19.86337	163987.7	301.2012	tri/oligopeptic	Y
58	Wheat	303.1671	1	2.189133	137609.3	302.1599	tri/oligopeptic	Y
64	Wheat	318.167	1	2.200575	190049	317.1597	tri/oligopeptic	Y
70	Wheat	325.1139	1	1.733992	173264.8	324.1066	tri/oligopeptic	Y
72	Wheat	331.1621	1	1.898875	294826	330.1548	tri/oligopeptic	Y
73	Wheat	331.1663	1	2.847033	107225.5	330.159	tri/oligopeptic	Y
79	Wheat	333.1572	1	11.04772	120039.1	332.1499	tri/oligopeptic	Y
82	Wheat	342.2402	1	21.80854	133644.8	341.2329	tri/oligopeptic	Y
86	Wheat	344.2557	1	20.19083	278917.1	343.2484	tri/oligopeptic	Y
89	Wheat	358.2713	1	24.00683	1704230	357.264	tri/oligopeptic	Y
90	Wheat	359.2301	1	2.481733	241363.9	358.2228	tri/oligopeptic	Y
91	Wheat	360.1966	1	18.7834	121297.9	359.1893	tri/oligopeptic	Y

112	Wheat	415.2126	1	36.47357	207144.3	414.2053	tri/oligopeptic	Y
144	Wheat	488.2516	1	19.12215	192371.4	487.2443	tri/oligopeptic	Y
159	Wheat	520.2789	1	21.12522	181091.8	519.2716	tri/oligopeptic	Y
174	Wheat	274.1682	2	35.89189	104890.5	546.3218	tri/oligopeptic	Y
208	Wheat	643.3327	1	20.7454	108512.3	642.3255	oligopeptide	Y
297	Wheat	990.7902	1	1.616408	325861.3	989.7829	oligopeptide	Y
306	Wheat	534.2818	2	22.60479	106278.1	1066.549	oligopeptide	Y
338	Wheat	223.1085	1	5.04975	143675.2	222.1012	dipeptide	N
339	Wheat	223.1085	1	10.4576	100831.6	222.1012	dipeptide	N
340	Wheat	229.1555	1	9.015492	525740.7	228.1482	di/tripeptide	N
341	Wheat	229.1556	1	11.89632	265757.9	228.1483	di/tripeptide	N
342	Wheat	231.1711	1	8.946125	158765.8	230.1638	di/tripeptide	N
343	Wheat	232.1297	1	2.166758	150864.8	231.1224	di/tripeptide	N
344	Wheat	233.1137	1	2.189133	150159.4	232.1064	di/tripeptide	N
345	Wheat	244.1297	1	1.804925	321045.2	243.1224	di/tripeptide	N
346	Wheat	244.1298	1	2.08405	173101.4	243.1225	di/tripeptide	N
347	Wheat	252.9632	1	7.0306	187169.8	251.9559	di/tripeptide	N
348	Wheat	263.1398	1	14.45386	617579.9	262.1325	di/tripeptide	N
349	Wheat	275.1358	1	1.698542	381413.7	274.1285	di/tripeptide	N
350	Wheat	276.1562	1	2.189133	149393.1	275.1489	di/tripeptide	N
351	Wheat	279.1712	1	19.91159	237269	278.164	di/tripeptide	N
352	Wheat	279.2325	1	37.00883	261662.2	278.2252	di/tripeptide	N
353	Wheat	290.1717	1	2.189133	103218.5	289.1644	di/tripeptide	N
354	Wheat	292.1302	1	9.447125	115922.8	291.123	di/tripeptide	N
355	Wheat	292.151	1	1.929767	153365.5	291.1437	di/tripeptide	N
356	Wheat	295.2276	1	37.374	130564.4	294.2203	di/tripeptide	N
357	Wheat	297.1181	1	1.698542	112820.6	296.1108	di/tripeptide	N
358	Wheat	297.2433	1	36.13935	107629.6	296.236	di/tripeptide	N
359	Wheat	300.2178	1	35.24678	106008.4	299.2105	di/tripeptide	N
360	Wheat	302.1719	1	2.282167	158436.5	301.1647	tri/oligopeptic	N
361	Wheat	302.172	1	2.493417	311180.8	301.1647	tri/oligopeptic	N
362	Wheat	302.2086	1	7.277817	219556.9	301.2013	tri/oligopeptic	N
363	Wheat	304.1878	1	6.612292	234280.8	303.1806	tri/oligopeptic	N
364	Wheat	304.2491	1	37.56288	103072.9	303.2418	tri/oligopeptic	N
365	Wheat	313.2381	1	35.80724	174658.4	312.2308	tri/oligopeptic	N
366	Wheat	314.2087	1	13.00219	144195.6	313.2015	tri/oligopeptic	N
367	Wheat	315.1671	1	2.072142	141060.5	314.1598	tri/oligopeptic	N
368	Wheat	315.2538	1	36.13935	339272.3	314.2466	tri/oligopeptic	N
369	Wheat	316.1881	1	13.05888	125323.2	315.1809	tri/oligopeptic	N
370	Wheat	316.2241	1	19.08293	382923.7	315.2168	tri/oligopeptic	N
371	Wheat	317.1829	1	2.189133	186687.9	316.1756	tri/oligopeptic	N
372	Wheat	317.183	1	2.423558	267195.3	316.1757	tri/oligopeptic	N
373	Wheat	318.1495	1	3.652992	324419.7	317.1423	tri/oligopeptic	N
374	Wheat	318.2033	1	2.341733	170113.1	317.196	tri/oligopeptic	N
375	Wheat	318.2034	1	14.59489	186303.6	317.1961	tri/oligopeptic	N
376	Wheat	318.2649	1	37.83672	128218.3	317.2576	tri/oligopeptic	N
377	Wheat	328.2244	1	17.63673	448632.9	327.2172	tri/oligopeptic	N
378	Wheat	328.2245	1	16.8859	195103.8	327.2172	tri/oligopeptic	N
379	Wheat	328.2343	1	15.48358	103910.1	327.227	tri/oligopeptic	N
380	Wheat	329.1831	1	2.544158	222642.1	328.1758	tri/oligopeptic	N

381	Wheat	330.2037	1	11.89632	466463.3	329.1964	tri/oligopeptic	N
382	Wheat	331.1985	1	2.40165	462315.8	330.1913	tri/oligopeptic	N
383	Wheat	331.1989	1	6.36225	141151.6	330.1916	tri/oligopeptic	N
384	Wheat	332.1827	1	9.713975	786844.8	331.1754	tri/oligopeptic	N
385	Wheat	332.2191	1	18.59551	466377.4	331.2118	tri/oligopeptic	N
386	Wheat	333.1778	1	2.189133	250241.8	332.1705	tri/oligopeptic	N
387	Wheat	334.1774	1	18.03428	433548.4	333.1701	tri/oligopeptic	N
388	Wheat	336.1932	1	23.0508	105320.2	335.1859	tri/oligopeptic	N
389	Wheat	337.2358	1	36.13935	142209.1	336.2285	tri/oligopeptic	N
390	Wheat	342.2401	1	18.74299	360119	341.2328	tri/oligopeptic	N
391	Wheat	342.2403	1	21.29544	159116.5	341.233	tri/oligopeptic	N
392	Wheat	343.1987	1	2.189133	114446.5	342.1914	tri/oligopeptic	N
393	Wheat	350.1725	1	17.50723	200268.6	349.1652	tri/oligopeptic	N
394	Wheat	351.0965	1	30.84455	125197.2	350.0893	tri/oligopeptic	N
395	Wheat	357.2143	1	2.211933	255006.3	356.207	tri/oligopeptic	N
396	Wheat	357.2145	1	2.544158	731017.1	356.2072	tri/oligopeptic	N
397	Wheat	357.2148	1	12.48922	206727.4	356.2076	tri/oligopeptic	N
398	Wheat	357.2148	1	13.26202	196346	356.2076	tri/oligopeptic	N
399	Wheat	357.2149	1	14.98073	133218.8	356.2076	tri/oligopeptic	N
400	Wheat	358.1732	1	1.929767	181459.8	357.1659	tri/oligopeptic	N
401	Wheat	358.2961	1	36.2718	164990.5	357.2889	tri/oligopeptic	N
402	Wheat	360.2144	1	17.6224	106532.5	359.2071	tri/oligopeptic	N
403	Wheat	361.2092	1	2.200575	119846.8	360.202	tri/oligopeptic	N
404	Wheat	362.2091	1	21.35706	139629.9	361.2019	tri/oligopeptic	N
405	Wheat	362.2123	1	18.17774	132028.2	361.205	tri/oligopeptic	N
406	Wheat	372.1888	1	1.952483	478441.3	371.1815	tri/oligopeptic	N
407	Wheat	372.1888	1	2.189133	1054658	371.1816	tri/oligopeptic	N
408	Wheat	373.2458	1	18.81772	208859.5	372.2386	tri/oligopeptic	N
409	Wheat	373.2462	1	3.161967	152504.3	372.2389	tri/oligopeptic	N
410	Wheat	373.2462	1	10.8438	129612.4	372.2389	tri/oligopeptic	N
411	Wheat	373.3799	1	37.59178	128892.1	372.3726	tri/oligopeptic	N
412	Wheat	374.2044	1	2.189133	277246.7	373.1971	tri/oligopeptic	N
413	Wheat	376.2275	1	20.34591	1033480	375.2202	tri/oligopeptic	N
414	Wheat	378.1709	1	10.53301	314982.8	377.1637	tri/oligopeptic	N
415	Wheat	380.1832	1	11.38504	841076.7	379.1759	tri/oligopeptic	N
416	Wheat	384.3117	1	37.32066	100712.2	383.3045	tri/oligopeptic	N
417	Wheat	385.2461	1	19.16666	136298.1	384.2388	tri/oligopeptic	N
418	Wheat	389.2041	1	2.189133	173705	388.1968	tri/oligopeptic	N
419	Wheat	391.1991	1	17.2908	371465.6	390.1918	tri/oligopeptic	N
420	Wheat	391.1992	1	14.68162	612563.4	390.1919	tri/oligopeptic	N
421	Wheat	391.2203	1	3.010458	529232.6	390.213	tri/oligopeptic	N
422	Wheat	391.2859	1	1.278858	140136	390.2787	tri/oligopeptic	N
423	Wheat	403.2204	1	11.78113	109819.4	402.2131	tri/oligopeptic	N
424	Wheat	404.1786	1	1.813992	103594.3	403.1714	tri/oligopeptic	N
425	Wheat	413.2674	1	36.74438	667595.9	412.2602	tri/oligopeptic	N
426	Wheat	415.2357	1	27.25548	291771.1	414.2284	tri/oligopeptic	N
427	Wheat	415.3904	1	37.03262	106951.5	414.3831	tri/oligopeptic	N
428	Wheat	428.252	1	15.45089	130038.8	427.2447	tri/oligopeptic	N
429	Wheat	428.2521	1	16.89828	191382.9	427.2449	tri/oligopeptic	N
430	Wheat	428.3379	1	37.27349	172705.5	427.3306	tri/oligopeptic	N

431	Wheat	431.2512	1	14.22363	1320132	430.2439	tri/oligopeptic	N
432	Wheat	220.1192	2	2.024483	124258.6	438.2238	tri/oligopeptic	N
433	Wheat	441.3091	1	24.46216	108320.3	440.3018	tri/oligopeptic	N
434	Wheat	442.2672	1	19.01901	821764.8	441.2599	tri/oligopeptic	N
435	Wheat	443.226	1	2.189133	151337	442.2187	tri/oligopeptic	N
436	Wheat	443.2878	1	17.37238	383505.6	442.2805	tri/oligopeptic	N
437	Wheat	444.2463	1	2.189133	276065.7	443.239	tri/oligopeptic	N
438	Wheat	445.3038	1	25.31478	196396	444.2965	tri/oligopeptic	N
439	Wheat	446.2052	1	20.65447	117262.1	445.1979	tri/oligopeptic	N
440	Wheat	446.208	1	2.317925	486242.9	445.2008	tri/oligopeptic	N
441	Wheat	447.2255	1	17.07339	144960.7	446.2183	tri/oligopeptic	N
442	Wheat	447.2621	1	23.57239	122806	446.2548	tri/oligopeptic	N
443	Wheat	449.2412	1	22.39721	252534.9	448.2339	tri/oligopeptic	N
444	Wheat	454.2677	1	14.49811	210567.1	453.2604	tri/oligopeptic	N
445	Wheat	454.2677	1	17.36105	108123.2	453.2605	tri/oligopeptic	N
446	Wheat	455.3247	1	26.09888	272265.6	454.3174	tri/oligopeptic	N
447	Wheat	455.3249	1	26.81138	123678.6	454.3176	tri/oligopeptic	N
448	Wheat	456.2835	1	13.74982	441219.5	455.2762	tri/oligopeptic	N
449	Wheat	456.2835	1	12.9017	218125.1	455.2763	tri/oligopeptic	N
450	Wheat	459.2209	1	2.024483	474682.9	458.2136	tri/oligopeptic	N
451	Wheat	461.2364	1	2.189133	134329	460.2291	tri/oligopeptic	N
452	Wheat	461.2443	1	20.25217	229034.9	460.237	tri/oligopeptic	N
453	Wheat	469.2416	1	2.200575	727148.3	468.2343	tri/oligopeptic	N
454	Wheat	470.2993	1	15.03004	109214.5	469.292	tri/oligopeptic	N
455	Wheat	471.2575	1	2.282167	699397.9	470.2502	tri/oligopeptic	N
456	Wheat	472.242	1	12.89518	615723.1	471.2348	tri/oligopeptic	N
457	Wheat	472.3642	1	37.23528	109615.2	471.357	tri/oligopeptic	N
458	Wheat	475.2521	1	2.189133	270159.6	474.2448	tri/oligopeptic	N
459	Wheat	478.2313	1	18.56444	274920	477.224	tri/oligopeptic	N
460	Wheat	478.2314	1	10.83716	187399	477.2242	tri/oligopeptic	N
461	Wheat	485.2728	1	2.189133	289038.7	484.2656	tri/oligopeptic	N
462	Wheat	485.2735	1	15.45774	647214.1	484.2662	tri/oligopeptic	N
463	Wheat	485.2737	1	13.70414	359215.9	484.2664	tri/oligopeptic	N
464	Wheat	485.2737	1	12.6889	126047.5	484.2664	tri/oligopeptic	N
465	Wheat	486.2578	1	13.40017	100818.6	485.2505	tri/oligopeptic	N
466	Wheat	488.2523	1	20.36193	249082.7	487.245	tri/oligopeptic	N
467	Wheat	489.2677	1	2.189133	184980	488.2605	tri/oligopeptic	N
468	Wheat	489.2685	1	13.77724	124873.5	488.2612	tri/oligopeptic	N
469	Wheat	489.2724	1	19.77329	387546.3	488.2651	tri/oligopeptic	N
470	Wheat	489.2726	1	17.68958	516003.5	488.2654	tri/oligopeptic	N
471	Wheat	489.3578	1	36.45119	107311.8	488.3505	tri/oligopeptic	N
472	Wheat	494.2731	1	2.189133	175446.7	493.2658	tri/oligopeptic	N
473	Wheat	247.6404	2	2.200575	570464.7	493.2662	tri/oligopeptic	N
474	Wheat	500.2473	1	2.072142	696182.8	499.2401	tri/oligopeptic	N
475	Wheat	502.2634	1	2.189133	177156.3	501.2562	tri/oligopeptic	N
476	Wheat	504.284	1	22.75258	181550.2	503.2767	tri/oligopeptic	N
477	Wheat	505.2675	1	21.91673	102107.4	504.2602	tri/oligopeptic	N
478	Wheat	511.1763	1	1.698542	119708.8	510.1691	tri/oligopeptic	N
479	Wheat	512.3465	1	26.21672	152896.6	511.3392	tri/oligopeptic	N
480	Wheat	519.2578	1	16.43059	1428516	518.2505	tri/oligopeptic	N

481	Wheat	519.2579	1	17.992	112976.9	518.2507	tri/oligopeptic	N
482	Wheat	260.1327	2	16.39715	355146.7	518.2509	tri/oligopeptic	N
483	Wheat	529.2629	1	2.189133	164063.9	528.2556	tri/oligopeptic	N
484	Wheat	529.2995	1	15.53552	475527.6	528.2922	tri/oligopeptic	N
485	Wheat	530.3206	1	21.39409	131369	529.3133	tri/oligopeptic	N
486	Wheat	530.3334	1	36.67673	101064.8	529.3261	tri/oligopeptic	N
487	Wheat	533.2399	1	2.211933	131580.6	532.2326	tri/oligopeptic	N
488	Wheat	535.253	1	14.75852	295984.3	534.2457	tri/oligopeptic	N
489	Wheat	535.253	1	13.18105	159693	534.2457	tri/oligopeptic	N
490	Wheat	539.3202	1	15.54583	641726.8	538.313	tri/oligopeptic	N
491	Wheat	270.1639	2	15.54583	537548.5	538.3132	tri/oligopeptic	N
492	Wheat	541.3356	1	22.84101	1561098	540.3283	tri/oligopeptic	N
493	Wheat	543.2466	1	16.70721	804114.4	542.2393	tri/oligopeptic	N
494	Wheat	543.2947	1	26.16046	268496.6	542.2874	tri/oligopeptic	N
495	Wheat	545.2948	1	14.2172	246477.6	544.2875	tri/oligopeptic	N
496	Wheat	547.2743	1	13.9547	107045.5	546.267	tri/oligopeptic	N
497	Wheat	556.2741	1	2.189133	336390.4	555.2669	tri/oligopeptic	N
498	Wheat	278.6593	2	13.36025	362918.8	555.304	tri/oligopeptic	N
499	Wheat	556.3114	1	13.36661	245003.2	555.3041	tri/oligopeptic	N
500	Wheat	559.3109	1	15.86658	306824.4	558.3036	tri/oligopeptic	N
501	Wheat	566.2948	1	2.200575	195615.8	565.2876	tri/oligopeptic	N
502	Wheat	567.8937	1	1.616408	141594.5	566.8864	tri/oligopeptic	N
503	Wheat	285.1875	2	22.05974	203605.9	568.3605	tri/oligopeptic	N
504	Wheat	569.368	1	22.05974	284404.9	568.3608	tri/oligopeptic	N
505	Wheat	291.667	2	15.9513	143288.8	581.3195	tri/oligopeptic	N
506	Wheat	582.3269	1	15.9513	155971.2	581.3196	tri/oligopeptic	N
507	Wheat	584.3426	1	19.2453	180708.2	583.3353	tri/oligopeptic	N
508	Wheat	585.3263	1	20.26817	466602.7	584.319	tri/oligopeptic	N
509	Wheat	587.2799	1	2.166758	208261.3	586.2726	tri/oligopeptic	N
510	Wheat	588.3419	1	20.59639	315050.1	587.3347	tri/oligopeptic	N
511	Wheat	597.3006	1	2.189133	474023.8	596.2933	tri/oligopeptic	N
512	Wheat	299.1541	2	2.200575	112194	596.2936	tri/oligopeptic	N
513	Wheat	598.358	1	14.68162	171679.8	597.3507	tri/oligopeptic	N
514	Wheat	299.6827	2	14.68162	255721.8	597.3508	tri/oligopeptic	N
515	Wheat	598.8385	1	1.6396	103937.8	597.8313	tri/oligopeptic	N
516	Wheat	300.1515	2	14.61748	148261	598.2885	tri/oligopeptic	N
517	Wheat	599.3162	1	2.317925	165411.7	598.3089	tri/oligopeptic	N
518	Wheat	300.1621	2	2.3299	102096.9	598.3096	tri/oligopeptic	N
519	Wheat	600.2684	1	18.87723	385201.8	599.2611	tri/oligopeptic	N
520	Wheat	601.3318	1	2.189133	106272.4	600.3245	tri/oligopeptic	N
521	Wheat	601.3369	1	23.87813	148753.6	600.3296	tri/oligopeptic	N
522	Wheat	301.1721	2	23.89423	128714.8	600.3297	tri/oligopeptic	N
523	Wheat	605.3164	1	14.23678	209380.3	604.3091	tri/oligopeptic	N
524	Wheat	303.6488	2	16.26488	596243.9	605.2831	tri/oligopeptic	N
525	Wheat	606.2903	1	16.27448	499820.9	605.2831	tri/oligopeptic	N
526	Wheat	303.6542	2	2.095875	136376.8	605.2939	tri/oligopeptic	N
527	Wheat	612.2798	1	19.58876	101095	611.2726	tri/oligopeptic	N
528	Wheat	613.3326	1	14.35231	357241.6	612.3253	tri/oligopeptic	N
529	Wheat	307.17	2	14.35921	294243.2	612.3254	tri/oligopeptic	N
530	Wheat	613.333	1	13.52729	109205.2	612.3258	tri/oligopeptic	N

531	Wheat	307.1702	2	13.51691	196226.8	612.3258	tri/oligopeptic	N
532	Wheat	307.1702	2	12.02376	114766.5	612.3259	tri/oligopeptic	N
533	Wheat	615.3123	1	2.189133	199222.2	614.3051	oligopeptide	N
534	Wheat	616.3112	1	20.36193	707698.7	615.3039	oligopeptide	N
535	Wheat	616.3114	1	17.232	235901.3	615.3041	oligopeptide	N
536	Wheat	616.3114	1	18.19935	352588.1	615.3041	oligopeptide	N
537	Wheat	308.6593	2	17.19618	299018.8	615.3041	oligopeptide	N
538	Wheat	308.6594	2	18.18406	351267.7	615.3042	oligopeptide	N
539	Wheat	309.6671	2	15.63941	150764.4	617.3196	oligopeptide	N
540	Wheat	618.3269	1	15.64733	118151.2	617.3196	oligopeptide	N
541	Wheat	622.2852	1	14.66581	191051.5	621.2779	oligopeptide	N
542	Wheat	313.176	2	2.189133	112583.4	624.3374	oligopeptide	N
543	Wheat	628.3065	1	2.189133	362314.6	627.2993	oligopeptide	N
544	Wheat	315.1566	2	14.58643	1071877	628.2987	oligopeptide	N
545	Wheat	315.628	2	16.24348	123795	629.2415	oligopeptide	N
546	Wheat	632.3433	1	24.75281	148220.8	631.336	oligopeptide	N
547	Wheat	633.3228	1	2.938692	160933.8	632.3155	oligopeptide	N
548	Wheat	635.8814	1	1.616408	145587.3	634.8741	oligopeptide	N
549	Wheat	319.1491	2	14.35231	131183.5	636.2837	oligopeptide	N
550	Wheat	647.317	1	16.4071	476929.6	646.3097	oligopeptide	N
551	Wheat	324.1622	2	16.4071	1237269	646.3099	oligopeptide	N
552	Wheat	324.6545	2	17.05995	146983.3	647.2945	oligopeptide	N
553	Wheat	652.3374	1	23.26678	208181.8	651.3301	oligopeptide	N
554	Wheat	326.7246	2	15.54583	250310.4	651.4347	oligopeptide	N
555	Wheat	655.3798	1	17.6224	114142.5	654.3725	oligopeptide	N
556	Wheat	328.1936	2	17.6224	228989.7	654.3727	oligopeptide	N
557	Wheat	328.693	2	26.44917	774176.2	655.3715	oligopeptide	N
558	Wheat	656.379	1	26.44187	756193.9	655.3717	oligopeptide	N
559	Wheat	329.1725	2	21.87318	692177.9	656.3305	oligopeptide	N
560	Wheat	657.3379	1	21.85995	208129.5	656.3306	oligopeptide	N
561	Wheat	668.6209	1	37.49818	190141.1	667.6136	oligopeptide	N
562	Wheat	670.3537	1	2.189133	308069.2	669.3465	oligopeptide	N
563	Wheat	335.6806	2	2.223333	116033.3	669.3465	oligopeptide	N
564	Wheat	336.1412	2	16.43059	313631.2	670.2679	oligopeptide	N
565	Wheat	672.338	1	26.08382	307226.8	671.3308	oligopeptide	N
566	Wheat	336.6727	2	26.09888	142789.7	671.3308	oligopeptide	N
567	Wheat	341.1517	2	21.85995	102231	680.2889	oligopeptide	N
568	Wheat	341.2017	2	15.90067	149730.3	680.3888	oligopeptide	N
569	Wheat	684.3329	1	2.189133	184202.7	683.3256	oligopeptide	N
570	Wheat	343.1359	2	16.43059	150965.2	684.2572	oligopeptide	N
571	Wheat	344.1783	2	18.24824	263569	686.342	oligopeptide	N
572	Wheat	688.3695	1	23.96225	215349.9	687.3623	oligopeptide	N
573	Wheat	692.3274	1	14.6904	254333.3	691.3201	oligopeptide	N
574	Wheat	346.6676	2	14.71693	133914.9	691.3207	oligopeptide	N
575	Wheat	346.6913	2	2.5241	278957.1	691.3681	oligopeptide	N
576	Wheat	348.2096	2	22.89433	102082.7	694.4047	oligopeptide	N
577	Wheat	348.2153	2	15.20952	100542.4	694.4161	oligopeptide	N
578	Wheat	350.694	2	22.4133	230535.7	699.3735	oligopeptide	N
579	Wheat	703.3434	1	18.79538	966370.7	702.3361	oligopeptide	N
580	Wheat	352.1755	2	18.80696	186023.8	702.3365	oligopeptide	N

581	Wheat	708.5136	1	37.36333	841671.1	707.5063	oligopeptide	N
582	Wheat	355.6968	2	17.5135	198417	709.3791	oligopeptide	N
583	Wheat	355.6969	2	16.82491	135712.5	709.3793	oligopeptide	N
584	Wheat	356.1473	2	16.92817	157478	710.2801	oligopeptide	N
585	Wheat	357.1863	2	21.08953	132958.5	712.358	oligopeptide	N
586	Wheat	364.1544	2	18.79538	177207.6	726.2943	oligopeptide	N
587	Wheat	364.1915	2	2.3299	108193.2	726.3685	oligopeptide	N
588	Wheat	728.3275	1	19.09289	241918.1	727.3202	oligopeptide	N
589	Wheat	364.6915	2	22.87449	109053.9	727.3685	oligopeptide	N
590	Wheat	729.3955	1	20.33465	101898	728.3882	oligopeptide	N
591	Wheat	365.2017	2	20.35348	292306.3	728.3889	oligopeptide	N
592	Wheat	371.1995	2	14.647	242381.9	740.3845	oligopeptide	N
593	Wheat	371.1998	2	13.87792	298785.6	740.3851	oligopeptide	N
594	Wheat	742.3492	1	2.189133	102689.7	741.3419	oligopeptide	N
595	Wheat	372.6889	2	19.5179	307268.3	743.3633	oligopeptide	N
596	Wheat	372.6889	2	20.3054	273835.8	743.3633	oligopeptide	N
597	Wheat	372.689	2	18.16271	280954.5	743.3635	oligopeptide	N
598	Wheat	375.6996	2	2.472525	119161	749.3847	oligopeptide	N
599	Wheat	376.2069	2	25.81879	107051	750.3991	oligopeptide	N
600	Wheat	376.7019	2	20.5231	628331.6	751.3893	oligopeptide	N
601	Wheat	377.1897	2	15.05987	100714.6	752.3648	oligopeptide	N
602	Wheat	377.2044	2	2.189133	114303.3	752.3942	oligopeptide	N
603	Wheat	756.3916	1	14.70883	106843.9	755.3843	oligopeptide	N
604	Wheat	378.6996	2	14.71693	185900.8	755.3846	oligopeptide	N
605	Wheat	384.7177	2	16.04879	217117.6	767.4209	oligopeptide	N
606	Wheat	386.7103	2	16.61057	175171.3	771.406	oligopeptide	N
607	Wheat	386.7103	2	15.70572	247907.6	771.406	oligopeptide	N
608	Wheat	388.1919	2	16.54799	166706.8	774.3692	oligopeptide	N
609	Wheat	389.7281	2	18.76152	231472	777.4416	oligopeptide	N
610	Wheat	392.2047	2	2.306058	229331.6	782.3948	oligopeptide	N
611	Wheat	394.7079	2	12.6889	118919.8	787.4012	oligopeptide	N
612	Wheat	396.736	2	19.81438	310104.4	791.4574	oligopeptide	N
613	Wheat	397.7489	2	19.01901	1638227	793.4833	oligopeptide	N
614	Wheat	399.2128	2	15.73055	694779.1	796.4111	oligopeptide	N
615	Wheat	400.702	2	19.62874	548263	799.3894	oligopeptide	N
616	Wheat	800.3968	1	19.62874	220442.9	799.3895	oligopeptide	N
617	Wheat	400.7024	2	24.40623	135786.7	799.3902	oligopeptide	N
618	Wheat	404.185	2	2.317925	118664.5	806.3555	oligopeptide	N
619	Wheat	406.2387	2	20.33465	195664.7	810.4629	oligopeptide	N
620	Wheat	408.2076	2	18.51542	193811.5	814.4007	oligopeptide	N
621	Wheat	408.7179	2	20.63295	272643.8	815.4213	oligopeptide	N
622	Wheat	410.721	2	14.20928	196196.6	819.4274	oligopeptide	N
623	Wheat	411.1917	2	15.72163	135450.9	820.3689	oligopeptide	N
624	Wheat	412.2388	2	22.53373	521375.6	822.463	oligopeptide	N
625	Wheat	412.239	2	19.31338	164808.4	822.4634	oligopeptide	N
626	Wheat	412.6809	2	19.63558	114284.3	823.3473	oligopeptide	N
627	Wheat	416.2049	2	18.86862	395090	830.3952	oligopeptide	N
628	Wheat	416.205	2	16.21573	119263.5	830.3954	oligopeptide	N
629	Wheat	416.205	2	20.19791	216267.4	830.3954	oligopeptide	N
630	Wheat	419.7262	2	14.20339	756009.2	837.4379	oligopeptide	N

631	Wheat	420.1766	2	16.46486	195854.1	838.3386	oligopeptide	N
632	Wheat	421.2154	2	19.495	266485.4	840.4162	oligopeptide	N
633	Wheat	421.2157	2	21.30374	162879.2	840.4169	oligopeptide	N
634	Wheat	421.7133	2	22.14129	100600.3	841.4121	oligopeptide	N
635	Wheat	421.7257	2	20.59639	220860.4	841.4369	oligopeptide	N
636	Wheat	423.2129	2	18.28563	375254.2	844.4113	oligopeptide	N
637	Wheat	424.6872	2	16.53934	116119.8	847.3599	oligopeptide	N
638	Wheat	426.2256	2	18.88708	149466.7	850.4367	oligopeptide	N
639	Wheat	428.1996	2	17.97488	268560.5	854.3846	oligopeptide	N
640	Wheat	429.2128	2	16.58339	114335.8	856.4111	oligopeptide	N
641	Wheat	429.2313	2	22.58882	133337.9	856.448	oligopeptide	N
642	Wheat	870.789	1	1.6396	113043.8	869.7818	oligopeptide	N
643	Wheat	436.7182	2	17.95167	833911	871.4218	oligopeptide	N
644	Wheat	444.2183	2	23.84257	411345.3	886.422	oligopeptide	N
645	Wheat	887.4294	1	23.84885	103265	886.4221	oligopeptide	N
646	Wheat	446.2703	2	23.9556	165754.1	890.5261	oligopeptide	N
647	Wheat	447.7574	2	17.47848	467439.3	893.5003	oligopeptide	N
648	Wheat	449.1795	2	16.86275	123779	896.3445	oligopeptide	N
649	Wheat	459.7208	2	18.7834	658196.6	917.4271	oligopeptide	N
650	Wheat	460.6671	2	14.26665	169924.2	919.3196	oligopeptide	N
651	Wheat	463.2627	2	26.12448	154960.3	924.5109	oligopeptide	N
652	Wheat	468.2527	2	15.73985	151845.2	934.4908	oligopeptide	N
653	Wheat	470.2522	2	22.15009	459573.7	938.4899	oligopeptide	N
654	Wheat	471.6998	2	18.79538	138735	941.3851	oligopeptide	N
655	Wheat	471.7397	2	21.02148	217689.3	941.4648	oligopeptide	N
656	Wheat	473.7424	2	17.47848	109510.6	945.4703	oligopeptide	N
657	Wheat	476.2682	2	19.47519	379594.4	950.5219	oligopeptide	N
658	Wheat	477.7576	2	23.07053	596393.3	953.5007	oligopeptide	N
659	Wheat	479.2554	2	16.0377	126481.6	956.4962	oligopeptide	N
660	Wheat	480.2344	2	20.19791	192942	958.4543	oligopeptide	N
661	Wheat	480.7434	2	19.01206	107312.4	959.4723	oligopeptide	N
662	Wheat	485.2448	2	19.28934	624467.8	968.4751	oligopeptide	N
663	Wheat	487.2422	2	19.84766	267392.7	972.4699	oligopeptide	N
664	Wheat	491.7422	2	16.29992	911125.2	981.4698	oligopeptide	N
665	Wheat	505.271	2	19.17697	115631.4	1008.527	oligopeptide	N
666	Wheat	506.779	2	24.44628	415070.7	1011.543	oligopeptide	N
667	Wheat	510.2999	2	22.51147	159614.5	1018.585	oligopeptide	N
668	Wheat	513.7867	2	25.31478	441640.5	1025.559	oligopeptide	N
669	Wheat	516.2637	2	23.40999	162552.1	1030.513	oligopeptide	N
670	Wheat	523.7503	2	18.80696	330986.5	1045.486	oligopeptide	N
671	Wheat	524.7947	2	18.62988	140753.2	1047.575	oligopeptide	N
672	Wheat	355.5309	3	18.06803	187312.9	1063.571	oligopeptide	N
673	Wheat	541.7874	2	21.67182	223329.1	1081.56	oligopeptide	N
674	Wheat	541.7874	2	23.43084	159664.4	1081.56	oligopeptide	N
675	Wheat	364.8663	3	18.59551	363299	1091.577	oligopeptide	N
676	Wheat	548.8702	2	1.627925	117107.5	1095.726	oligopeptide	N
677	Wheat	549.2742	2	19.61592	182450	1096.534	oligopeptide	N
678	Wheat	552.7463	2	17.85991	128433.4	1103.478	oligopeptide	N
679	Wheat	557.2907	2	21.79439	160892.6	1112.567	oligopeptide	N
680	Wheat	559.8028	2	18.90566	202553.9	1117.591	oligopeptide	N

681	Wheat	561.7335	2	15.59232	100407	1121.452	oligopeptide	N
682	Wheat	563.3218	2	27.99865	115393.8	1124.629	oligopeptide	N
683	Wheat	574.283	2	23.16401	245753.4	1146.551	oligopeptide	N
684	Wheat	582.8063	2	16.07075	184543.3	1163.598	oligopeptide	N
685	Wheat	588.314	2	16.43059	183824.9	1174.614	oligopeptide	N
686	Wheat	596.3115	2	17.50723	220328.2	1190.608	oligopeptide	N
687	Wheat	597.801	2	20.22518	401616.1	1193.587	oligopeptide	N
688	Wheat	597.801	2	19.82518	194385.1	1193.588	oligopeptide	N
689	Wheat	1194.752	1	1.616408	119313.9	1193.745	oligopeptide	N
690	Wheat	598.3301	2	26.85128	120798.4	1194.646	oligopeptide	N
691	Wheat	599.7985	2	19.46938	658090.3	1197.582	oligopeptide	N
692	Wheat	408.1875	3	19.45954	141319.2	1221.541	oligopeptide	N
693	Wheat	613.3039	2	18.80696	390177.6	1224.593	oligopeptide	N
694	Wheat	619.8197	2	16.27448	166253.1	1237.625	oligopeptide	N
695	Wheat	624.8473	2	1.6396	119724.5	1247.68	oligopeptide	N
696	Wheat	627.3096	2	21.1766	100156.6	1252.605	oligopeptide	N
697	Wheat	638.3123	2	22.51147	111815.9	1274.61	oligopeptide	N
698	Wheat	646.8566	2	25.29243	209216.7	1291.699	oligopeptide	N
699	Wheat	661.8306	2	19.5179	137709.1	1321.647	oligopeptide	N
700	Wheat	696.85	2	14.9669	613452.2	1391.686	oligopeptide	N
701	Wheat	701.8601	2	17.44475	160552.1	1401.706	oligopeptide	N
702	Wheat	705.347	2	20.15254	387314.3	1408.679	oligopeptide	N
703	Wheat	472.8885	3	14.97448	155407.7	1415.644	oligopeptide	N
704	Wheat	718.3728	2	21.85995	331598.3	1434.731	oligopeptide	N
705	Wheat	726.8269	2	1.616408	109221.2	1451.639	oligopeptide	N
706	Wheat	732.3763	2	17.0188	250674.8	1462.738	oligopeptide	N
707	Wheat	758.3688	2	25.17736	118855.2	1514.723	oligopeptide	N
708	Wheat	776.3843	2	19.62874	119545.5	1550.754	oligopeptide	N
709	Wheat	794.8146	2	1.616408	106007	1587.615	oligopeptide	N
710	Wheat	804.4051	2	26.43405	153814.7	1606.796	oligopeptide	N
711	Wheat	896.7969	2	1.627925	111893	1791.579	oligopeptide	N
712	Wheat	922.8025	2	1.616408	395674.2	1843.59	oligopeptide	N
713	Wheat	641.9863	3	25.52629	180353.8	1922.937	oligopeptide	N
714	Wheat	678.3501	3	25.77462	168916.5	2032.028	oligopeptide	N
715	Wheat	683.3396	3	23.9556	158281	2046.997	oligopeptide	N
716	Wheat	1024.783	2	1.616408	162879.4	2047.552	oligopeptide	N
717	Wheat	684.6727	3	23.2907	102251.8	2050.996	oligopeptide	N
718	Wheat	692.3533	3	24.38337	108215.4	2074.038	oligopeptide	N
719	Wheat	702.9715	3	23.11389	137250.6	2105.893	oligopeptide	N
720	Wheat	717.0238	3	24.4759	716765.5	2148.05	oligopeptide	N
721	Wheat	724.6817	3	24.4759	150359.9	2171.023	oligopeptide	N
722	Wheat	1092.77	2	1.616408	137542.8	2183.526	oligopeptide	N
723	Wheat	745.6577	3	23.0574	113453.7	2233.951	oligopeptide	N
724	Wheat	753.387	3	25.68066	138182.5	2257.139	oligopeptide	N
725	Wheat	1160.757	2	1.605	103111.3	2319.5	oligopeptide	N
726	Wheat	778.7185	3	26.11214	117383.6	2333.134	oligopeptide	N
727	Wheat	821.4131	3	27.24807	155953.6	2461.217	oligopeptide	N
728	Wheat	833.4161	3	24.35449	103407.9	2497.227	oligopeptide	N
729	Wheat	911.1304	3	28.61151	171073.2	2730.369	oligopeptide	N
ID	Protein	mz	charge	retentiont	raw.abund	mass	type	Match

2	Corn	231.1712	1	5.396017	158307.3	230.1639	di/tripeptide	Y
3	Corn	231.1712	1	9.687583	1375387	230.1639	di/tripeptide	Y
4	Corn	231.1709	1	12.05802	504631	230.1636	di/tripeptide	Y
5	Corn	233.1501	1	2.193917	628819.8	232.1428	di/tripeptide	Y
6	Corn	233.1502	1	2.79675	348150.9	232.1429	di/tripeptide	Y
11	Corn	245.1867	1	16.43508	690402.1	244.1794	di/tripeptide	Y
12	Corn	245.1869	1	15.90157	119932.8	244.1796	di/tripeptide	Y
13	Corn	245.1868	1	14.56174	236287.6	244.1795	di/tripeptide	Y
14	Corn	245.187	1	17.69018	706226.6	244.1797	di/tripeptide	Y
15	Corn	246.1454	1	1.949375	508336.1	245.1381	di/tripeptide	Y
15	Corn	246.1454	1	2.182567	297023.3	245.1382	di/tripeptide	Y
16	Corn	246.1455	1	2.664908	643265.2	245.1382	di/tripeptide	Y
17	Corn	246.1457	1	3.409842	260183	245.1384	di/tripeptide	Y
20	Corn	247.1295	1	2.182567	166699.4	246.1222	di/tripeptide	Y
22	Corn	247.1298	1	4.743933	227432.5	246.1225	di/tripeptide	Y
24	Corn	250.1784	1	34.86191	816965.1	249.1711	di/tripeptide	Y
25	Corn	250.1784	1	37.25553	148185.1	249.1711	di/tripeptide	Y
27	Corn	253.1189	1	2.489325	1404604	252.1116	di/tripeptide	Y
28	Corn	253.1191	1	9.327025	214991.2	252.1118	di/tripeptide	Y
30	Corn	260.161	1	2.193917	2704490	259.1537	di/tripeptide	Y
32	Corn	260.1615	1	3.7618	1519225	259.1542	di/tripeptide	Y
34	Corn	261.1452	1	2.182567	162573.2	260.1379	di/tripeptide	Y
36	Corn	261.1455	1	5.516133	207755.8	260.1382	di/tripeptide	Y
37	Corn	262.1405	1	1.961492	263812.8	261.1332	di/tripeptide	Y
39	Corn	263.1971	1	2.193917	106503.8	262.1898	di/tripeptide	Y
40	Corn	265.1557	1	16.28073	177791.6	264.1484	di/tripeptide	Y
42	Corn	269.1614	1	2.182567	481306	268.1541	di/tripeptide	Y
46	Corn	281.1507	1	7.137492	117675.9	280.1434	di/tripeptide	Y
47	Corn	288.1929	1	15.94198	197663.6	287.1856	di/tripeptide	Y
48	Corn	290.1718	1	2.718258	542986.1	289.1645	di/tripeptide	Y
50	Corn	294.1457	1	2.337883	141425.2	293.1384	di/tripeptide	Y
51	Corn	295.1301	1	3.356242	121027.8	294.1228	di/tripeptide	Y
52	Corn	295.1664	1	16.42814	1690716	294.1592	di/tripeptide	Y
57	Corn	302.2084	1	19.85123	255201.7	301.2011	tri/oligopeptic	Y
58	Corn	303.1671	1	2.182567	266528.3	302.1598	tri/oligopeptic	Y
64	Corn	318.1669	1	2.205492	130550.4	317.1596	tri/oligopeptic	Y
65	Corn	318.1672	1	9.149433	324312.8	317.16	tri/oligopeptic	Y
73	Corn	331.1662	1	2.848308	252746.3	330.1589	tri/oligopeptic	Y
80	Corn	334.1619	1	2.193917	108305	333.1546	tri/oligopeptic	Y
94	Corn	363.156	1	2.193917	133228.8	362.1487	tri/oligopeptic	Y
112	Corn	415.2125	1	36.47488	160288.5	414.2052	tri/oligopeptic	Y
141	Corn	479.3111	1	37.21091	118698.6	478.3038	tri/oligopeptic	Y
144	Corn	488.252	1	19.12538	190063.4	487.2447	tri/oligopeptic	Y
151	Corn	500.2889	1	25.18949	152810	499.2817	tri/oligopeptic	Y
159	Corn	520.2785	1	21.18292	102050.8	519.2713	tri/oligopeptic	Y
297	Corn	990.7901	1	1.610975	351365.3	989.7828	oligopeptide	Y
338	Corn	229.1555	1	9.030592	231401.1	228.1482	di/tripeptide	N
339	Corn	229.1556	1	11.96377	4366027	228.1483	di/tripeptide	N
340	Corn	232.1298	1	1.912475	123972.1	231.1225	di/tripeptide	N
341	Corn	233.1504	1	4.096017	332920.7	232.1431	di/tripeptide	N

342	Corn	237.1242	1	11.20312	789891.2	236.1169	di/tripeptide	N
343	Corn	237.1242	1	4.635675	122518.3	236.1169	di/tripeptide	N
344	Corn	261.1203	1	1.69265	173963.5	260.113	di/tripeptide	N
345	Corn	263.1399	1	14.46187	691946.9	262.1327	di/tripeptide	N
346	Corn	263.1433	1	13.13114	232306.1	262.136	di/tripeptide	N
347	Corn	269.1138	1	2.182567	156450.1	268.1065	di/tripeptide	N
348	Corn	269.114	1	2.337883	119950.9	268.1067	di/tripeptide	N
349	Corn	274.1404	1	2.079408	380530	273.1331	di/tripeptide	N
350	Corn	274.1767	1	2.489325	722792.5	273.1694	di/tripeptide	N
351	Corn	274.1768	1	2.275783	281117.8	273.1695	di/tripeptide	N
352	Corn	274.1769	1	2.894767	534145.2	273.1696	di/tripeptide	N
353	Corn	274.177	1	9.687583	241279.1	273.1697	di/tripeptide	N
354	Corn	274.1771	1	6.849683	335612.8	273.1698	di/tripeptide	N
355	Corn	275.1357	1	1.704392	472490.6	274.1284	di/tripeptide	N
356	Corn	276.1561	1	2.182567	254731.3	275.1488	di/tripeptide	N
357	Corn	278.1176	1	2.182567	100959.8	277.1103	di/tripeptide	N
358	Corn	279.135	1	3.783342	175937.2	278.1277	di/tripeptide	N
359	Corn	279.138	1	2.182567	108668	278.1307	di/tripeptide	N
360	Corn	279.1707	1	19.90277	335838.2	278.1634	di/tripeptide	N
361	Corn	279.1715	1	21.02548	473756.7	278.1642	di/tripeptide	N
362	Corn	281.1506	1	6.155742	150799.8	280.1434	di/tripeptide	N
363	Corn	288.1561	1	2.182567	230096.8	287.1488	di/tripeptide	N
364	Corn	288.1925	1	2.240483	109420.2	287.1853	di/tripeptide	N
365	Corn	290.1717	1	2.193917	303802.6	289.1645	di/tripeptide	N
366	Corn	297.118	1	1.704392	131204.9	296.1107	di/tripeptide	N
367	Corn	300.1929	1	12.07529	139994.4	299.1856	di/tripeptide	N
368	Corn	300.193	1	4.027008	133843.1	299.1857	di/tripeptide	N
369	Corn	300.193	1	9.846067	255261.1	299.1857	di/tripeptide	N
370	Corn	301.1515	1	2.121342	190535.9	300.1442	di/tripeptide	N
371	Corn	302.2085	1	10.28011	403052.7	301.2013	tri/oligopeptic	N
372	Corn	302.2086	1	11.14295	229869.5	301.2014	tri/oligopeptic	N
373	Corn	304.1875	1	2.263875	584293.4	303.1802	tri/oligopeptic	N
374	Corn	304.1876	1	2.584183	436108.2	303.1803	tri/oligopeptic	N
375	Corn	310.1404	1	2.182567	545970.4	309.1332	tri/oligopeptic	N
376	Corn	313.1765	1	2.182567	107425.8	312.1692	tri/oligopeptic	N
377	Corn	316.1881	1	13.02993	5200033	315.1808	tri/oligopeptic	N
378	Corn	316.2244	1	16.36875	129879.2	315.2171	tri/oligopeptic	N
379	Corn	317.1828	1	2.193917	666098.9	316.1755	tri/oligopeptic	N
380	Corn	317.183	1	2.760175	388664.2	316.1757	tri/oligopeptic	N
381	Corn	318.2037	1	15.90739	245150.2	317.1965	tri/oligopeptic	N
382	Corn	328.2245	1	18.06751	208407.2	327.2172	tri/oligopeptic	N
383	Corn	328.2245	1	17.80022	289791.7	327.2173	tri/oligopeptic	N
384	Corn	328.2247	1	16.85918	148257.3	327.2174	tri/oligopeptic	N
385	Corn	328.2343	1	15.48559	114213.2	327.227	tri/oligopeptic	N
386	Corn	329.1828	1	2.216942	205386.3	328.1756	tri/oligopeptic	N
387	Corn	331.1985	1	2.193917	305414.6	330.1913	tri/oligopeptic	N
388	Corn	331.1986	1	2.729283	442041.4	330.1913	tri/oligopeptic	N
389	Corn	332.2193	1	19.43598	186956.4	331.212	tri/oligopeptic	N
390	Corn	333.1778	1	2.022767	249091.8	332.1706	tri/oligopeptic	N
391	Corn	342.2402	1	19.85123	12301501	341.233	tri/oligopeptic	N

392	Corn	343.1986	1	2.182567	258281.5	342.1913	tri/oligopeptic	N
393	Corn	343.1988	1	13.38618	1065880	342.1916	tri/oligopeptic	N
394	Corn	344.2465	1	19.85123	245701.3	343.2392	tri/oligopeptic	N
395	Corn	345.2145	1	14.61305	110405.2	344.2073	tri/oligopeptic	N
396	Corn	346.1732	1	1.912475	118890.3	345.1659	tri/oligopeptic	N
397	Corn	346.2352	1	13.55542	165911.2	345.2279	tri/oligopeptic	N
398	Corn	348.1777	1	2.193917	126454.7	347.1704	tri/oligopeptic	N
399	Corn	350.1726	1	12.39429	162205.6	349.1653	tri/oligopeptic	N
400	Corn	350.1728	1	10.41498	132505.9	349.1655	tri/oligopeptic	N
401	Corn	350.1756	1	2.534883	132069.9	349.1683	tri/oligopeptic	N
402	Corn	351.0965	1	30.86048	107204.5	350.0893	tri/oligopeptic	N
403	Corn	357.2146	1	15.02083	1760807	356.2073	tri/oligopeptic	N
404	Corn	359.2305	1	15.67595	182320.5	358.2232	tri/oligopeptic	N
405	Corn	359.2306	1	18.51229	129561.8	358.2233	tri/oligopeptic	N
406	Corn	361.2091	1	2.205492	182828.8	360.2018	tri/oligopeptic	N
407	Corn	361.2093	1	2.374408	144989.5	360.2021	tri/oligopeptic	N
408	Corn	362.1681	1	1.880458	146314.3	361.1608	tri/oligopeptic	N
409	Corn	366.1675	1	10.4215	150271.2	365.1602	tri/oligopeptic	N
410	Corn	372.1888	1	2.182567	129363.5	371.1815	tri/oligopeptic	N
411	Corn	372.1889	1	2.055175	142540.3	371.1817	tri/oligopeptic	N
412	Corn	373.2463	1	18.82	113259.8	372.239	tri/oligopeptic	N
413	Corn	374.2043	1	2.205492	490812.8	373.197	tri/oligopeptic	N
414	Corn	376.188	1	14.71723	105097	375.1807	tri/oligopeptic	N
415	Corn	376.2245	1	22.17804	4468277	375.2172	tri/oligopeptic	N
416	Corn	376.2246	1	25.40067	4434877	375.2173	tri/oligopeptic	N
417	Corn	378.231	1	25.40067	118667.2	377.2237	tri/oligopeptic	N
418	Corn	378.2311	1	22.17804	111013.7	377.2238	tri/oligopeptic	N
419	Corn	387.225	1	2.751233	551356	386.2177	tri/oligopeptic	N
420	Corn	387.2254	1	6.291717	113909	386.2181	tri/oligopeptic	N
421	Corn	387.2978	1	19.85924	114396.3	386.2906	tri/oligopeptic	N
422	Corn	388.1838	1	2.159617	255587.7	387.1765	tri/oligopeptic	N
423	Corn	388.1838	1	1.979608	118602.8	387.1765	tri/oligopeptic	N
424	Corn	388.2199	1	2.193917	716871.1	387.2126	tri/oligopeptic	N
425	Corn	388.2205	1	3.606933	135160.6	387.2132	tri/oligopeptic	N
426	Corn	388.2205	1	7.719858	108796	387.2133	tri/oligopeptic	N
427	Corn	389.2043	1	2.47845	104492.1	388.197	tri/oligopeptic	N
428	Corn	389.2047	1	8.24745	147489.7	388.1975	tri/oligopeptic	N
429	Corn	391.22	1	2.350358	134382.7	390.2127	tri/oligopeptic	N
430	Corn	391.286	1	0.020133	609909.3	390.2787	tri/oligopeptic	N
431	Corn	392.2193	1	19.74492	114768.4	391.212	tri/oligopeptic	N
432	Corn	393.1818	1	11.25363	166960.9	392.1745	tri/oligopeptic	N
433	Corn	399.2616	1	16.40899	118159.5	398.2543	tri/oligopeptic	N
434	Corn	399.2618	1	23.52373	469979.7	398.2545	tri/oligopeptic	N
435	Corn	400.22	1	2.193917	119275	399.2127	tri/oligopeptic	N
436	Corn	402.1993	1	2.182567	299310.5	401.192	tri/oligopeptic	N
437	Corn	403.2206	1	13.83088	111375.7	402.2133	tri/oligopeptic	N
438	Corn	407.1938	1	2.444425	191975.2	406.1865	tri/oligopeptic	N
439	Corn	407.1941	1	11.14295	279338.1	406.1868	tri/oligopeptic	N
440	Corn	407.1941	1	18.31378	368751.4	406.1868	tri/oligopeptic	N
441	Corn	413.2773	1	23.34236	194554.4	412.27	tri/oligopeptic	N

442	Corn	413.2778	1	23.60378	348084.7	412.2705	tri/oligopeptic	N
443	Corn	414.2359	1	2.522992	176117.8	413.2286	tri/oligopeptic	N
444	Corn	414.236	1	14.65268	409086.4	413.2287	tri/oligopeptic	N
445	Corn	415.2568	1	14.16772	261213.8	414.2495	tri/oligopeptic	N
446	Corn	415.2569	1	16.67753	114975.6	414.2496	tri/oligopeptic	N
447	Corn	418.2308	1	2.829542	633497.9	417.2236	tri/oligopeptic	N
448	Corn	425.2774	1	19.41129	168611	424.2701	tri/oligopeptic	N
449	Corn	427.2928	1	21.15314	1076153	426.2855	tri/oligopeptic	N
450	Corn	428.2519	1	14.30353	750787.4	427.2446	tri/oligopeptic	N
451	Corn	429.2727	1	23.96705	114953.6	428.2654	tri/oligopeptic	N
452	Corn	430.2306	1	2.228658	167267.3	429.2233	tri/oligopeptic	N
453	Corn	430.2314	1	13.94725	310405.9	429.2242	tri/oligopeptic	N
454	Corn	430.267	1	14.68648	1103708	429.2598	tri/oligopeptic	N
455	Corn	433.2464	1	23.9258	213494.6	432.2391	tri/oligopeptic	N
456	Corn	433.2673	1	16.38399	114721.6	432.26	tri/oligopeptic	N
457	Corn	436.344	1	0.020133	257768.3	435.3367	tri/oligopeptic	N
458	Corn	439.2931	1	19.90277	800375.1	438.2858	tri/oligopeptic	N
459	Corn	441.3089	1	24.74833	328827.2	440.3016	tri/oligopeptic	N
460	Corn	442.2676	1	13.3633	212568.6	441.2603	tri/oligopeptic	N
461	Corn	442.2679	1	15.72072	103760.3	441.2606	tri/oligopeptic	N
462	Corn	443.2259	1	2.193917	234191.6	442.2186	tri/oligopeptic	N
463	Corn	444.2469	1	11.69911	385719.8	443.2396	tri/oligopeptic	N
464	Corn	445.2415	1	2.182567	151465.3	444.2343	tri/oligopeptic	N
465	Corn	445.2419	1	2.885633	633838.8	444.2346	tri/oligopeptic	N
466	Corn	447.262	1	20.74559	444748.9	446.2547	tri/oligopeptic	N
467	Corn	447.262	1	25.55214	230948.3	446.2547	tri/oligopeptic	N
468	Corn	451.2204	1	14.03378	426214.2	450.2132	tri/oligopeptic	N
469	Corn	453.2728	1	27.5805	231617.1	452.2656	tri/oligopeptic	N
470	Corn	455.3245	1	26.47475	100017.7	454.3172	tri/oligopeptic	N
471	Corn	455.3246	1	26.80086	455916.6	454.3173	tri/oligopeptic	N
472	Corn	455.3248	1	27.76863	175582	454.3176	tri/oligopeptic	N
473	Corn	456.2832	1	22.19738	283522.5	455.2759	tri/oligopeptic	N
474	Corn	456.2833	1	20.49612	474204.5	455.276	tri/oligopeptic	N
475	Corn	459.2571	1	2.182567	615002.9	458.2498	tri/oligopeptic	N
476	Corn	462.2571	1	2.38445	317838.9	461.2498	tri/oligopeptic	N
477	Corn	463.2564	1	22.69029	1019681	462.2491	tri/oligopeptic	N
478	Corn	463.2568	1	19.06212	206891.1	462.2495	tri/oligopeptic	N
479	Corn	463.2572	1	25.77708	108852.7	462.2499	tri/oligopeptic	N
480	Corn	470.298	1	23.12191	7142067	469.2907	tri/oligopeptic	N
481	Corn	470.2987	1	15.02083	1287939	469.2914	tri/oligopeptic	N
482	Corn	235.6531	2	15.01209	269174.8	469.2916	tri/oligopeptic	N
483	Corn	471.2573	1	2.182567	276945.8	470.25	tri/oligopeptic	N
484	Corn	473.2367	1	2.182567	111728.3	472.2294	tri/oligopeptic	N
485	Corn	473.2774	1	22.05129	140183.9	472.2701	tri/oligopeptic	N
486	Corn	475.252	1	2.182567	258107.8	474.2447	tri/oligopeptic	N
487	Corn	475.2523	1	2.522992	134919.3	474.2451	tri/oligopeptic	N
488	Corn	242.6666	2	12.99005	138787.9	483.3187	tri/oligopeptic	N
489	Corn	485.2734	1	12.70335	1144959	484.2662	tri/oligopeptic	N
490	Corn	485.2736	1	11.25363	159230	484.2663	tri/oligopeptic	N
491	Corn	485.2736	1	16.17312	577227.9	484.2664	tri/oligopeptic	N

492	Corn	487.2573	1	29.3331	132183.8	486.25	tri/oligopeptic	N
493	Corn	487.2892	1	15.63426	291668.7	486.2819	tri/oligopeptic	N
494	Corn	489.2682	1	2.5683	111633.7	488.2609	tri/oligopeptic	N
495	Corn	489.2729	1	20.61214	568516.7	488.2656	tri/oligopeptic	N
496	Corn	490.2521	1	14.57228	819257	489.2448	tri/oligopeptic	N
497	Corn	490.2679	1	21.78767	150210.8	489.2606	tri/oligopeptic	N
498	Corn	499.2889	1	14.64181	159545.6	498.2816	tri/oligopeptic	N
499	Corn	499.2892	1	13.47342	467868.3	498.2819	tri/oligopeptic	N
500	Corn	500.3092	1	19.92463	187494.7	499.3019	tri/oligopeptic	N
501	Corn	500.3099	1	23.74262	113619.5	499.3026	tri/oligopeptic	N
502	Corn	501.268	1	2.534883	1140694	500.2607	tri/oligopeptic	N
503	Corn	501.2681	1	15.45965	929826.6	500.2609	tri/oligopeptic	N
504	Corn	501.2682	1	14.72839	776547.8	500.2609	tri/oligopeptic	N
505	Corn	251.138	2	15.46473	109901.3	500.2615	tri/oligopeptic	N
506	Corn	504.2828	1	24.82333	3444882	503.2755	tri/oligopeptic	N
507	Corn	504.2833	1	22.97654	274978.7	503.276	tri/oligopeptic	N
508	Corn	504.2835	1	19.33528	318501.1	503.2762	tri/oligopeptic	N
509	Corn	506.2628	1	15.90739	110675.8	505.2555	tri/oligopeptic	N
510	Corn	254.6264	2	23.11553	147769.4	507.2383	tri/oligopeptic	N
511	Corn	512.3451	1	28.89864	2895232	511.3378	tri/oligopeptic	N
512	Corn	513.3051	1	18.8046	295723.9	512.2978	tri/oligopeptic	N
513	Corn	515.2837	1	2.407683	118520.7	514.2765	tri/oligopeptic	N
514	Corn	515.2838	1	14.71723	343090	514.2765	tri/oligopeptic	N
515	Corn	515.284	1	13.26439	116655	514.2767	tri/oligopeptic	N
516	Corn	515.2841	1	12.03925	350117.8	514.2768	tri/oligopeptic	N
517	Corn	520.2785	1	18.49042	439136.2	519.2712	tri/oligopeptic	N
518	Corn	522.3304	1	19.98531	105382.5	521.3231	tri/oligopeptic	N
519	Corn	526.3253	1	23.09667	1570616	525.318	tri/oligopeptic	N
520	Corn	526.3612	1	28.90485	269459	525.3539	tri/oligopeptic	N
521	Corn	527.1853	1	2.444425	128831.3	526.178	tri/oligopeptic	N
522	Corn	527.3208	1	16.95263	157565.6	526.3135	tri/oligopeptic	N
523	Corn	530.2579	1	2.182567	198903.2	529.2506	tri/oligopeptic	N
524	Corn	531.2613	1	19.38906	137485.3	530.254	tri/oligopeptic	N
525	Corn	531.2615	1	14.23405	270422.2	530.2543	tri/oligopeptic	N
526	Corn	541.3359	1	19.11893	890820.9	540.3286	tri/oligopeptic	N
527	Corn	271.1717	2	19.12538	553041.6	540.3289	tri/oligopeptic	N
528	Corn	542.2945	1	2.240483	309734.3	541.2872	tri/oligopeptic	N
529	Corn	543.3154	1	21.58534	501710	542.3081	tri/oligopeptic	N
530	Corn	543.3154	1	20.04357	105232	542.3082	tri/oligopeptic	N
531	Corn	544.2736	1	2.193917	138879.4	543.2664	tri/oligopeptic	N
532	Corn	549.2685	1	12.86502	153646.1	548.2612	tri/oligopeptic	N
533	Corn	276.66	2	33.84269	102886.4	551.3055	tri/oligopeptic	N
534	Corn	556.3107	1	14.84938	736337.9	555.3034	tri/oligopeptic	N
535	Corn	556.3113	1	12.35729	137749.1	555.304	tri/oligopeptic	N
536	Corn	557.3311	1	19.83533	167236.3	556.3239	tri/oligopeptic	N
537	Corn	559.274	1	2.407683	209712	558.2667	tri/oligopeptic	N
538	Corn	560.3105	1	24.54669	130390.2	559.3032	tri/oligopeptic	N
539	Corn	560.3106	1	20.71625	198781.8	559.3033	tri/oligopeptic	N
540	Corn	560.3216	1	14.42962	284901.4	559.3143	tri/oligopeptic	N
541	Corn	570.3272	1	15.83318	178211.6	569.3199	tri/oligopeptic	N

542	Corn	572.3053	1	2.182567	308430.4	571.2981	tri/oligopeptic	N
543	Corn	572.3465	1	26.21093	1583364	571.3393	tri/oligopeptic	N
544	Corn	575.3209	1	23.35982	549359.4	574.3136	tri/oligopeptic	N
545	Corn	581.3317	1	26.3655	282903.8	580.3244	tri/oligopeptic	N
546	Corn	583.3827	1	28.98068	2769437	582.3754	tri/oligopeptic	N
547	Corn	583.383	1	23.12191	101107.4	582.3757	tri/oligopeptic	N
548	Corn	292.1953	2	28.96868	140773.7	582.3761	tri/oligopeptic	N
549	Corn	584.3421	1	19.70915	858209.8	583.3348	tri/oligopeptic	N
550	Corn	584.3428	1	17.24573	107379.4	583.3355	tri/oligopeptic	N
551	Corn	585.3263	1	19.14884	748268	584.319	tri/oligopeptic	N
552	Corn	586.3209	1	2.193917	324173.3	585.3136	tri/oligopeptic	N
553	Corn	586.3214	1	15.02083	1428076	585.3141	tri/oligopeptic	N
554	Corn	293.6645	2	15.01209	127967	585.3144	tri/oligopeptic	N
555	Corn	587.306	1	16.1594	350305.2	586.2988	tri/oligopeptic	N
556	Corn	590.2959	1	16.04208	122779.8	589.2886	tri/oligopeptic	N
557	Corn	591.3155	1	22.07953	125966.2	590.3082	tri/oligopeptic	N
558	Corn	598.3572	1	23.20908	4265186	597.35	tri/oligopeptic	N
559	Corn	299.6824	2	23.20203	1058825	597.3503	tri/oligopeptic	N
560	Corn	598.3583	1	22.91849	921363.8	597.351	tri/oligopeptic	N
561	Corn	299.6828	2	15.6513	223429.7	597.3511	tri/oligopeptic	N
562	Corn	598.3584	1	15.6447	168191.2	597.3512	tri/oligopeptic	N
563	Corn	299.6829	2	20.23801	546063.5	597.3512	tri/oligopeptic	N
564	Corn	598.3586	1	20.23801	444305.5	597.3513	tri/oligopeptic	N
565	Corn	598.3586	1	18.61213	508694.8	597.3514	tri/oligopeptic	N
566	Corn	299.683	2	18.60463	189103.7	597.3515	tri/oligopeptic	N
567	Corn	300.6437	2	13.00488	786453.5	599.2728	tri/oligopeptic	N
568	Corn	602.2992	1	15.99201	263615.1	601.2919	tri/oligopeptic	N
569	Corn	602.3163	1	2.522992	368182.3	601.3091	tri/oligopeptic	N
570	Corn	602.3167	1	14.44234	717505.6	601.3094	tri/oligopeptic	N
571	Corn	301.6621	2	14.44234	150353.9	601.3096	tri/oligopeptic	N
572	Corn	602.3577	1	25.08481	154119	601.3504	tri/oligopeptic	N
573	Corn	603.3371	1	19.37633	672852.4	602.3298	tri/oligopeptic	N
574	Corn	604.2827	1	18.28582	307615.7	603.2754	tri/oligopeptic	N
575	Corn	605.3308	1	22.01991	3401053	604.3235	tri/oligopeptic	N
576	Corn	605.331	1	23.19293	119274.9	604.3237	tri/oligopeptic	N
577	Corn	303.1693	2	22.01991	3259027	604.324	tri/oligopeptic	N
578	Corn	607.3112	1	20.40947	222176.9	606.304	tri/oligopeptic	N
579	Corn	304.1828	2	19.72981	335145.9	606.351	tri/oligopeptic	N
580	Corn	304.1832	2	17.19853	258879.9	606.3518	tri/oligopeptic	N
581	Corn	305.6435	2	15.01209	173335.7	609.2724	tri/oligopeptic	N
582	Corn	305.6829	2	21.77626	115990.3	609.3513	tri/oligopeptic	N
583	Corn	612.3741	1	21.14709	129768.7	611.3668	tri/oligopeptic	N
584	Corn	612.3743	1	20.75984	141997	611.367	tri/oligopeptic	N
585	Corn	307.1702	2	16.62097	483966.4	612.3259	tri/oligopeptic	N
586	Corn	613.3332	1	16.60424	228256.3	612.3259	tri/oligopeptic	N
587	Corn	613.3939	1	28.95146	336203.4	612.3866	tri/oligopeptic	N
588	Corn	614.3527	1	19.85123	441196.6	613.3454	oligopeptide	N
589	Corn	614.353	1	21.61801	880579.7	613.3457	oligopeptide	N
590	Corn	307.6802	2	21.62668	303047	613.3457	oligopeptide	N
591	Corn	616.3321	1	14.70728	428634.5	615.3248	oligopeptide	N

592	Corn	309.1877	2	25.5411	569429.4	616.3608	oligopeptide	N
593	Corn	617.3681	1	25.5411	666602.9	616.3609	oligopeptide	N
594	Corn	618.2933	1	2.288042	279242.1	617.286	oligopeptide	N
595	Corn	620.2775	1	15.27055	177726.8	619.2703	oligopeptide	N
596	Corn	620.3066	1	13.5986	127026	619.2993	oligopeptide	N
597	Corn	310.657	2	13.59221	103268.6	619.2994	oligopeptide	N
598	Corn	310.6672	2	23.20908	126410.5	619.3199	oligopeptide	N
599	Corn	311.1689	2	28.96868	110217.7	620.3232	oligopeptide	N
600	Corn	311.6614	2	23.20908	476032.3	621.3083	oligopeptide	N
601	Corn	312.638	2	15.01209	104258	623.2614	oligopeptide	N
602	Corn	313.641	2	14.44234	155813.6	625.2675	oligopeptide	N
603	Corn	314.1538	2	22.01058	101571.1	626.2931	oligopeptide	N
604	Corn	627.3487	1	16.17959	196004	626.3414	oligopeptide	N
605	Corn	315.1481	2	22.01058	436918.1	628.2816	oligopeptide	N
606	Corn	629.3281	1	2.193917	123269.7	628.3208	oligopeptide	N
607	Corn	631.3477	1	21.56201	139130.5	630.3404	oligopeptide	N
608	Corn	632.3104	1	19.52863	139073.9	631.3031	oligopeptide	N
609	Corn	632.3432	1	24.77268	367105	631.3359	oligopeptide	N
610	Corn	634.3226	1	21.41064	102500.6	633.3153	oligopeptide	N
611	Corn	318.6561	2	23.20908	274492.3	635.2976	oligopeptide	N
612	Corn	319.1494	2	16.62097	110160.2	636.2843	oligopeptide	N
613	Corn	319.1575	2	2.030925	112645.5	636.3004	oligopeptide	N
614	Corn	319.6594	2	21.62668	144736.8	637.3042	oligopeptide	N
615	Corn	321.1753	2	22.19738	101926.8	640.336	oligopeptide	N
616	Corn	642.3851	1	24.0136	135788.2	641.3778	oligopeptide	N
617	Corn	322.1427	2	22.01991	272867.9	642.2709	oligopeptide	N
618	Corn	643.343	1	14.73782	211957.8	642.3357	oligopeptide	N
619	Corn	322.1753	2	14.73782	207328.1	642.3361	oligopeptide	N
620	Corn	322.1755	2	12.48661	100324.9	642.3365	oligopeptide	N
621	Corn	323.1911	2	15.05633	110406.7	644.3676	oligopeptide	N
622	Corn	646.3216	1	14.88147	345036.8	645.3143	oligopeptide	N
623	Corn	323.6645	2	14.88952	302013.1	645.3145	oligopeptide	N
624	Corn	648.304	1	14.88952	141268.8	647.2967	oligopeptide	N
625	Corn	648.3379	1	18.21343	208803	647.3307	oligopeptide	N
626	Corn	324.6727	2	18.21343	201084.5	647.3308	oligopeptide	N
627	Corn	326.6422	2	23.19293	106396.8	651.2698	oligopeptide	N
628	Corn	655.38	1	18.05075	492222.4	654.3727	oligopeptide	N
629	Corn	328.1936	2	18.05075	252031.3	654.3727	oligopeptide	N
630	Corn	329.1543	2	14.87133	378404.6	656.2941	oligopeptide	N
631	Corn	330.199	2	18.34035	794034.6	658.3834	oligopeptide	N
632	Corn	334.1612	2	20.42355	101293.1	666.3078	oligopeptide	N
633	Corn	673.3543	1	15.93387	164648.5	672.347	oligopeptide	N
634	Corn	673.3953	1	24.16437	151029.2	672.388	oligopeptide	N
635	Corn	677.3279	1	13.3789	192834.6	676.3206	oligopeptide	N
636	Corn	339.1678	2	13.3633	467941.8	676.3211	oligopeptide	N
637	Corn	688.3697	1	23.98862	224659.9	687.3625	oligopeptide	N
638	Corn	698.2517	1	17.48576	259592.2	697.2444	oligopeptide	N
639	Corn	351.1467	2	13.3789	175686.5	700.2789	oligopeptide	N
640	Corn	703.3798	1	23.32184	238260.1	702.3725	oligopeptide	N
641	Corn	352.1937	2	23.32184	194881	702.3729	oligopeptide	N

642	Corn	354.1991	2	25.81663	679004.5	706.3836	oligopeptide	N
643	Corn	356.2248	2	26.13642	1202231	710.435	oligopeptide	N
644	Corn	711.4425	1	26.13642	370493.8	710.4352	oligopeptide	N
645	Corn	718.4169	1	27.19068	101267.1	717.4097	oligopeptide	N
646	Corn	359.7122	2	27.19068	154301.4	717.4098	oligopeptide	N
647	Corn	360.1914	2	18.08528	105958.8	718.3682	oligopeptide	N
648	Corn	360.725	2	23.20908	384169.3	719.4354	oligopeptide	N
649	Corn	361.6085	2	17.48576	147232.4	721.2025	oligopeptide	N
650	Corn	363.1424	2	22.01991	104669	724.2703	oligopeptide	N
651	Corn	363.7123	2	19.58842	370876.5	725.41	oligopeptide	N
652	Corn	363.7123	2	22.97654	408083.8	725.41	oligopeptide	N
653	Corn	368.2039	2	26.14323	192481.4	734.3933	oligopeptide	N
654	Corn	369.1532	2	14.19923	138647.4	736.2919	oligopeptide	N
655	Corn	371.2	2	16.68396	300055.6	740.3854	oligopeptide	N
656	Corn	760.4266	1	23.11553	312462.7	759.4193	oligopeptide	N
657	Corn	380.717	2	23.09667	166966.2	759.4195	oligopeptide	N
658	Corn	767.3855	1	20.97956	104991.1	766.3782	oligopeptide	N
659	Corn	384.1966	2	21.01863	251893.9	766.3787	oligopeptide	N
660	Corn	384.7361	2	21.4909	245696.7	767.4576	oligopeptide	N
661	Corn	385.2155	2	17.21054	260170.3	768.4164	oligopeptide	N
662	Corn	769.4237	1	17.21939	175653.7	768.4164	oligopeptide	N
663	Corn	390.2146	2	24.82333	128089.2	778.4146	oligopeptide	N
664	Corn	392.223	2	19.36508	385366.7	782.4315	oligopeptide	N
665	Corn	783.4389	1	19.36508	241395.9	782.4316	oligopeptide	N
666	Corn	392.7334	2	22.69993	306940.6	783.4523	oligopeptide	N
667	Corn	786.4431	1	25.90492	358052.3	785.4358	oligopeptide	N
668	Corn	393.7252	2	25.91128	371461	785.4359	oligopeptide	N
669	Corn	400.689	2	2.205492	132110.8	799.3634	oligopeptide	N
670	Corn	402.6726	2	16.19513	103214.9	803.3307	oligopeptide	N
671	Corn	404.2021	2	19.36508	110355	806.3896	oligopeptide	N
672	Corn	409.7258	2	21.68743	139314.3	817.437	oligopeptide	N
673	Corn	412.2261	2	15.5482	693335.3	822.4376	oligopeptide	N
674	Corn	413.2469	2	26.60053	110524.4	824.4793	oligopeptide	N
675	Corn	841.3103	1	20.50386	142090	840.303	oligopeptide	N
676	Corn	857.4802	1	26.46451	245611.4	856.4729	oligopeptide	N
677	Corn	429.2438	2	26.43755	527062.4	856.473	oligopeptide	N
678	Corn	870.7886	1	1.634117	121397.1	869.7813	oligopeptide	N
679	Corn	450.2673	2	28.37774	943931.1	898.52	oligopeptide	N
680	Corn	899.5277	1	28.36293	429008.9	898.5204	oligopeptide	N
681	Corn	452.2619	2	27.20595	135405.3	902.5092	oligopeptide	N
682	Corn	454.2352	2	22.87473	201039.7	906.4558	oligopeptide	N
683	Corn	460.2589	2	23.18525	225862.5	918.5033	oligopeptide	N
684	Corn	460.6673	2	14.26923	130321.1	919.3201	oligopeptide	N
685	Corn	473.7544	2	21.95956	1363195	945.4942	oligopeptide	N
686	Corn	946.502	1	21.95956	113751.4	945.4947	oligopeptide	N
687	Corn	478.2555	2	23.74262	132849.3	954.4964	oligopeptide	N
688	Corn	479.7371	2	17.14424	226288.4	957.4596	oligopeptide	N
689	Corn	485.7333	2	21.95183	141419.4	969.452	oligopeptide	N
690	Corn	324.1582	3	21.95956	255105.1	969.4529	oligopeptide	N
691	Corn	485.786	2	28.70022	648638.8	969.5574	oligopeptide	N

692	Corn	970.565	1	28.70022	154372.8	969.5577	oligopeptide	N
693	Corn	485.7864	2	29.42978	120972.9	969.5583	oligopeptide	N
694	Corn	328.8213	3	21.95956	163587.8	983.4421	oligopeptide	N
695	Corn	493.7838	2	29.72158	402074.4	985.5531	oligopeptide	N
696	Corn	986.5606	1	29.71512	139164.9	985.5533	oligopeptide	N
697	Corn	502.7786	2	29.42218	107942.8	1003.543	oligopeptide	N
698	Corn	529.3024	2	30.03608	330984.5	1056.59	oligopeptide	N
699	Corn	1057.598	1	30.02927	103196.7	1056.59	oligopeptide	N
700	Corn	529.3025	2	29.73788	107592.6	1056.591	oligopeptide	N
701	Corn	548.8702	2	1.634117	137416.4	1095.726	oligopeptide	N
702	Corn	565.7491	2	21.85148	144722.6	1129.484	oligopeptide	N
703	Corn	1194.753	1	1.610975	118491.4	1193.746	oligopeptide	N
704	Corn	598.3114	2	19.12538	138468.4	1194.608	oligopeptide	N
705	Corn	609.8198	2	24.71795	211653.5	1217.625	oligopeptide	N
706	Corn	624.8459	2	1.634117	109724.7	1247.677	oligopeptide	N
707	Corn	433.926	3	24.79603	157337.1	1298.756	oligopeptide	N
708	Corn	654.8291	2	26.86219	1063134	1307.644	oligopeptide	N
709	Corn	658.8389	2	1.634117	107272.2	1315.663	oligopeptide	N
710	Corn	691.3515	2	26.13001	1004758	1380.688	oligopeptide	N
711	Corn	692.8334	2	1.634117	128214.7	1383.652	oligopeptide	N
712	Corn	469.2227	3	26.13001	213933.5	1404.646	oligopeptide	N
713	Corn	473.8856	3	26.13001	159725.5	1418.635	oligopeptide	N
714	Corn	726.8266	2	1.622633	128678.8	1451.639	oligopeptide	N
715	Corn	760.8213	2	1.622633	133128.7	1519.628	oligopeptide	N
716	Corn	794.8147	2	1.610975	116345.5	1587.615	oligopeptide	N
717	Corn	828.8083	2	1.634117	125133.6	1655.602	oligopeptide	N
718	Corn	854.8145	2	1.622633	547858.8	1707.615	oligopeptide	N
719	Corn	896.796	2	1.634117	105134.2	1791.577	oligopeptide	N
720	Corn	930.7894	2	1.634117	109844.8	1859.564	oligopeptide	N
721	Corn	964.7838	2	1.622633	105788.4	1927.553	oligopeptide	N
722	Corn	1024.783	2	1.622633	176518.1	2047.552	oligopeptide	N
723	Corn	1092.771	2	1.622633	144535.3	2183.527	oligopeptide	N
ID	Protein	mz	charge	retentiont	raw.abund	mass	type	Match
2	YE	231.171	1	5.497858	1190194	230.1638	di/tripeptide	Y
3	YE	231.1711	1	9.780558	824826.9	230.1639	di/tripeptide	Y
4	YE	231.1712	1	12.10568	680263.8	230.1639	di/tripeptide	Y
5	YE	233.15	1	2.196242	534975.4	232.1427	di/tripeptide	Y
6	YE	233.1501	1	2.812542	229030	232.1428	di/tripeptide	Y
8	YE	239.1031	1	2.456542	172782.6	238.0958	di/tripeptide	Y
9	YE	243.1347	1	5.5521	106941.9	242.1274	di/tripeptide	Y
11	YE	245.1866	1	16.43819	1812308	244.1793	di/tripeptide	Y
12	YE	245.1866	1	15.91131	358027.9	244.1794	di/tripeptide	Y
13	YE	245.1866	1	14.57323	633322.1	244.1793	di/tripeptide	Y
14	YE	245.1867	1	17.69823	352740.7	244.1794	di/tripeptide	Y
15	YE	246.1452	1	2.184975	654701.6	245.138	di/tripeptide	Y
15	YE	246.1453	1	1.945508	126124.8	245.138	di/tripeptide	Y
16	YE	246.1454	1	2.684583	310383.5	245.1381	di/tripeptide	Y
17	YE	246.1456	1	3.444925	339697.1	245.1383	di/tripeptide	Y
20	YE	247.1293	1	2.173933	675307.4	246.122	di/tripeptide	Y
21	YE	247.1296	1	3.217592	237388.8	246.1224	di/tripeptide	Y

22	YE	247.1296	1	4.784458	1096393	246.1223	di/tripeptide	Y
23	YE	249.1273	1	2.832325	300403.4	248.12	di/tripeptide	Y
24	YE	250.1785	1	34.85711	730495.3	249.1712	di/tripeptide	Y
27	YE	253.1188	1	2.309683	229451.8	252.1115	di/tripeptide	Y
27	YE	253.1189	1	2.51905	173233.4	252.1116	di/tripeptide	Y
28	YE	253.1191	1	9.39085	191214.1	252.1118	di/tripeptide	Y
30	YE	260.161	1	2.196242	536679.5	259.1537	di/tripeptide	Y
31	YE	260.1611	1	2.488433	127111.6	259.1539	di/tripeptide	Y
31	YE	260.1612	1	2.690967	194497.5	259.1539	di/tripeptide	Y
32	YE	260.1614	1	3.811275	210822.4	259.1541	di/tripeptide	Y
33	YE	260.1973	1	1.81575	158369.1	259.19	di/tripeptide	Y
33	YE	260.1974	1	2.184975	120896.7	259.1901	di/tripeptide	Y
34	YE	261.145	1	2.196242	501473.2	260.1377	di/tripeptide	Y
35	YE	261.1453	1	3.361192	320402.7	260.138	di/tripeptide	Y
36	YE	261.1452	1	5.5521	759601.3	260.138	di/tripeptide	Y
38	YE	263.1432	1	11.58537	375937.8	262.1359	di/tripeptide	Y
40	YE	265.1554	1	16.29458	252147	264.1482	di/tripeptide	Y
41	YE	267.1345	1	2.435458	282947.4	266.1272	di/tripeptide	Y
42	YE	269.1612	1	2.184975	431557.5	268.154	di/tripeptide	Y
45	YE	281.1139	1	2.602867	163996.2	280.1066	di/tripeptide	Y
47	YE	288.1927	1	15.95877	314291.4	287.1854	di/tripeptide	Y
48	YE	290.1718	1	2.712283	141005.9	289.1645	di/tripeptide	Y
50	YE	294.1454	1	2.355983	181183.1	293.1381	di/tripeptide	Y
51	YE	295.1298	1	3.47915	465745.2	294.1225	di/tripeptide	Y
52	YE	295.166	1	16.43819	356399.5	294.1587	di/tripeptide	Y
56	YE	302.2085	1	15.18645	167422.9	301.2012	tri/oligopeptic	Y
57	YE	302.2083	1	19.87277	468137.9	301.2011	tri/oligopeptic	Y
59	YE	304.1509	1	2.196242	486988.9	303.1436	tri/oligopeptic	Y
61	YE	304.1667	1	19.0076	120995.3	303.1594	tri/oligopeptic	Y
62	YE	316.2244	1	13.94554	121245.8	315.2171	tri/oligopeptic	Y
63	YE	318.1669	1	2.712283	174129	317.1596	tri/oligopeptic	Y
64	YE	318.1667	1	2.207625	687463.6	317.1595	tri/oligopeptic	Y
65	YE	318.1671	1	9.201875	334390	317.1599	tri/oligopeptic	Y
66	YE	318.1826	1	22.56827	187702.9	317.1753	tri/oligopeptic	Y
69	YE	319.1619	1	2.048192	114421.3	318.1547	tri/oligopeptic	Y
71	YE	330.2398	1	18.13513	219673.2	329.2326	tri/oligopeptic	Y
73	YE	331.1661	1	2.864967	207506	330.1588	tri/oligopeptic	Y
75	YE	332.1825	1	2.612583	228661.2	331.1752	tri/oligopeptic	Y
77	YE	332.2192	1	3.256367	224036.5	331.2119	tri/oligopeptic	Y
80	YE	334.1616	1	2.207625	397969	333.1543	tri/oligopeptic	Y
82	YE	342.2403	1	21.78346	227522	341.2331	tri/oligopeptic	Y
85	YE	344.2558	1	21.37145	154503.4	343.2486	tri/oligopeptic	Y
85	YE	344.256	1	21.05839	125248.7	343.2487	tri/oligopeptic	Y
86	YE	344.2558	1	20.18418	201659.1	343.2485	tri/oligopeptic	Y
89	YE	358.2716	1	24.0101	160803.4	357.2643	tri/oligopeptic	Y
92	YE	360.214	1	2.812542	163194.7	359.2067	tri/oligopeptic	Y
94	YE	363.156	1	2.184975	129474	362.1487	tri/oligopeptic	Y
97	YE	374.2302	1	19.3437	101469	373.2229	tri/oligopeptic	Y
98	YE	375.2246	1	2.207625	174458.4	374.2173	tri/oligopeptic	Y
100	YE	376.1725	1	2.196242	101993.8	375.1652	tri/oligopeptic	Y

112	YE	415.2125	1	36.46834	254887.9	414.2052	tri/oligopeptic	Y
141	YE	479.3111	1	36.95858	156796.6	478.3038	tri/oligopeptic	Y
144	YE	488.2522	1	19.14389	184445.2	487.2449	tri/oligopeptic	Y
151	YE	500.2887	1	25.1933	132686.6	499.2814	tri/oligopeptic	Y
159	YE	520.2786	1	21.15384	200469.5	519.2713	tri/oligopeptic	Y
161	YE	264.1753	2	35.27877	108153.1	526.336	tri/oligopeptic	Y
174	YE	274.1682	2	35.9381	111896.5	546.3219	tri/oligopeptic	Y
194	YE	599.0025	1	1.594167	266572.6	597.9953	tri/oligopeptic	Y
218	YE	666.9901	1	1.594167	263443.4	665.9828	oligopeptide	Y
240	YE	734.9776	1	1.605367	235867.1	733.9703	oligopeptide	Y
258	YE	802.9653	1	1.594167	199460.7	801.958	oligopeptide	Y
276	YE	870.9526	1	1.594167	137118.1	869.9453	oligopeptide	Y
296	YE	974.8154	1	1.62775	217186.6	973.8081	oligopeptide	Y
297	YE	990.7897	1	1.616633	161957.3	989.7825	oligopeptide	Y
300	YE	514.9585	2	1.594167	104504.7	1027.902	oligopeptide	Y
302	YE	1042.803	1	1.62775	127040.1	1041.796	oligopeptide	Y
307	YE	548.9522	2	1.594167	110935.5	1095.89	oligopeptide	Y
313	YE	582.9463	2	1.605367	115257	1163.878	oligopeptide	Y
316	YE	616.9402	2	1.605367	110421.4	1231.866	oligopeptide	Y
318	YE	624.9271	2	1.616633	109614.4	1247.84	oligopeptide	Y
321	YE	650.9339	2	1.594167	100357.7	1299.853	oligopeptide	Y
322	YE	658.921	2	1.605367	111240.2	1315.828	oligopeptide	Y
329	YE	692.9148	2	1.605367	102078.4	1383.815	oligopeptide	Y
333	YE	760.9022	2	1.616633	106427.5	1519.79	oligopeptide	Y
337	YE	828.8899	2	1.616633	104260.2	1655.765	oligopeptide	Y
338	YE	223.1084	1	10.50456	187393	222.1011	dipeptide	N
339	YE	223.1084	1	5.159825	253814.7	222.1011	dipeptide	N
340	YE	229.1554	1	5.081875	152400.9	228.1481	di/tripeptide	N
341	YE	229.1554	1	9.090325	256806.2	228.1482	di/tripeptide	N
342	YE	231.171	1	9.030367	424632.2	230.1638	di/tripeptide	N
343	YE	232.1296	1	2.173933	339057.9	231.1223	di/tripeptide	N
344	YE	233.1136	1	2.196242	178585	232.1063	di/tripeptide	N
345	YE	233.1503	1	4.175633	368845.9	232.143	di/tripeptide	N
346	YE	237.1241	1	4.729642	270171.8	236.1168	di/tripeptide	N
347	YE	237.1241	1	11.25327	285473.4	236.1168	di/tripeptide	N
348	YE	262.1195	1	17.0005	106792.1	261.1122	di/tripeptide	N
349	YE	263.1398	1	14.47137	214545.9	262.1325	di/tripeptide	N
350	YE	263.1432	1	13.16478	186436.8	262.1359	di/tripeptide	N
351	YE	265.1555	1	14.77233	428008.7	264.1482	di/tripeptide	N
352	YE	274.1766	1	2.27535	214487.9	273.1693	di/tripeptide	N
353	YE	274.1767	1	2.508467	103371.4	273.1695	di/tripeptide	N
354	YE	276.1195	1	1.902642	136298.4	275.1123	di/tripeptide	N
355	YE	276.1353	1	14.36702	107785.6	275.128	di/tripeptide	N
356	YE	276.1353	1	17.19737	138802.5	275.128	di/tripeptide	N
357	YE	276.156	1	2.196242	289542.3	275.1487	di/tripeptide	N
358	YE	278.1174	1	2.196242	109044	277.1101	di/tripeptide	N
359	YE	279.1711	1	19.9121	548017.5	278.1638	di/tripeptide	N
360	YE	279.1713	1	18.99816	139388.1	278.164	di/tripeptide	N
361	YE	279.1714	1	21.03353	158442.1	278.1641	di/tripeptide	N
362	YE	281.1141	1	11.61453	401297.5	280.1068	di/tripeptide	N

363	YE	281.1506	1	6.300292	286173.9	280.1433	di/tripeptide	N
364	YE	288.1928	1	9.610658	122052.2	287.1855	di/tripeptide	N
365	YE	288.1928	1	14.92691	118236.4	287.1855	di/tripeptide	N
366	YE	288.2036	1	1.989775	104161.3	287.1963	di/tripeptide	N
367	YE	290.1352	1	2.024842	157413.7	289.1279	di/tripeptide	N
368	YE	290.1716	1	2.196242	765013.5	289.1643	di/tripeptide	N
369	YE	292.1299	1	16.43819	186322.5	291.1226	di/tripeptide	N
370	YE	295.13	1	12.6764	208297.7	294.1228	di/tripeptide	N
371	YE	295.1662	1	14.63085	170181.7	294.1589	di/tripeptide	N
372	YE	295.1664	1	13.45718	317679.4	294.1591	di/tripeptide	N
373	YE	297.1089	1	2.735192	196501.6	296.1016	di/tripeptide	N
374	YE	300.1929	1	13.43245	216356.1	299.1857	di/tripeptide	N
375	YE	302.1717	1	2.298183	138308.2	301.1644	tri/oligopeptic	N
376	YE	302.2085	1	15.83851	118239.7	301.2012	tri/oligopeptic	N
377	YE	304.1665	1	19.91812	147463.7	303.1593	tri/oligopeptic	N
378	YE	304.1873	1	2.196242	305329.2	303.18	tri/oligopeptic	N
379	YE	304.1875	1	2.612583	134034	303.1802	tri/oligopeptic	N
380	YE	304.1878	1	6.941133	135156.7	303.1805	tri/oligopeptic	N
381	YE	304.1878	1	11.43273	160857.7	303.1805	tri/oligopeptic	N
382	YE	306.1302	1	1.890767	131380.7	305.1229	tri/oligopeptic	N
383	YE	306.1459	1	10.83633	127156.4	305.1386	tri/oligopeptic	N
384	YE	306.1666	1	2.196242	201778.3	305.1593	tri/oligopeptic	N
385	YE	310.1403	1	2.184975	102666.5	309.133	tri/oligopeptic	N
386	YE	311.1246	1	2.804983	142599.8	310.1173	tri/oligopeptic	N
387	YE	316.1875	1	2.3902	103795.5	315.1803	tri/oligopeptic	N
388	YE	316.1877	1	2.712283	118669.6	315.1804	tri/oligopeptic	N
389	YE	316.1879	1	11.29463	159799.9	315.1807	tri/oligopeptic	N
390	YE	316.188	1	13.0767	111033.3	315.1807	tri/oligopeptic	N
391	YE	316.2243	1	14.84729	222396.1	315.217	tri/oligopeptic	N
392	YE	316.2243	1	19.59787	238667.6	315.2171	tri/oligopeptic	N
393	YE	317.1826	1	2.196242	302640.2	316.1753	tri/oligopeptic	N
394	YE	318.203	1	2.196242	193894.8	317.1957	tri/oligopeptic	N
395	YE	318.2032	1	2.367633	121975.9	317.1959	tri/oligopeptic	N
396	YE	318.2034	1	14.62357	157099	317.1961	tri/oligopeptic	N
397	YE	320.1252	1	17.66828	183604.4	319.118	tri/oligopeptic	N
398	YE	320.146	1	2.173933	256418.4	319.1387	tri/oligopeptic	N
399	YE	328.2242	1	16.9083	419516.7	327.217	tri/oligopeptic	N
400	YE	328.2244	1	18.07284	120656.9	327.2171	tri/oligopeptic	N
401	YE	328.2244	1	17.66214	116055	327.2171	tri/oligopeptic	N
402	YE	328.2342	1	15.4886	104249.5	327.2269	tri/oligopeptic	N
403	YE	330.24	1	17.43218	110360.3	329.2327	tri/oligopeptic	N
404	YE	331.1984	1	2.207625	161045.8	330.1911	tri/oligopeptic	N
405	YE	332.1828	1	3.423267	117695.7	331.1755	tri/oligopeptic	N
406	YE	332.1828	1	9.842183	108398.2	331.1756	tri/oligopeptic	N
407	YE	332.1829	1	11.67633	142268.1	331.1756	tri/oligopeptic	N
408	YE	332.2191	1	14.52533	130355.1	331.2118	tri/oligopeptic	N
409	YE	332.2193	1	19.45861	136398.6	331.212	tri/oligopeptic	N
410	YE	333.1775	1	2.196242	305090	332.1702	tri/oligopeptic	N
411	YE	334.1252	1	1.923833	101175.9	333.1179	tri/oligopeptic	N
412	YE	334.141	1	13.78176	140854.8	333.1338	tri/oligopeptic	N

413	YE	334.162	1	5.237692	181352.1	333.1548	tri/oligopeptic	N
414	YE	334.1775	1	18.0397	121355.1	333.1702	tri/oligopeptic	N
415	YE	334.1978	1	2.207625	125766.9	333.1905	tri/oligopeptic	N
416	YE	342.2403	1	21.27968	129255.6	341.233	tri/oligopeptic	N
417	YE	342.2403	1	20.73499	194365.5	341.2331	tri/oligopeptic	N
418	YE	343.199	1	14.79712	131916.2	342.1917	tri/oligopeptic	N
419	YE	345.2142	1	2.196242	107205.3	344.2069	tri/oligopeptic	N
420	YE	345.2145	1	14.80333	391403.8	344.2072	tri/oligopeptic	N
421	YE	345.2146	1	15.60019	227379.8	344.2073	tri/oligopeptic	N
422	YE	345.2146	1	11.48936	156298.8	344.2073	tri/oligopeptic	N
423	YE	346.1983	1	2.4992	131386.1	345.191	tri/oligopeptic	N
424	YE	346.1985	1	17.08503	379361.5	345.1912	tri/oligopeptic	N
425	YE	346.1985	1	16.50322	176558.7	345.1913	tri/oligopeptic	N
426	YE	346.1987	1	16.17463	123848.1	345.1914	tri/oligopeptic	N
427	YE	346.1988	1	12.82072	213356.9	345.1915	tri/oligopeptic	N
428	YE	346.2352	1	19.19771	115144.2	345.2279	tri/oligopeptic	N
429	YE	347.1569	1	2.048192	151138.1	346.1497	tri/oligopeptic	N
430	YE	348.1773	1	2.196242	543947.2	347.1701	tri/oligopeptic	N
431	YE	348.1777	1	2.874758	139812.7	347.1705	tri/oligopeptic	N
432	YE	359.2303	1	14.31116	160863.6	358.223	tri/oligopeptic	N
433	YE	359.2305	1	18.53333	110934.2	358.2232	tri/oligopeptic	N
434	YE	360.2142	1	20.45641	352057.8	359.2069	tri/oligopeptic	N
435	YE	360.2142	1	19.86595	103242.5	359.2069	tri/oligopeptic	N
436	YE	360.2143	1	16.87777	146211	359.207	tri/oligopeptic	N
437	YE	360.2144	1	15.0582	105957.8	359.2071	tri/oligopeptic	N
438	YE	360.2144	1	20.11532	100928.9	359.2072	tri/oligopeptic	N
439	YE	360.2145	1	21.26401	118149.2	359.2072	tri/oligopeptic	N
440	YE	361.1725	1	2.196242	302069.5	360.1653	tri/oligopeptic	N
441	YE	361.2089	1	2.196242	164818.3	360.2016	tri/oligopeptic	N
442	YE	362.1567	1	2.286783	136090.5	361.1494	tri/oligopeptic	N
443	YE	362.193	1	2.286783	263646.8	361.1858	tri/oligopeptic	N
444	YE	364.1359	1	1.923833	105016.5	363.1286	tri/oligopeptic	N
445	YE	374.2041	1	2.196242	198733	373.1969	tri/oligopeptic	N
446	YE	376.1729	1	9.66725	138050	375.1656	tri/oligopeptic	N
447	YE	377.1867	1	15.57564	108639	376.1794	tri/oligopeptic	N
448	YE	377.2039	1	2.207625	140343.9	376.1967	tri/oligopeptic	N
449	YE	385.2462	1	19.28834	131027.8	384.2389	tri/oligopeptic	N
450	YE	387.2616	1	16.28014	117652.4	386.2543	tri/oligopeptic	N
451	YE	389.2038	1	2.207625	174556.2	388.1966	tri/oligopeptic	N
452	YE	391.2198	1	2.207625	136615	390.2125	tri/oligopeptic	N
453	YE	391.2859	1	0.14335	653230.9	390.2786	tri/oligopeptic	N
454	YE	399.2619	1	22.64109	132470	398.2546	tri/oligopeptic	N
455	YE	400.2199	1	2.219092	106093.9	399.2126	tri/oligopeptic	N
456	YE	404.2143	1	2.184975	122509.4	403.207	tri/oligopeptic	N
457	YE	405.1989	1	2.207625	253734.2	404.1916	tri/oligopeptic	N
458	YE	415.2357	1	27.25169	219948	414.2284	tri/oligopeptic	N
459	YE	417.2359	1	14.38291	109890.8	416.2287	tri/oligopeptic	N
460	YE	418.1939	1	2.083283	137781.4	417.1866	tri/oligopeptic	N
461	YE	421.2129	1	15.78793	160880.5	420.2056	tri/oligopeptic	N
462	YE	427.2933	1	21.15384	108636.3	426.2861	tri/oligopeptic	N

463	YE	429.2722	1	15.15354	248515.3	428.265	tri/oligopeptic	N
464	YE	431.2879	1	15.98033	127384.6	430.2806	tri/oligopeptic	N
465	YE	432.2096	1	2.184975	150105.9	431.2023	tri/oligopeptic	N
466	YE	436.3435	1	35.94663	434376.7	435.3362	tri/oligopeptic	N
467	YE	436.3439	1	0.018492	261848.8	435.3366	tri/oligopeptic	N
468	YE	442.2675	1	19.03069	322411.1	441.2602	tri/oligopeptic	N
469	YE	446.2252	1	2.252975	186807.4	445.218	tri/oligopeptic	N
470	YE	457.2672	1	15.85853	238630.5	456.2599	tri/oligopeptic	N
471	YE	462.2203	1	2.196242	191625.8	461.213	tri/oligopeptic	N
472	YE	463.257	1	22.75768	120386.1	462.2497	tri/oligopeptic	N
473	YE	470.2992	1	21.89658	103127.2	469.2919	tri/oligopeptic	N
474	YE	476.2357	1	2.184975	100291.3	475.2285	tri/oligopeptic	N
475	YE	488.3096	1	20.26623	107536.8	487.3024	tri/oligopeptic	N
476	YE	492.2106	1	19.09788	130764.6	491.2033	tri/oligopeptic	N
477	YE	502.2888	1	17.4033	137952.1	501.2815	tri/oligopeptic	N
478	YE	503.2842	1	15.2452	112502.9	502.2769	tri/oligopeptic	N
479	YE	504.268	1	14.3971	127801.7	503.2607	tri/oligopeptic	N
480	YE	506.2296	1	11.5715	209447	505.2223	tri/oligopeptic	N
481	YE	518.2994	1	24.41252	154083.5	517.2921	tri/oligopeptic	N
482	YE	524.3464	1	23.75523	156540.2	523.3391	tri/oligopeptic	N
483	YE	529.2791	1	27.91106	161922.2	528.2718	tri/oligopeptic	N
484	YE	543.3156	1	19.69556	132602.1	542.3084	tri/oligopeptic	N
485	YE	548.31	1	24.50338	115205.3	547.3027	tri/oligopeptic	N
486	YE	605.2958	1	24.53668	196717.3	604.2885	tri/oligopeptic	N
487	YE	310.183	2	20.08391	132539.4	618.3514	oligopeptide	N
488	YE	343.2171	2	16.31572	324690.8	684.4196	oligopeptide	N
489	YE	686.4108	1	19.0076	111856.6	685.4035	oligopeptide	N
490	YE	708.5135	1	37.28652	139446.8	707.5062	oligopeptide	N
491	YE	378.2202	2	23.00203	117583.1	754.4259	oligopeptide	N
492	YE	393.2311	2	19.78028	100985.3	784.4477	oligopeptide	N
493	YE	403.2192	2	18.8692	211326	804.4238	oligopeptide	N
494	YE	417.2312	2	22.98896	185628.6	832.4478	oligopeptide	N
495	YE	424.7364	2	16.98438	130106.7	847.4583	oligopeptide	N
496	YE	476.2684	2	22.56827	116293.4	950.5223	oligopeptide	N
497	YE	684.9278	2	1.594167	105072.7	1367.841	oligopeptide	N

Supplementary figure 3. Shows Bayesian Information Criterion (BIC) analysis of the proteomic data set. Showing EEV as the best model when using 3 different clusters.

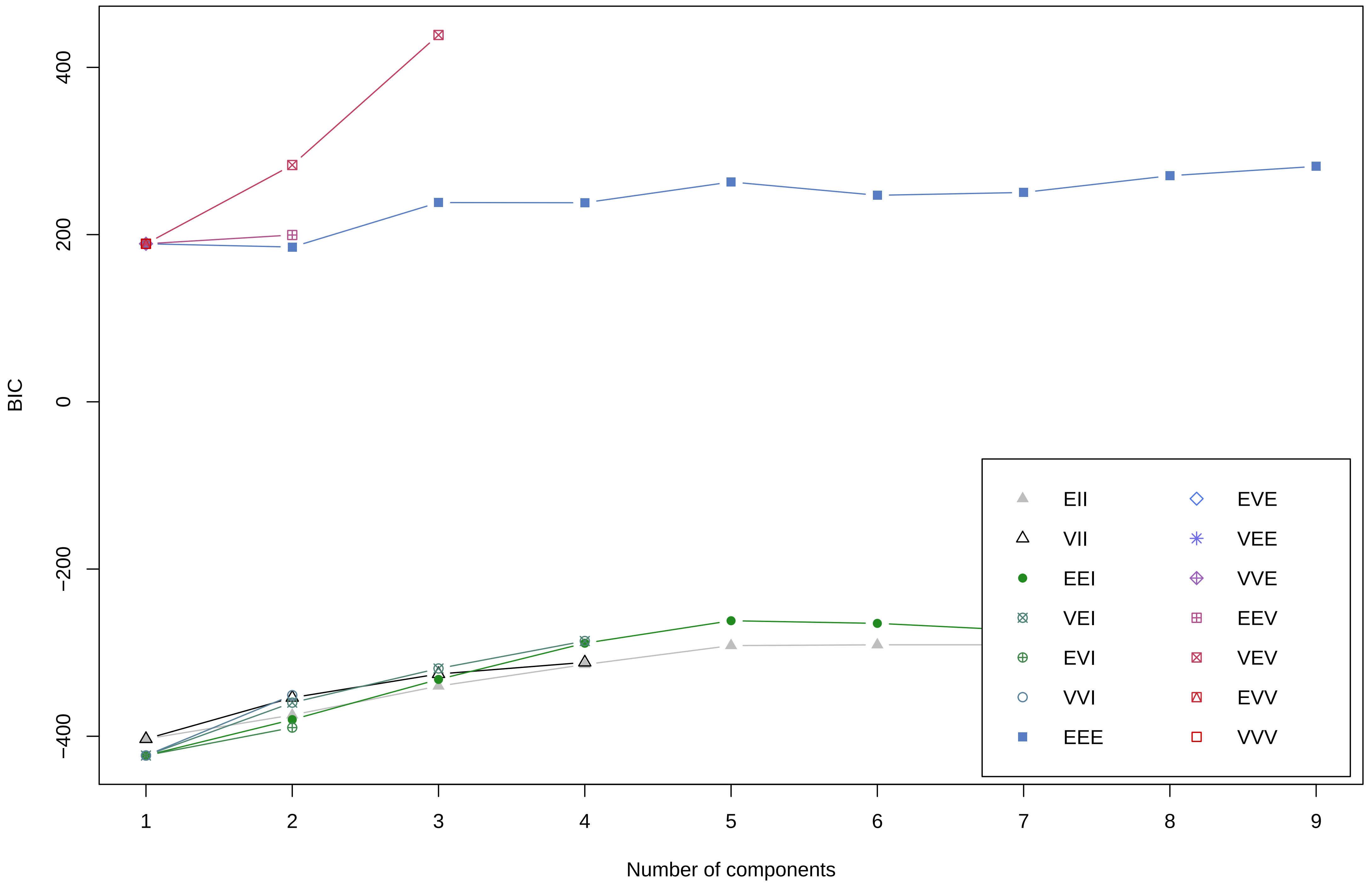

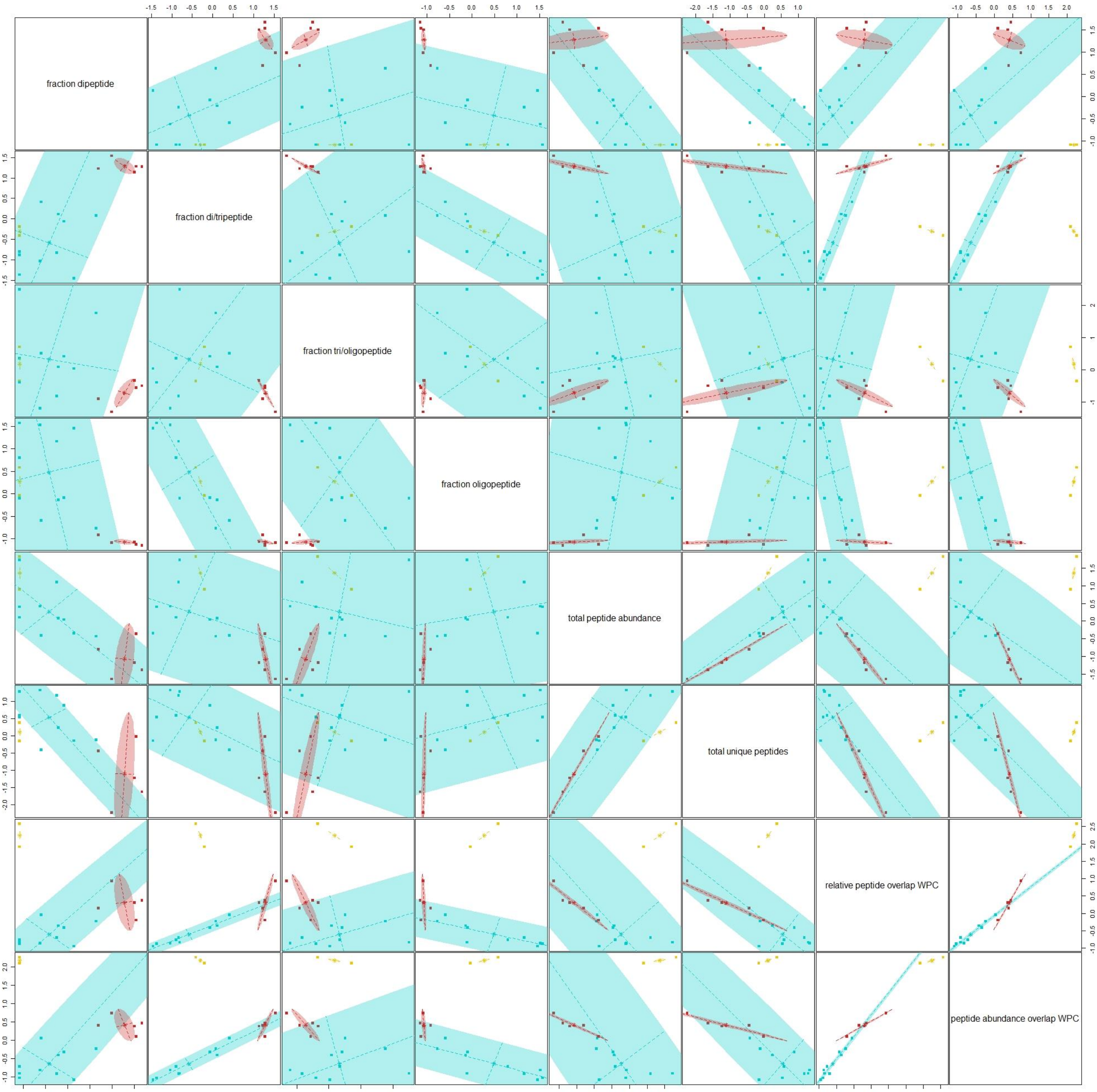

Supplementary figure 4, all varieties of two component plots showing the protein clustering based on proteomic data. Protein clustering was achieved based on the statistical cluster model; ellipsoidal equal volume, shape and orientation (EEV). Considered data were peptide fractions, peptide abundance, total amount of unique peptides, relative overlap with whey protein concentrate (WPC) and the relative peptide abundance of the WPC overlap.

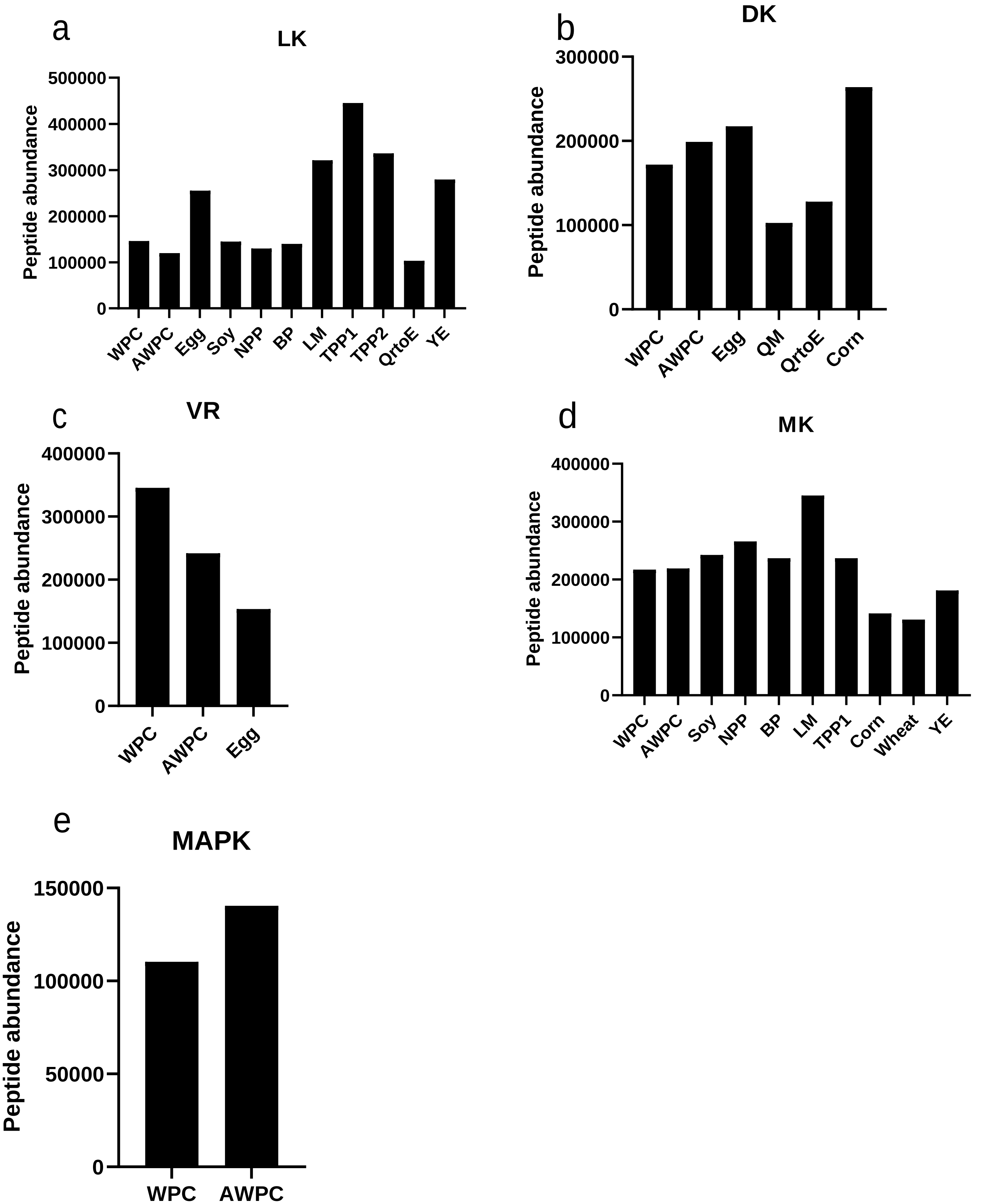

Supplementary figure 5. Abundance of identified peptide sequences with a de novo score >90. A) dipeptide leusine-lysine, B) dipeptide aspartic acid-lysine, C) dipeptide valine-arginine, D) dipeptide methionine-lysine and E) oligopeptide methionine-alanine-proline-lysine.

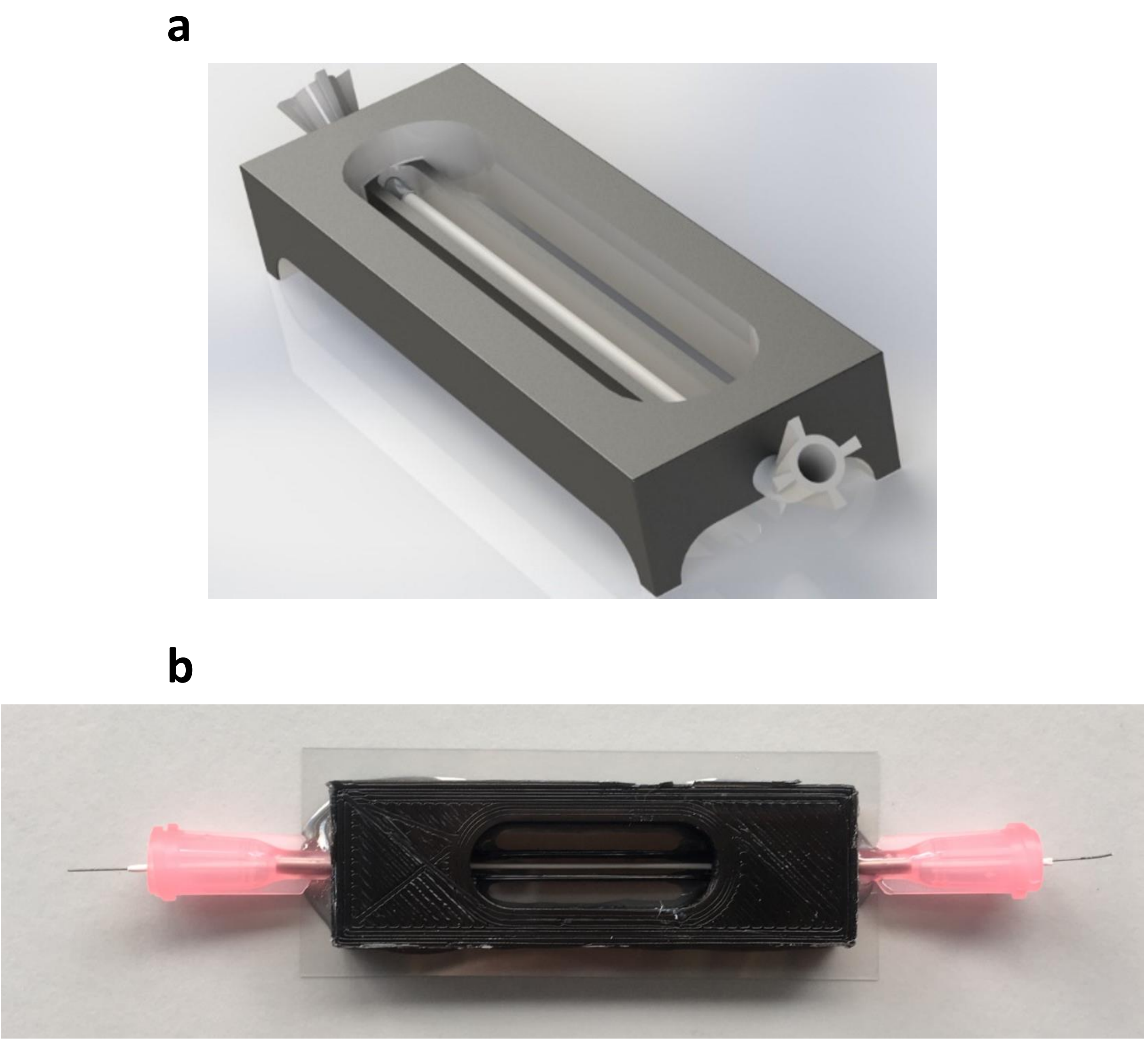

Supplementary figure 6. Image of Bioengineered Intestinal Tubules. A) schematic representation of the bioengineered intestinal tubules model, b) photo of a bioengineered intestinal tubules before coating and seeding.

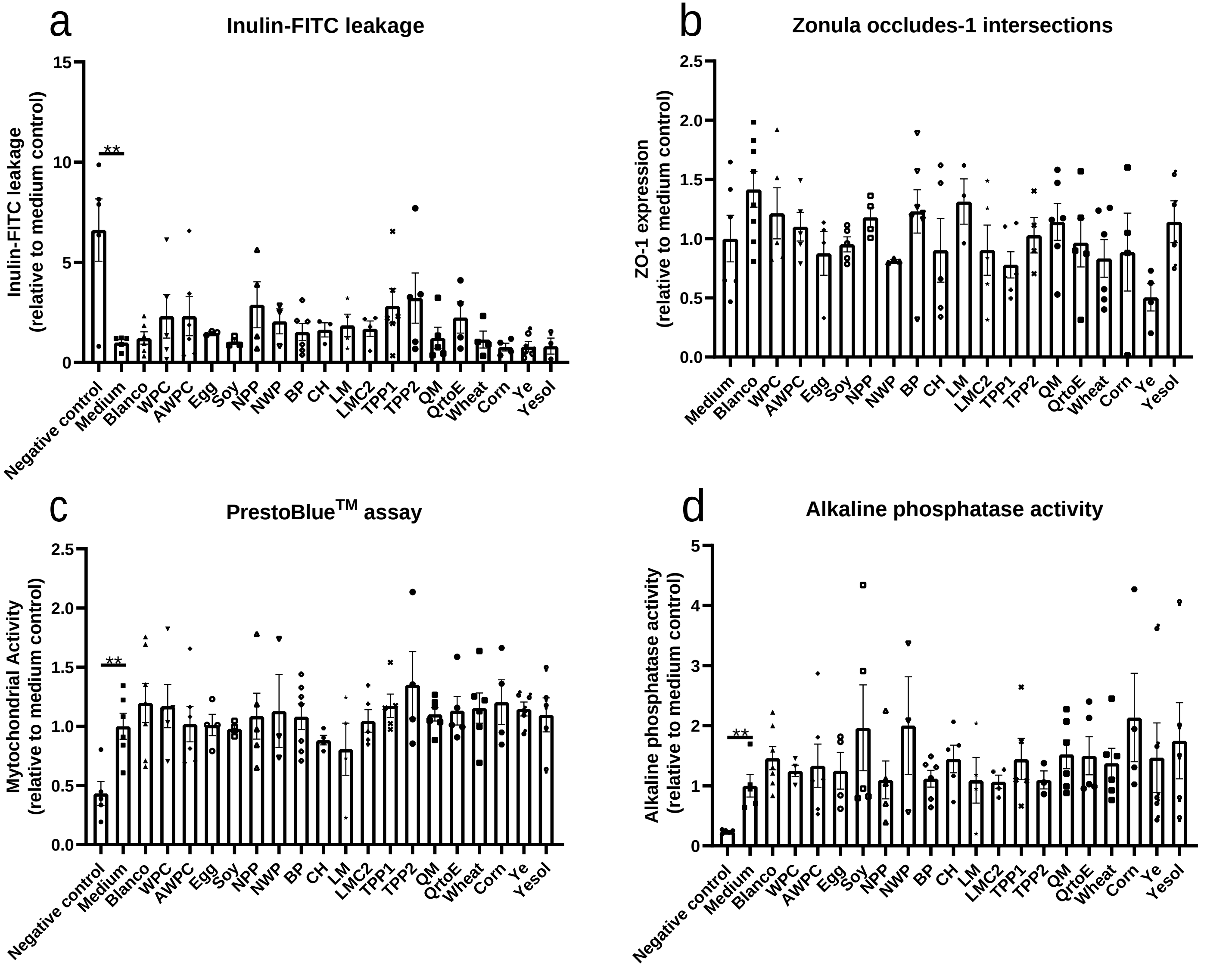

Supplementary figure 7. Bioengineered intestinal tubules were exposed to potential dietary proteins assessing the effect on epithelial barrier, viability and alkaline phosphatase activity. Experiment ready bioengineered intestinal tubules were exposed to in vitro digested proteins (diluted 1:4 in cultivation medium) for 3 hours. As measure for epithelial barrier integrity, inulin-FITC leakage (A) and zonula occludens-1 intersects corrected for surface area and number of nuclei (B) were quantified. As measure for cell viability mitochondrial activity was assessed (C) and for brush border enzyme activity alkaline phosphatase activity was assessed (D). Values of C and D were corrected for bioengineered intestinal tubule length. Data are presented relative to positive control (experiment ready bioengineered intestinal tubule exposed to culture medium), data are presented as mean ± SEM of n=3-7 independent experiments, corrected for outliers (1 outliers of 400 data points) and tested for significance using one-way ANOVA and student T-test, ** $P < 0.005$.

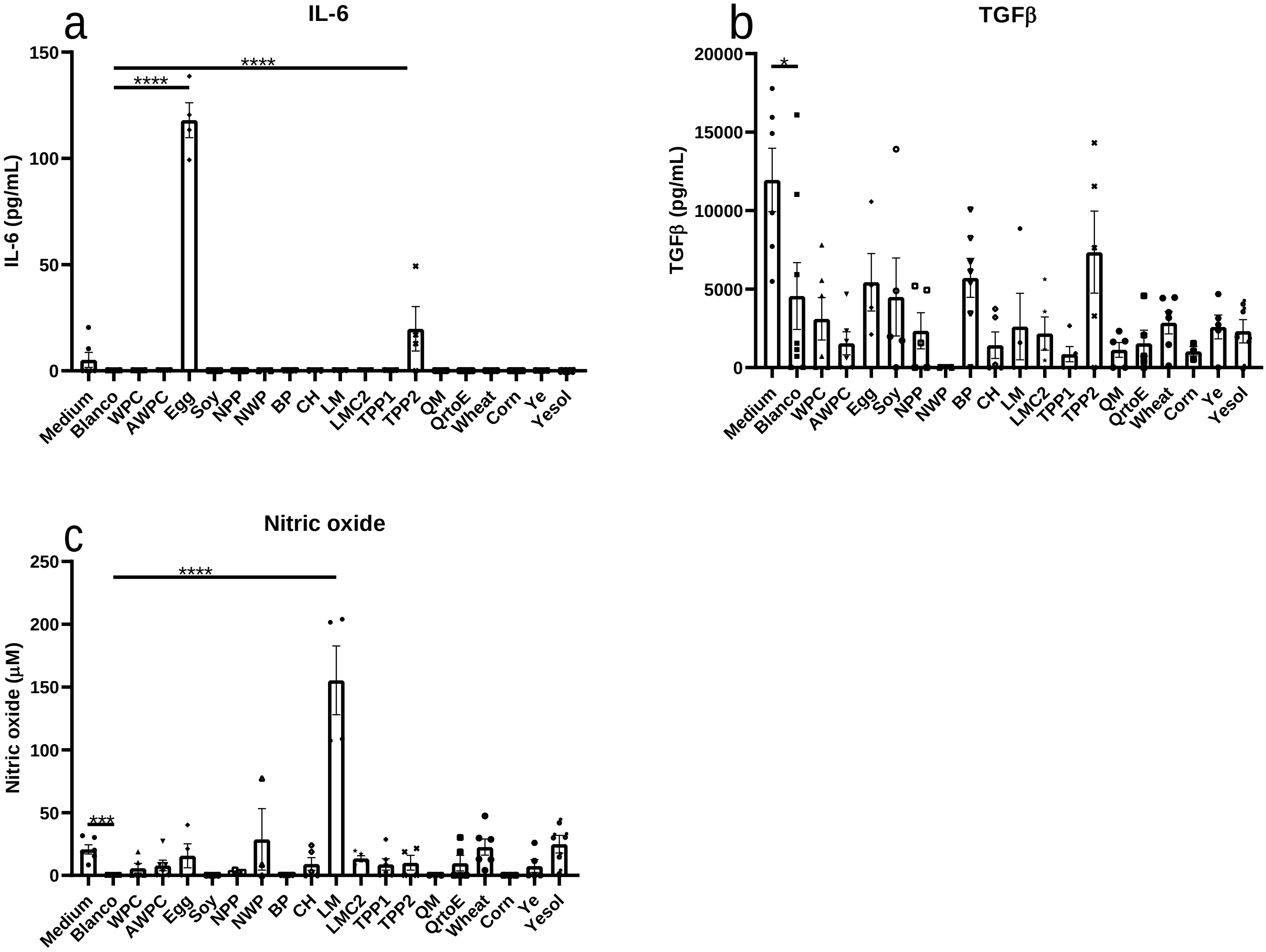

Supplementary figure 8. Bioengineered intestinal tubules were exposed to potential dietary proteins assessing the effect on IL-6, TGF**β** and nitric oxide (NO) secretion. Experiment ready bioengineered intestinal tubules were exposed to in vitro digested proteins (diluted 1:4 in cultivation medium) for 3 hours. Thereafter, supernatant was collected and analyzed for, IL-6 (A), TGF-β (B), NO (C). Values were corrected for basal levels in culture medium of negative control (bioengineered intestinal tubule without cells). Data are presented as mean ± SEM of at least 3-7 independent experiments, corrected for outliers (12 outliers of 306 data points) and tested for significance using one-way ANOVA, * P < 0.05, *** P < 0.001, **** P < 0.0001.

Supplementary figure 9. Shows Bayesian Information Criterion (BIC) analysis of the in vitro biological efficacy data. Showing diagonal equal volume and shape as the best model when using 6 different clusters.

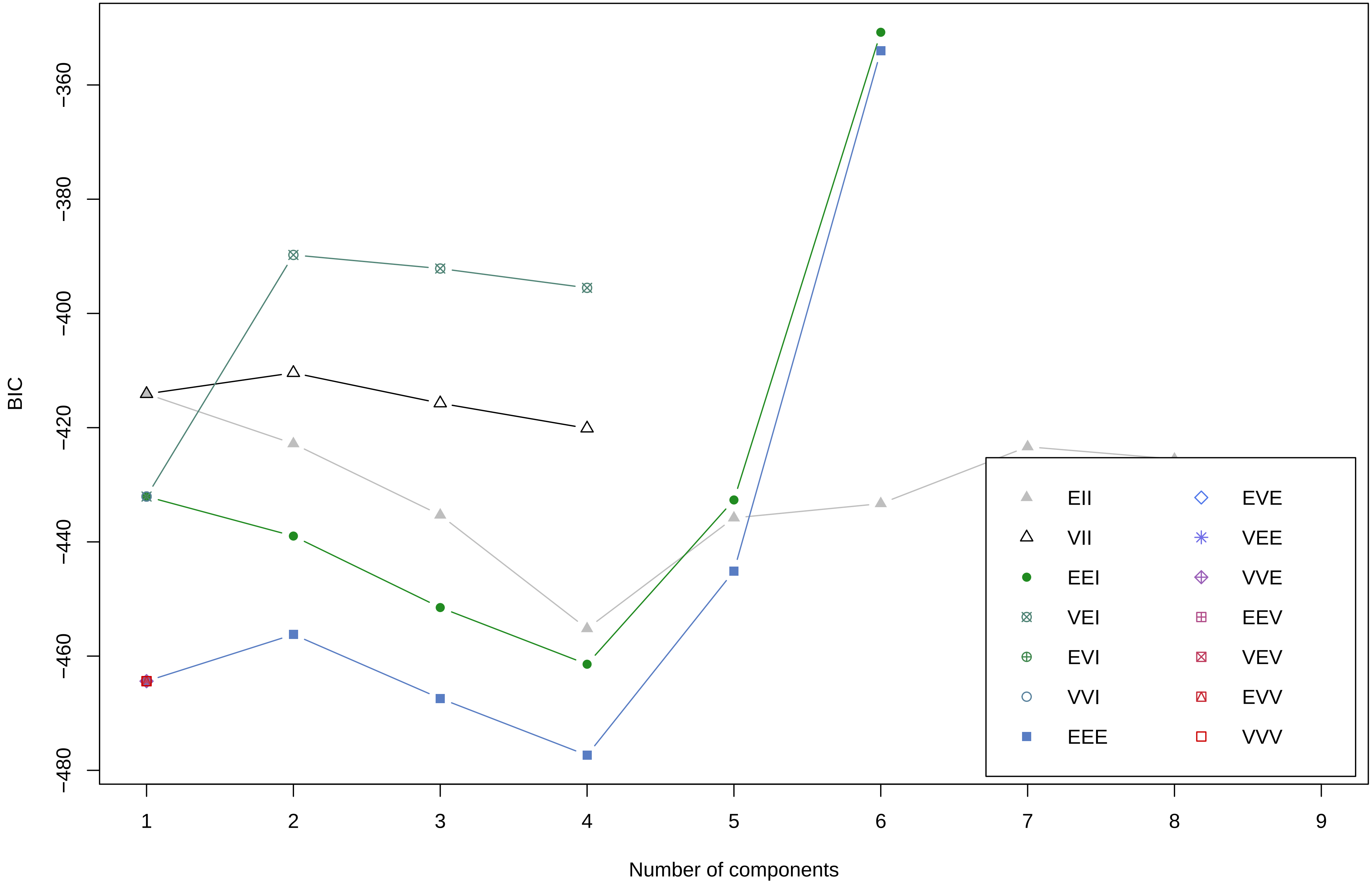

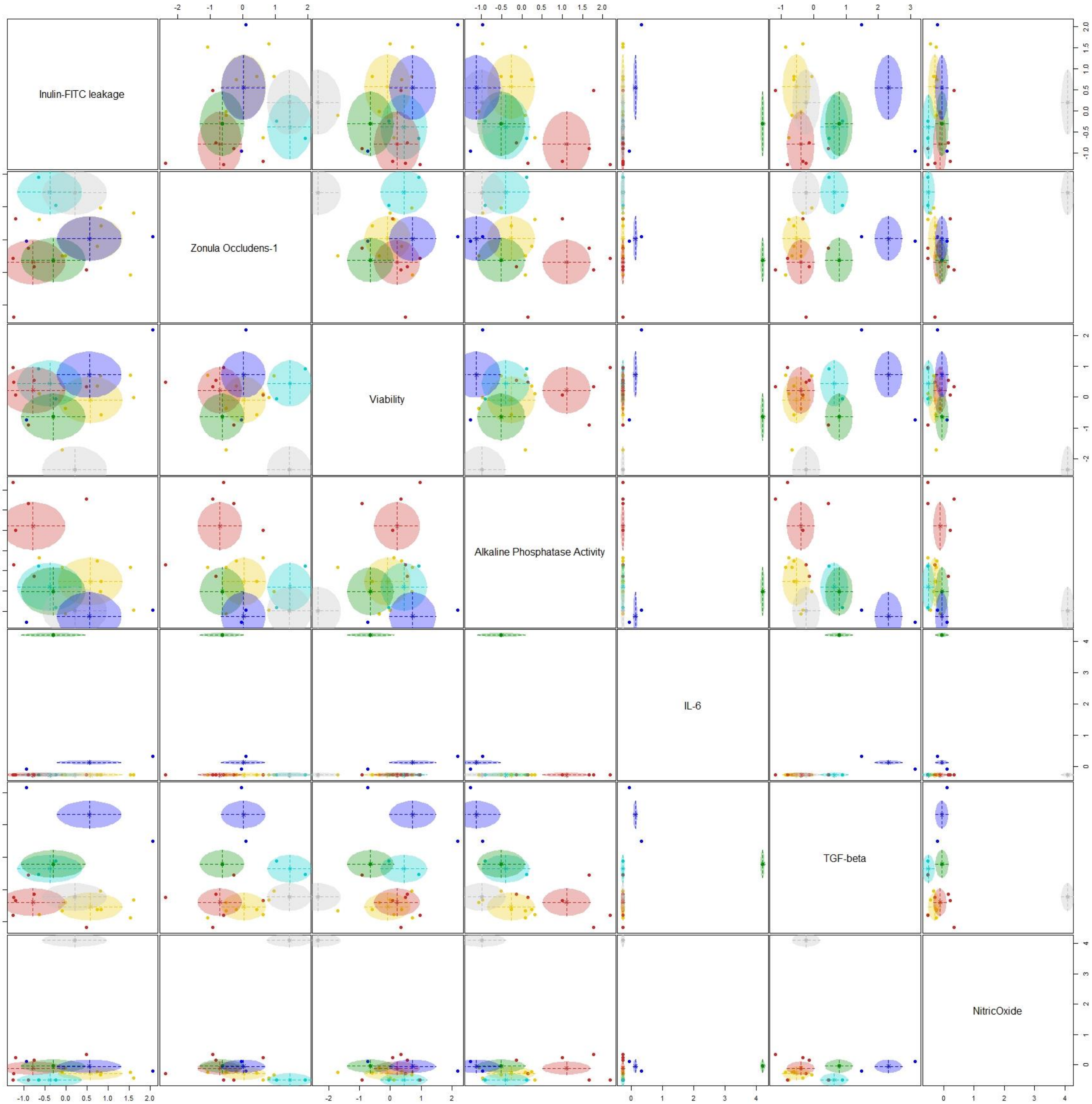

Supplementary figure 10. All varieties of two component plots based on *in vitro* biological efficacy data. Protein clustering was achieved based on the statistical cluster model; equal volume and shape axis parallel orientation (EEI). Considered data were Inulin-FITC leakage, Zonula occludens-1 quantification, cell viability, alkaline phosphatase activity, IL-6, TGFβ and nitric oxide.

Supplementary figure 11. Shows Bayesian Information Criterion (BIC) analysis of both proteomic and in vitro biological efficacy data. Showing ellipsoidal equal volume shape and orientation (EEE) as the best model when using 5 different clusters.

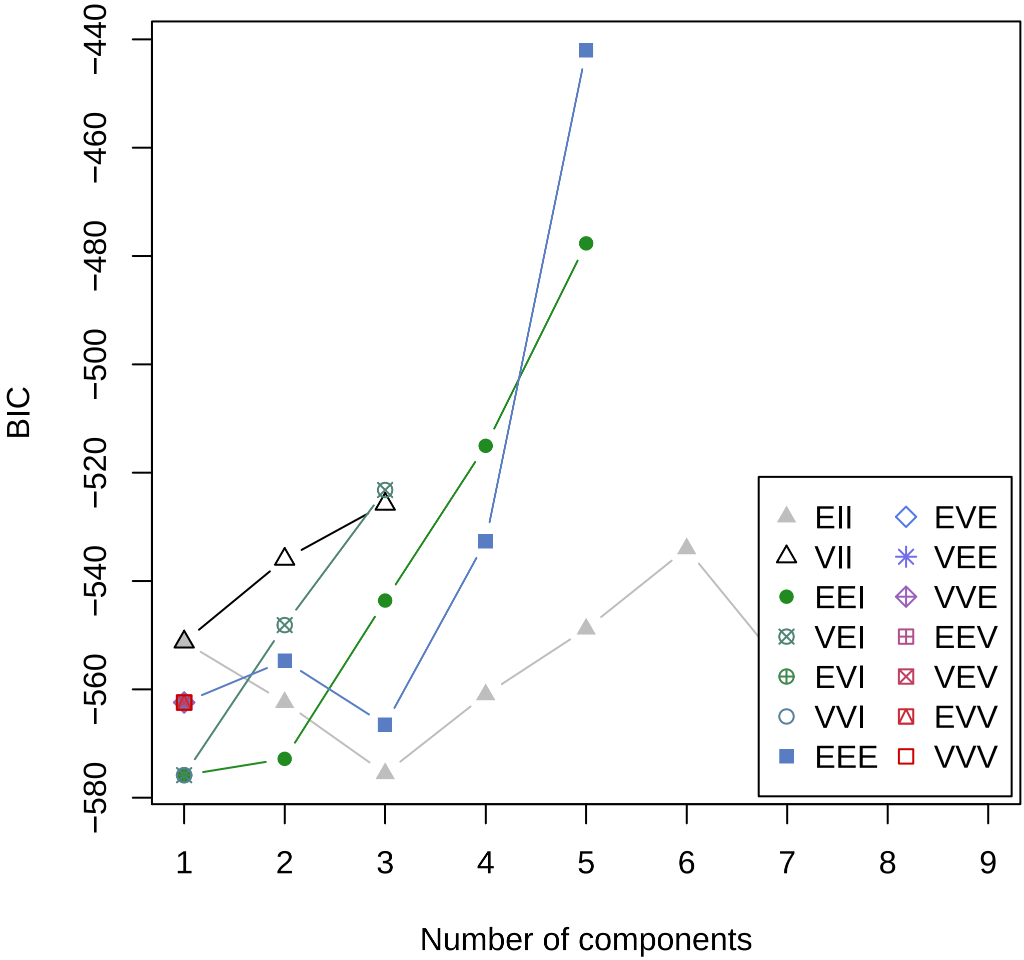

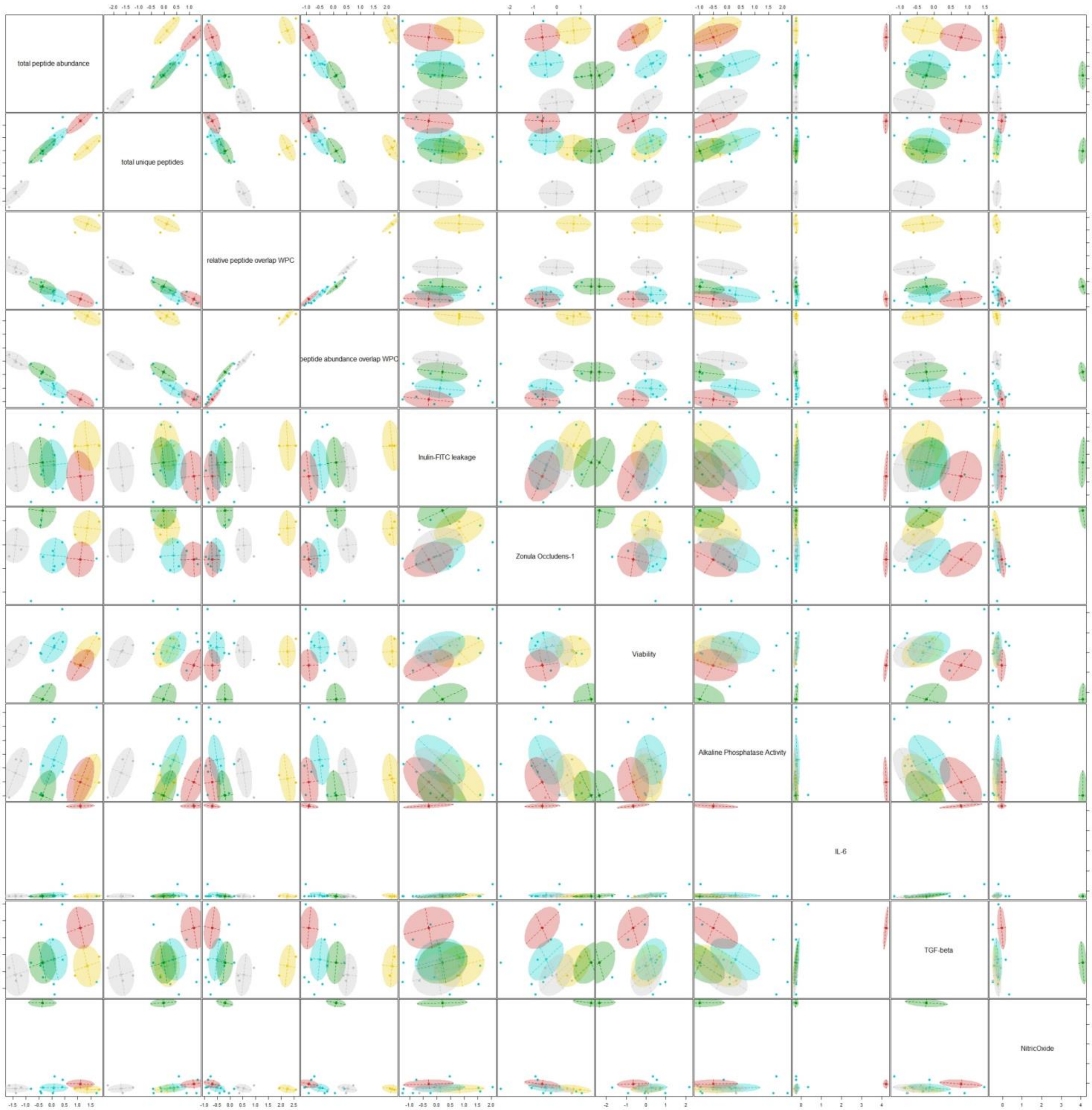

Supplementary figure 12. All varieties of two component plots based on proteomic and *in vitro* biological efficacy data. Protein clustering was achieved based on the statistical cluster model; ellipsoidal equal volume, shape and orientation (EEE) with 5 distinct clusters. Considered data were peptide abundance, total amount of unique peptides, relative overlap with whey protein concentrate (WPC), the relative peptide abundance of the WPC overlap, Inulin-FITC leakage, Zonula occludens-1 quantification, cell viability, alkaline phosphatase activity, IL-6, TGFβ and nitric oxide.

Supplementary figure 13, a schematic image of the 3-dimensional printed chamber including dimensions in mm.

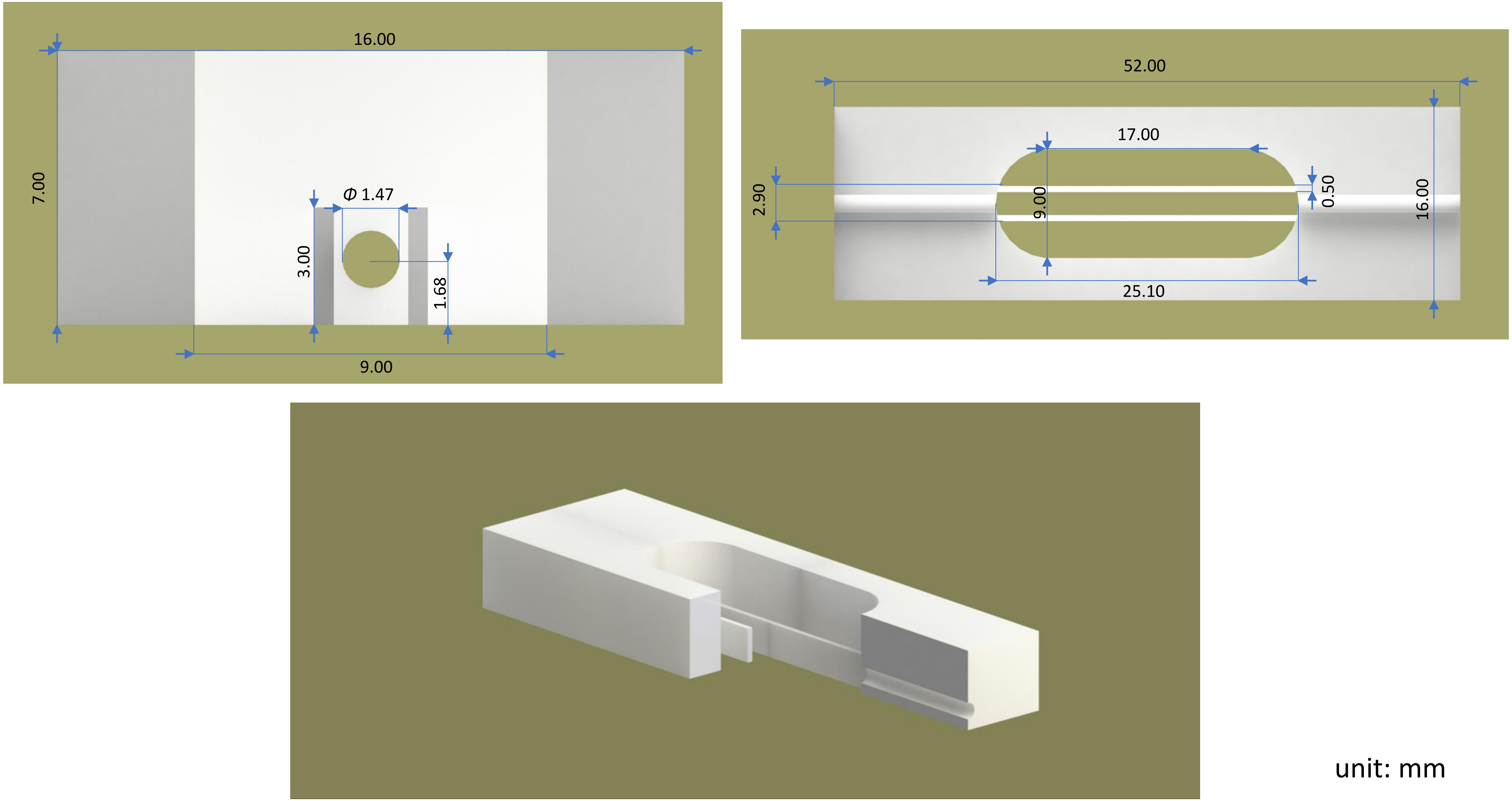

Supplementary Methods 1. Step by step protocol for zonula occludens-1 quantification

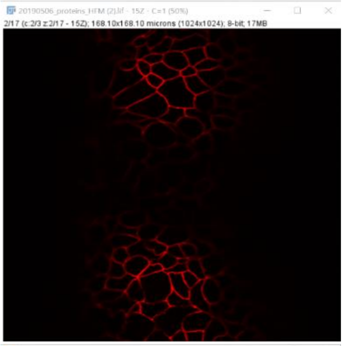

A single image of a bioengineered intestinal tubule Z-stack of the Zonula Occludens-1 network

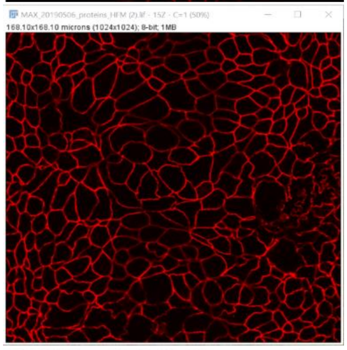

Maximum intensity projection (MIP) created from the Z-stack. Noise level was determined with the plot profile function

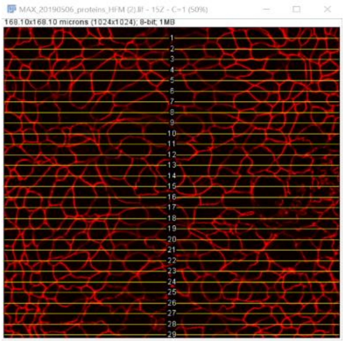

A custom made ROI SET is applied over the image. The ROI SET consists of 29 horizontal lines evenly distributed over the full image

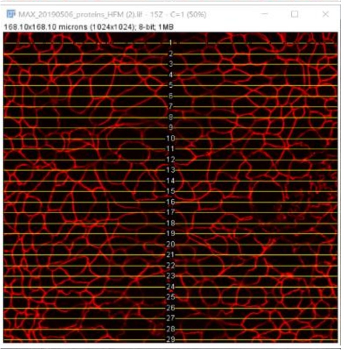

Peakfinder plugin is activated and tolerance is set to 2x noise level. First line is selected with the ROI manager and peakfinder displays a handle for each intersect on the line

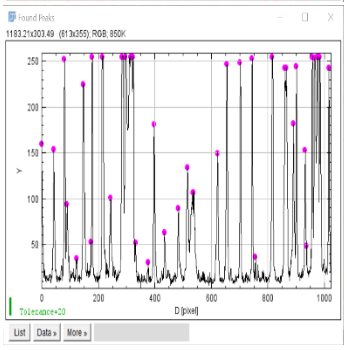

Peakfinder creates a graph displaying peaks for each intersect

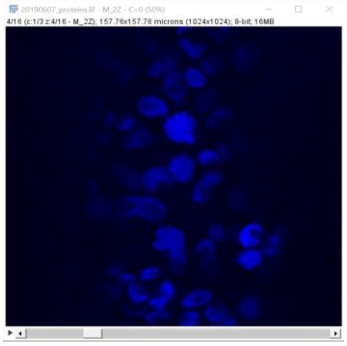

A single image of a bioengineered intestinal tubule Z-stack of the nuclei

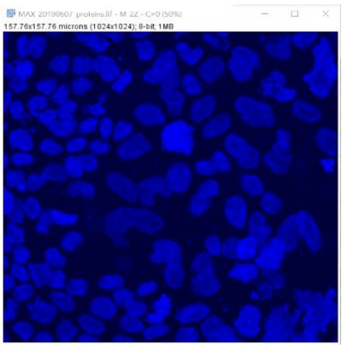

Maximum intensity projection (MIP) created from the Z-stack. This image is then used for counting nuclei

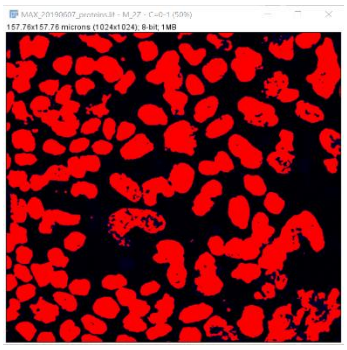

The threshold is determined and applied using Otsu method

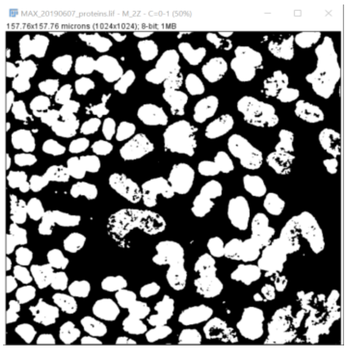

Imaged including threshold limit is converted to a binary image and the watershed function is applied

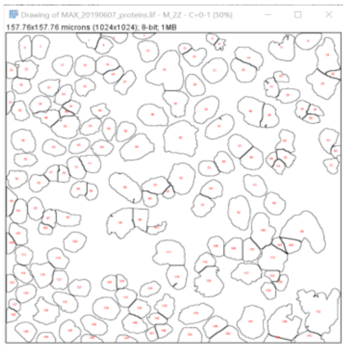

The nuclei are counted with the analyse particle function
